# Natural Enantiomers: Occurrence, Biogenesis and Biological Properties

**DOI:** 10.3390/molecules27041279

**Published:** 2022-02-14

**Authors:** Jin-Hai Yu, Zhi-Pu Yu, Robert J. Capon, Hua Zhang

**Affiliations:** 1 School of Biological Science and Technology, University of Jinan, Jinan 250022, China; yujinhai12@sina.com (J.-H.Y.); yuzhipu911108@163.com (Z.-P.Y.); 2 Institute for Molecular Bioscience, The University of Queensland, St. Lucia, Brisbane, QLD 4072, Australia

**Keywords:** enantiomers, natural products, biogenesis, biological properties

## Abstract

The knowledge that natural products (NPs) are potent and selective modulators of important biomacromolecules (e.g., DNA and proteins) has inspired some of the world’s most successful pharmaceuticals and agrochemicals. Notwithstanding these successes and despite a growing number of reports on naturally occurring pairs of enantiomers, this area of NP science still remains largely unexplored, consistent with the adage “If you don’t seek, you don’t find”. Statistically, a rapidly growing number of enantiomeric NPs have been reported in the last several years. The current review provides a comprehensive overview of recent records on natural enantiomers, with the aim of advancing awareness and providing a better understanding of the chemical diversity and biogenetic context, as well as the biological properties and therapeutic (drug discovery) potential, of enantiomeric NPs.

## 1. Introduction

Natural products (NPs) are usually regarded as small molecule organic compounds which are produced in the metabolic processes of living organisms [1]. Although studies on NPs have informed many areas of science, industry and commerce, including flavorings, perfumes, cosmeceuticals and nutraceuticals, arguably, their most important contribution to society has been as pharmaceuticals and agrochemicals [2]. For example, NPs and NP-inspired chemical entities still account for more than two thirds of all the drugs approved by Food and Drug Administration (FDA) in the USA in roughly the past four decades [2].

The vast majority of reported NPs are chiral molecules that exist in nature as single enantiomers [3]. However, as the adage goes, “Beware of exceptions to the rule”; indeed, there is increasing evidence that both enantiomers of selected NPs exist in nature. Surprisingly, NPs were generally believed to exist as single enantiomers until the 1970s, despite reports of several exceptions, probably owing to the standpoint of the famous French chemist/microbiologist Louis Pasteur, i.e., that life processes were asymmetrical [4]. Benefiting from scientific and technical advances in our understanding of NP biosynthesis, there is increasing acceptance and documentation of the occurrence of natural enantiomers. Finefield et al. reported this trend in a 2012 review, documenting the occurrence and biogenesis (where applicable) of the well-known NP enantiomers reported before 2012 [3].

During our research into bioactive NPs from medicinal plants and other sources, we have regularly encountered NP enantiomers and have documented differences in their bioactivities [5,6,7,8,9]. Surveying the scientific literature revealed the aforementioned report by Finefield et al. as the only systematic record of the occurrence of natural enantiomers [3], supported by a 2018 review by Cass et al. on the techniques for separation and absolute configuration (abs. config.) assignment of enantiomeric NPs [10]. This survey also revealed a dramatic increase in the number of publications on natural enantiomers, especially in the last few years. Against this background, the present review seeks to summarize advances in this fascinating field over the period of January 2012 to December 2019.

## 2. Enantiomers from Kingdom Plantae

The kingdom Plantae is an important part of nature, providing rich resources and a beautiful environment for human beings. In the field of medicine, various plants have served as the basis of traditional herbal medication to treat a variety of diseases for thousands of years. Phytochemical research on herbs has provided thousands of structural models or leads for modern drug discovery, and some NPs can even be used directly as drugs, such as taxol. NPs derived from plants have been well studied for decades, and a comprehensive system of classification has been devised. On the other hand, new NPs from kingdom Plantae are being identified all the time due to the abundance of resources. Accordingly, enantiomers produced by plants occupy the vast majority of enantiomeric NPs from natural sources.

In this section, natural enantiomers from kingdom Plantae will be classified into fourteen subcategories on the basis of their structural type, i.e., lignans, coumarins, simple phenylpropanoids, alkaloids, flavonoids, terpenoids, phloroglucinols, naphthalene and phenanthrenes, chromanes, acetophenones, diarylheptanoids, triphenylmethanes, fatty acid and miscellaneous. Where appropriate, their biogenesis and structure will also be described.

### 2.1. Lignans

Lignans are a common class of NPs which is widely distributed in the plant kingdom and which exhibits a broad spectrum of bioactivities including antioxidant, antitumor, anti-inflammatory, antineurodegenerative, antiviral and antimicrobial properties [11,12]. Lignans usually consist of two (sometimes three or even more) C_6_-C_3_ units (also known as phenylpropanoids). Their structural diversity arises from the different degrees of oxidation, as well as various substitution and connection patterns. Consistent with IUPAC recommendations [13], lignans are normally divided into classical lignans (only direct 8,8′-connection between the two C_6_-C_3_ units), neolignans (non-8,8′ and direct connection between the two C_6_-C_3_ units), oxyneolignans (ether oxygen linkage between the two C_6_-C_3_ units), and higher lignans (above two C_6_-C_3_ units, e.g., sesquineolignans and dineolignans). However, this classification is suggested mainly as a means of clarifying the confusing lignan nomenclature in the past, and is far from sufficient to assort the vast number of natural lignans. In general, NP chemists tend to sort lignans according to their detailed structural types, such as dibenzylbutanes, arylnaphthalenes, benzofurans, etc. [14].

Based on structural features, and for the convenience of discussion, the lignan enantiomers in the period covered by this review are classified into three subcategories: acyclic lignans, cyclic lignans and sesquineolignans. Acyclic lignans refer to those without extra rings except for the existing aromatic rings in the phenylpropanoid units, whereas cyclic lignans possess additional rings. According to the reported compound numbers, acyclic lignans are further divided into 8-4′-oxyneolignans and other acyclic lignans, while cyclic lignans will be presented as furan-incorporating lignans and other cyclic lignans. In the interests of brevity, only the structure of one enantiomer of each pair is provided; this rule applies to all structural classes in the current review.

#### 2.1.1. Acyclic Lignans

**Figure 1:**
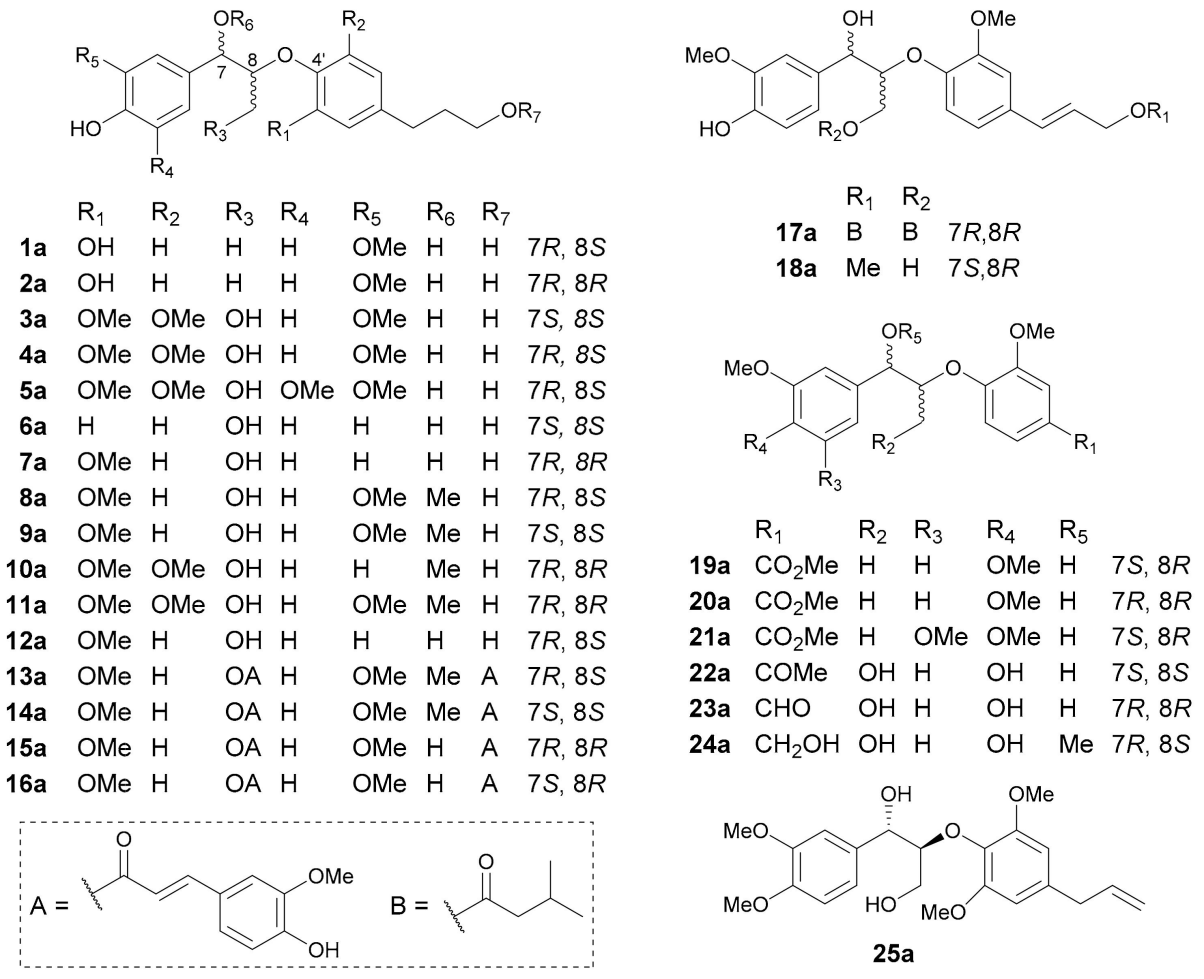
Structures of 8,4′-oxyneolignans.

*8,4′-Oxyneolignans.* 8,4′-Oxyneolignans (for structures, see Figure 1; for names, see Table S1 in Supplementary Materials) are formed via 8-*O*-4′ ether bonds. Also, the C-7 in these lignans is usually oxidized in a nonstereoselective manner. Then, *erythro*- or *threo*-isomers are generated, leading to the occurrence of two pairs of enantiomers.

The first reported cases in the period covered by this study were **1a**/**1b** and **2a**/**2b**, isolated from *Paeonia lactiflora* in 2015 [15]. Compounds **1** and **2** have the same constitutional structure but different relative configurations (rel. configs.). The *erythro* rel. config. for **1**, and the *threo* rel. config. for **2**, were determined via the *J*_7,8_ values (3.5 Hz for **1** vs. 6.8 Hz for **2**), while their absolute configurations (abs. configs.) were established by the time-dependent density functional theory electronic circular dichroism (TDDFT-ECD) method. In the same year, three dinorneolignan pairs **19a**/**19b**−**21a**/**21b**, along with a neolignan pair **25a**/**25b**, were reported and found as scalemic mixtures in *Acorus tatarinowii* [16]. The determination of their abs. configs. was based on ECD data analysis and the TDDFT-ECD method. A modified Mosher’s method was also used to further confirm the abs. configs. of **19a**/**19b**. In 2016, Gu and coworkers reported the presence of **13a**/**13b**−**16a**/**16b** in *Euphorbia sikkimensis* [17], among which **13a**/**13b** and **14a**/**14b** and their rel. configs. had been reported in 2001 [18]. In addition, compounds **13** and **14** are diastereoisomers, as is the case for **15** and **16**. In 2017, compounds **6a**/**6b** and **7a**/**7b** were isolated from *Rubus idaeus* [19]. In 2019, Song and colleagues discovered **3a**/**3b**−**5a**/**5b**, **22a**/**22b** and **23a**/**23b** from *Crataegus pinnatifida* [20], as well as **8a**/**8b**−**12a**/**12b** from *Ailanthus altissima* [21]. Among them, compounds **3** and **4** possess identical planar structures but different rel. configs., as is the case for **8** and **9**.

Due to the structural flexibility of 8,4′-oxyneolignans, it has often been a challenge to correctly assign their configurations at C-7 and C-8. In order to solve this problem, three empirical rules have been developed to determine the rel. configs.: the comparison of *J*_7,8_ coupling constants [22,23], and the utilization of ^13^C (Δ*δ*_(C-8−C-7)_) [24] and ^1^H (Δ*δ*_(H-9a−H-9b)_) [25] NMR chemical shift differences, although each method has its limitations.

The application of the *J*_7,8_ value, first reported by Ruveda et al. in 1984 [23], is the simplest and most commonly used method (data see Table 1), but different substituents and their substitution positions could significantly impact the magnitude of *J*_7,8_, and sometimes even result in close *J*_7,8_ values for *erythro* and *threo* configurations. Additionally, the use of different deuterated solvents for NMR measurements will also influence the *J*_7,8_ value. Therefore, the configuration assignments based on this empirical rule are sometimes ambiguous or even improper due to misuse. Shi and coworkers have summarized three types of 8,4′-oxyneolignans that are suitable for the application of this rule, i.e., aglycones (*J*_7,8_ ≤ 5.0 Hz for *erythro* and *J*_7,8_ ≥ 7.0 Hz for *threo*), aglycone acetonides (*J*_7,8_ > 7.0 Hz for *erythro* and *J*_7,8_ < 2.0 Hz for *threo*) and glycoside acetates (*J*_7,8_ ≤5.3 Hz for *erythro* and *J*_7,8_ ≥ 6.3 Hz for *threo*); the NMR solvent must be CDCl_3_ [22]. As reflected by the previously reported data shown in Table 1, some researchers tend to apply this method without being aware of the aforementioned limitations, which could have resulted in incorrect configuration assignments and caused confusion in later studies of other NPs.

The Δ*δ*_(C-8−C-7)_ value was introduced to differentiate between *erythro* and *threo* 8,4′-oxyneolignans by Gan et al. [24,26], whereas only a few lignans are applied as reference compounds, and their Δ*δ*_(C-8−C-7)_ values also vary in different deuterated solvents, thus causing this method to lack universality. In 2019, the third method of use of Δ*δ*_(H-9a−H-9b)_ value was developed by Zhang and coworkers [25]. However, as with the rule of Δ*δ*_(C-8−C-7)_ value, lack of enough model compounds has limited its application. In summary, the rel. config. determination for 8,4′-oxyneolignans could be very complicated due to their structural flexibility and diversity, and special cautions are always suggested to avoid erroneous assignments.

Up to now, three methods, i.e., direct ECD analysis by utilizing the Cotton effect at 235 ± 5 nm, TDDFT-ECD method and modified Mosher’s method, have been used to establish the abs. configs. of 8,4′-oxyneolignans. For the first method, it is claimed that the positive Cotton effect at around 235 ± 5 nm is related to 8*S-*configuration, while a negative one corresponds to 8*R*-configuration [22]. However, different substituents on the aryl group, C-7, C-8 and C-9 would cause evident impact on the Cotton effects and the corresponding wavelengths. Caution thus should be taken when applying this rule, as improper applications have often been encountered in the literature. The TDDFT-ECD method is to theoretically predict the ECD spectra of the two possible enantiomers and then compare the calculated curves with the experimental ones. It is by far the most commonly used approach to assign abs. configs. of natural enantiomers owing to its easy operability, without the need for chemical derivatization and for constructing theoretical mechanisms to explain the observed properties [27]. Although this method is nowadays readily accessible to nonexperts, experience is still required since unexpected wrong assignments are easily made. As shown in Table 1, the abs. configs. determined by the first two methods are often inconsistent, and those assigned via the TDDFT-ECD method are usually accepted as the final determination in these reports. The third one is modified Mosher’s method that requires chemical derivatization, and its accuracy and feasibility have been proved and accepted by almost all chemists. Nonetheless, a secondary alcohol and enough amount of sample for derivatization are a must for this method, and only pure enantiomers are suitable for investigation. In addition, it is worthwhile to note that the specific optical rotation data have no straight-forward correlation with the abs. configs. of studied structures (Table 1).

**Figure 2:**
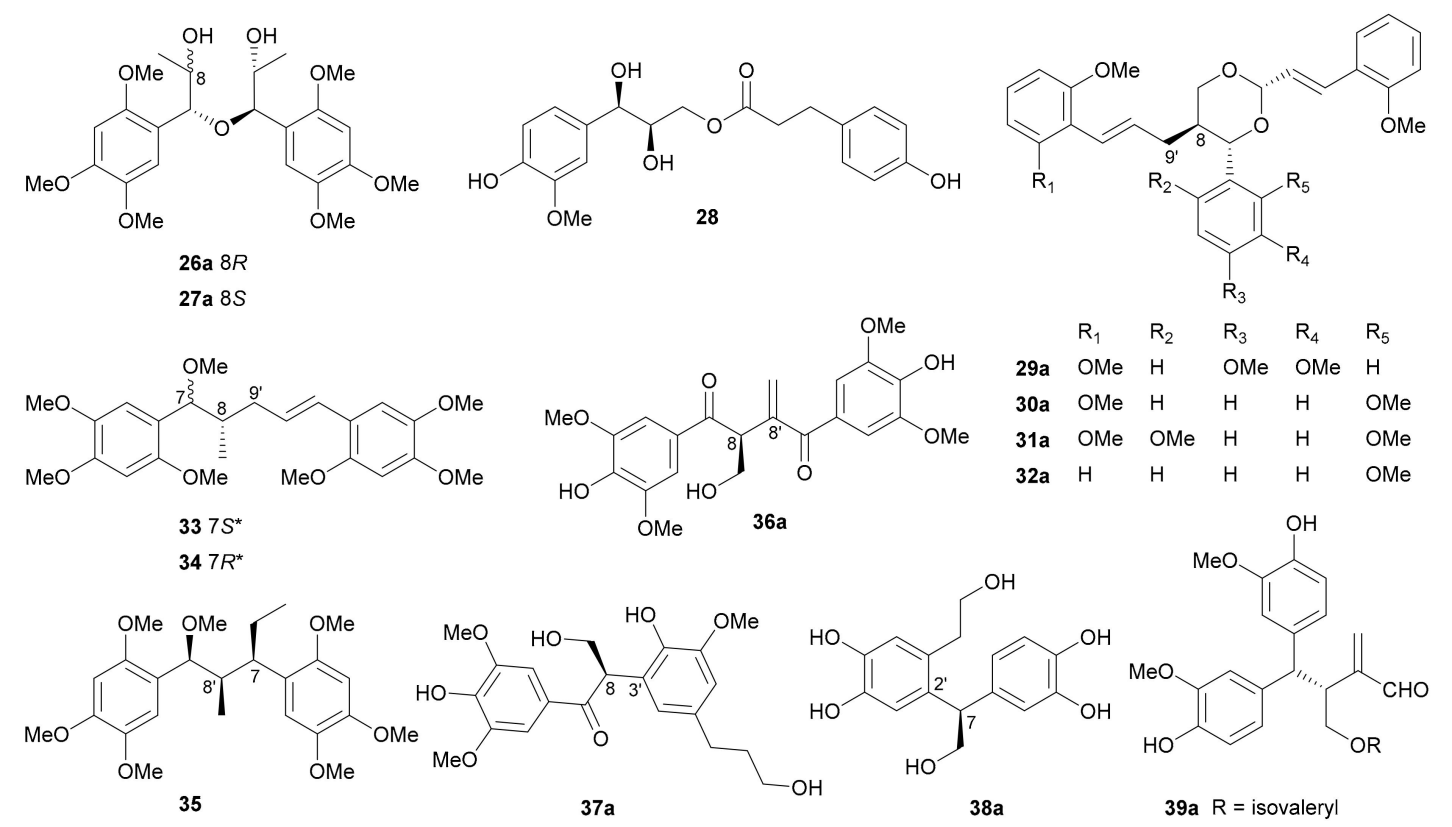
Structures of other acyclic lignans.

*Other acyclic lignans.* In addition to 8,4′-oxyneolignans, many other acyclic lignan enantiomers with various connection patterns were reported in this period, as shown in Figure 2 (names see Table S2 in Supplementary Materials). Owing to the limited numbers, they have all been put together and are discussed in the current section.

**Scheme 1:**
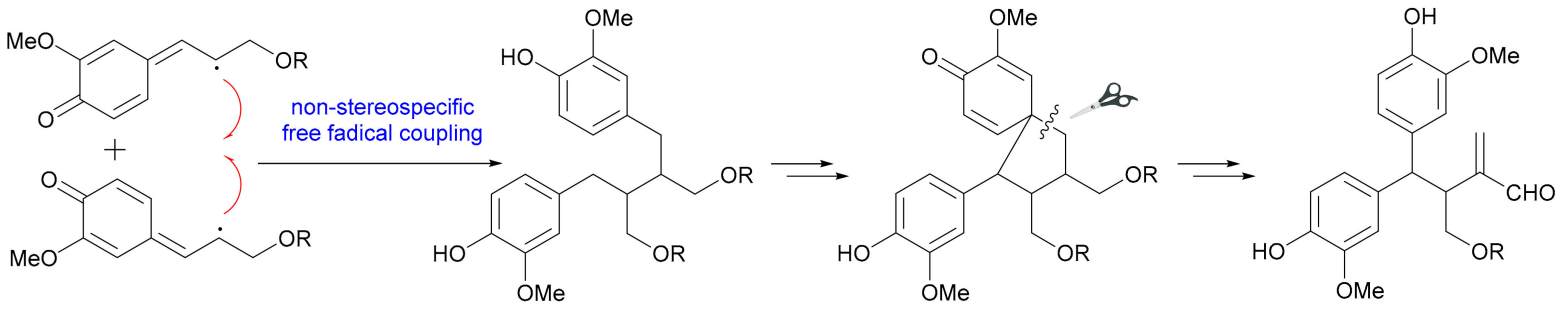
Plausible biosynthetic pathway for **39**.

Compounds **26** and **27** from *Acorus tatarinowii* are rare cases of naturally occurring 7,7′-oxyneolignans [28]. The *threo*-configurations for C-7/C-8 and C-7′/C-8′ of **26** were first determined by the large *J*_7,8_ and *J*_7′,8′_ values (both 6.5 Hz), which was further confirmed by single crystal X-ray diffraction analysis, while the abs. configs. for **26a**/**26b** and **27a**/**27b** were established by comparing the calculated and experimental ECD curves. The 9,9′-oxyneolignan **28** with an ester linkage was obtained as a racemic mixture from *Bulbophyllum retusiusculum* [29]. Compounds **29**−**32** from the trunk of *Torreya yunnanensis* are rare examples of 8,9′-neolignans and all feature a 1,3-dioxane motif by acetalization with a 2-methoxy-cinnamaldehyde [30]. Yao and coworkers reported two 8,9′-neolignans (**33** and **34**) and one 7,8′-neolignan (**35**) as racemic mixtures from *Acorus tatarinowii* [28]. The *J*_7,8_ values (7.6 Hz for **33** and 6.1 Hz for **34** in CD_3_OD) were used by the authors to determine the *threo* and *erythro* rel. configs for **33** and **34**, while the rel. configs for **35** was assigned by single crystal X-ray diffraction analysis. An 8,8′-lignan (**36**), an 8,3′-neolignan (**37**) and a 7,2′-neolignan (**38**) were isolated from *Liriodendron hybrid* [31], *Selaginella moellendorffii* [32] and *Syringa pinnatifolia* [33], respectively. Sasaki et al. acquired a pair of novel 8,8′-lignan enantiomers (**39a**/**39b**) with rearranged skeleton (also known as secolignan) from *Brachanthemum gobicum*, with the abs. configs. being determined by comparing the ECD and specific optical rotation data with those of (−)-lucidenal [34]. The plausible biosynthetic pathway of **39** was also proposed as shown in Scheme 1, with the nonstereospecific free radical coupling being the key factor to generate enantiomers.

#### 2.1.2. Cyclic Lignans

**Figure 3:**
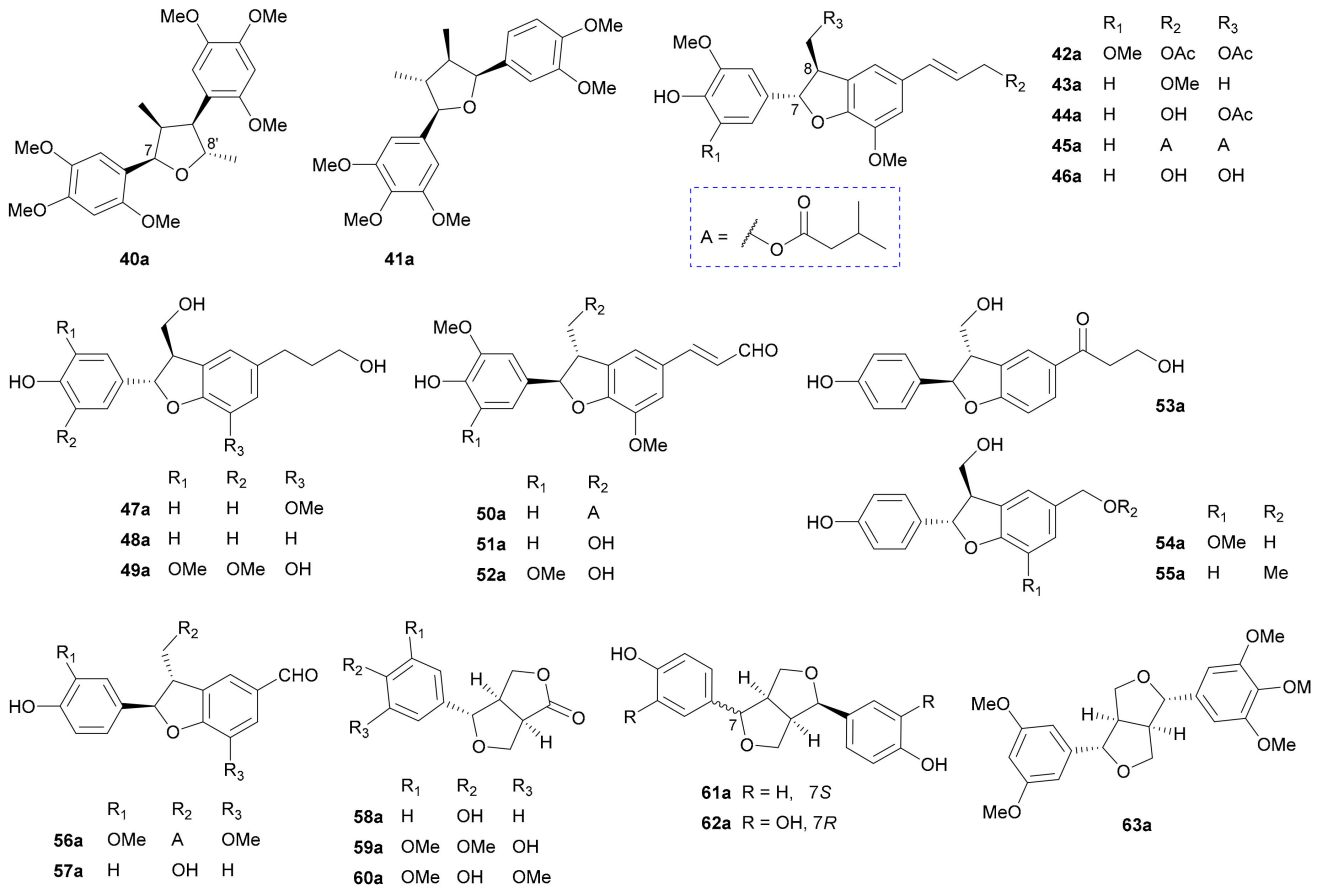
Structures of Furan-incorporating lignans.

*Furan-incorporating lignans.* Furan-incorporating lignans are a class of common NPs with one or more or modified furan rings in the structures. These lignan enantiomers reported in this period mainly comprise three subtypes, namely, normal furan-type (**40** and **41**), benzofuran-type (**42**–**57**) and furofuran-type (**58**–**63**) (for structures, see Figure 3; for names, see Table S3 in Supplementary Materials).

Compound **40**, a normal furan-type lignan enantiomer pair with 8-7′ and 7-*O*-8′ connections, was reported as a racemic mixture from *magnolia salicifolia* in 1984 [35], and was synthesized in 1992 [36]; however, its chiral separation and abs. config. determination were only realized by Lu et al. in 2018 [16]. The chiral separation and abs. config. assignment of **41**, whose structure with rel. config. was reported in 1996 [37], were accomplished by Zhang and coworkers in their phytochemical investigation of *Acorus tatarinowii* in 2016 [38].

Benzofuran-type lignan enantiomer pairs **42**−**44**, **45** and **46** were discovered from *Jatropha integerrima* in 2015 [39], *Brachanthemum gobicum* in 2018 [34] and *Picrasma quassioides* in 2018 [40], respectively. The 7,8-*trans* configurations for **42**−**46** were determined by the large *J*_7,8_ values (7.5 Hz for **42**−**45** in CDCl_3_, and 6.7 Hz for **46** in DMSO-*d*_6_) and NOE analyses, while the abs. config. assignments for these compounds were based on the reversed helicity rule [39,41]. According to this empirical rule, *P*-helicity of the nonaromatic ring will lead to a positive Cotton effect within the ^1^L_b_ band (around 280 nm) and *M*-helicity will result in a negative Cotton effect. Phytochemical investigation into the plants *Rubus idaeus* [42,43] and *Phyllanthus glaucus* [44] led to the isolation of three enantiomer pairs **47**−**49**, and their abs. configs. were established by TDDFT-ECD method. Compounds **50−52** incorporating an *α*,*β*-unsaturated aldehyde unit were obtained from *Brachanthemum gobicum* [34] and *Picrasma quassioides* [40], with the abs. configs. also being assigned by the reversed helicity rule, where their ^1^L_b_ (α) bands red shifted to around 340 nm due to the conjugation effect from the *α*,*β*-unsaturated aldehyde group. In 2018, Huang et al. discovered compound **53** as a racemic mixture from *Rubus ideaus* and further resolved it into two enantiomers (**53a**/**53b**), with the abs. configs. being assigned by application of the TDDFT-ECD method [43]. In fact, compound **53** had been previously reported as an optically pure molecule from *Broussonetia papyrifera* in 2009, with a much smaller [*α*]_D_ value [45], suggesting its potential scalemic nature. Compounds **54**−**57** are a group of dinorneolignans and were isolated from *Rubus idaeus* [42,43] and *Brachanthemum gobicum* [34].

In 2019, Song and colleagues obtained the trinorneolignan furolactone **58** as a racemic mixture from *Rubus idaeus* and resolved it into a pair of enantiomers (**58a**/**58b**), the abs. configs. of which were established by analyses of the calculated shielding tensor values and ECD data [46], while the enantiomer **58b** had been reported as an optically pure molecule from *Lycium chinense* in 2013 [47]. The other two pairs of furolactone enantiomers **59a**/**59b** and **60a**/**60b** were isolated from *Archidendron clypearia* in 2018 [48] and *Dendrobium nobile* in 2016 [49], respectively. Song and coworkers reported **61a**/**61b**, from *Rubus idaeus* in 2019 and assigned their abs. configs. by using the TDDFT-ECD method [47]. Compounds **62** and **63** represent another two pairs of furofuran-type lignan enantiomers isolated from *Morinda citrifolia* [50] and *Acorus tatarinowii* [16], respectively.

**Figure 4:**
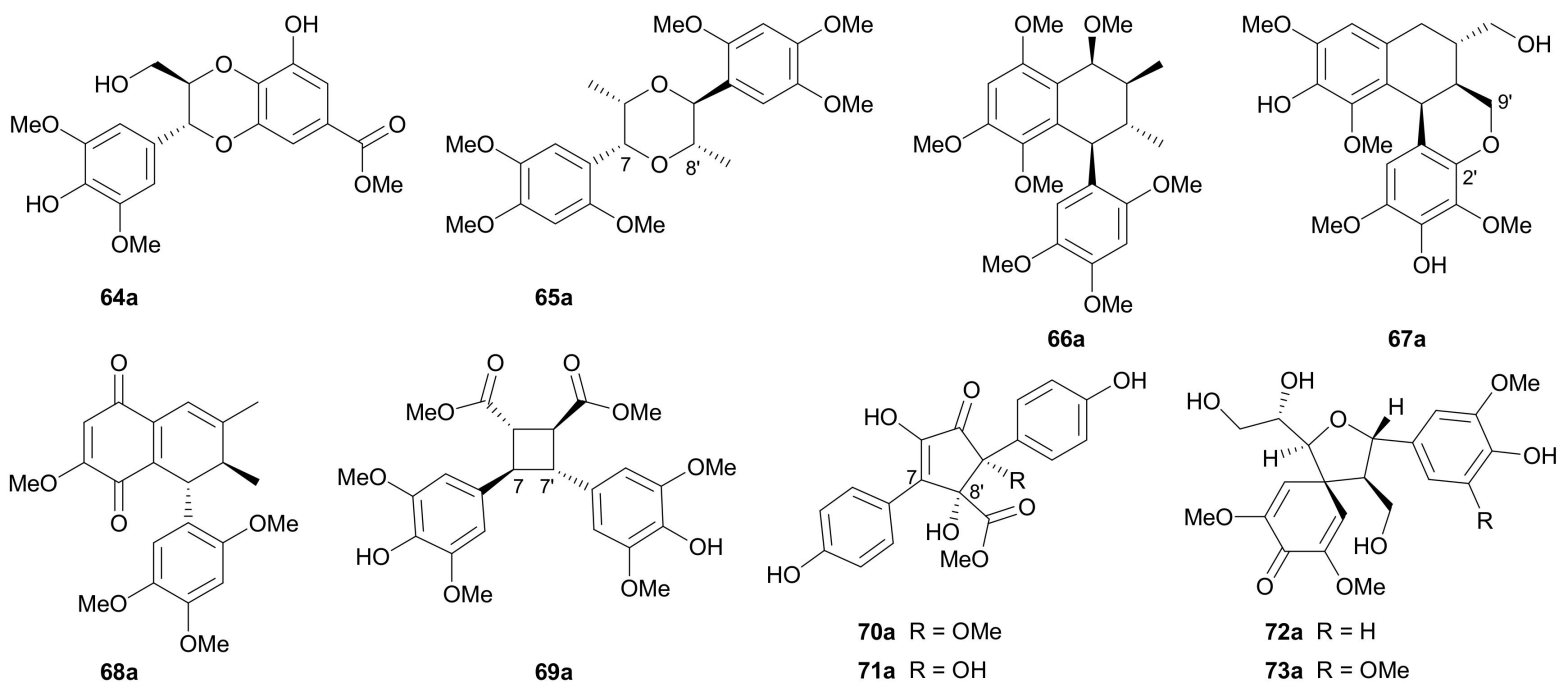
Structures of other cyclic lignans.

*Other cyclic lignans.* Except for the aforementioned Furan-incorporating lignan enantiomers, there are also some other cyclic lignan enantiomers with diverse ring systems as listed in Figure 4 (names see Table S4 in Supplementary Materials).

The unusual dinorneolignans **64a**/**64b** incorporating a 1,4-dioxane motif and the arylnaphthalene-type lignans **67a**/**67b** bearing a 2′,9′-epoxy ring were separated from *Pithecellobium clypearia* in 2018, with their abs. configs. being determined by the TDDFT-ECD method [51]. In 2015, Zhang et al. reported a pair of 7,8′-epoxy-8,7′-oxyneolignans (**65a**/**65b**) and a pair of oxidized arylnaphthalene-type lignans (**68a**/**68b**) from *Acorus tatarinowii*, and the abs. configs. of the two pairs were established by employing the TDDFT-ECD method and comparing the Cotton effect at 315 nm with those of known analogues, respectively [38]. The other pair of enantiomeric arylnaphthalenes **66a**/**66b** were also isolated from *Acorus tatarinowii*, with their abs. configs. being assigned by comparing the Cotton effect at 285 nm with those of known analogues [28]. Compounds **69a**/**69b** featuring a cyclobutane ring via 7,7′ and 8,8′ connections represent a pair of [2 + 2] cycloaddition adducts of two phenylpropanoid units and were obtained from *Isatis indigotica* in 2019 [52]. Isolated from *Tylopilus eximius* in 2012 are two pairs of enantiomers **70a**/**70b** and **71a**/**71b** both incorporating a cyclopentenone ring formed by 7,8′ and 9,7′ linkages [53]. The rel. config. of racemic **71** was established by X-ray crystallography, while the abs. configs. of **70a**/**70b** and **71a**/**71b** were confirmed by TDDFT-ECD method. In 2015, two pairs of rare spirodienone neolignans (**72a**/**72b** and **73a**/**73b**) were reported from *Cinnamomum subavenium*, with the absolute structures being elucidated by X-ray crystallographic analysis and TDDFT-ECD method [54].

#### 2.1.3. Sesquineolignans

**Figure 5:**
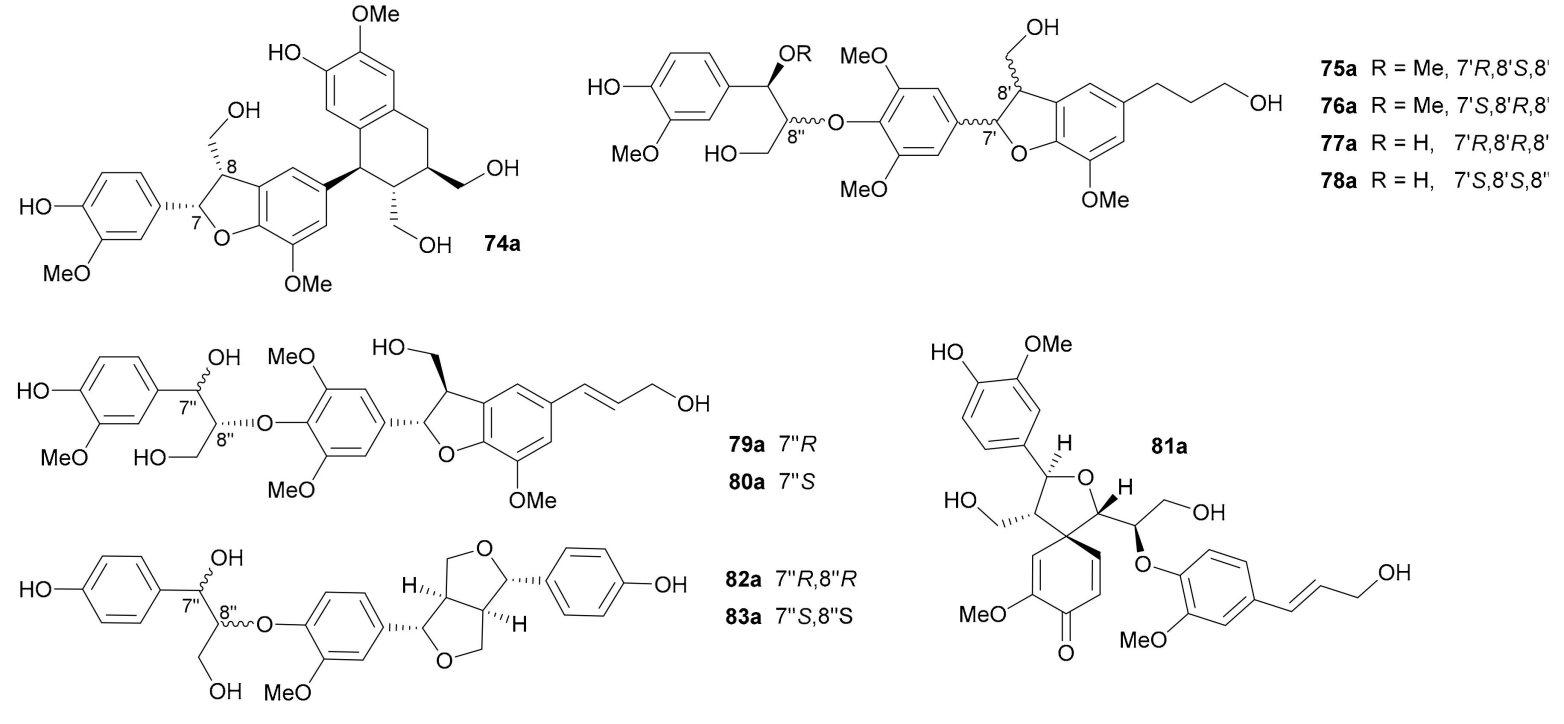
Structures of sesquineolignans.

Sesquineolignans refer to lignans bearing three phenylpropanoid units with various connection patterns. Ten pairs of enantiomeric sesquineolignans were reported in this period, and their structures are shown in Figure 5, with the names being listed in Table S5 in Supplementary Materials.

**Scheme 2:**
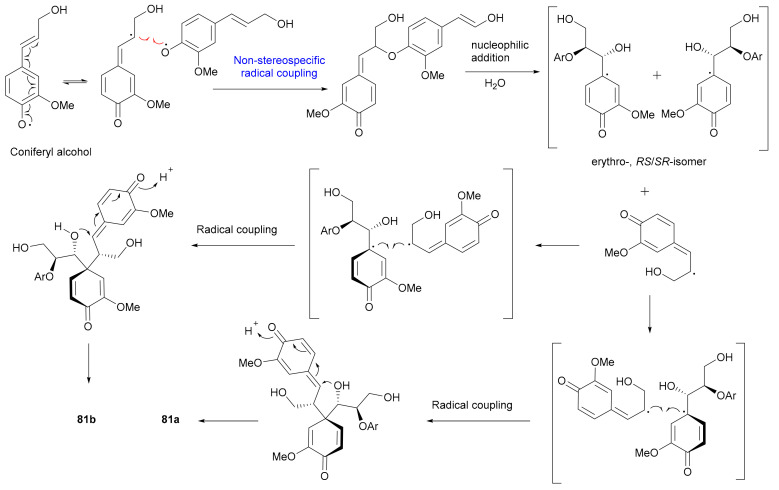
Plausible biosynthetic pathways for **81a**/**81b**.

In 2015, Zhang and colleagues obtained **74**−**78** from *Phyllanthus glaucus*, with the 7,8-*cis* configuration of **74** being determined via the small *J*_7,8_ value (2.1 Hz) and the abs. configs. of **74a**/**74b** being assigned by comparing ECD data with those of known analogues [44]. In addition, the 7″,8″-*threo* configurations of **75** and **76** and 7″,8″-*erythro* configurations of **77** and **78** were determined by the *J*_7″,8″_ values (6.1 Hz in CDCl_3_ for **75** and 6.3 Hz in Me_2_CO-*d*_6_ for **76**; 5.1 Hz in CD_3_OD for **77** and 4.2 Hz in Me_2_CO-*d*_6_ for **78**). In 2015, Yin and coworkers reported **79** and **80** from *Brachanthemum gobicum* and applied the reversed helicity rule to assign the abs. configs. at C-7′ and C-8′ [34], while the assignments of abs. configs. at C-7″ and C-8″ were established by Rh_2_(OCOCF_3_)_4_-induced ECD analysis. On the basis of the bulkiness rule for secondary alcohols, a positive Cotton effect at around 350 nm (E band) in the Rh_2_(OCOCF_3_)_4_-induced ECD spectrum indicated a *S*-configuration, while a negative Cotton effect implied a *R*-configuration. In 2014, Yu’s group discovered a pair of novel enantiomeric tetrahydrofuran spirodienone sesquineolignans (**81a**/**81b**) from *Xanthium sibiricum* and proposed coniferyl alcohol as the biosynthetic precursor (Scheme 2), and the nonstereospecific radical coupling between the two C_6_-C_3_ units was the key factor to result in enantiomers [55]. Song and coworkers reported **82** and **83** from *Rubus idaeus* in 2019 and assigned their abs. configs. by using the TDDFT-ECD method [47].

In summary, a large number of lignans (except lignan glycosides) have been discovered as racemic or scalemic mixtures and chirally separated in recent years, and their structural types, from simple to complex (via rearrangement), cover more than half of the known classes. It is self-evident from the aforementioned examples that enantiomerism widely occurs in the structural categories of lignans especially for 8,4′-oxyneolignans and furan-incorporating lignans. These lignan enantiomers exist as either racemic or scalemic mixtures in plants and can be relatively easily separated by commercially available chiral chromatographic columns. Therefore, it is conceivable that many examples previously reported as optically pure lignans could in fact be scalemic mixtures, and NP researchers should pay extra attention to the enantiomeric purity of lignans in their future work.

### 2.2. Coumarins

**Figure 6:**
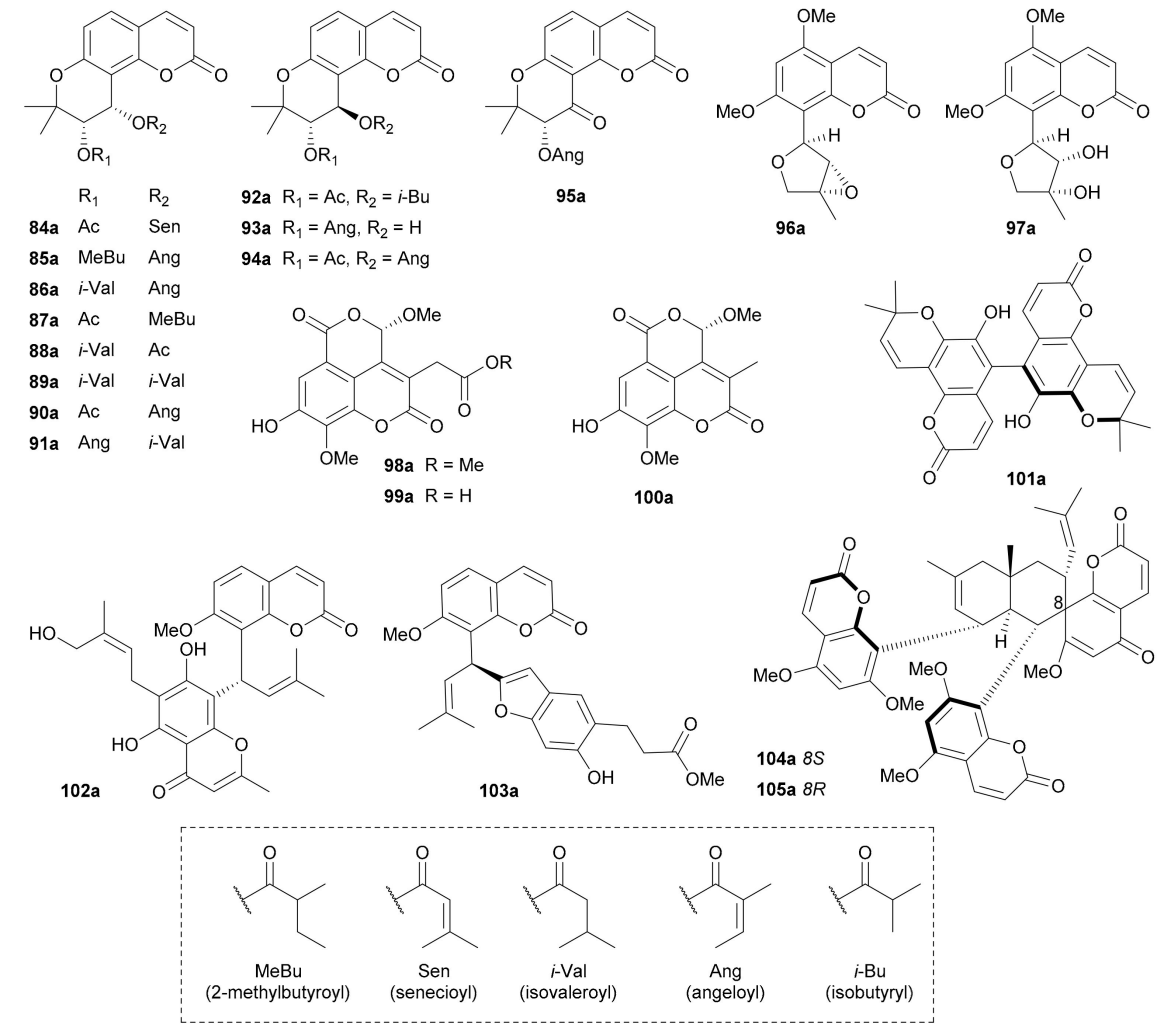
Structures of coumarins.

‘Coumarin’ is the general name of *ortho*-hydroxycinnamate lactones that are derived from the Shikimate biosynthetic pathway^1^ (for structures, see Figure 6; for names, see Table S6 in Supplementary Materials). As the core backbone of coumarins does not contain chiral factors, the generation of their enantiomersim usually comes from the chiral carbons of substituents (e.g., prenyl substitution) or axial chirality of oligomers. Coumarins are important secondary metabolites in plants and have shown various biological properties such as antitumor, anti-HIV, antimicrobial and anti-inflammatory activities [56,57].

Compounds **84a**−**87a** are a group of angular dihydropyranocoumarins and were obtained as 3′*S*,4′*S*-configured pure enantiomers from *Peucedanum japonicum* in 2017 [58], while their corresponding 3′*R*,4′*R*-enantiomers (**84b**−**87b**) had been previously reported from *Angelica morii* in 1974 [59], *Peucedanum praeruptorum* in 2012 [60], *Seseli gummiferum* in 1971 [61] and *Angelica furcijuga* in 2000 [62], respectively. Another eight pairs of analogues **88**−**95** were found to be present as scalemic mixtures in *Peucedani Radix* [63] and were successfully separated into pure enantiomers for the first time. Except **88a**, **89b**, **92a** and **92b**, the others have been formerly reported as optically pure compounds [63], but the small [α]_D_ values compared with those for the purified enantiomers suggested their scalemic natures. Tang and coworkers isolated two pairs of coumarin enantiomers (**96a**/**96b** and **97a**/**97b**) from *Toddalia asiatica* and assigned the rel. and abs. configs. of **96a**/**96b** via X-ray diffraction experiment and TDDFT-ECD method, respectively [64]. From *Sapium baccatum*, three coumarin enantiomer pairs incorporating one additional *α*-pyrone ring (**98**−**100**) were assigned the abs. configs. by comparing their specific optical rotations with those of known analogues [65]. Compounds **102** and **103** are two pairs of hybrid dimer enantiomers from *Cnidium monnieri* and they were also total synthesized for further biological test [66]. The most complex coumarin enantiomers so far are the oligomeric coumarin hybrids **104** and **105** bearing a spirodienone-sesquiterpene skeleton, and they were isolated from *Toddalia asiatica* in 2016 [67]. The only coumarin enantiomers generated by axial chirality are the prenylated coumarin dimers **101a**/**101b**, with the abs. configs. being determined by TDDFT-ECD method [68].

### 2.3. Simple Phenylpropanoids

**Figure 7:**
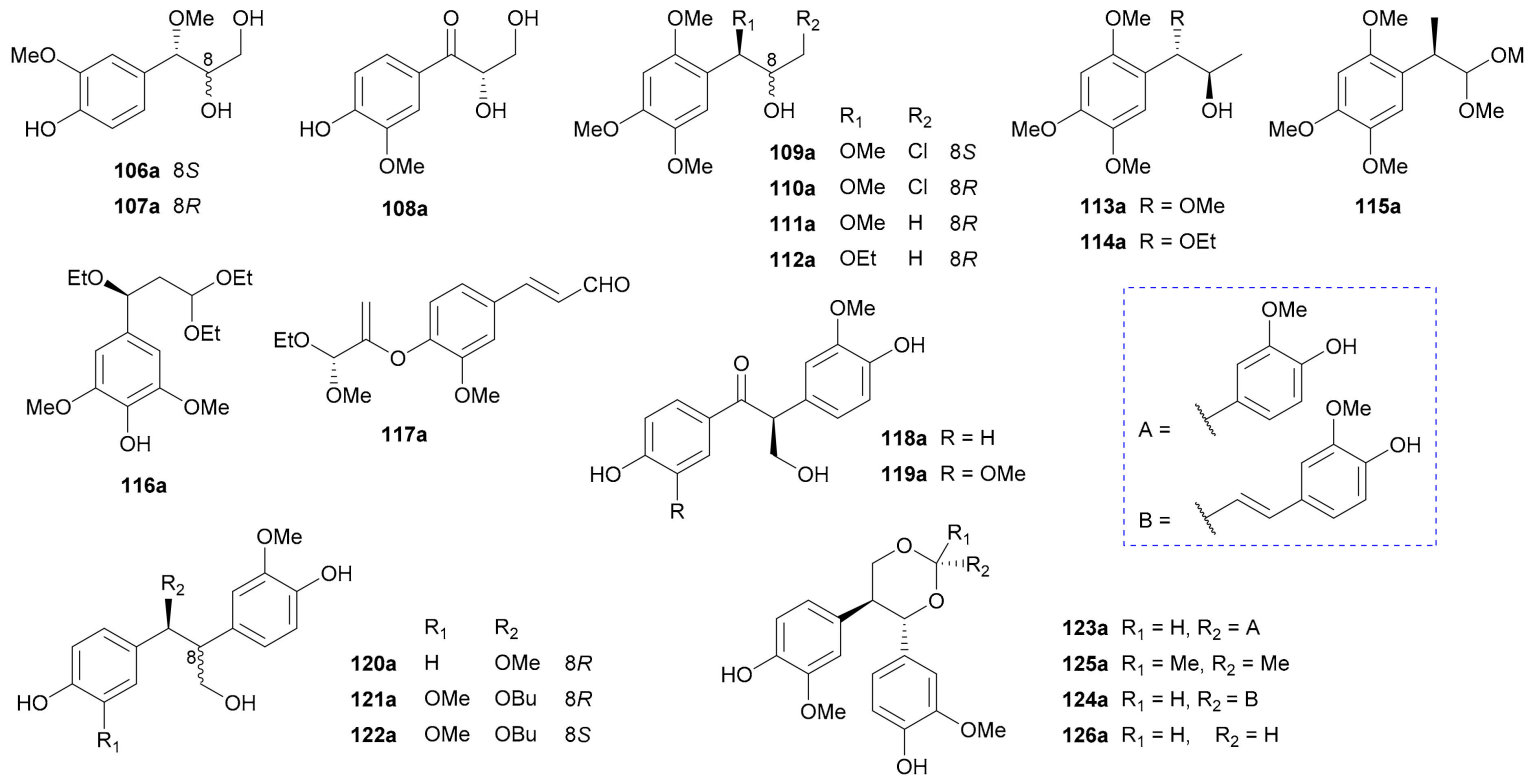
Structures of simple phenylpropanoids.

Simple phenylpropanoids are naturally occurring phenolic substances containing only one C_6_-C_3_ biosynthetic block. They often exist as racemic or scalemic mixtures in nature (for structures, see Figure 7; for names, see Table S7 in Supplementary Materials). They are also derived from the Shikimate biosynthetic pathway [1]. Many phenylpropanoids play vital roles in plant growth regulation and pathogen defense by acting as essential components of cell wall, as protectants against high light and UV radiation, and as phytoalexins against herbivores and pathogens [69]. It is generally difficult to acquire high quality crystals for X-ray diffraction analysis due to the rotary nature of the sidechains in most phenylpropanoids, so normally their rel. configs. are assigned by *J* values and the abs. configs. are determined on the basis of Snatzke’s rule, modified Mosher’s method or TDDFT-ECD calculation.

Qiu and coworkers reported two pairs of phenylpropanoid enantiomers, **106a**/**106b** and **107a**/**107b**, from the leaves of *Eucommia ulmoides* and assigned their rel. and abs. configs. by analysis of *J*_7,8_ values and Snatzke’s method, respectively [70]. The planar structure of **108** had already been reported in 2001 [71], but its enantiomeric nature was not revealed by Liu et al. until 2017, with the abs. configs. being determined by Snatzke’s rule [72]. Two pairs of rare chlorine-containing enantiomers (**109a**/**109b** and **110a**/**110b**) were isolated from *Acorus tatarinowii* in 2017, and their rel. and abs. configs. were established by analyzing *J*_7,8_ values and employing modified Mosher’s method, respectively [73]. The enantiomer pairs **111a**/**111b**−**115a**/**115b** were obtained from *Acorus tatarinowi* in 2017 by Gao’s group, among which **111b** and **113b** had been reported previously [74]. Compounds **115a**/**115b** are the first cases in nature of asarone-derived phenylpropanoids with an isopropyl fragment tethered to the benzene core, and their abs. configs. were assigned by TDDFT-ECD method [74]. Song and colleagues isolated **116a**/**116b** and **117a**/**117b** from the fruit of *Crataegus pinnatifida* in 2018 and applied the TDDFT-ECD method to establish their abs. configs [74]. Compounds **118**−**126** with an extra phenyl group on the sidechain are also considered to be 1,2-diphenylpropane derivatives. The enantiomer pairs **118**−**120** were separated from *Rubus idaeus* in 2018 with their abs. configs. being determined via the TDDFT-ECD method [75]. Compounds **121** and **122** were first reported as racemic mixtures from *Casearia grewiifolia* in 2012 [76] and were resolved into two pairs of enantiomers by Qiu et al. in 2018, with the rel. and abs. configs. being determined by analyzing *J*_7,8_ values and applying TDDFT-ECD method, respectively [77]. Compounds **123a**/**123b**−**126a**/**126b** featuring a 1,3-dioxane ring derived from condensation of diol with different aldehydes, were obtained from *Crataegus pinnatifida* with their abs. configs. being determined by TDDFT-ECD method [78].

### 2.4. Alkaloids

The term “alkaloids” traditionally describes nitrogen-containing small molecule organic compounds with basicity, although there is no unified definition. In this section, we include all nitrogen-bearing NPs in this category. Based on the structural types, natural alkaloid enantiomers from plants in the period of 2012–2019 are classified into indole alkaloids, quinoline and isoquinoline alkaloids, *β*-carboline and carbazole alkaloids, piperidine alkaloids, thiohydantoin alkaloids, indolizidine and quinolizidine alkaloids, and other alkaloids.

#### 2.4.1. Indole Alkaloids

**Figure 8:**
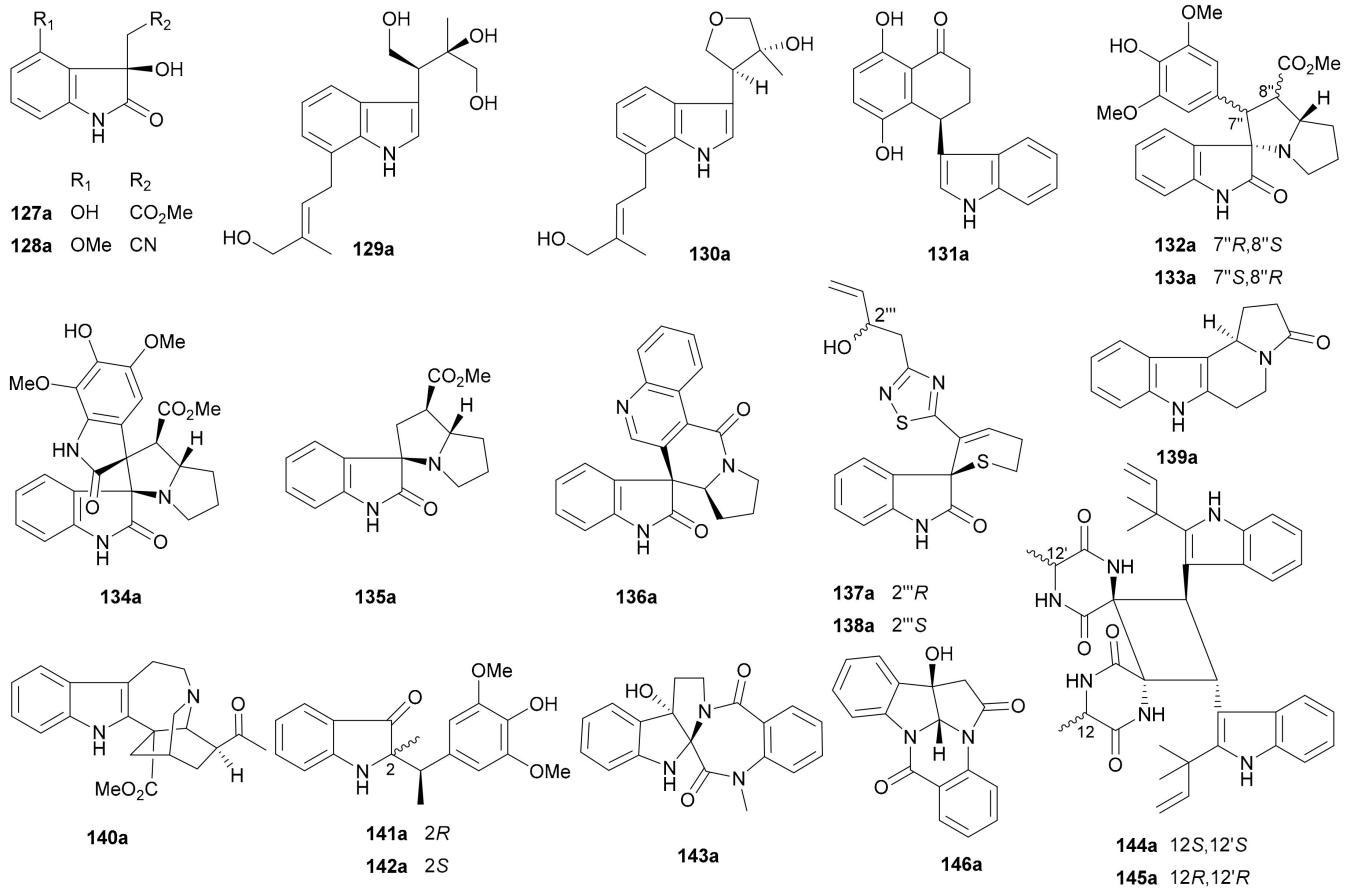
Structures of indole alkaloids.

Indoles are biogenetically derived from tryptophan or tryptamine and make up one of the largest groups of alkaloid metabolites. They have attracted tremendous attention because of their therapeutic values such as anti-inflammatory, antinociceptive, antitumor, antioxidant and antimicrobial effects [79,80]. The structures of indole alkaloid enantiomers reported in this period are depicted in Figure 8 and the names summarized in Table S8 in Supplementary Materials. 

Song and coworkers reported two pairs of oxindole enantiomers **127a**/**127b** and **128a**/**128b** from *Isatis tinctoria* in 2019 [81], while **128b** had been previously reported in optically pure form from *Isatis indigotica* in 2012 [82]. In 2017, Zhang and colleagues discovered two pairs of enantiomers bearing two prenyl groups (**129a**/**129b** and **130a**/**130b**) from *Clausena lansium* and assigned the rel. config. of **129b** by converting it into an acetonide derivative [83]. Two pairs of novel enantiomers including the indole 3,4-dihydronaphthalen-1(*2H*)-one hybrids (**131a**/**131b**) and the indolizino [7,8-*b*]indol alkaloids (**139a**/**139b**) were found to exist as scalemic mixtures in *Juglans regia* [84]. In 2018, Liu et al. reported **132**−**135** featuring a spiropyrrolizidine oxindole skeleton from *Isatis indigotica* [85]. Reported from *Isatis indigotica* in 2019, the enantiomers **136a**/**136b** incorporate an interesting spiro-oxindole skeleton [86]. Concurrently isolated with **136a**/**136b** is another pairs of enantiomers **146a**/**146b** featuring a pyrrolo[2,3-*b*]indolo[5,5a,6-*b*,*a*]quinazoline skeleton that had also been reported from the same species in 2012 [82]. The abs. configs. of **146a**/**146b** were determined by using the bulkiness rule for the Rh_2_(OCOCF_3_)_4_-induced ECD data, wherein the E band (around 350 nm) was demonstrated to be useful for determining the abs. configs. of chiral secondary and tertiary alcohols [82]. Characterized by the presence of a dihydrothiopyran ring and a 1,2,4-thiadiazole ring in the structure, the oxindole alkaloid enantiomers **137a**/**137b** and **138a**/**138b** were reported from *Isatis indigotica* by Shi’s research group in 2018 and 2012, respectively [87,88]. The iboga-type indole alkaloid **140a** was obtained as an optically pure molecule from *Tabernaemontana corymbosa* in 2016 [89] with the rel. config. being determined by X-ray diffraction analysis, while its enantiomer **140b** was reported from *Ervatamia hainanensis* in 2015 [90]. Two pairs of rare indole-styrene hybrid derivatives **141a**/**141b** and **142a**/**142b** were isolated from *Isatis indigotica* [91]. The rearranged rutaecarpine-type indole alkaloid enantiomers **143a**/**143b** from *Evodia rutaecarpa* incorporate an unprecedented 6/5/5/7/6 skeleton [92]. The dimeric isoechinulin-type indole alkaloid enantiomers **144a**/**144b** and **145a**/**145b** from *Uncaria rhynchophylla* feature an intriguing and complex skeleton with a symmetrical cyclobutane ring, and their rel. configs. were assigned by X-ray crystallography [93]. Except for **146a**/**146b**, the abs. configs. of other indole enantiomers were assigned by using the TDDFT-ECD method, with the abs. config. of **138a** being further confirmed by modified Mosher’s method.

It is interesting to note that suitable crystals for X-ray diffraction analysis of the enantiomeric mixtures seem relatively easy to be obtained in these reports, but the acquisition of high quality crystals of pure single enantiomers appears difficult. As above described, from simple indoles (e.g., **127**) to monoterpenoid indole hybrids (e.g., **140**), from single indoles (e.g., **129**) to dimeric indoles (e.g., **134**), from one-chiral-center examples (e.g., **131**) to complex multiple-chiral-center indole dimers (e.g., **144**), natural indole alkaloid enantiomers spread in a wide range of structural subtypes. Therefore, checking enantiomeric purity for this important class of NPs appears to be key in the future work.

#### 2.4.2. Quinoline and Isoquinoline Alkaloids

**Figure 9:**
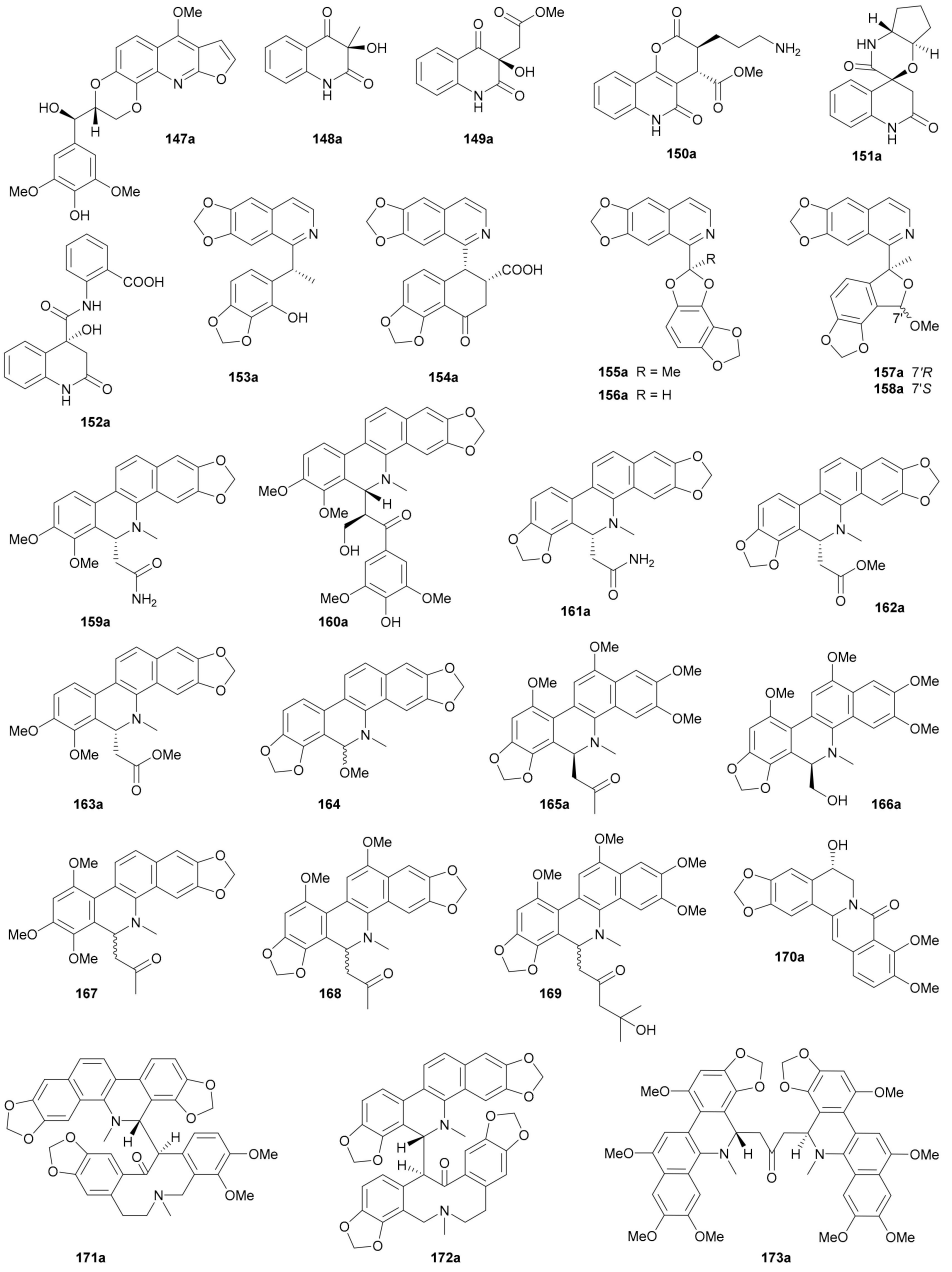
Structures of quinoline and isoquinoline alkaloids.

Most quinoline and isoquinoline alkaloids biosynthetically originate from anthranilic acid or from indoles via rearrangement [94]. Quinoline & isoquinoline alkaloids have attracted great interest from researchers worldwide because of their wide-range biological activities, including antitumor, antiparasitic and insecticidal, antibacterial and antifungal, cardioprotective, antiviral, antiinflammatory, hepatoprotective, antioxidant, anti-asthma, antitussive, and other activities [95,96]. The structures and names of these alkaloid enantiomers in the covered stage are summarized in Figure 9 and Table S9 in Supplementary Materials, respectively.

Compounds **147a**/**147b**, featuring a furoquinoline core hybridized with a phenylpropanoid unit via a 1,4-dioxane ring, were separated and characterized from *Zanthoxylum nitidum* in 2018 [97]. Three 2-quinolinone enantiomer pairs **148**−**150** were reported from *Isatis tinctoria* by Song and coworkers in 2019 [81], and in the same year, Zhang et al. discovered the same type of alkaloid enantiomers **151a**/**151b** from the roots of *Isatis indigotica* [86]. The last example of quinolinone enantiomer pair is compound **152** also from *I. indigotica*, and it incorporates an additional anthranilic acid residue [98].

**Scheme 3:**
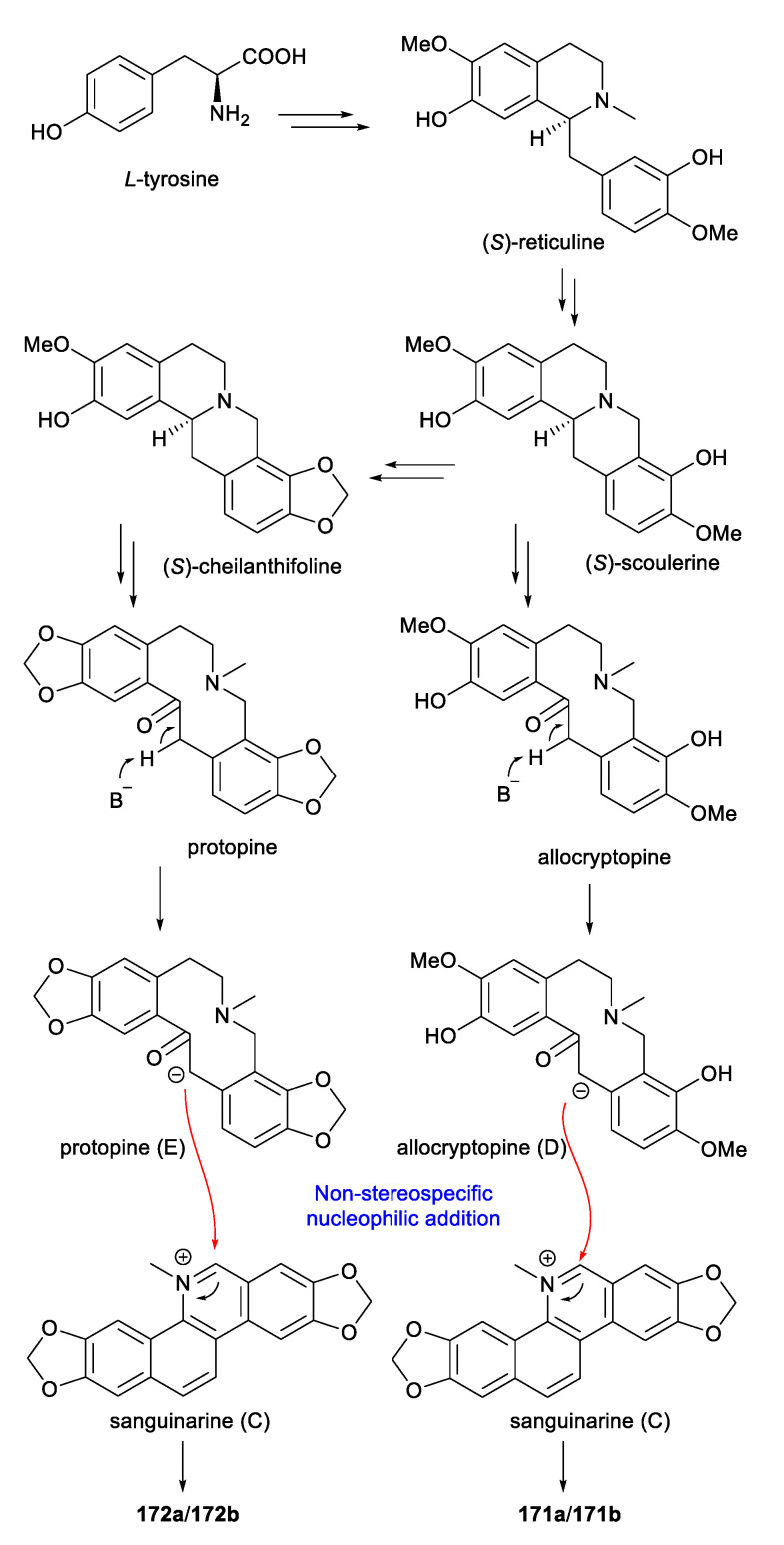
Plausible biosynthetic pathways for **171** and **172**.

Compounds **153**−**158** are a series of isoquinoline enantiomers, among which **154a**/**154b** were acquired from *Corydalis hendersonii* in 2016 [99] and the others were obtained from *Corydalis mucronifera* in 2018 [100]. Compounds **154a**/**154b** were proposed to be derived from the condensation of a benzylisoquinoline and a succinic acid [99]. In 2016, Hua and colleagues reported from *Macleaya cordata* five dihydrobenzophenanthridine enantiomer pairs **159**−**163** and a racemate **164**, among which **162** and **163** had been previously isolated in racemic form from *Macleaya cordata* [101] and here is the first record of their chiral separation [102]. Three same type of enantiomer pairs **165**, **166** and **173** were isolated and characterized from *Corydalis ambigua* var. *amurensis* by Han and coworkers, and three racemic mixtures **167**−**169** were also acquired and analyzed by chiral chromatography but without further separation due to their limited amount [103]. As for structure elucidation, single-crystal X-ray diffraction analysis was applied to determine the abs. config. of **165a**, followed by the abs. config. assignments for **165b** and **166a**/**166b** via comparing the ECD curves with that of **165a**. In addition, Ye’s group reported a pair of berberine-type alkaloid enantiomers **170a**/**170b** from *Coptis chinensis* in 2014 [104]. Sai et al. discovered from *Corydalis ambigua* two pairs of alkaloid dimers **171a**/**171b** and **172a**/**172b**, featuring a novel dimerization pattern from two different types of monomers via a C–C single bond [105]. The plausible biosynthetic pathways for **171** and **172** were also proposed as shown in Scheme 3 by the authors, and the nonstereospecific nucleophilic addition was assumed to be the key factor to generate enantiomers [105]. As with the aforementioned indole alkaloids, the assignments of abs. configs. for most quinoline and isoquinoline enantiomers have been based on the TDDFT-ECD method.

#### 2.4.3. *β*-Carboline and Carbazole Alkaloids

**Figure 10:**
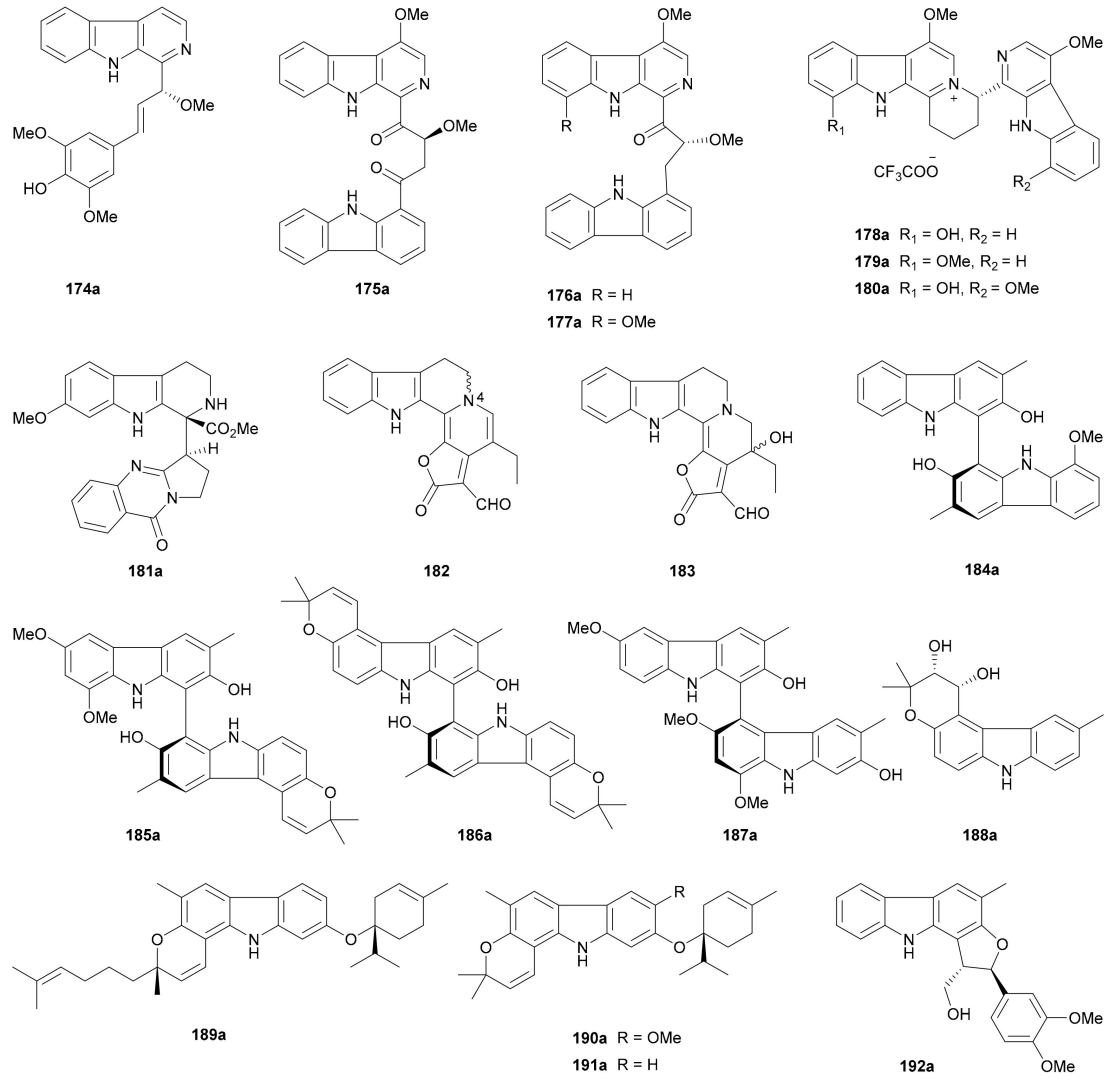
Structures of *β*-carboline and carbazole alkaloids.

*β*-Carbolines and carbazoles are among the most intriguing alkaloid groups; they derive from various sources. They have gained increasing attention due to their broad spectrum of biological activities [106,107]. Seven *β*-carboline (**174**, **178**−**183**), three *β*-carboline-carbazole hybrid (**175**−**177**) and nine carbazole (**184**−**192**) enantiomer pairs have been reported in this period (for structures, see Figure 10; for names, see Table S10 in Supplementary Materials). The abs. configs. for all separated enantiomers in this section were determined by the TDDFT-ECD method unless otherwise specified.

Song and coworkers phytochemically studied the stems of *Picrasma quassioides* to detect four enantiomer pairs **174a**/**174b**−**177a**/**177b**. While **174a**/**174b** possess a *β*-carboline-phenylpropanoid hybrid skeleton [108], the latter three pairs represent alkaloid heterodimers of a *β*-carboline and a carbazole units which are linked via a C_4_ fragment. Alkaloids **178a**/**178b**−**180a**/**180b** are dimeric *β*-carbolines obtained as trifluoroacetates from *Picrasma quassioides* in different years [109,110]. Compounds **181a**/**181b**, as *β*-carboline-quinazoline hybrid dimers from *Peganum harmala*, were biogenetically produced through Mannich/Pictet–Spengler-type and intermolecular Michael addition reactions [111]. Compounds **182** and **183** from *Pausinystalia yohimbe* were characterized in racemic forms in 2018 without further chiral separation, and their racemic nature was further proved by X-ray diffraction analysis [112]. Interestingly, the enantiomerism of **182** results from the *N*-4 chiral center which is very rare in nature [112].

The enantiomerism of carbazole alkaloids comes from the axial chirality of dimers or from the chiral centers in the additional structural fragments. Four pairs of biscarbazole atropisomers (**184a**/**184b**−**187a**/**187b**) and a pair of dihydropyranocarbazole enantiomers (**188a**/**188b**) were discovered by Jiang and colleagues from *Clausena dunniana*, where the planar structure of **185** had been previously described from *Clausena wallichii* in 2011 [113]. The same authors from Jiang’s group further reported **189a**/**189b**−**192a**/**192b** from *Murraya microphylla* [114,115], with the rel. config. of **189** being confirmed by X-ray crystallographic data [115].

#### 2.4.4. Piperidine Alkaloids

**Figure 11:**
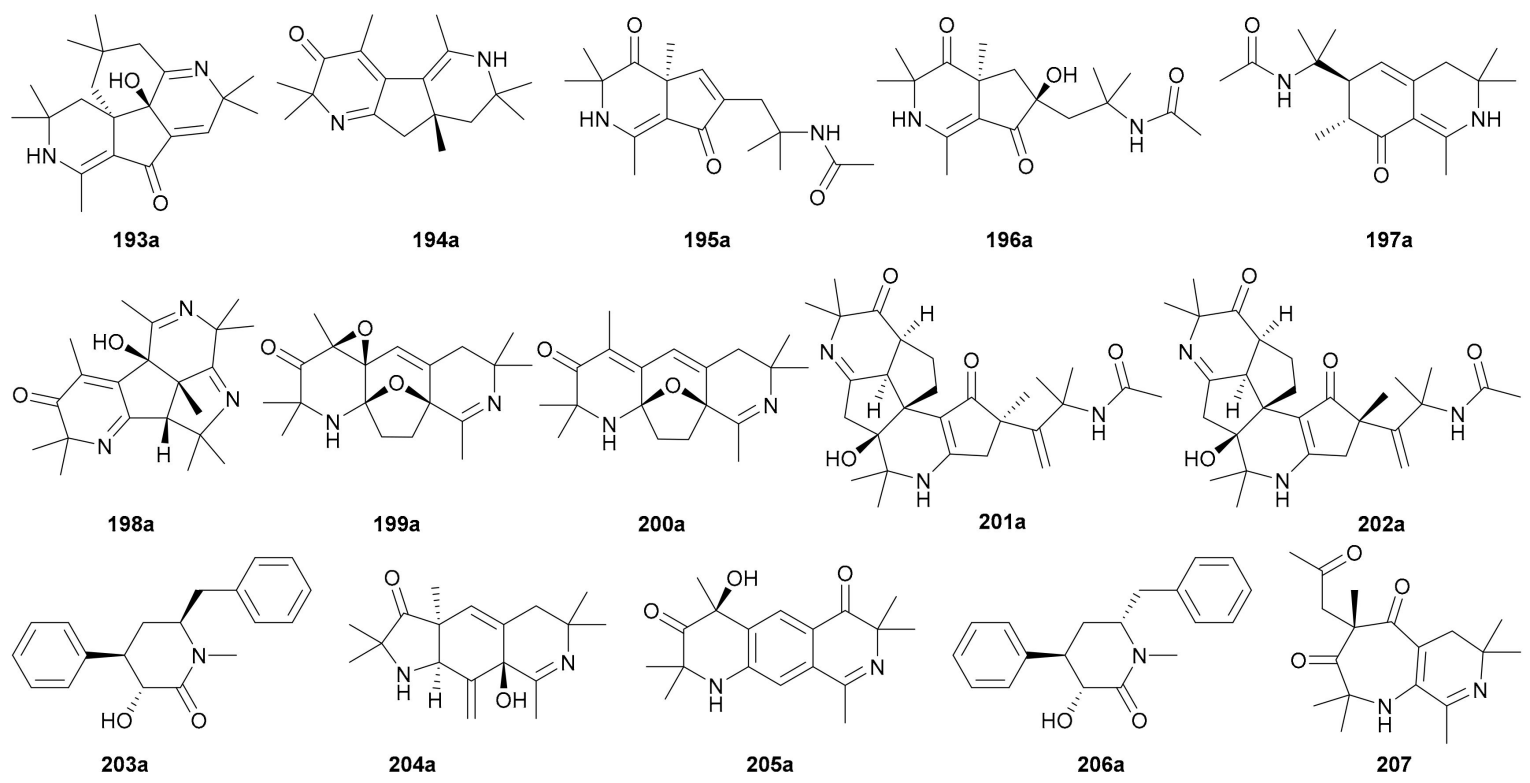
Structures of piperidine alkaloids.

Piperidine alkaloids that have one or more piperidine rings in the structures are generally believed to be biogenetically derived from lysine [1]. During the period covered by this review, fifteen pairs of piperidine enantiomers (for structures, see Figure 11; for names, see Table S11 in Supplementary Materials) have been reported.

The enantiomer pairs **193**−**197** were isolated from *Anacyclus pyrethrum* in 2018 [116]. Among them, compounds **193** and **194** possess novel dimeric piperidine backbones with 6/5/6/6 and 6/5/6 ring systems, respectively, while **195a**/**195b** incorporate a rare cyclopentane-piperidine framework. In 2017, compounds **198**−**205** were obtained from *Viola tianschanica*. All of them bear more than one nitrogen atom and incorporate fascinating heterocyclic architectures such as the 6/5/6/5 and 6/5/5/6/5 ring systems in **198** and **201**–**202**, respectively [117]. The abs. configs. of these alkaloid enantiomers were established by the TDDFT-ECD method. Compounds **206** and **207** were found to occur as enantiomeric pairs in *Clausena lansium* with only **206** being successfully resolved into pure enantiomers. The rel. and abs. configs. of **206a**/**206b** were established by X-ray crystal data and comparing ECD and specific optical rotation data with calculated ones [118].

The biogenetic origins of these piperidines, especially of those with highly complex skeletons and multiple chiral centers like **198** and **201**–**202**, have not been examined, and this intriguing puzzle definitely deserves further investigations.

#### 2.4.5. Thiohydantoin Alkaloids

**Figure 12:**
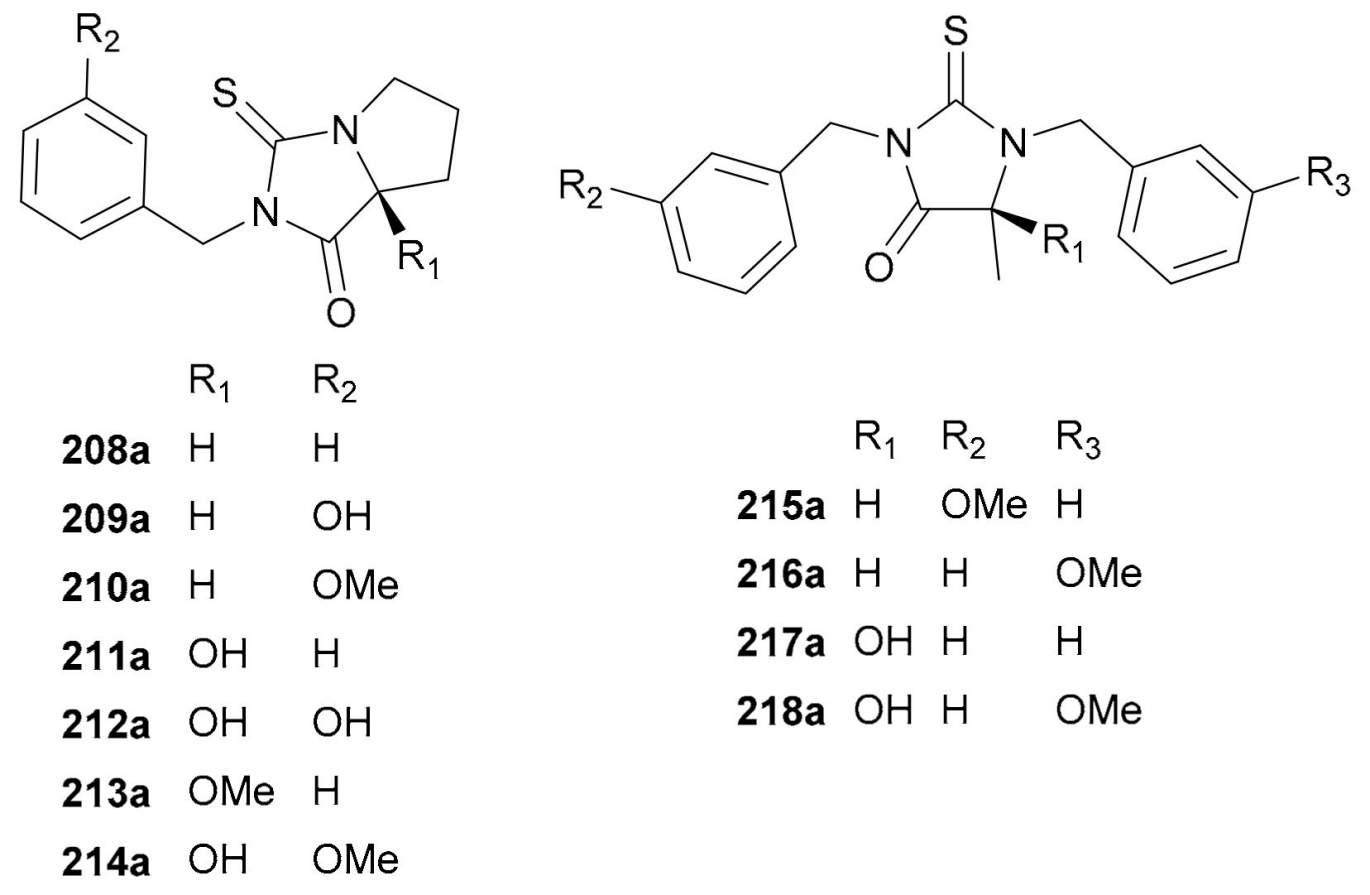
Structures of thiohydantoin alkaloids.

Naturally occurring thiohydantoin alkaloids are a rare class of NPs. Compounds **208a**/**208b−218a/218b** (for structures, see Figure 12, names see Table S12 in Supplementary Materials), a panel of thiohydantoin derivatives of two structural groups, were initially obtained as racemic mixtures from *Lepidium meyenii* and further resolved into eleven pairs of enantiomers [119]. Among them, an unidentified enantiomer of **208** had been reported as a synthetic product in 2007 [120]. Although the biogenesis of these alkaloids has never been studied, they very likely belong to the imidazole class originating from histidine on the basis of their core structures [1]. 

#### 2.4.6. Indolizidine and Quinolizidine Alkaloids

**Figure 13:**
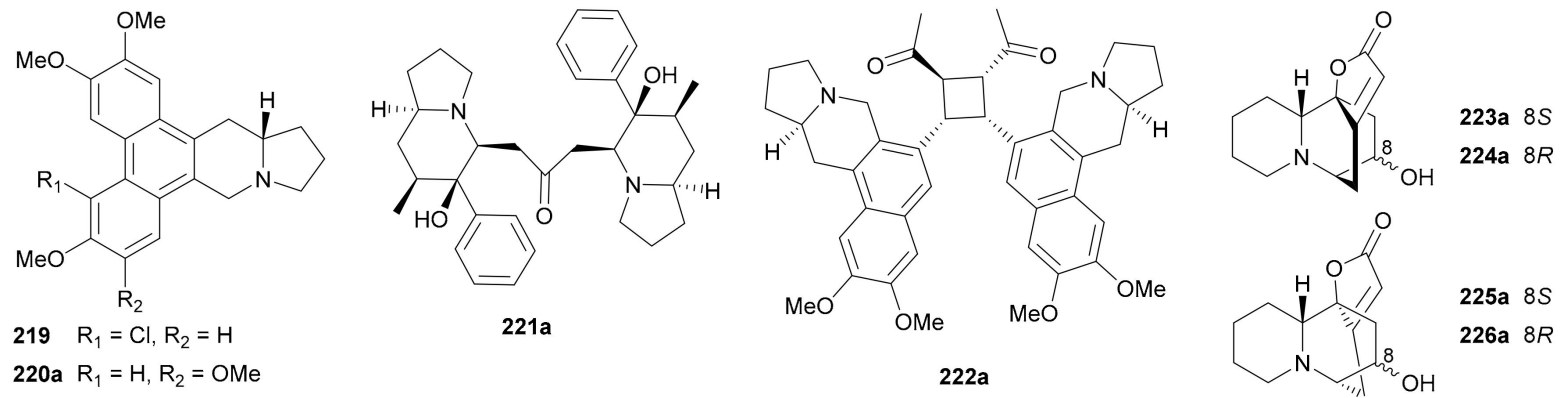
Structures of indolizidine and quinolizidine alkaloids.

Both indolizidine and quinolizidine alkaloids are biogenetically originated from lysine [1], and an equal number of four pairs of indolizidine (**219**−**222**) and quinolizidine (**223**−**226**) enantiomers (for structures, see Figure 13; for names, see Table S13 in Supplementary Materials) have been reported in the period covered by this review.

Alkaloid **219** from *Ficus fistulosa* var. *tengerensis* was identified as a scalemic mixture by [α]_D_, ECD and X-ray crystallographic data [121], while **220** as a long-known NP re-isolated from *Tylophora indica* [122] was demonstrated to be a nearly racemic mixture with only a slight excess of the *R*-enantiomer [123]. Compounds **221a**/**221b**, a pair of enantiomeric indolizidine alkaloid dimers from *Dendrobium crepidatum*, were assigned the abs. configs. by single-crystal X-ray diffraction analysis [124]. Enantiomers **222a**/**222b**, whose structures were also confirmed by X-ray diffraction analysis to be indolizidine dimers linked via a cyclobutane ring, were obtained from the same species as **219** [121]. Zhang et al. discovered four pairs of neosecurinane-type alkaloid enantiomers **223a**/**223b**−**226a**/**226b** of the quinolizidine class from *Flueggea virosa* in 2017, and it is the first time to report the enantiomerism of this interesting type of alkaloids [5]. The rel. and abs. configs. of **223a**/**223b**−**226a**/**226b** were characterized by a variety of techniques including X-ray crystallography and ECD experiments.

#### 2.4.7. Other Alkaloids

**Figure 14:**
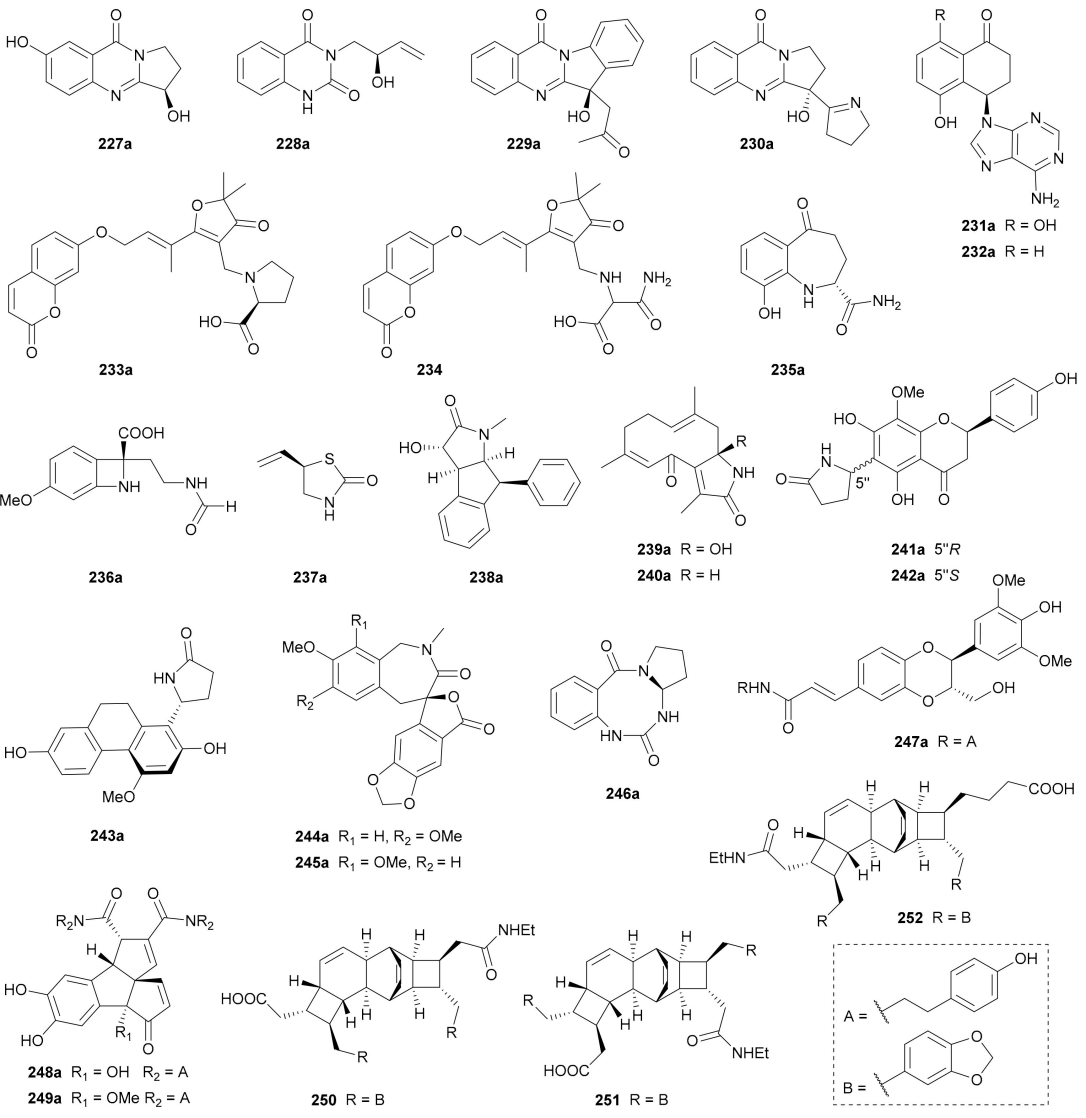
Structures of other alkaloids.

In addition to the aforementioned alkaloid enantiomers occurring naturally in plants, there are also many other types of alkaloid enantiomers reported in this period, as summarized in Figure 14 (names see Table S14 in Supplementary Materials).

Compounds **227a**/**227b−230a**/**230b** are a panel of quinazoline enantiomer pairs obtained from *Peganum harmala* in 2018 [125], *Isatis indigotica* in 2019 [86], *I. indigotica* in 2016 [98] and *P. harmala* in 2016 [126], respectively. Biogenetically, quinazoline alkaloids have been demonstrated to be derived from anthranilic acid [1].

Qin and coworkers discovered two pairs of adenine alkaloids **231a**/**231b** and **232a**/**232b** from *Juglans regia* in 2016 [84]. Compounds **233a**/**233b**, along with its scalemic analogue **224**, were reported from *Geijera parviflora*, and they feature a novel heterotrimer structure incorporating a norsesquiterpenoid unit between a coumarin moiety and a proline residue [127]. Compounds **235a**/**235b** from *Juglans regia* possess a benzo[*b*]azepine-2-carboxamide skeleton [84], while **236a**/**236b** from *Peganum harmala* are amphoteric alkaloids with a four-membered *N*-heterocyclic ring [126].

Compounds **237**−**252** are amide alkaloid enantiomers with miscellaneous backbones. The simplest cases are **237a**/**237b** bearing a thiazolidin-2-one ring and they were isolated from *Isatis indigotica* [87]. Zhang and coworkers discovered **238a**/**238b** from *Clausena lansium* and assigned their abs. configs. by using modified Mother’s method [83], and two pairs of germacrane-type sesquiterpenoid lactams **239** and **240** were obtained from *Curcuma phaeocaulis* by Qiu and colleagues [128]. Compounds **241a**/**241b** and **242a**/**242b** are flavonoid alkaloid enantiomers reported from *Scutellaria moniliorrhiza* in 2018 [129], while **243a**/**243b** represent a pair of 9,10-dihydrophenanthrene alkaloid enantiomers from *Bletilla striata* [130]. The enantiomer pairs **244a**/**244b** and **245a**/**245b** featuring a spiro[benzofuranone-benzazepine] skeleton from *Juglans mandshurica* [131], as well as **246a**/**246b** incorporating a benzo[*f*][1,3,5]triazocine backbone from *Isatis tinctoria* [81], were all reported by the research team of Song and Huang. Compounds **247a**/**247b** are a pair of enantiomers formed by an oxyneolignan and a phenethylamine units from *Lycium chinense* [132], while **248a**/**248b** and **249a**/**249b** are rearranged nor-lignan amide enantiomers featuring a unique benzo-angular triquinane skeleton from *Cannabis sativa* [133]. Alkaloids **250**−**252** were obtained as racemic mixtures from *Endiandra kingiana* without further chiral separation, and their racemic nature was claimed on the basis of their zero [α]_D_ values [134].

Except for the specified ones, the abs. configs. of the alkaloid enantiomers in this section were all established by applying the TDDFT-ECD method.

### 2.5. Flavonoids

Flavonoids are a large family of secondary metabolites that exist widely in the plant kingdom. They exhibit a variety of bioactivities such as anti-inflammatory, antioxidant, antibacterial, antiviral, anticancer and neuroprotective effects [135]. Traditionally, flavonoids mainly refer to compounds incorporating a 2-phenylchromone core, and nowadays, this term has extended to all structures with two phenyl units linked via a C_3_ fragment [14]. In addition, some NPs such as xanthones and furanochromones are also included in this structural family as atypical flavonoids. Flavonoid enantiomers reported in this period are classified into three subgroups: flavones and isoflavones, chalcones and xanthones

#### 2.5.1. Flavones and Isoflavones

**Figure 15:**
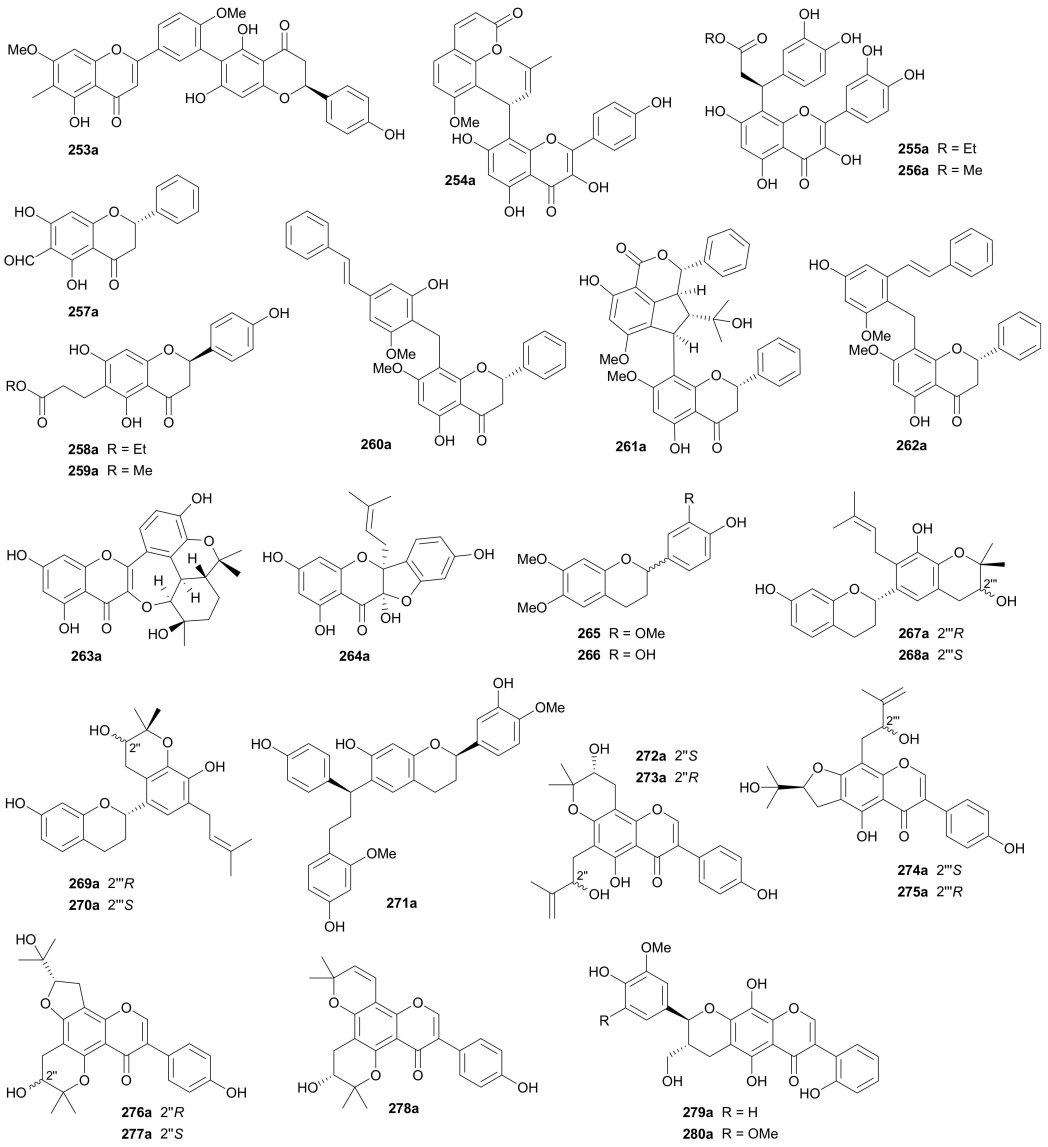
Structures of flavones and isoflavones.

In this section, the definition ‘flavones’ refers to all those incorporating the basic 2-phenylchromone backbone, including classical flavones, flavanones, flavanes, etc. The abs. configs. of these enantiomers are mostly determined by the TDDFT-ECD method unless otherwise specified. Their structures and names are shown in Figure 15 and Table S15 in Supplementary Materials, respectively.

The biflavonoid enantiomers **253a**/**253b** were isolated from *Selaginella trichoclad* by Tan and coworkers in 2019 [136], with the abs. configs. being assigned by an empirical rule developed by Gaffield [137]. This rule was described as that 2*S*-configured flavanones and 2*R*,3*R*-configured 3-hydroxyflavanones have a positive Cotton effect at ~330 nm caused by the n→π* transition and a negative Cotton effect due to the π→π* transition at around 280−290 nm. In 2017, a pair of enantiomers hybridized from a flavonol and a coumarin via a prenyl unit (**254a**/**254b**) were isolated from *Cnidium monnieri*, and their constitutional structure was further confirmed by semi-synthesis through condensation of the monomeric precursors [66]. Compounds **255a**/**255b** and **256a**/**256b** are flavanol-phenylpropanoid adducts and were discovered from *Uncaria rhynchophylla* in 2017 [138,139]. Muhammad and colleagues reported a pair of 6-formylated flavanone enantiomers **257a**/**257b** from *Eugenia rigida* and also semi-synthesized them for further biological studies [140]. Two pairs of flavanones coupled with a propionate residue (**258a**/**258b** and **259a**/**259b**) were separated from the aerial parts of *Abrus precatorius* in 2019 by Li et al. [141]. Wang and coworkers reported three pairs of flavanone-stilbene hybrid enantiomers **260a**/**260b**−**262a**/**262b** from *Cajanus cajan*, and **261a**/**261b** feature a cyclopenta[1,2,3-*de*]isobenzopyran-1-one tricyclic unit with cajanolactone A being proposed as the biosynthetic precursor [142]. Two prenylated flavones **263** and **264** were isolated from *Morus nigra* and successfully resolved into two pairs of enantiomers in 2019 [143], while **264** had been previously reported as a racemic mixture in 2018 [144]. Compounds **263a**/**263b** incorporate an interesting framework with a novel 7/6/6 heterocyclic ring system. Flavanes **265** and **266** were characterized as two racemic mixtures by X-ray diffraction analysis but without further separation into pure enantiomers [145]. Compounds **267a**/**267b**−**270a**/**270b** are four pairs of diprenylated flavane enantiomers from *Daphne giraldii*, with the abs. configs. being determined by Rh_2_(OCOCF_3_)_4_ induced ECD method [146]. Compounds **271a**/**271b**, as heterodimers derived from a flavane and a diphenylpropanoid, were isolated from *Dracaena cochinchinensis* in 2016 [147].

Compounds **272a**/**272b**−**278a**/**278b**, seven pairs of enantiomeric diprenylated isoflavones with diverse ring systems, were reported from *Maclura tricuspidata* by Lee’s research group in 2018 [148]. The enantiomer pairs **279a**/**279b** and **280a**/**280b** were obtained from the stems of *Pisonia umbellifera* and characterized as hybrids from an isoflavone and a phenylpropanoid [149].

As can been from the above-mentioned structures, the enantiomerism of these flavones mainly comes from either the chirality of flavanone/flavane core or that of additional structural units especially prenyl group(s), or both. Meanwhile, the enantiomerism of the described isoflavones arises exclusively from the chirality of extra structural units, i.e., prenyl group(s) and phenylpropanoid fragment for the current cases.

#### 2.5.2. Chalcones

**Figure 16:**
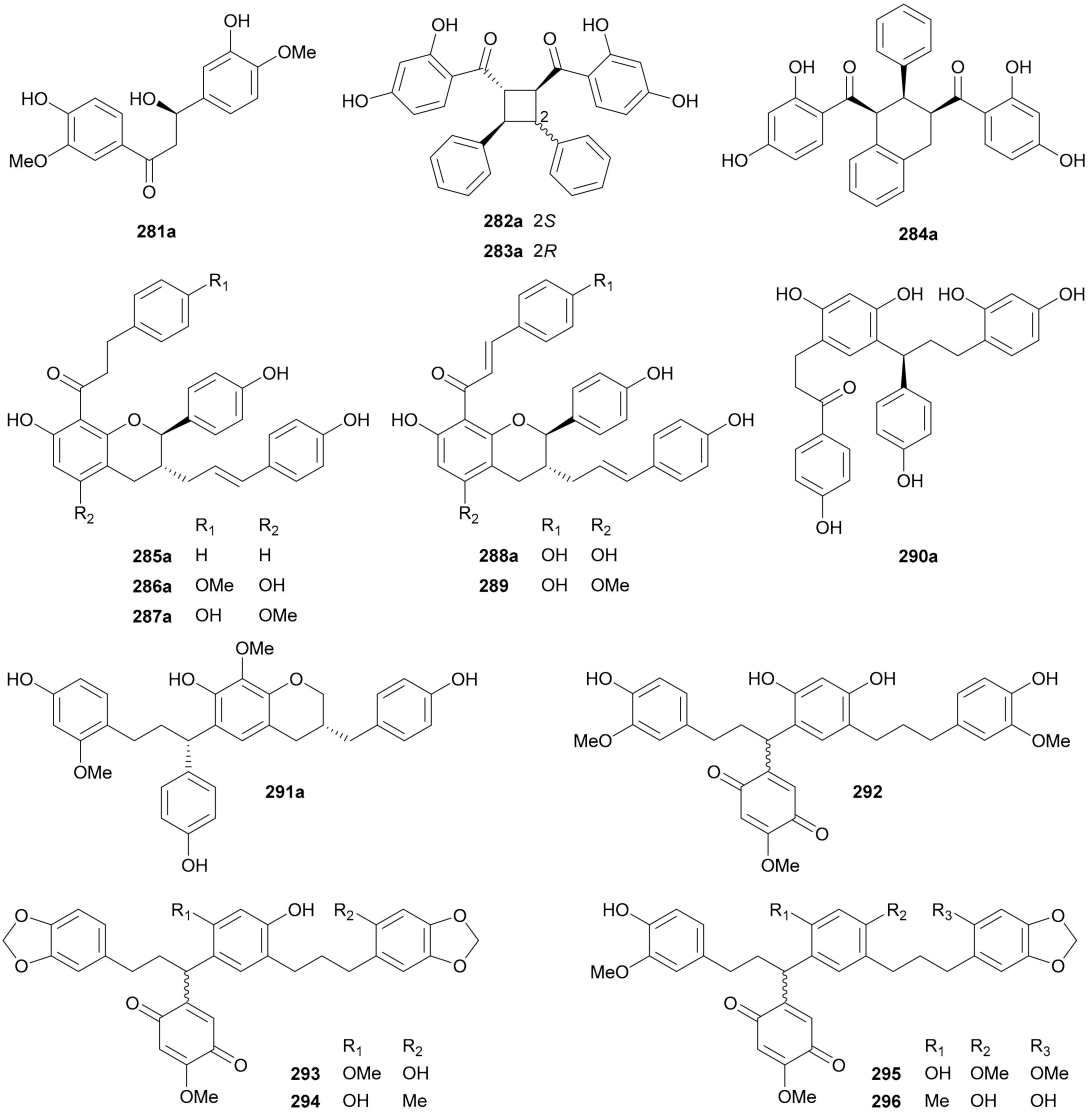
Structures of chalcones.

Chalcones are open-chain flavonoids which have attracted increasing attention from researchers due to the wide range of their bioactivities, including antimicrobial, antimalarial, anticancer, anti-inflammatory, antiprotozoal, anti-HIV, antioxidant properties, etc. [150]. The chalcone enantiomers covered by this review, including monomers and dimers, are summarized in Figure 16 (Names see Table S16 in Supplementary Materials). Similarly, the determination of abs. configs. by TDDFT-ECD method will not be specified.

Compounds **281a**/**281b** are a pair of dihydrochalcone enantiomers from *Pteris ensiformis* [151] with the abs. configs. being determined by Rh_2_(O_2_CCF_3_)_4_-induced ECD method. Zhang and coworkers reported three chalcone dimers formed by [2 + 2] (**282a**/**282b** and **283a**/**283b**) and [2 + 4] (**284a**/**284a**) cycloaddition reactions from *Oxytropis chiliophylla* in 2018 [152]. The hydroxycinnamoylated chalcones, including four pairs of separated enantiomers **285a**/**285b**−**288a**/**288b** and one racemate **289**, were obtained from *Populus balsamifera*, with the abs. configs. for **285b** being established by single-crystal X-ray diffraction analysis [153]. Li and coworkers reported two pairs of enantiomeric dimers formed by a dihydrochalcone and a deoxohydrochalcone (**290a/290b**) and by a deoxohydrochalcone and a homoisoflavane (**291a**/**291b**) from *Dracaena cochinchinensis* in 2016 [147]. From *Horsfieldia tetratepala*, compounds **292**−**296** were obtained as scalemic deoxohydrochalcone dimers without chiral separation [154].

#### 2.5.3. Xanthones

**Figure 17:**
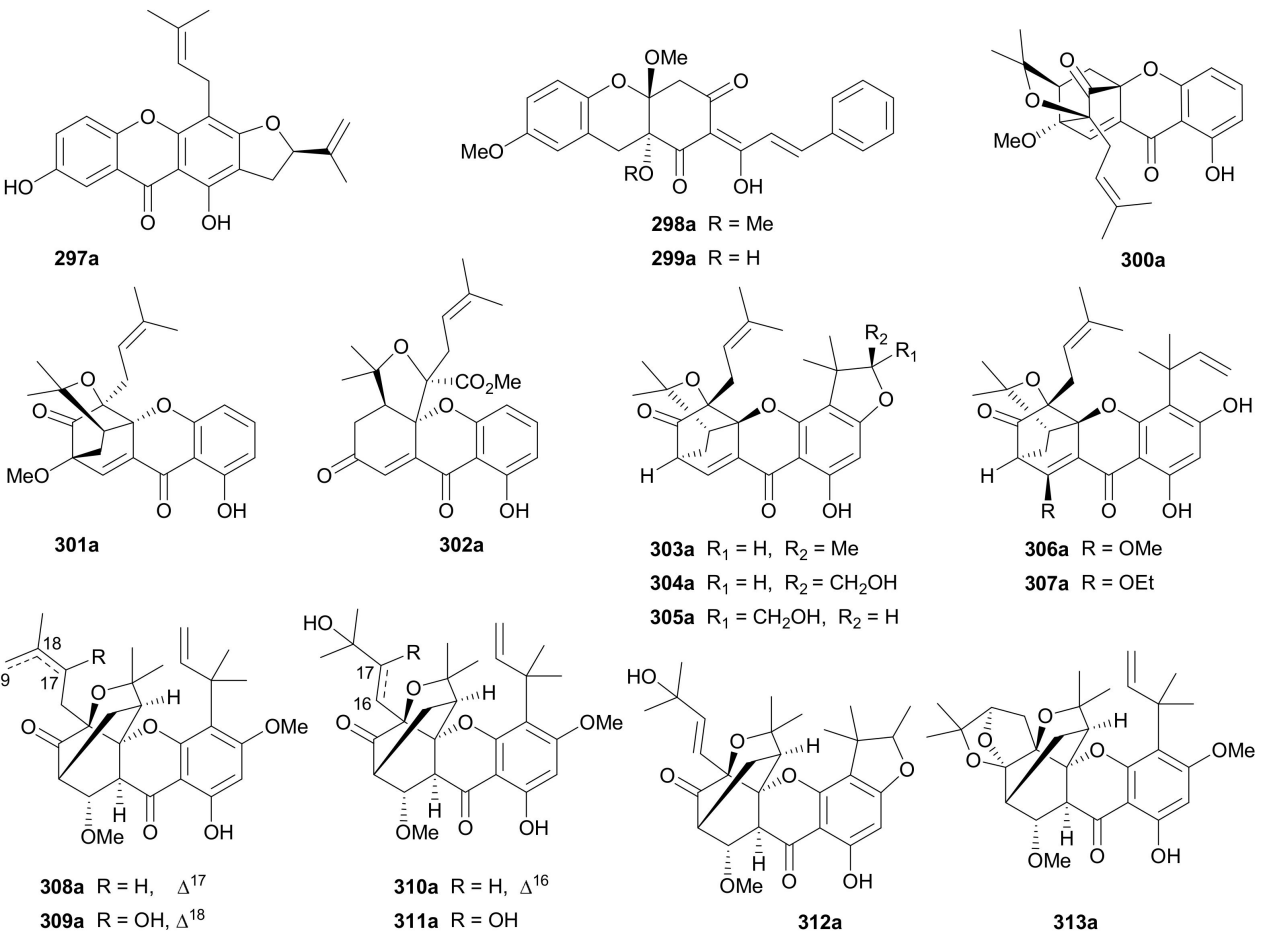
Structures of xanthones.

Xanthones are polyphenolic compounds incorporating a common 9*H*-xanthen-9-one scaffold with various substituents, making them ‘privileged structures’ which are likely to bind to a variety of biological targets. They have been shown to display significant bioactivities including antimicrobial, antioxidant, cytotoxic activities, and so on [155]. Most xanthone enantiomers reported in this period are prenylated; their structures are listed in Figure 17 (names see Table S17 in Supplementary Materials).

**Scheme 4:**
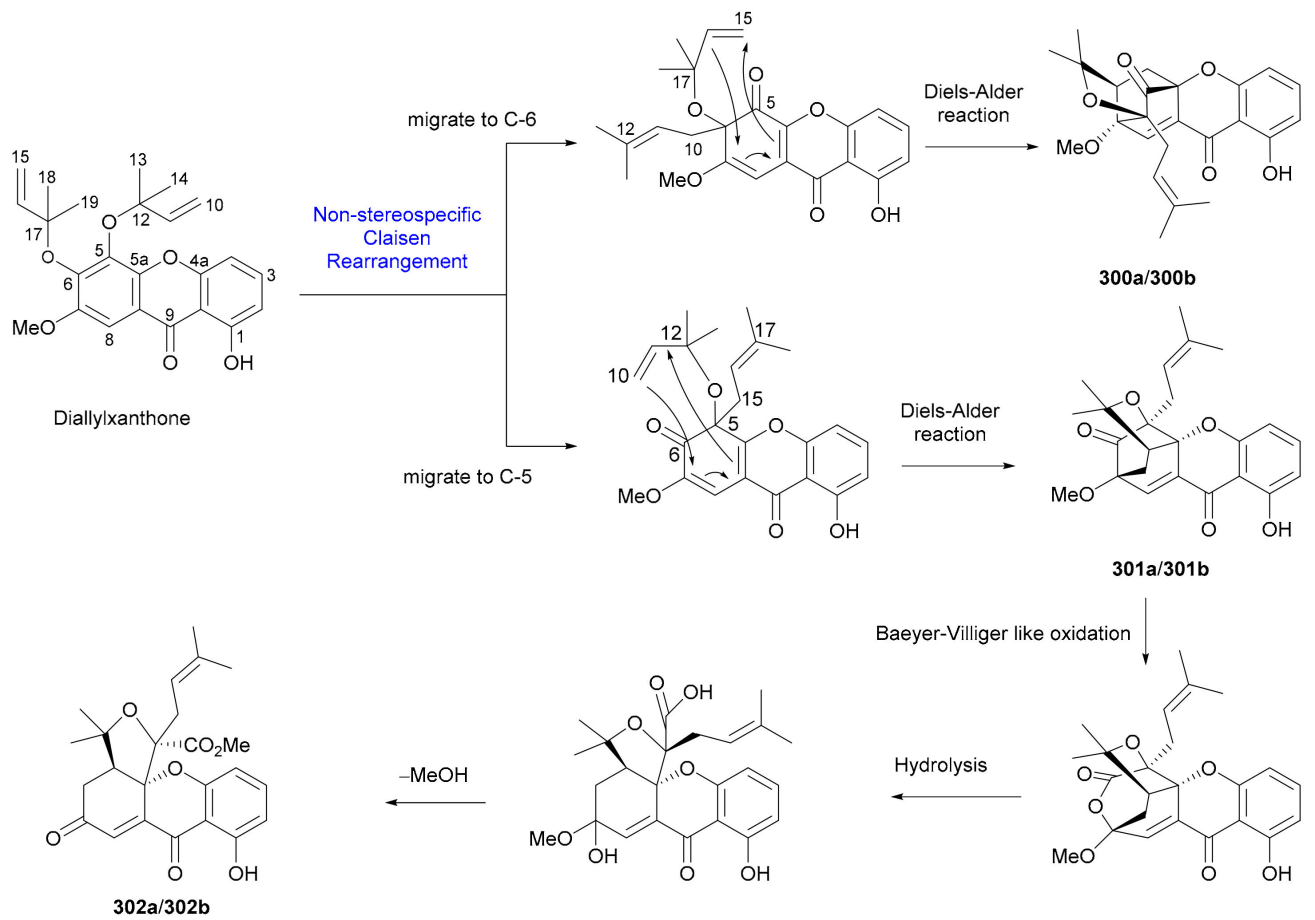
Plausible biosynthetic pathways for **300a**/**300b**−**302a**/**302b**.

Hua and coworkers reported a pair of diprenylated xanthone enantiomers **297a**/**297b** with only one chiral center from *Cratoxylum cochinchinense* in 2019 [156]. The deoxoxanthone enantiomers **298a**/**298b** and **299a**/**299b** incorporating a phenylpropanoid unit were isolated from *Uvaria valderramensis* in 2014 [157]. Also in 2014, three pairs of prenylxanthone enantiomers (**300a**/**300b**−**302a**/**302b**) were isolated from *Cratoxylum formosum*, with the abs. configs. being established by X-ray crystallographic experiment [158]. As shown in Scheme 4, the generation of enantiomeric **300a**/**300b**−**302a**/**302b** is plausibly derived from diallylxanthone through a key process of nonstereospecific Claisen rearrangement [158]. In addition to **300**−**302**, eleven pairs of caged prenylxanthone enantiomers **303a**/**303b**−**307a**/**307b** and **308a**/**308b**−**313a**/**313b** were reported from *Garcinia bracteata* in 2018 [159] and from *Garcinia propinqua* in 2017 [160], respectively, with the abs. config. of **313a** being determined by single-crystal X-ray diffraction analysis. The biogenetic origins of those xanthones with multiple chiral centers are indeed interesting topics that deserves further investigations.

### 2.6. Terpenoids

Terpenoids are probably the biggest family of NPs with diverse structures and various biological activities [3]. All terpenoids are initially assembled from the head-to-tail condensation of repeated isoprene units (C_5_), and according to the number of isoprene residues, terpenoids are normally classified into monoterpenoids (C_10_), sesquiterpenoids (C_15_), diterpenoids (C_20_), sesterterpenoids (C_25_) and triterpenoids (C_30_). Additionally, meroterpenoids are also an interesting class of terpenoid products with mixed biogenesis [3]. To the best of our knowledge, enantiomeric cases have been reported for all terpenoid subclasses except triterpenoids.

#### 2.6.1. Sesquiterpenoids

**Figure 18:**
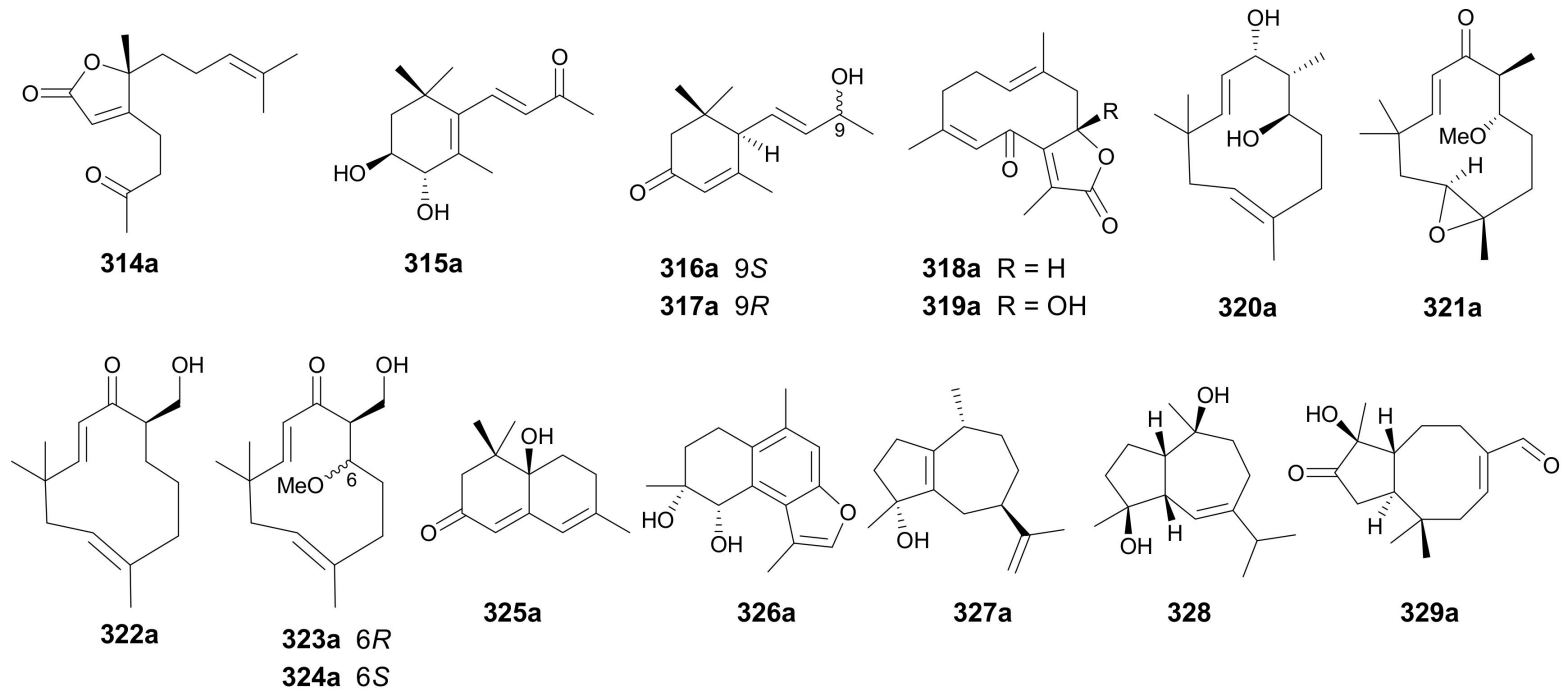
Structures of sesquiterpenoids.

Sesquiterpenoids are constructed from three isoprenyl fragments. Among all the terpenoid classes, they have the most diverse carbon skeletons and are probably the largest group of terpenoid NPs. Corresponding to their various structural types, natural sesquiterpenoids have also exhibited a myriad of biological properties [161], and this has been well reflected by the success of artemisinin (for malaria), the most famous sesquiterpenoid whose discovery was rewarded the Nobel prize in Physiology or Medicine in 2015. Enantiomeric sesquiterpenoids reported in this period include 16 pairs (**314**−**329**) with different backbones (for structures, see Figure 18, names see Table S18 in Supplementary Materials).

Enantiomers **314a**/**314b** represent the first examples of 1,2-*seco* bisabolane-type sesquiterpenoid lactones from *Artabotrys hexapetalus*, with the abs. configs. being determined by employing the helicity rule to analyze the Cotton effect at around 220 nm [162]. Qiu and coworkers reported four pairs of megastigmane-type norsesquiterpenoid enantiomers **315a**/**315b**−**317a**/**317b** and **325a**/**325b** from *Eucommia ulmoides* in 2017, while the racemic mixtures of **315** and **325**, along with pure enantiomers **316b** and **317b**, had been previously reported [163]. Compounds **318a**/**318b** and **319a**/**319b** are two pairs of enantiomeric germacrane type sesquiterpenes from *Curcuma phaeocaulis* reported in 2017 [128]. Chai and colleagues discovered **320a**/**320b**−**324a**/**324b** with a humulane framework and **329a**/**329b** incorporating a rare 2,2,5,9-tetramethylbicyclo[6.3.0]-undecane skeleton from *Syringa pinnatifolia* [164]. The abs. configs. of these compounds were established by single-crystal X-ray diffraction analysis, modified Mosher’s method and TDDFT-ECD calculation [164]. Compounds **326a**/**326b** and **327a**/**327b** were isolated from *Commiphora myrrha* [165] and *Daphne genkwa* [166], respectively. The guaianolide sesquiterpenoid **328** was reported as a racemic mixture as indicated by X-ray crystallography from *Kadsura interior* in 2013 [167].

#### 2.6.2. Diterpenoids

**Figure 19:**
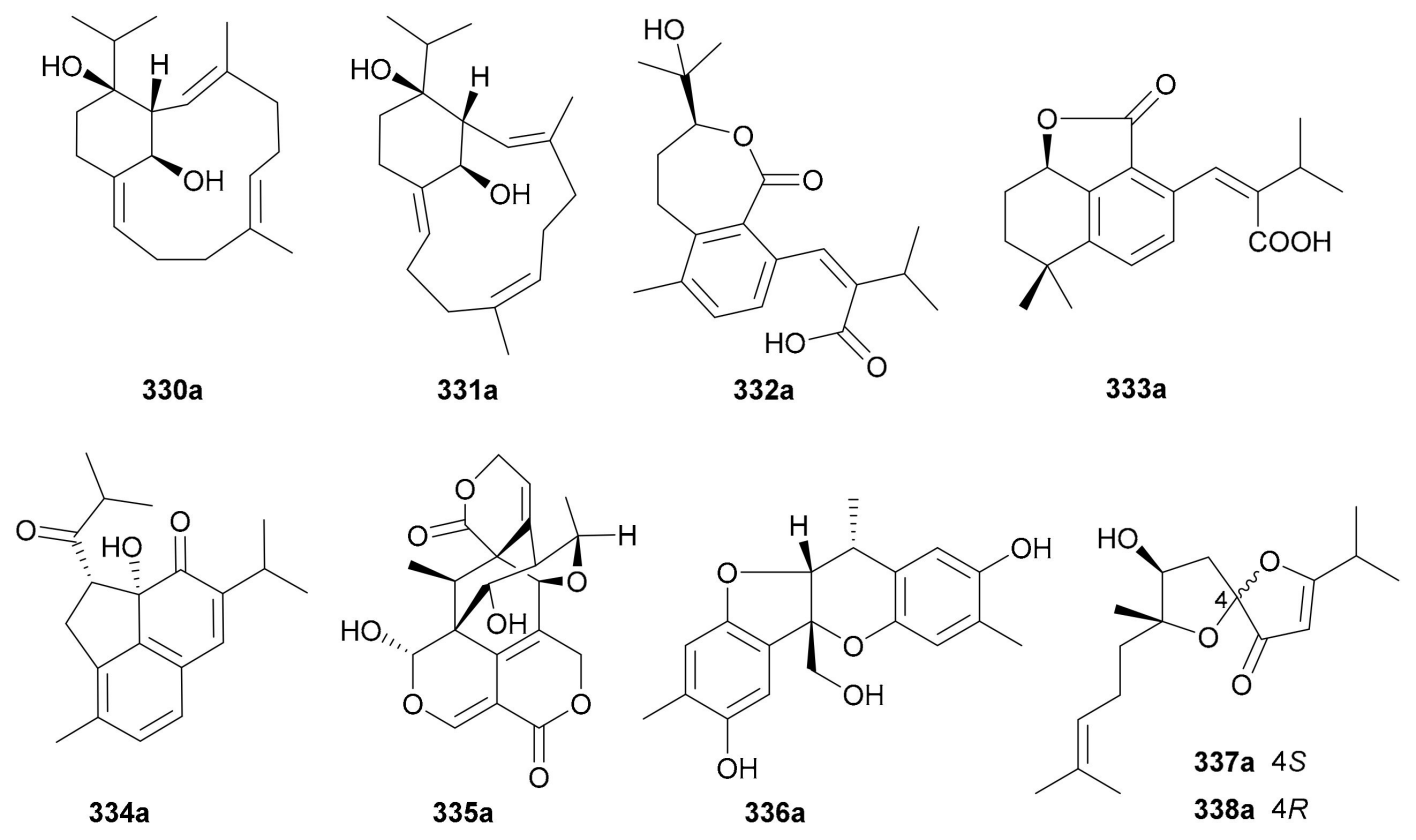
Structures of diterpenoids.

Diterpenoids are biosynthesized from the head-to-tail condensation of four isoprene (C_20_) units, and have the second largest number of carbon backbones in the terpenoid family. Like sesquiterpenoids, they are also well-known in the NP community for their diverse bioactivities particularly antitumor effects with therapeutic values [168]. As is well known, the most notable diterpenoid is taxol, which has been used as a cancer treatment for over three decades. Nine pairs of enantiomeric diterpenoids **330a**/**330b**−**338a**/**338b** (for structures, see Figure 19, names see Table S19 in Supplementary Materials) with different structural skeletons were recorded in the study period.

Yue and coworkers phytochemically investigated *Croton mangelong* to afford two pairs of macrocyclic diterpenoid enantiomers **330a**/**330b** and **331a**/**331b** featuring a bicyclo[9.3.1]pentadecane core and a rare bridgehead double bond, with the abs. config. for **330b** being determined by single-crystal X-ray diffraction analysis [169]. Compounds **332a**/**332b** are bis-*seco*-abietane diterpenoids from *Cryptomeria fortune* and were asymmetrically synthesized through a readily made intermediate orthoquinone from sugiol [170]. Compounds **333a**/**333b** are a pair of norditerpenoid enantiomers from the roots of *Salvia miltiorrhiza* [171]. Compounds **334a**/**334b**, rearranged abietane-type diterpeniods featuring a 5/6/6 tricyclic architecture with the five-membered ring formed via C-2–C-11 single bond, were isolated and characterized from *Salvia prionitis* in 2015 [171]. Compounds **335a**/**335b** are a pair of diterpenoid enantiomers with a highly oxygenated novel backbone obtained from *Swertia leducii* in 2014, with the rel. config. being determined by X-ray diffraction analysis and the abs. configs. by TDDFT-ECD method [172]. In the same year, compounds **336a**/**336b** were isolated from *Paeonia veitchii* [173], and they incorporate an aromatized norditerpenoid skeleton, with the rel. config. being confirmed by X-ray crystallography. Compounds **337a**/**337b** and **338a**/**338b**, two pairs of norditerpenoid enantiomers with unusual 5,5-spiroketal core, were obtained from *Hypericum japonicum* in 2016, with the abs. configs. being assigned by a combination of TDDFT-ECD calculation, modified Mosher’s method and quantum chemical predictions (QCP) of ^13^C NMR data [174].

#### 2.6.3. Meroterpenoids

**Figure 20:**
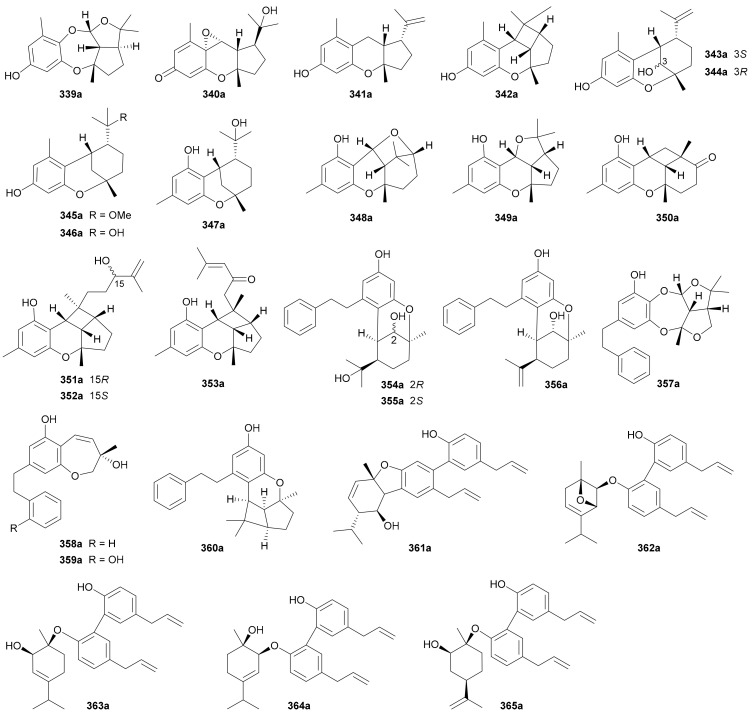
Structures of meroterpenoids.

The term meroterpenoid was first proposed by Cornforth in 1968 to describe NPs of mixed biosynthetic origin which are partially derived from terpenoids [175]. Enantiomeric meroterpenoids reported in the covered period are exclusively formed by the condensation of phenolic compounds with a monoterpenoid or a sesquiterpenoid via at least one ether bond, and the chirality generating enantiomerism all exists in the terpenoid part except for **340**. There are 27 pairs of enantiomeric meroterpenoids reported in this period (for structures, see Figure 20; for names, see Table S20).

Compounds **339a**/**339b**−**346a**/**346b** are eight pairs of enantiomeric meromonoterpenoids with diverse heterocyclic frameworks from *Rhododendron nyingchiense* [176]. Among them, **339a**/**339b** possess a rare 6/7/5/5 heterocyclic ring system, while **340a**/**340b** incorporate a 6/6/5 tricyclic backbone with an extra oxirane ring coupled to the quinone motif. The assignments of abs. configs. for **339a** and **341a** were based on X-ray crystallographic experiment, while those for the others were via TDDFT-ECD method. Four enantiomeric meromonoterpenoid pairs **347a**/**347b**−**350a**/**350b**, along with three merosesquiterpenoid pairs **351a**/**351b**−**352a**/**352b**, were isolated and identified from *Rhododendron capitatum* by Hou and coworkers [177,178]. Of these interesting molecules, compounds **348** and **349** bear unprecedented 6/6/6/5 and 6/6/5/5 ring systems, respectively, while **350** and **351**−**333** possess unique 6/6/6/4 and 6/6/5/4 heterocyclic architectures, respectively. In addition, the abs. configs. of **350**−**353** pairs were confirmed by X-ray diffraction and ECD analyses. Compounds **354a**/**354b**−**360a**/**360b**, seven pairs of bibenzyl-based meroterpenoid enantiomers, were obtained from the Chinese liverwort *Radula sumatrana* in 2017 by Lou’s group [179]. Compounds **361a**/**361b**−**365a**/**365b** are five pairs of magnolol-derived lignan-monoterpenoid hybrid enantiomers that have been isolated from *Magnolia officinalis* in 2019 [180].

### 2.7. Phloroglucinols

**Figure 21:**
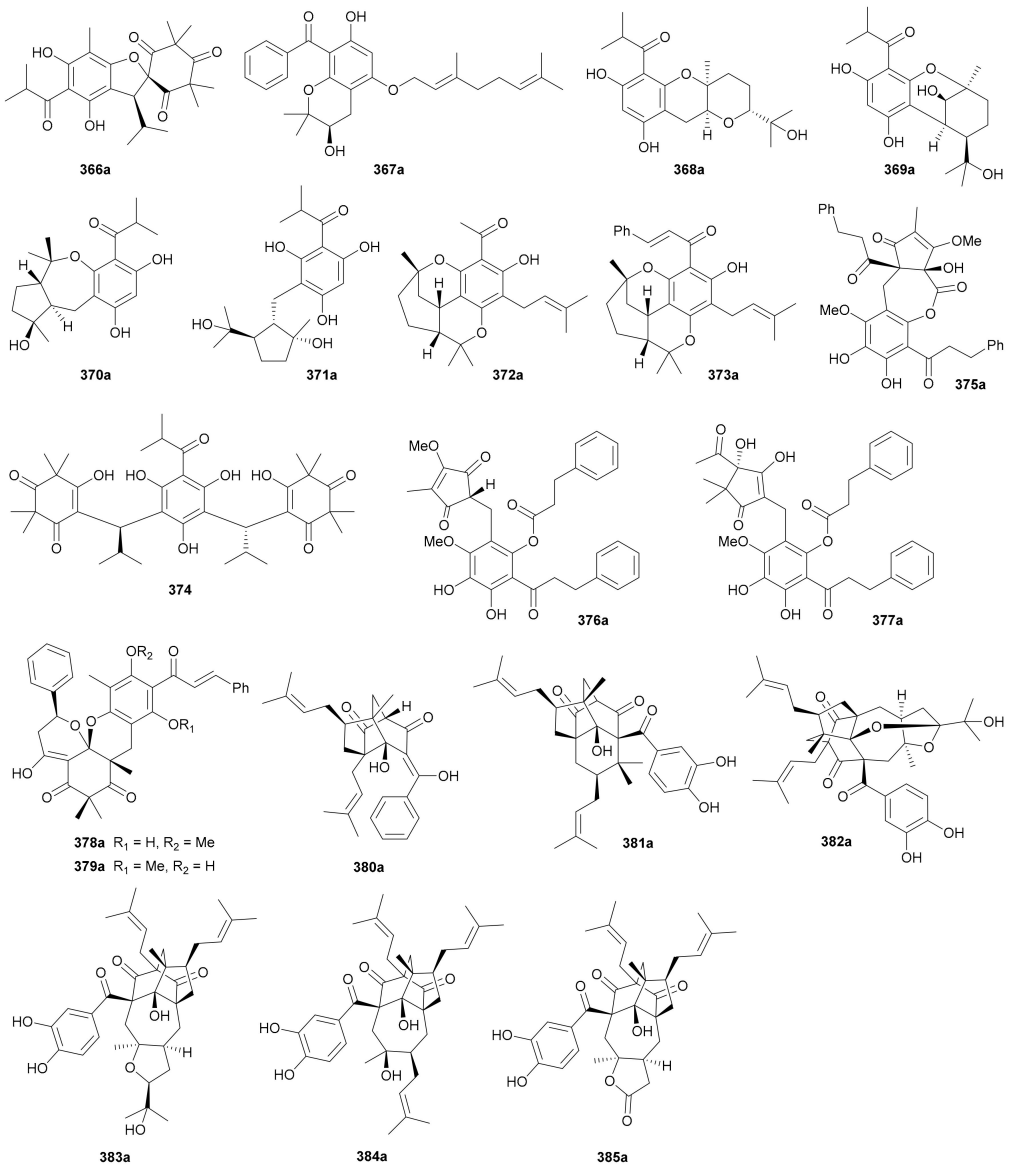
Structures of phloroglucinols.

Phloroglucinol derivatives represent a unique class of NPs featuring one or more intact or modified phloroglucinol units, with alkylation and acylation as the common structural modifications [181]. The chirality of them arises usually from their prenyl/terpenyl substituents and/or from the dearomatization of phloroglucinol core. In most cases, they can also be classified into the ‘meroterpenoid’ group, but here we describe them separately owing to the considerable number of reports and their popularity among NP workers in recent years. Phloroglucinal enantiomers reported in this period all incorporate one or more acyl groups including acetyl, isobutyryl, benzoyl, cinnamoyl and dihydrocinnamoyl, and their structures are shown in Figure 21 (names see Table S21 in Supplementary Materials). Wherever the abs. configs. are determined by the TDDFT-ECD method, it will be not specifically mentioned in this section.

**Scheme 5:**
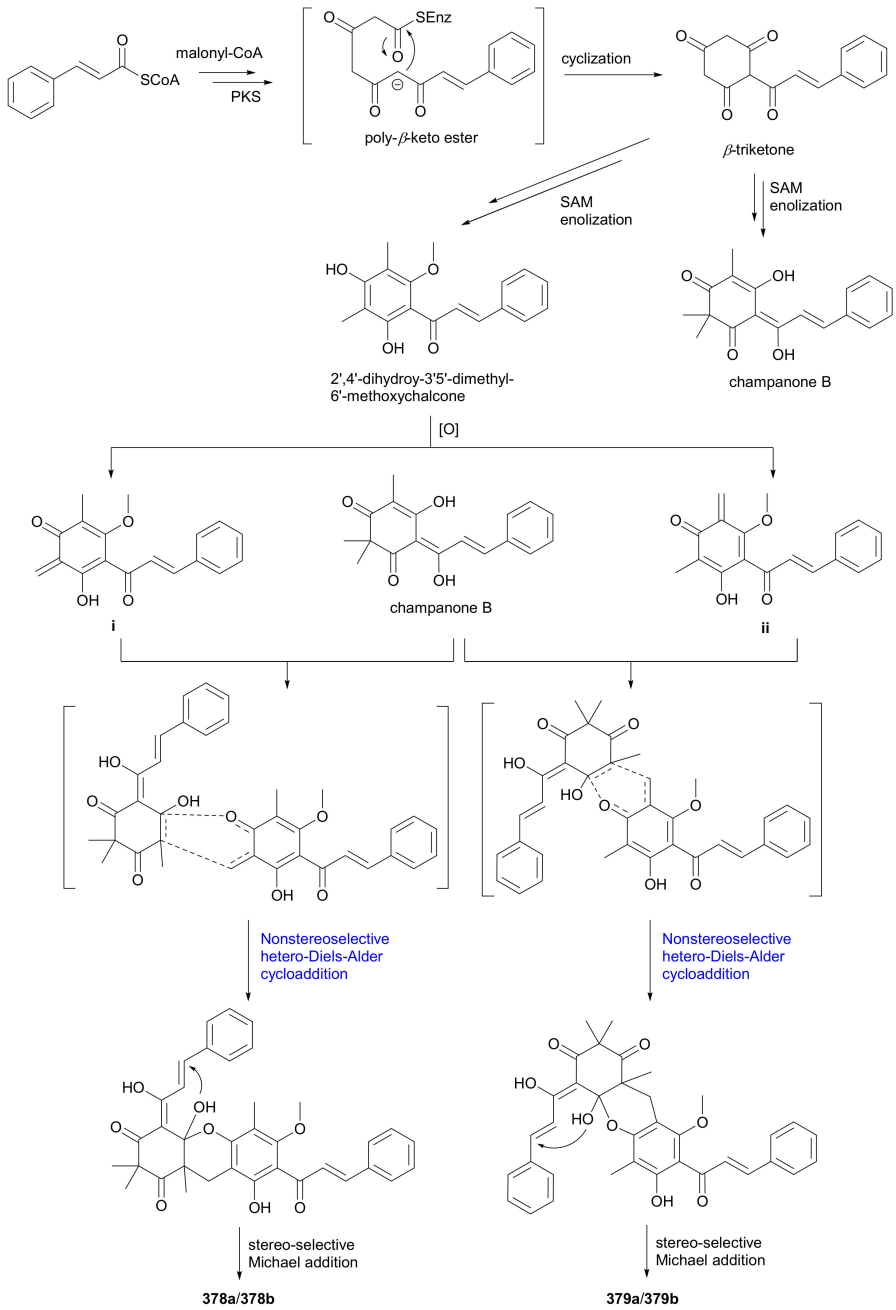
Plausible biosynthetic pathways for **378a**/**378b** and **379a**/**379b**.

**Scheme 6:**
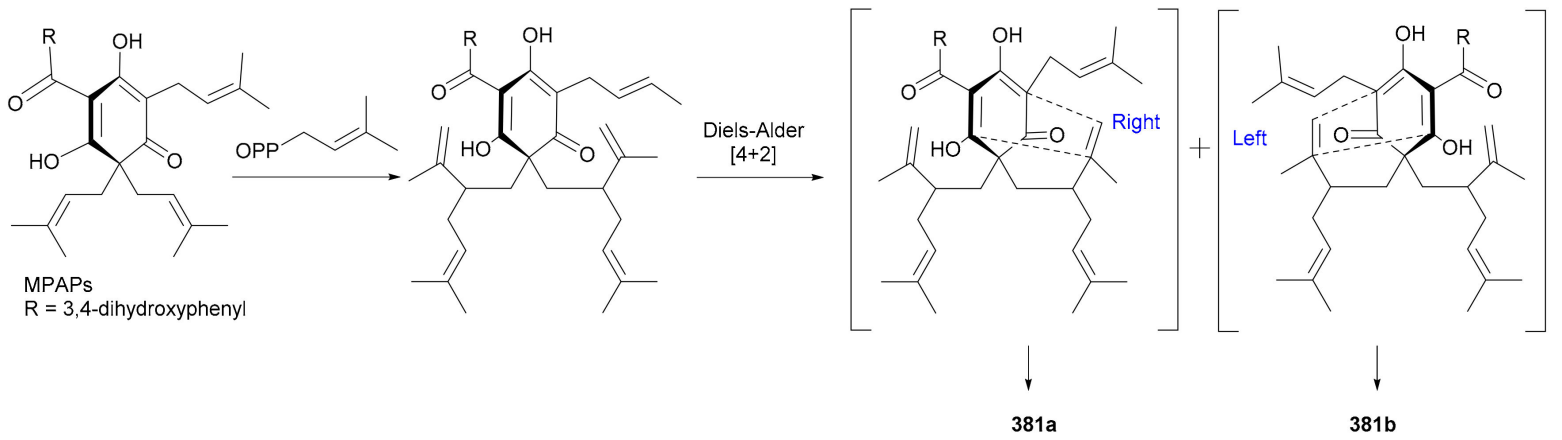
Plausible biosynthetic pathways for **381a**/**381b**.

Ye and coworkers reported a pair of enantiomeric isobutyrylated phloroglucinol dimers **366a**/**366b** from *Myrtus communis* in 2019 and also completed their total synthesis in the same year [182]. Laphookhieo and colleagues discovered the benzoylated phloroglucinol enantiomers **367a**/**367b** from *Cratoxylum sumatranum* ssp. *Neriifolium* and assigned their abs. configs. by comparing the specific optical rotations with those of known analogues [183]. Compounds **368a**/**368b**−**371a**/**371b**, as phloroglucinol-monoterpenoid hybrids, were reported from *Hypericum japonicum* in 2016, with the abs. configs. of **370b** and **371b** being determined by single-crystal X-ray diffraction analysis [184]. Compounds **368a**/**368b** and **369a**/**369b** incorporate interesting pyrano[3,2-*b*]pyran and 2-oxabicyclo[3.3.1]nonane skeletons, respectively, while **370a**/**370b** possess a benzo[*b*]cyclopenta[*e*]oxepine ring system. Laphookhieo and coworkers also obtained acetylated (**372a**/**372b**) and cinnamoylated (**373a**/**373b**) phloroglucinol enantiomers from *Mallotus philippensis* in 2019 and established their abs. configs. on the basis of X-ray crystallographic studies [185]. Hans et al. re-acquired myrtucommulone A (**374**) from *Myrtus communis* in 2015 and proved that, by converting it into separable derivatives, **374** consisted of the racemate and the meso form in a ca. 1:1 ratio [186]. Compounds **375a**/**375b**−**377a**/**377b**, dihydrocinnamoylated and rearranged phloroglucinol dimers, were isolated from *Xanthostemon chrysanthus* in 2019 [187]. Compounds **375a**/**375b** feature an bis-phenylpropanoyl-benzo[*b*]cyclopent[*e*]oxepine tricyclic backbone and **376a**/**376b** and **377a**/**377b** represent the first examples of 1-(cyclopentylmethyl)-3-(3-phenylpropanoyl)benzene scaffold [187]. Compounds **378a**/**378b** and **379a**/**379b**, cinnamoylated phloroglucinol dimers from *Cleistocalyx operculatus*, possess a polycyclic skeleton with a highly functionalized dihydropyrano[3,2-*d*]xanthene tetracyclic core, and the enantiotropy could be derived from the nonstereoselective hetero-Diels-Alder [4 + 2] cycloaddition as shown in the proposed plausible biosynthetic pathway (Scheme 5) [188]. The benzoylated phloroglucinol **380a** was isolated from *Triadenum japonicum* in 2015 [189] and identified as the enantiomer of (+)-nemorosonol (**380b**) previously reported from *Clusia nemorosa* [190]. Compounds **381a**/**381b** are a pair of digeranylated phloroglucinol enantiomers from *Garcinia multiflora* and feature a caged tetracyclo[5.4.1.1^1,5^.0^9,13^]tridecane skeleton, with the generation of enantiomerism being likely from the intramolecular Diels-Alder [4 + 2] cycloaddition on different sides as shown in Scheme 6 [191]. Compounds **382a**/**382b**−**385a**/**385b**, four similar type of enantiomer pairs as **381**, were obtained from *Garcinia multiflora*, and **382a**/**382b** are characterized by the coupling of two novel caged fragments, i.e., 2,11-dioxatricyclo[4.4.1.0^3,9^]undecane and tricyclo[4.3.1.0^3,7^]decane, with the rel. config. being determined by X-ray diffraction analysis [192].

### 2.8. Naphthalenes and Phenanthrenes

**Figure 22:**
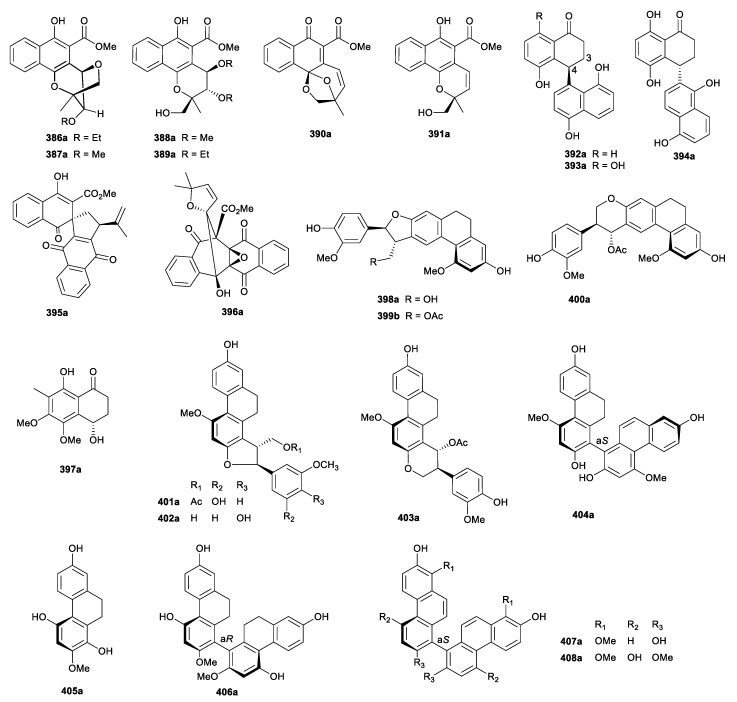
Structures of naphthalene and phenanthrenes.

The enantiomerism of naphthalene and phenanthrene derivatives is generally attributable to chiral centers and, in many cases, axial chirality. The structures of the naphthalene and phenanthrene enantiomers reported in this period are displayed in Figure 22 (names see Table S22 in Supplementary Materials). The abs. configs. of most of these enantiomers were determined by the TDDFT-ECD method, unless otherwise specified.

#### 2.8.1. Naphthalenes

Compounds **386**−**397** are 12 pairs of naphthalene enantiomers reported in this period. Among them, **386**−**391** were isolated from the roots of *Morinda officinalis* var. *officinalis* and identified as prenylated methyl 2-naphthoates by the authors’ team, with the rel. configs. of **386** and **388** being confirmed by X-ray diffraction analysis and ^13^C NMR calculation, respectively [8]. Compounds **392**−**394**, three pairs of 3,4-dihydro-4-naphthyl-naphthalen-1(2*H*)-one enantiomers, were obtained from *Juglans regia* in 2019 [193]. Compounds **395a**/**395b** from *Rubia oncotricha* were characterized by Tan and coworkers as novel naphthoquinone dimers with an unprecedented spiro[4.5] carbon core [194]. Another pair of dimeric naphthoquinone enantiomers **396a**/**396b** were also reported by the same research team from *Rubia alata* in 2014, with the rel. config. being corroborated by X-ray crystallographic experiment [195]. Compounds **397a**/**397b** are simple tetrahydronaphthoquinone enantiomers reported from *Eremurus altaicus* in 2015 [196].

#### 2.8.2. Phenanthrenes

Phenanthrene enantiomers (**398a**/**398b**−**408a**/**408b**) in this period have been solely reported from *Bletilla striata* by Li’s and Hou’s research teams in 2019 [130], and they can be divided into three groups, namely, phenanthrene monomer (**405**), phenanthrene dimers (**404** and **406−408**) and phenanthrene-phenylpropanoid hybrids (**398**−**403**). The enantiomerism of these compounds has been generated from the axial chirality of phenanthrene moiety and/or from the chiral centers of phenylpropanoid unit. Notably, compounds **404**−**408** with only axial chirality were able to be separated into five pairs of enantiomers. Axial chirality, although well known to organic chemists, has often been overlooked by NP researchers, owing to its rare presence in natural molecules. Therefore, the enantiomeric purity of NPs with axial chirality is strongly recommended to be checked no matter they are new or known.

### 2.9. Chromanes

**Figure 23:**
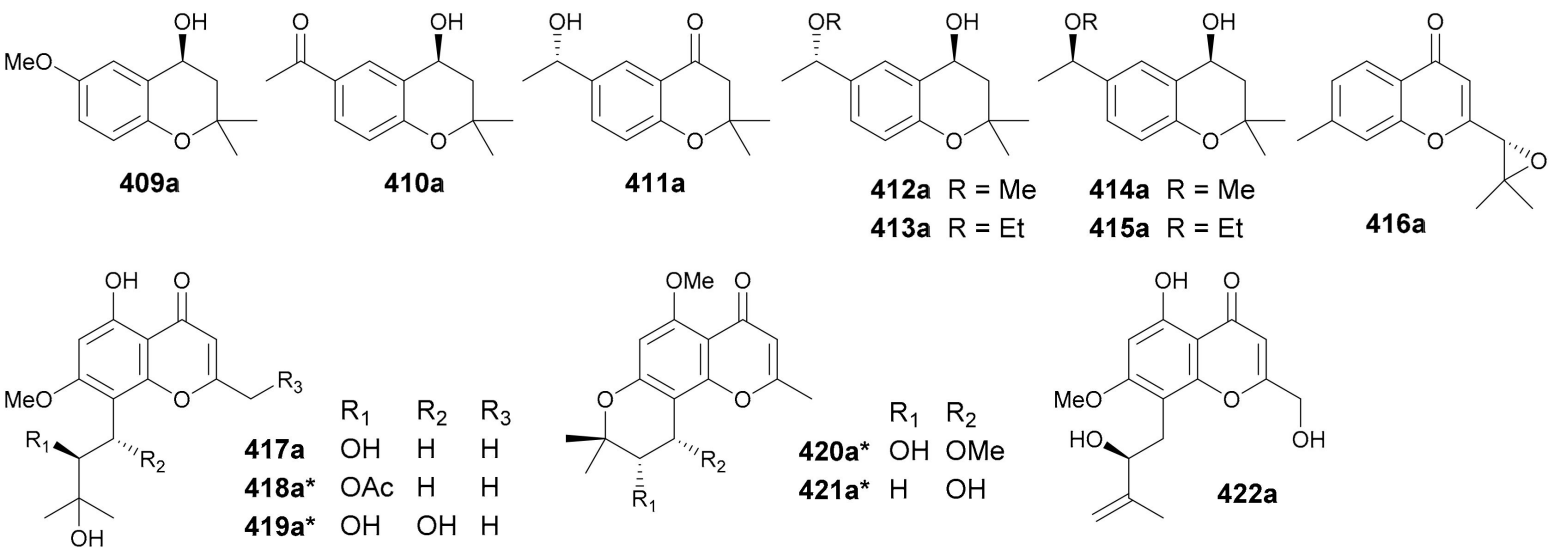
Structures of chromanes (* abs. configs. undetermined).

Chromane derivatives are a class of NPs having a chromane core or a modified one (e.g., chromone, chromanone) in their structures. Enantiomeric chromane derivatives reported in this period are listed in Figure 23 (names see Table S23 in Supplementary Materials). Compounds **409a**/**409b**−**415a**/**415b** had been studied previously in many occasions as pure enantiomers, scalemic mixtures or racemates, and as natural molecules, biotransformation products or synthetic intermediates, but none of these reports had paid attention to the enantiomerism of this group of structures. They were separated from the flower buds of *Tussilago farfara* in the authors’ lab in 2018, with the abs. configs. being determined by chemical method as well as TDDFT-ECD calculation and ECD comparison [7]. Compounds **416a**/**416b** were proposed to be a pair of norbisabolane sesquiterpenoid enantiomers yet incorporating a chromone core and were obtained from *Curcuma longa* in 2019 [197]. Compounds **417a**/**417b**−**421a**/**421b** are five pairs of prenylated chromone enantiomers isolated from *Harrisonia perforate* in 2014, whereas only the abs. configs. of **417a**/**417b** were assigned by Mosher’s method [198]. From the same plant, **422a**/**422b** were reported as a pair of enantiomeric molecules by Yuan et al. in 2017 [199].

### 2.10. Acetophenones

**Figure 24:**
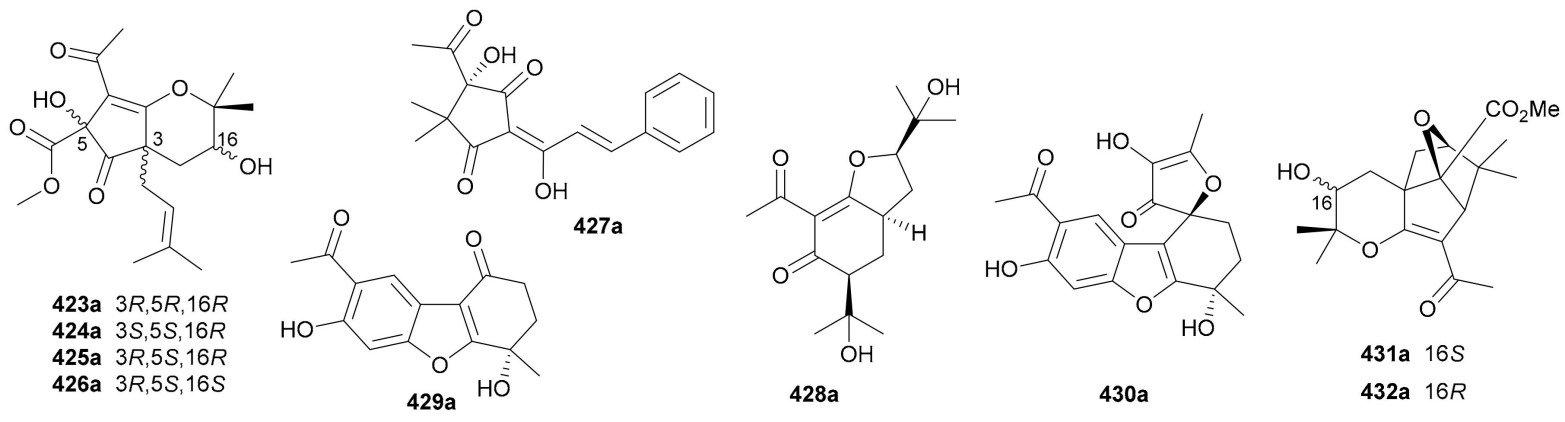
Structures of acetophenones.

Acetophenones are a rare class of NPs bearing normal or rearranged acetophenone units in their structures. To date, ten pairs of acetophenone enantiomers have been reported, and their structures are depicted in Figure 24 and names listed in Table S24 in Supplementary Materials. Kong and coworkers reported four pairs of diprenylated and rearranged acetophenone enantiomers **423a**/**423b**−**426a**/**426b** from the leaves of *Melicope ptelefolia* in 2019 and assigned their abs. configs. by a combination of modified Mosher’s and TDDFT-ECD methods [200], while **423a**/**423b** and **424** in racemic form had been previously isolated from *Evodia lepta* by Tang et al. in 2018 [201]. Also identified as rearranged acetophenones, compounds **427a**/**427b** coupled with a phenylpropanoid fragment were isolated from *Xanthostemon chrysanthus* in 2019 [187]. Compounds **428a**/**428b** are prenylated hydroacetophenone enantiomers obtained from *Melicope viticina* in 2019 [202], while **429a**/**429b** and **430a**/**430b** with intact acetophenone unit were discovered from *Eupatorium chinense* in 2013 [203]. Compounds **431a**/**431b** and **432a**/**432b**, rearranged acetophenones with a novel 9-oxatricyclo[3.2.1.1^3,8^]nonane core from *Melicope ptelefolia*, were assigned the abs. configs. by single-crystal X-ray diffraction analysis [204].

### 2.11. Diarylheptanoids

**Figure 25:**
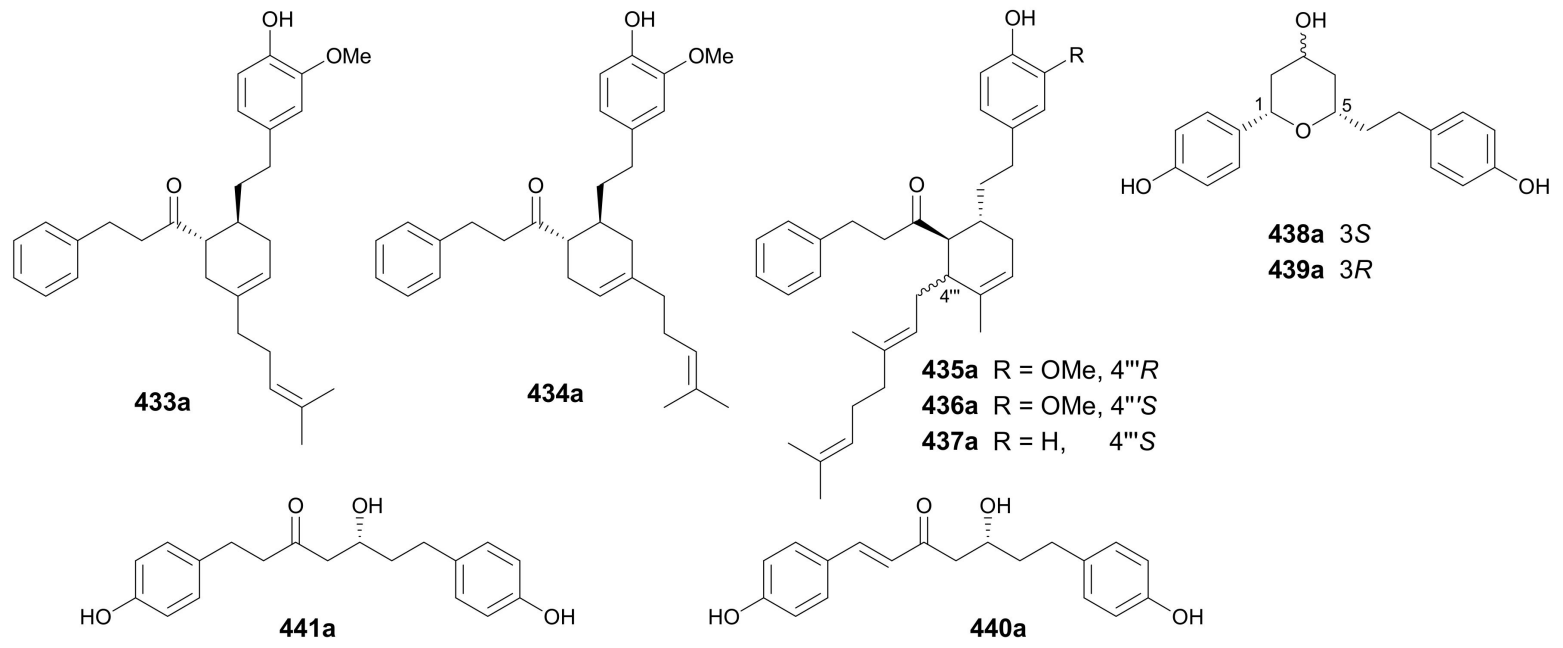
Structures of diarylheptanoids.

Diarylheptanoids, a class of NPs characteristic of a 1,7-diphenylheptane core, have been increasingly recognized as potential therapeutic agents for their diverse biological properties including antiinflammatory, antitumor, antioxidant, antiestrogen, hepatoprotective, antileishmania and neuroprotective activities [205]. Nine pairs of diarylheptanoid enantiomers (**433**−**441**, for structures, see Figure 25; for names, see Table S25 in Supplementary Materials) have been documented in the covered period. The occurrence of enantiomerism in these compounds results from the chiral centers generated by oxidation or Diels-Alder cycloaddition with other molecules.

The enantiomeric pairs of diarylheptanoid-monnoterpene adduct (**433** & **434**) and diarylheptanoid-sesquiterpene hybrid (**435**–**437**) from *Alpinia officinarum*, were hypothesized to be produced via a crucial Diels-Alder cycloaddition between the diarylheptanoids and corresponding terpenyl units. The rel. configs. for the chiral centers in the cyclohexene ring were assigned by comparing the experimental and calculated ^13^C NMR data, followed by the establishment of the abs. configs. via the TDDFT-ECD method [206]. Compounds **438**−**441** are four pairs of diarylheptanoid enantiomers acquired from *Dioscorea villosa* in 2012, with the abs. configs. being determined by the modified Mosher’s method [207]. Compounds **438** and **439** incorporate an extra tetrahydropyran ring formed via C-1 and C-5, while **440** and **441** are normal linear examples.

### 2.12. Triphenylmethanes

**Figure 26:**
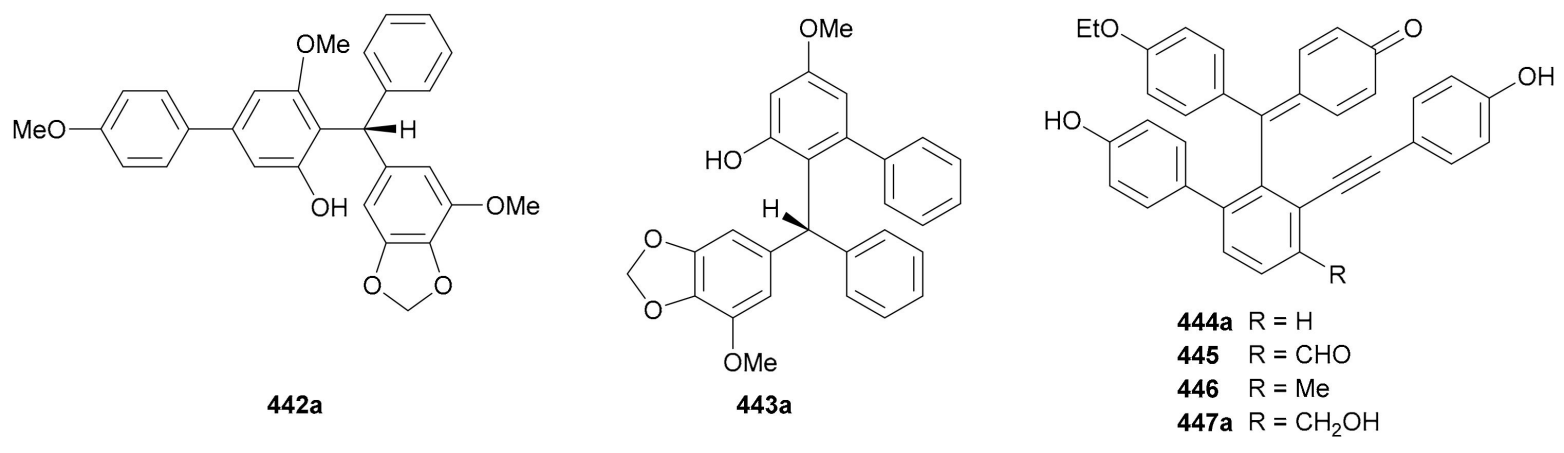
Structures of triarylmethanes.

Triphenylmethanes are a unique class of NPs with one central carbon being linked by three aryl groups. They have been discovered to have a wide range of biological activities including antioxidant, antitumor, anti-HPK (histidine protein kinases) activities, etc. [208]. Six pairs of triphenylmethane enantiomers (**442−447**, Figure 26, Table S26 in Supplementary Materials) were reported in this period; their enantiomerism is attributable to chiral centers (**442** and **443**) or axial chirality (**444−447**).

Compounds **442a**/**442b** and **443a**/**443b** are two pairs of triarylmethane enantiomers reported from *Securidaca inappendiculata* in 2018, with the abs. configs. being determined by X-ray crystallography [209]. In addition, bio-inspired total syntheses for these compounds were also completed [209]. Compounds **444**−**447** occurred as racemates generated by axial chirality in the plant *Selaginella pulvinate* [210], and subsequent chiral fractionation divided **444** and **447** into **444a**/**444b** and **447a**/**447b**, respectively, with the abs. configs. being assigned by TDDFT-ECD method. However, **445** and **446** had not been enantiomerically separated.

### 2.13. Fatty Acids

**Figure 27:**
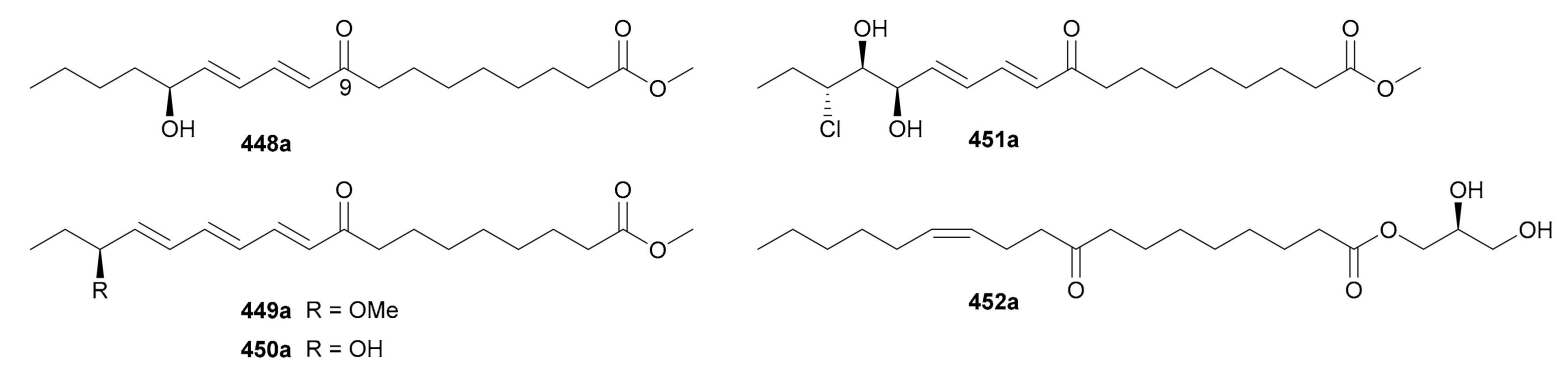
Structures of fatty acids.

Five pairs of enantiomeric fatty acid esters (**448a/448b−452a/452b**) were recorded in this covered stage and their structures are listed in Figure 27, with names being shown in Table S27 in Supplementary Materials. Usually, the generation of chirality in these compounds derives from the nonstereoselective oxidations on the aliphatic chain (**448−451**) or substitution on the glycerol moiety (**452**). Compounds **448a**/**448b**−**452a**/**452b** from *Plantago depressa* were characterized as four pairs of 9-oxo octadecanoid derivatives by the authors’ group, with **451** bearing a rare chlorine atom [6]. We have also isolated **452a**/**452b** as octadecanoid monoglycerides from the seeds of *Ipomoea nil* in 2019 and established their abs. configs. via an in situ dimolybdenum ECD method [9].

### 2.14. Miscellaneous

**Figure 28:**
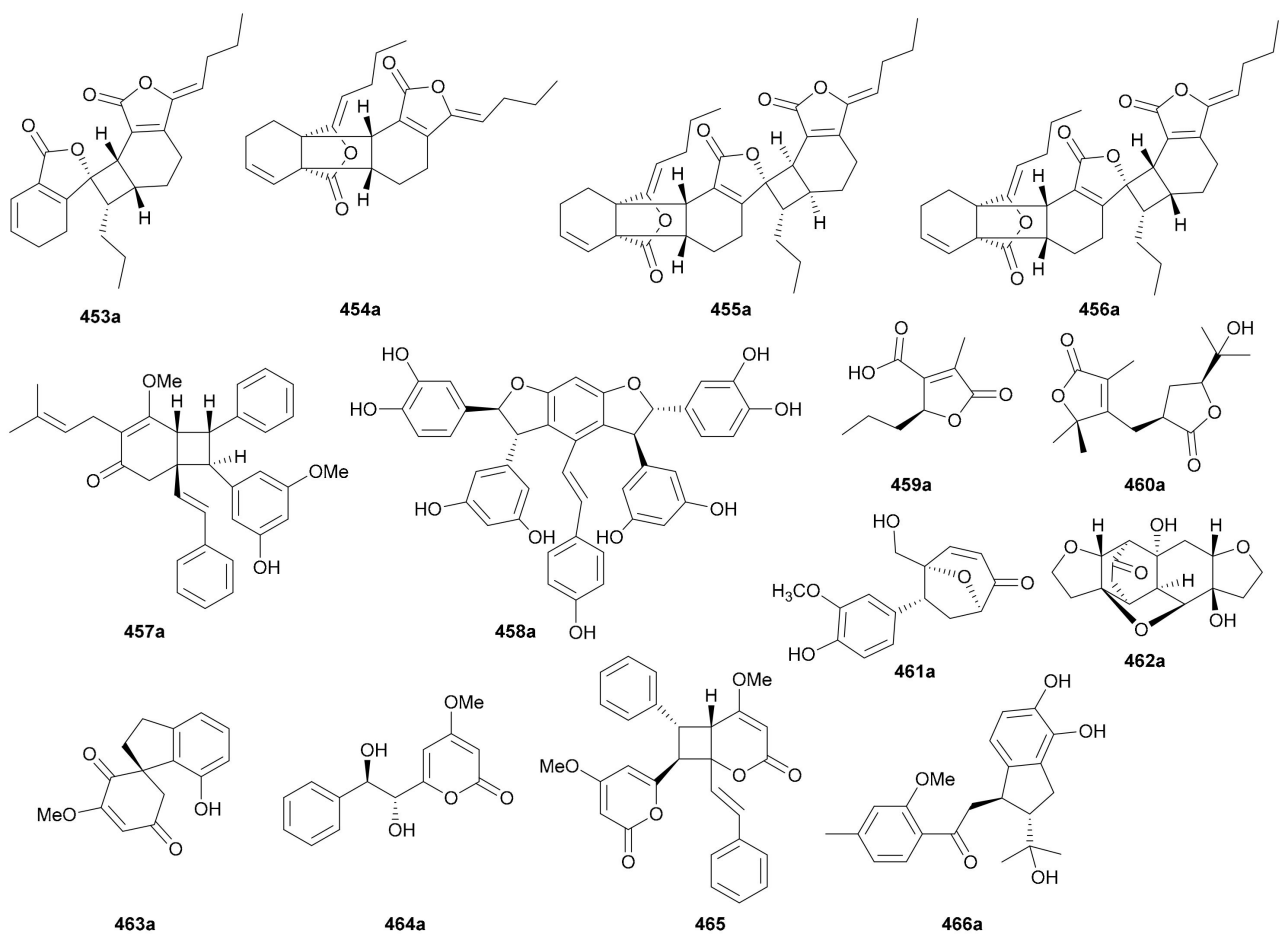
Structures of miscellaneous classes.

Other enantiomeric NPs from plants reported in this period are displayed in Figure 28 (names see Table S28). The abs. configs. of all these enantiomers were assigned by the TDDFT-ECD method unless otherwise specified.

Compounds **453**−**456** are four pairs of enantiomeric phthalide derivatives, all of which were isolated and characterized from *Angelica sinensis* in 2018 [211]. Phthalides are a rare class of NPs referring to lactones of 2-hydroxymethyl benzoic acids. They exist in nature as monomers or oligomers, and the latter are generally produced via [2 + 2] or [4 + 2] cycloaddition to form a number of complex polycyclic skeletons with multiple chiral centers [211]. Among them, **453** and **454** are dimers, while **455** and **456** are trimers.

**Scheme 7:**
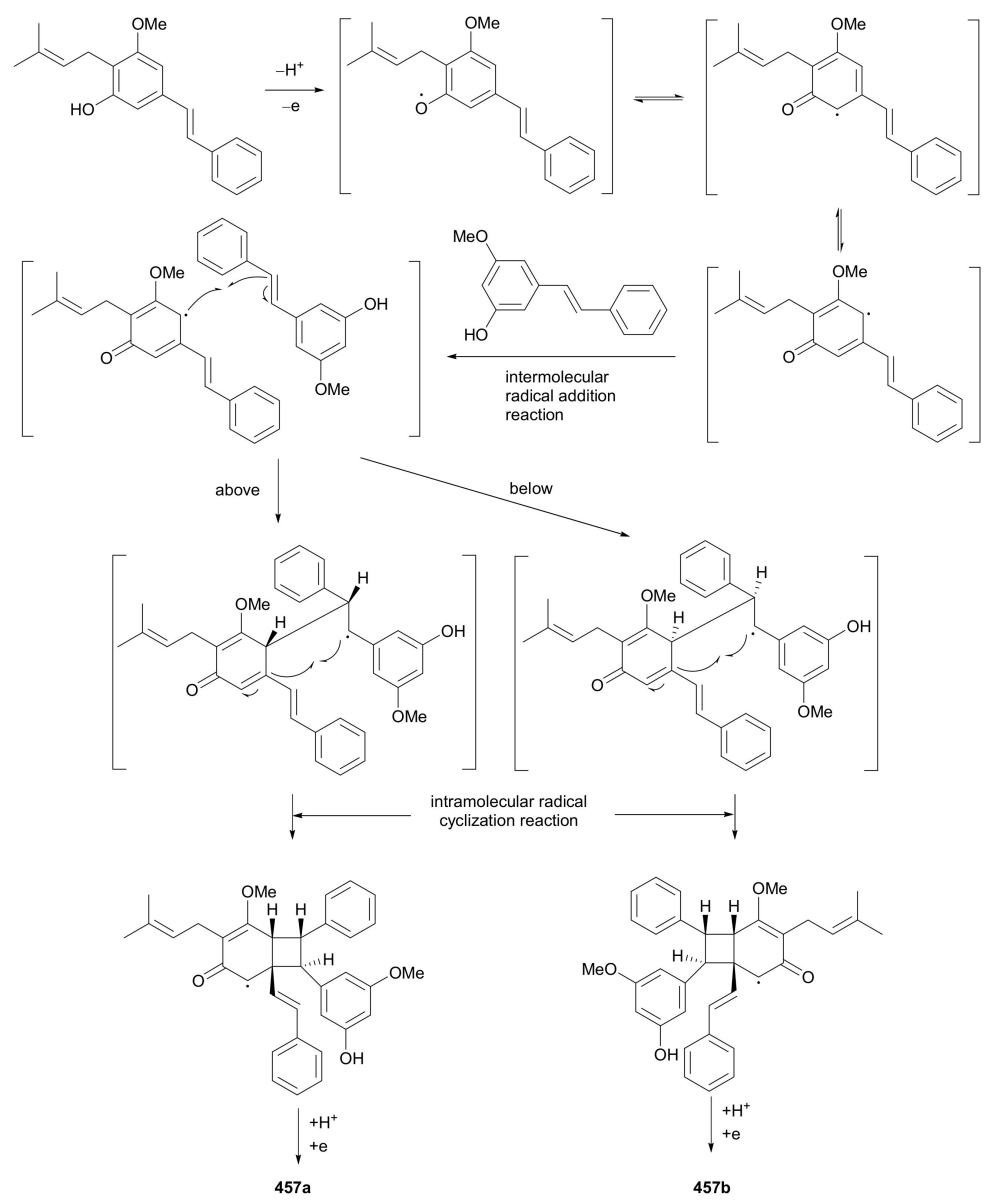
Plausible biosynthetic pathways for **457a**/**457b**.

Compounds **457a**/**457b** and **458a**/**458b** are enantiomeric stilbenoids that have 1,2-diphenylethylene (stilbene) as their basic scaffold and exist as monomers or oligomers in nature. They normally act as phytoalexins to assist plants in their resistance to pathogens or stress factors [212]. Compounds **457a**/**457b**, prenylated stilbenoid dimers isolated from *Cajanus cajan* in 2014, possess an interesting dimerization pattern generated from nonstereoselective radical addition as shown in Scheme 7, and their structures including the abs. configs. were determined by a combination of X-ray diffraction analysis and TDDFT-ECD calculation [213]. Compounds **458a**/**458b** are enantiomeric stilbenoid trimers obtained from *Cyperus rhizomes* in 2012, and their abs. configs. were established by comparing the [α]_D_ and ECD data with those of known analogues [214,215].

Compounds **459a**/**459b** are butenolide derivatives isolated from *Dendrobium nobile* in 2016 [49], while **460a**/**460b**, with an unprecedented skeleton incorporating both butyrolactone and butenolide moieties, were obtained from *Melicope viticina* in 2019 [202]. Compounds **461a**/**461b** featuring an oxabicyclo[3.2.1]octane ring were discovered from *Ligusticum chuanxiong* in 2019 [216], and **462a**/**462b** are a pair of enantiomeric cyclohexylethanoid dimers acquired from *Incarvillea younghusbandii* in 2012 [217]. Compounds **463a**/**463b** from *Dendrobium nobile* were identified as a pair of spirodiketone enantiomers in 2016 [218]. Styrylpyrone monomer (**464a**/**464b**) and dimer (**465**) enantiomers were reported from *Sanrafaelia ruffonammari* and *Ophrypetalum odoratum*, respectively, but the dimer **465** was only obtained as a racemate without further chiral separation [219]. Compounds **466a**/**466b** are a pair of enantiomeric 2,3-dihydro-1*H*-indene derivatives discovered from *Streblus indicus* in 2016 [220].

## 3. Enantiomers from Kingdom Fungi

Enantiomers originating from fungi, i.e., from phyla Ascomycota and Basidiomycota, will be presented in this section. The structural classification of NPs from fungi is not as regular and clear as those from plants; a myriad of fungal NPs belong to the super family of polyketides that derive biogenetically from the acetate pathway [1]. Also, considering the limited number of molecules described in this section, the enantiomers described here are simply divided into nonalkaloids and alkaloids. Where applicable, their biogenesis and structure will also be described.

### 3.1. Enantiomers from Phylum Ascomycota

#### 3.1.1. Nonalkaloids

**Figure 29:**
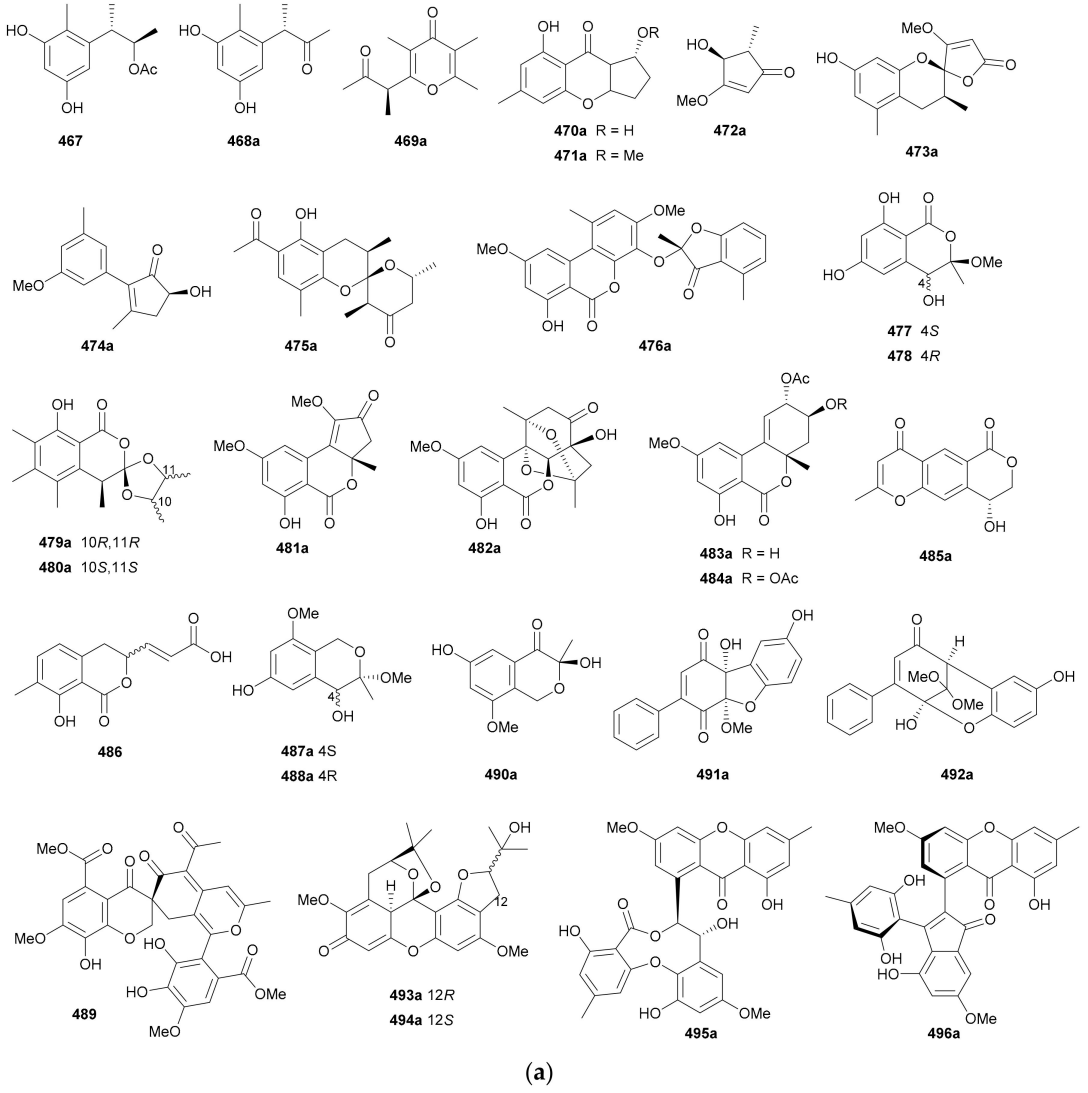

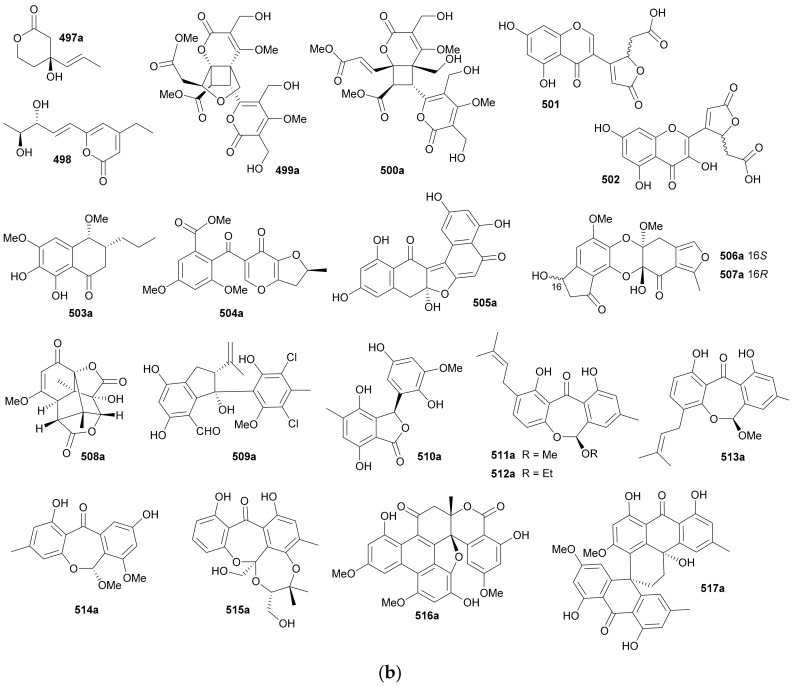
(**a**) Structures of nonalkaloids from phylum Ascomycota (Part 1). (**b**) Structures of nonalkaloids from phylum Ascomycota (Part 2).

Nonalkaloid enantiomers from phylum Ascomycota show great structural diversity and biological importance. The structures of those documented in the covered period are summarized in Figure 29a,b, and their names are presented in Table S29 in Supplementary Materials. The abs. configs. of those established by TDDFT-ECD method are not specifically mentioned in this section.

Zhang and co-authors discovered a racemic polyketide **467**, together with four pairs of analogue enantiomers **468a**/**468b**–**471a**/**471b**, from the starfish-symbiotic fungus *Penicillium* sp. GGF16-1-2 in 2019 [221]. The enantiomeric cyclopentenones **472a**/**472b** and spiro-butenolides **473a**/**473b** were isolated from *Aspergillus Sclerotiorum* in 2019 [222]. Gao and coworkers investigated three endolichenic fungal strains *Nigrospora sphaerica*, *Alternaria alternata* and *Phialophora* sp. in 2016 and obtained the same polyketide enantiomers **474a**/**474b**, whose abs. configs. were determined by modified Mosher’s method [223]. Compounds **475a**/**475b** incorporating a benzannulated 6,6-spiroketal skeleton were isolated from the mangrove-derived fungus *Penicillium dipodomyicola* HN4-3A. Compounds **476a**/**476b**, a pair of ketal enantiomers from *Paraconiothyrium sporulosum*, were assigned the abs. configs. by application of Snatzke’s chirality rule for cyclopentenones [224]. The isocoumarins **477** and **478** were reported as racemic mixtures from *Penicillium coffeae* MA-314 in 2019 [225]. Compounds **479a**/**479b** and **480a**/**480b** are spiro-orthoester enantiomers bearing a novel 1,4,6-trioxaspiro[4,5]decane-7-one unit from *Penicillium minioluteum*, and their rel. configs. were assigned by single-crystal X-ray diffraction analysis [226]. Compounds **481a**/**481b** were characterized as a pair of cyclopentaisochromenone enantiomers from *Alternaria* sp. TNXY-P-1 in 2018 [227]. Puno and coworkers discovered **482a**/**482b** as dibenzo-*α*-pyrones bearing a diepoxy-cage-like moiety from an Endophytic *Alternaria* sp. in 2019 and confirmed their rel. configs. by X-ray crystallography [228]. Also elucidated as dibenzo-*α*-pyrones, **483a**/**483b** and **484a**/**484b** were reported from the endophytic fungus *Alternaria alternate* in 2014 [229]. Compounds **485a**/**485b**, a pair of enantiomeric chromone derivatives from the marine-derived fungus *Taeniolella* sp. BCC31839, were established the abs. configs. by the modified Mosher’s method in 2019 [230]. Compound **486** was obtained as a racemate from *Periconia* sp. in 2015, without further chiral fractionation [231], while **487a**/**487b** and **488a**/**488b** were isolated from the endophytic fungus *Aspergillus Fumigatus* in 2018 [232]. Compound **489** was elucidated as a racemic mixture from the cordyceps-colonizing fungus *Fimetariella* sp. in 2012, and it incorporates a novel spiro[chroman-3,7′-isochromene]-4,6′(8′*H*)-dione skeleton [233]. Compounds **490a**/**490b** were characterized as a pair of enantiomeric isochromanes from an endophytic fungus *Aspergillus fumigatus* in 2019 [234]. Enantiomers **491a**/**491b** and **492a**/**492b** were identified as *p*-terphenyl derivatives from the endolichenic fungus *Floricola striata* [235], with the abs. configs. being determined by using the helicity rule for *α*,*β*-unsaturated ketone. Compounds **493a**/**493b** and **494a**/**494b**, as xanthene enantiomers with an unprecedented hexacyclic heterocylic backbone, were isolated from *Xylaria feejeensis* GM06 in 2018 [236]. The abs. config. assignment for **493a**/**493b** was based on the X-ray crystallographic experiment. Compounds **495a**/**495b** were characterized as a pair of dimeric polyketide enantiomers from a mangrove endophytic fungus *Ascomycota* sp. SK2YWS-L [237], with the absolute structures being determined by X-ray diffraction analysis and TDDFT-ECD calculation. Compounds **496a**/**496b** are a pair of 2,3-diaryl indone atropisomers isolated from *Ascomycota* sp. SK2YWS-L in 2018 [238].

**Scheme 8:**
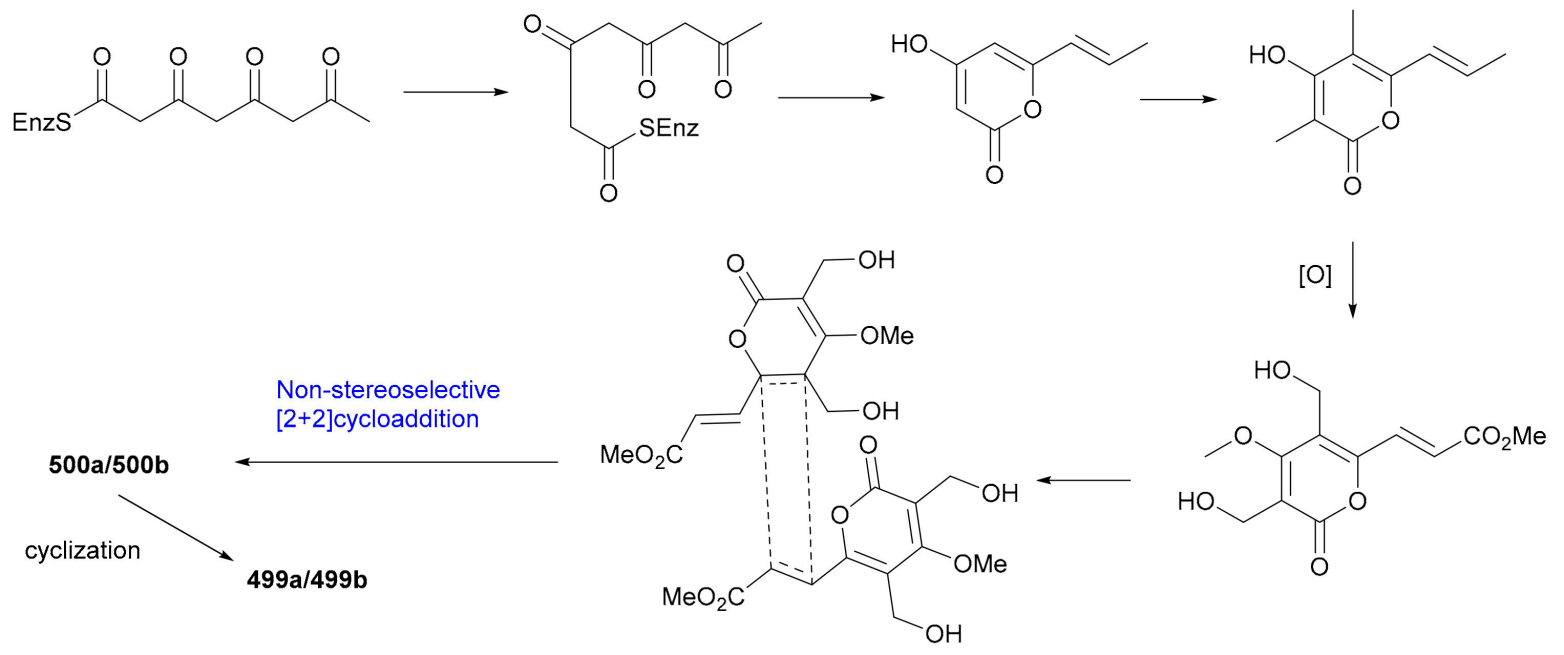
Plausible biosynthetic pathways for **499a/499b** and **500a/500b**.

Compounds **497a**/**497b** were identified as simple *δ*-lactone enantiomers from the fungus *Aspergillus terreus* in 2018 [239], while **498** is a α-pyrone derivative obtained as a nearly racemic mixture from the endolichenic fungus *Tolypocladium* sp. in 2017 [240]. Pei and coworkers discovered two pairs of dimeric α-pyrone enantiomers (**499a**/**499b** and **500a**/**500b**), which was formed via intermolecular nonstereoselective [2 + 2] cycloaddition reaction (Scheme 8), from the endophytic fungus *Phoma* sp. YN02-P-3 in 2017, and **499a**/**499b** possess a novel 6/4/5/6 tetracyclic ring system. Moreover, the rel. config. assignment for **500a**/**500b** was confirmed by single-crystal X-ray diffraction analysis [241]. Compounds **501** and **502** were elucidated as C-ring open flavonoids from *Pochonia chlamydosporia* var. *spinulospora* FKI-7537 in 2018, and **502** was successfully resolved into two enantiomers but without assigning the abs. configs., while **501** was not subjected to chiral separation due to limited amount [242]. Compounds **503a**/**503b** and **505a**/**505b** are two pairs of polyketides isolated from *Penicillium chrysogenum* MT-12 in 2017, where the racemic mixture of **505** had been previously reported from an endophytic fungus *Aspergillus* sp [243]. Compounds **504a**/**504b**, a pair of funicone enantiomers, were obtained from the mangrove sediment-derived fungus *Penicillium pinophilum* SCAU037 [244]. The polyketide dimers **506a**/**506b** and **507a**/**507b** bearing a rare pentacyclic dihydrobenzo[1,4]dioxine core were isolated from *Penicillium canescens* in 2019 [245]. Enantiomers **508a**/**508b**, a pair of caged norsesquiterpenoids with a novel tricyclo[4.4.0^1,6^.0^2,8^]decane carbon skeleton, were obtained from the endophytic fungus *Preussia isomera* in 2019, with the rel. config. being confirmed by X-ray diffraction data [246]. Kong and coworkers discovered **509a**/**509b**, featuring a prenylated chlorobenzophenone backbone, from the plant endophytic fungus *Pestalotiopsis* sp. in 2017 [247]. Compounds **510a**/**510b** are 2-benzofuran-1(3*H*)-one derivatives isolated from a mangrove-derived fungus *Eurotium rubrum* MA-150 in 2016 [248]. Compounds **511a**/**511b**−**513a**/**513b**, three prenylated dibenzo[*b*,*e*]oxepinone enantiomer pairs, were reported from a wetland soil-derived fungus *Talaromyces flavus* in 2016 [249], and the same type of enantiomers **514a**/**514b** were obtained from an endophytic fungus *Xylaria* sp. in 2015 [250]. The benzophenone-hemiterpene adducts **515a**/**515b** were separated from the endophytic fungus *Cytospora rhizophorae* in 2019 [251]. Compounds **516a**/**516b** are a pair of enantiomeric polyketides incorporating a 6/6/6/6/5/6/6 heptacyclic backbone and were isolated from fungus *Alternaria* sp. MG1 in 2019 [252]. Compounds **517a**/**517b** were identified as dimeric polyketide enantiomers from a marine-derived fungus *Eurotium* sp. SCSIO F452 in 2019 [253].

#### 3.1.2. Alkaloids

**Figure 30:**
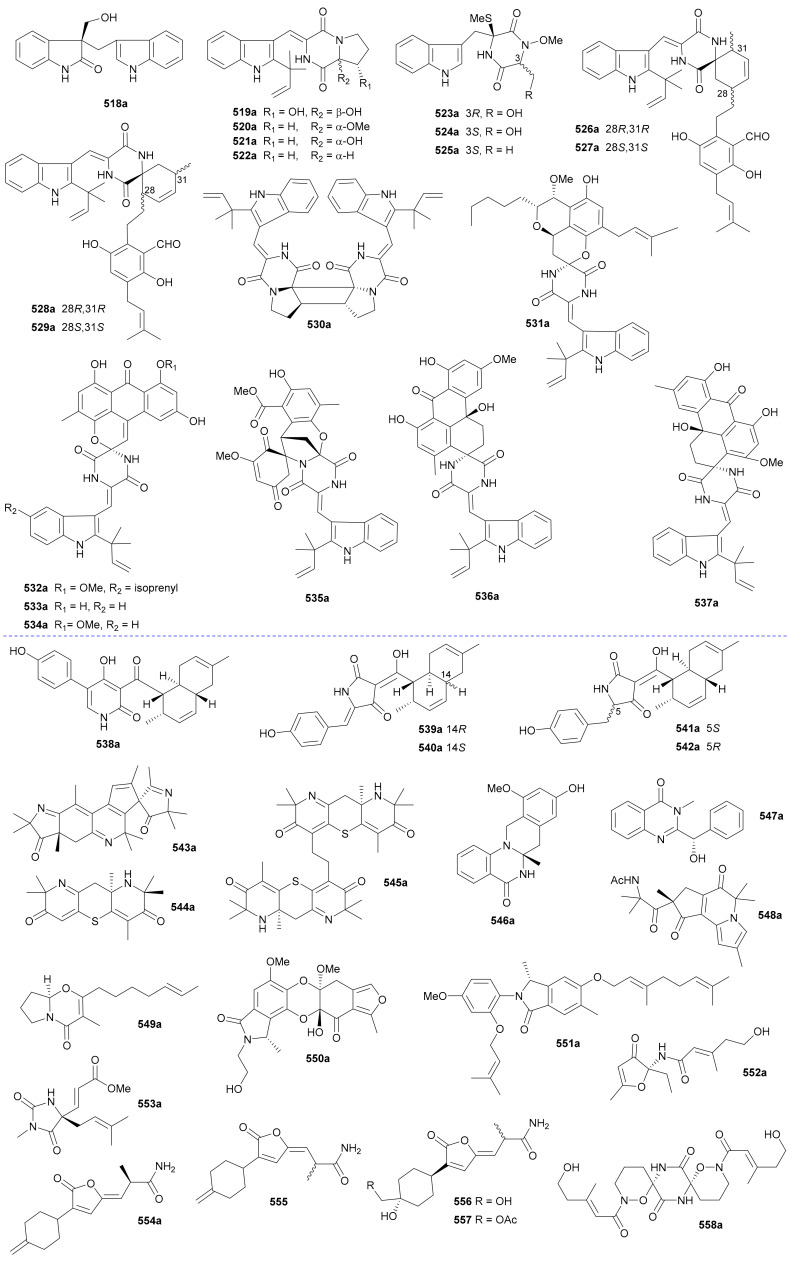
Structures of alkaloids from phylum Ascomycota.

Alkaloid enantiomers found in phylum Ascomycota during the reported period are displayed in Figure 30, with their names being shown in Table S30 in Supplementary Materials. The abs. configs. of those established by TDDFT-ECD method are not specifically mentioned in this section.

**Scheme 9:**
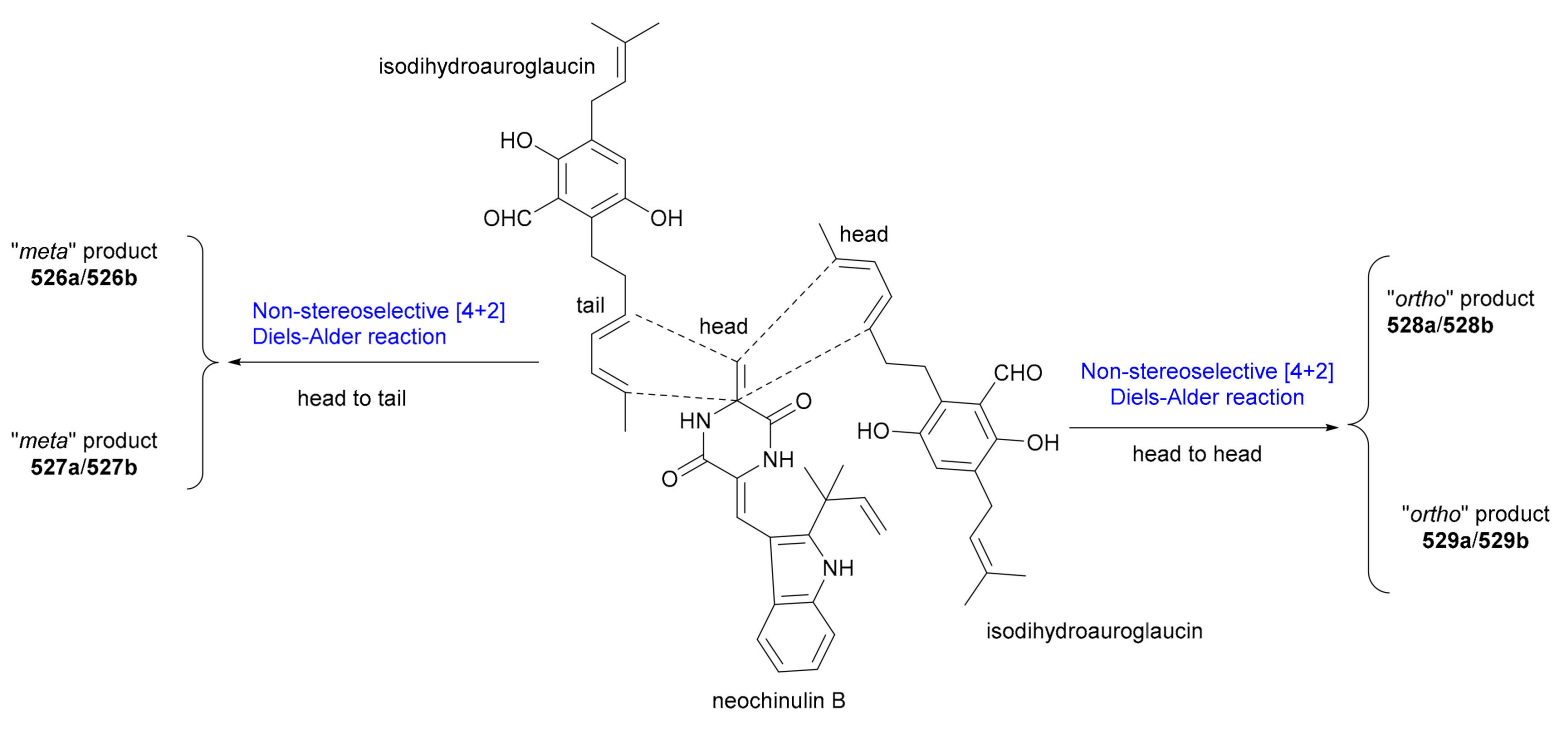
Plausible biosynthetic pathways for **526/526b−529a/529b**.

**Scheme 10:**
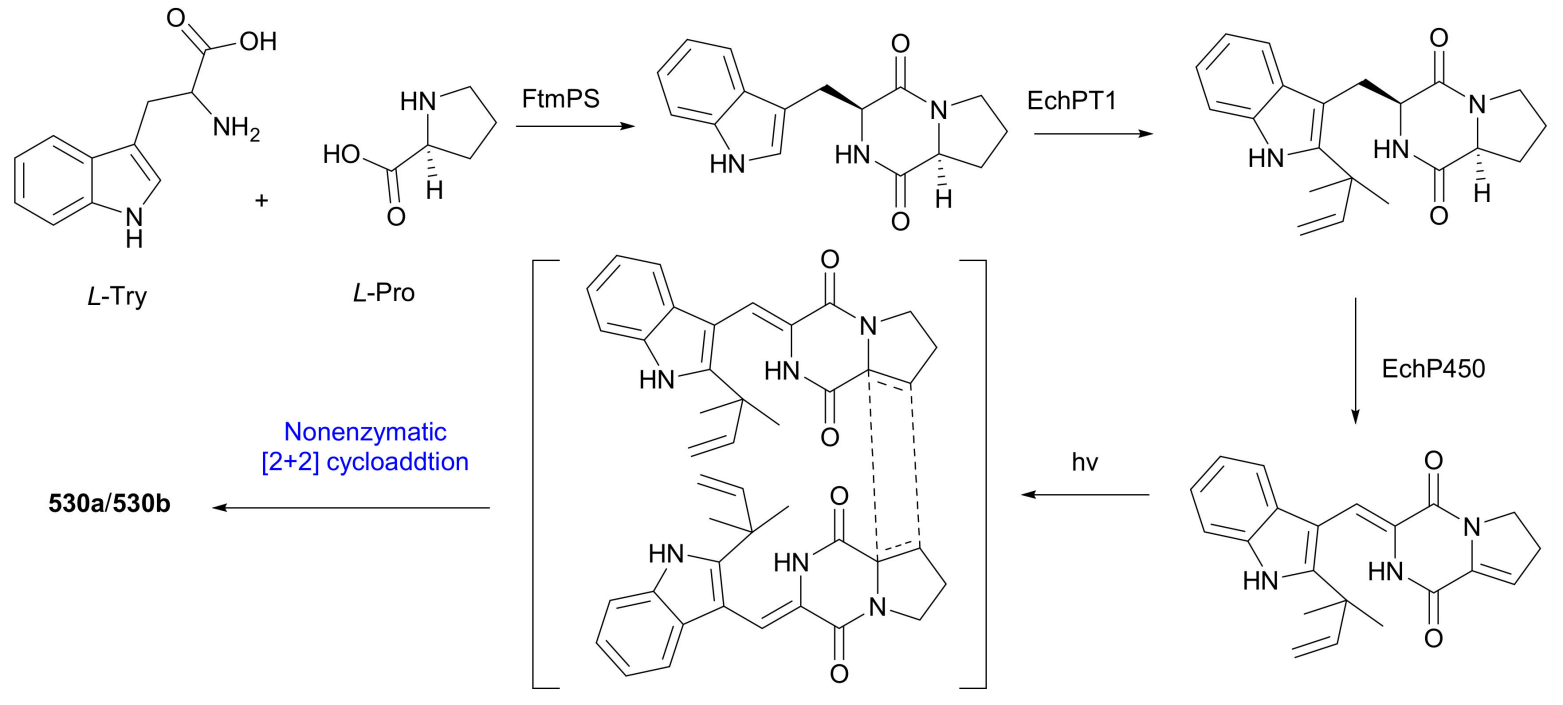
Plausible biosynthetic pathway for **530a/530b**.

**Scheme 11:**
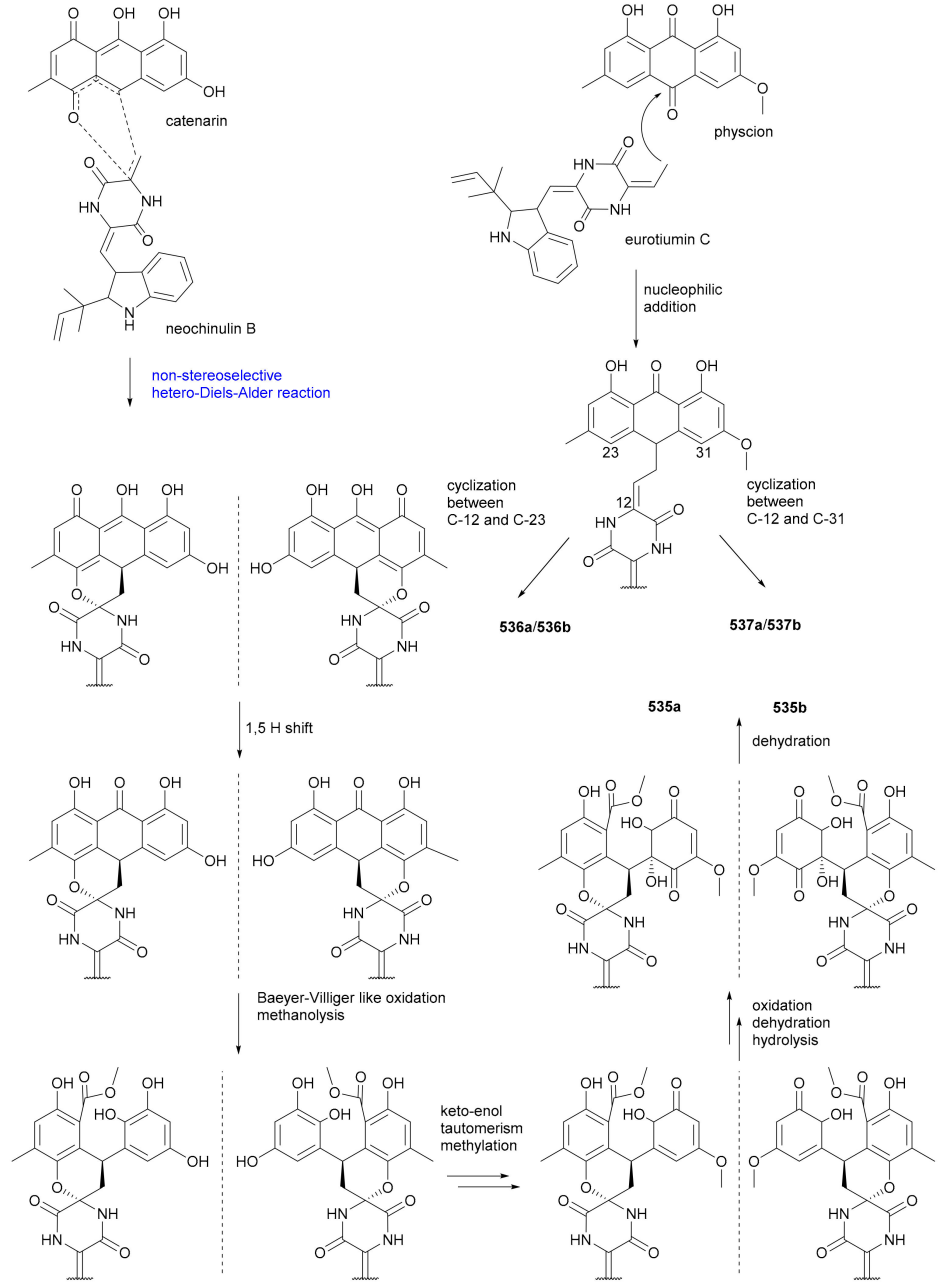
Plausible biosynthetic pathways for **535a**/**535b−537a**/**537b**.

Compounds **518**−**537** are a series of indole alkaloid derivatives reported from different fungal species. Except for the bisindole enantiomers **518a**/**518b** (from *Fusarium* sp. XBB-9), whose abs. configs. were assigned by X-ray diffraction analysis [254], all other alkaloids possess a cyclodipeptide scaffold (also known as diketopiperazine) formed from tryptophan and a second amino acid. Four pairs of enantiomers (**519a**/**519b**−**522a**/**522b**) biosynthesized from tryptophan and proline were isolated from the marine-derived species *Aspergillus versicolor* OUCMDZ-2738 in 2019 [255], while the tryptophan-alanine dipeptide enantiomers **523a**/**523b**−**525a**/**525b** bearing rare thiomethyl and *N*-methoxy groups were obtained from an alga-derived endophytic fungus *Acrostalagmus luteoalbus* TK-43 in 2019 [256]. Wang and coworkers discovered the spirocyclic alkaloids **526a**/**526b**−**529a**/**529b** from *Eurotium* sp. SCSIO F452 in 2019 and proposed their biosynthetic pathways as shown in Scheme 9 [257], while **529a**/**529b** had also been reported from *Aspergillus effuses* H1-1 in 2012 [258]. Compounds **530a**/**530b**, whose abs. configs. were determined by X-ray crystallography, incorporate a novel 6/5/4/5/6 pentacyclic motif and were acquired from the mangrove endophytic fungus *Aspergillus* sp. SK-28 in 2019. As shown in Scheme 10, the nonenzymatic catalyzed [2 + 2] cycloaddition could be the plausible key biosynthetic step to generate both enantiomers of **530** [259]. Alkaloids **531a**/**531b** with a 3′,3a′,5′,6′-tetrahydrospiro[piperazine-2,2′-pyrano[2,3,4-de]chromene] ring system were isolated from a mangrove rhizosphere soil derived fungus *Aspergillus effuses* H1-1 in 2012 [258], and compounds **532a**/**532b**−**534a**/**534b** as three pairs of variecolortide enantiomers were reported from the fungus *Eurotium* sp. [260]. Wang and coworkers disclosed three spirocyclic diketopiperazine enantiomer pairs (**535a**/**535b**−**537a**/**537b**) from the marine-derived fungus *Eurotium* sp. SCSIO F452 in 2019 and proposed their plausible biosynthetic pathways as shown in Scheme 11 [261]. Compounds **535a**/**535b** possess a highly functionalized *seco*-anthronopyranoid structural unit with a 2-oxa-7-azabicyclo[3.2.1]octane core, while **536a**/**536b** and **537a**/**537b** represent rare examples of diketopiperazines with a 6/6/6/6 tetracyclic cyclohexene-anthrone fragment.

The decalin-containing 4-hydroxy-2-pyridones (**538a**/**538b**) and their four pairs of rearranged analogues (**539a**/**539b**−**542a**/**542b**) were isolated from the solid culture of fungus *Coniochaeta cephalothecoides* in 2017 [262]. In 2019, Liu and colleagues investigated the metabolites of fungus *Xylaria longipes* to detect two highly conjugated alkaloids, **543a/543b** and **548a/548b**. The former possesses a 5/6/6/5/5 fused ring system with a unique 2-azaspiro[4.4]nonane substructure [263]. From the same fungal species, the same authors reported **544a**/**544b** and their dimers **545a**/**545b** [264] as thiopyranodipyridine enantiomers. Compounds **546a**/**546b** and **547a/547b** were identified as *N*,*N*′-ketal quinazolinone alkaloid enantiomers from an ascidian-derived fungus *Penicillium* sp. 4829 in 2019 [265] and from an algicolous *Talaromyces* sp. in 2016 [266], respectively. Compounds **549a**/**549b** are a pair of enantiomeric 4-oxabicyclo[4.3.0]lactam derivatives from the marine-derived fungus *Penicillium griseofulvum* reported in 2017 [267]. The aromatic polyketide enantiomers **550a**/**550b** with a 5/6/6/6/5 heterocyclic architecture were separated from *Penicillium canescens* in 2019 [245], while the enantiomeric phthalimidine derivatives **551a**/**551b** were acquired from the sponge-derived fungus *Stachylidium* sp. in 2012 [268]. Compounds **552a**/**552b** were characterized as a pair of *N*-furanone amide enantiomers from the solid culture of *Trichoderma atroviride* S361 in 2018 [269], and **553a**/**553b**, a pair of enantiomeric hydantoin (imidazolidin-2,4-dione) derivatives, were obtained from the fungus *Fusarium* sp. in 2015 [270]. Compounds **554−557** were isolated as bisabolane sesquiterpenoid amide racemates from the plant endophytic fungus *Paraconiothyrium brasiliense* in 2015, but only **554** was chirally separated into pure enantiomers [271]. Compounds **558a**/**558b** are a pair of enantiomeric alkaloid dimers with a symmetrical spiro[oxazinane-piperazinedione] skeleton from *Pestalotiopsis* sp. in 2015 [272].

### 3.2. Enantiomers from Phylum Basidiomycota

**Figure 31:**
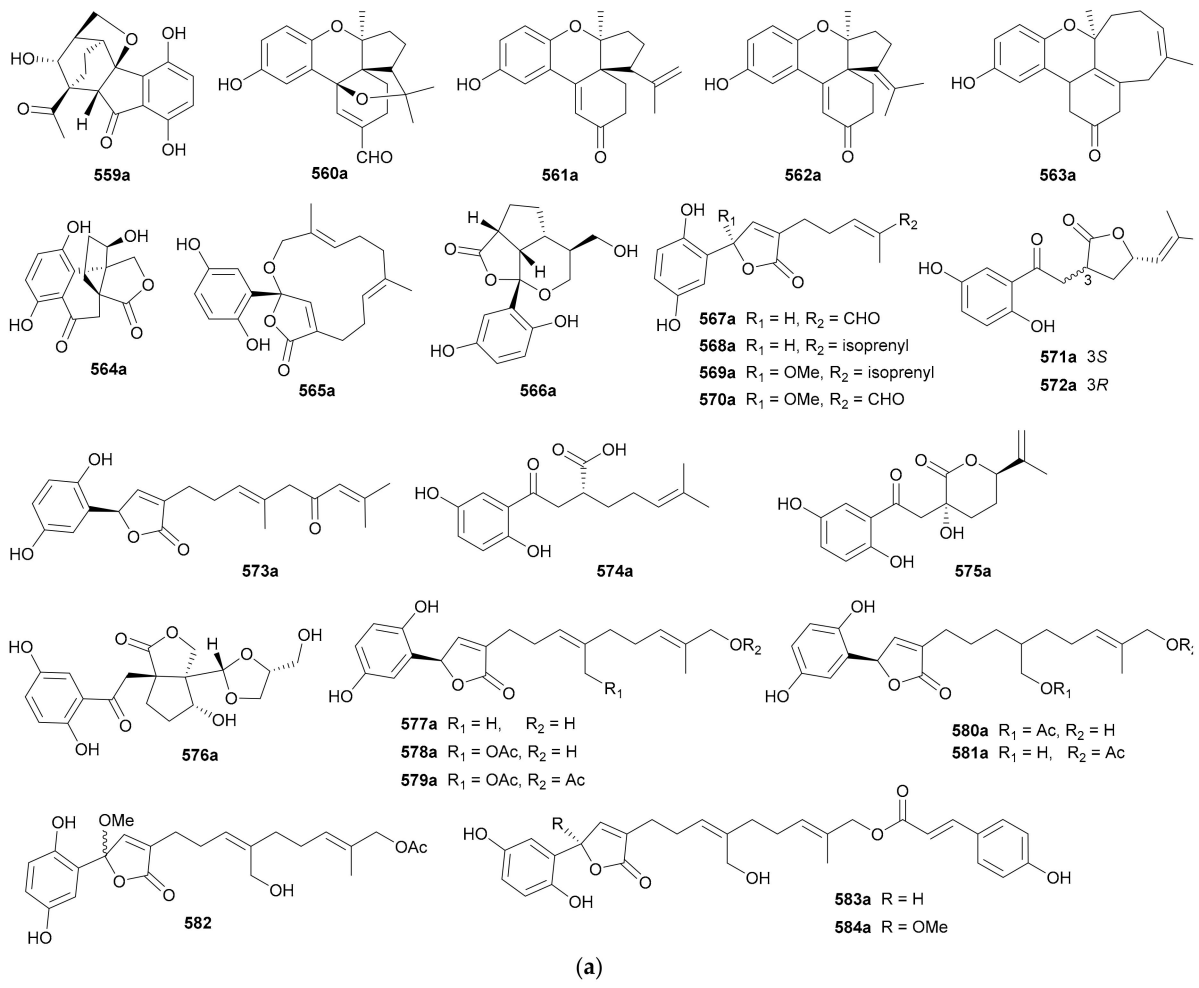

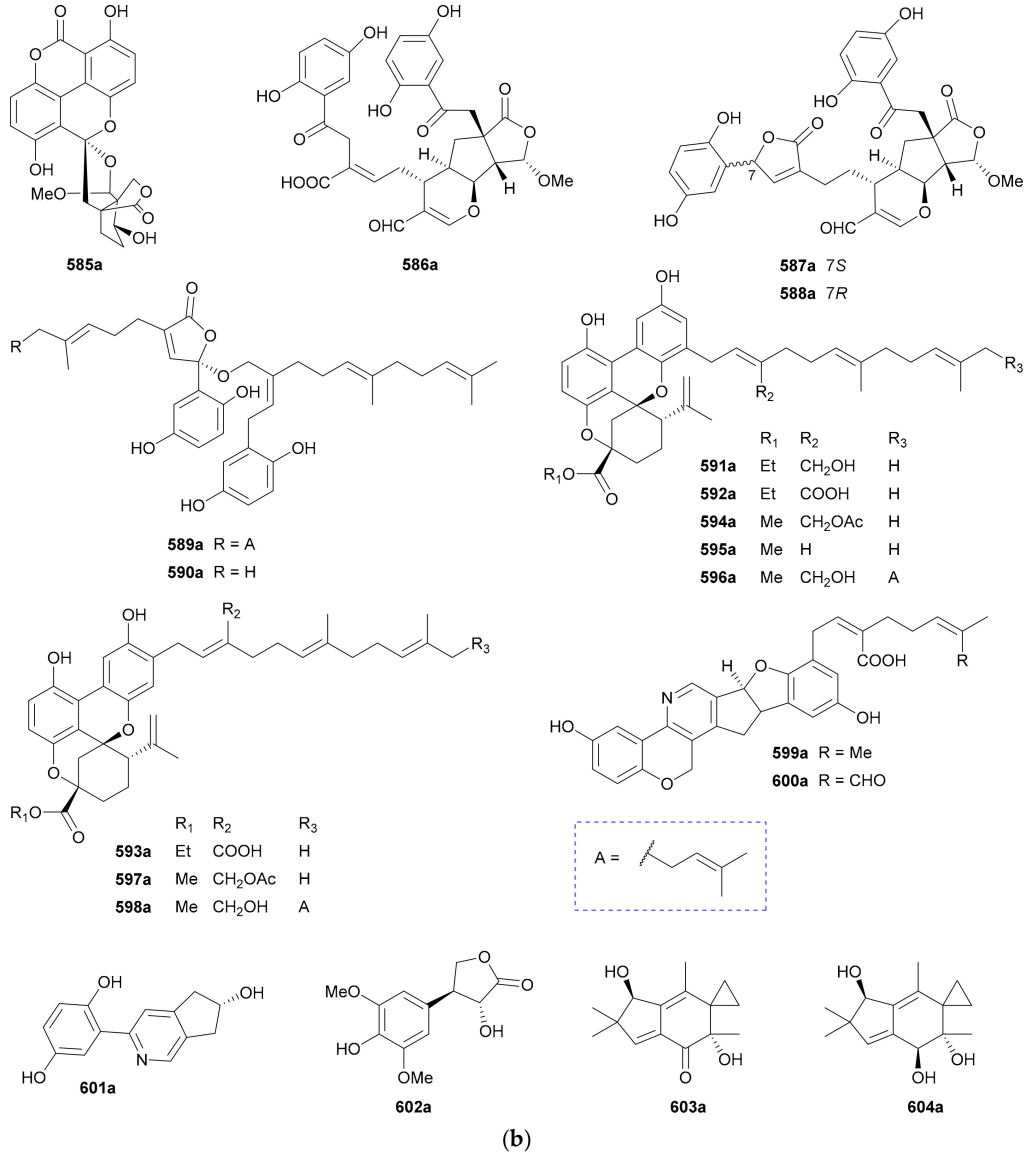
(**a**) Structures of metabolites from phylum Basidiomycota (Part 1); (**b**) Structures of metabolites from phylum Basidiomycota (Part 2).

It is interesting to note that all natural enantiomers from phylum Basidiomycota collected in this period, with only one exception (*Granulobasidium vellereum*), were reported from species of the well-known medicinal macrofungus genus *Ganoderma*. More interestingly, all the enantiomers from *Ganoderma* fungi, with one exception., are hydroquinone derivatives (**602**). In addition, the majority of these enantiomers belong to the meroterpenoid class (hydroquinone-terpenoid hybrid), and the terpenyl units here are usually monoterpenoid or sesquiterpenoid. Their structures and names are summarized in Figure 31a,b and Table S31, respectively. The abs. configs. of these enantiomers in this section have all been determined by TDDFT-ECD calculation unless otherwise specified.

Compounds **559**–**584** represent monomeric hydroquinone-terpenoid enantiomers. Cheng and coworkers discovered a pair of hydroquinone-trinorsesquiterpenoid enantiomers (**559a**/**559b**) possessing a fused 6/5/6/6/5 polycyclic skeleton from *G. lucidum* in 2019 [273]. Compounds **560a**/**560b−563a**/**563b**, identified as a series of hydroquinone-mononorsesquiterpenoid hybrids from *G. cochlear* in 2014, possess a spiro[4,5]decane ring system (**560−562**) and an eight-membered ring (**563**), with the abs. configs. being assigned by single-crystal X-ray diffraction analysis [274]. Compounds **564a**/**564b** from *G. lucidum* are a pair of rotary door-shaped hydroquinone-normonoterpenoid enantiomers with an unusual 5/5/6/6 ring system, and their abs. configs. were established by interpretation of X-ray crystallographic data [275]. Compounds **565a**/**565b**, a pair of macrocyclic meroterpenoid enantiomers derived from a hydroquinone and an intact sesquiterpenoid, were isolated from *G. resinaceum* by Chen et al. in 2017 [276]. The hydroquinone-monoterpenoid enantiomers **566a**/**566b** with an unusual dioxacyclopenta[*c,d*]inden motif were reported from *G. applanatum* in 2016 [277]. Nine pairs of enantiomers **567a**/**567b−575a**/**575b** incorporating either monoterpenoid or sesquiterpenoid fragments were obtained from *G. applanatum* in 2015 [278], and **570a**/**570b** was also reported from *G. lucidum* in the same year with the abs. configs. being not assigned [279]. Compounds **576a**/**576b** featuring an interesting polycyclic meroterpenoid skeleton with a glycerol unit were isolated from *G. applanatum* in 2017 [280]. Five pairs of hydroquinone-sesquiterpenoid enantiomers **577a**/**577b**−**581a**/**581b** and a racemate **582**, all bearing a butenolide fragment, were isolated from *G. sinense* in 2016 [281]. Compounds **583a**/**583b** and **584a**/**584b**, two pairs of farnesylated hydroquinone enantiomers incorporating a *p*-hydroxycinnamoyl residue, were discovered from *G. sinense* in 2016 [281].

Compounds **585**–**600** represent dimeric hydroquinone-terpenoid enantiomers. Enantiomers **585a**/**585b** were elucidated as hydroquinone dimers hybridized with a highly oxygenated monoterpenoid moiety from *G. applanatum* in 2016 [282]. They feature an unprecedented dioxaspirocyclic skeleton constructed from a 6/6/6/6 tetracyclic system and an unusual tricyclo[4.3.3.0^3′,7′^]dodecane unit, and their abs. configs. were determined by single-crystal X-ray diffraction analysis [282]. Also from *G. applanatum*, Cheng and coworkers separated three pairs of dimeric hydroquinone-monoterpenoid enantiomers **586a**/**586b−588a**/**588b** [283]. Two types of meroterpenoid heterodimer enantiomers (**589a**/**589b** & **590a**/**590b**; **591a**/**591b**–**593a/593b**) from *G. cochlear* were reported by the same authors from Cheng’s group, and their abs. configs. were assigned by comparing the ECD curves with those of reported analogues [284,285]. Five pairs of enantiomers (**594a**/**594b**−**598a**/**598b**) of the same type as **591**–**593**, along with the novel hybrid dimers (**599a**/**599b** & **600a**/**600b**) formed by a hydroquinone-pyridine and a hydroquinone-monoterpenoid, were also isolated and characterized from *G. cochlear* in 2015 [286].

Compounds **601a**/**601b** were identified as a pair of hydroquinone-pyridine alkaloid enantiomers from *G. luteomarginatum* in 2019 [287], while butyrolactone **602** from *G. lucidum* was chirally separated without assigning the abs. configs. of the enantiomers [279]. In 2015, sesquiterpenoids **603a** and **604a** [288] from the fungus *Granulobasidium vellereum* were identified as the enantiomers of illidin M (**603b**) [289] and dihydroilludin (**604b**) [290], respectively.

## 4. Enantiomers from Kingdom Prokaryota

**Figure 32:**
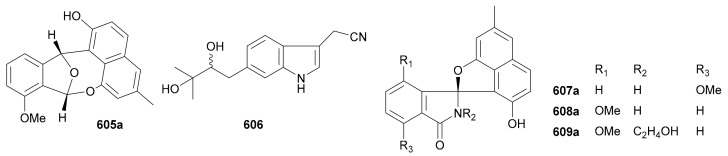
Structures of metabolites from actinomycetes.

Few enantiomers have been reported from the kingdom Prokaryota, i.e., only five pairs (**605**–**609**) to date, all of which were discovered from actinomycetes. Their structures and names are provided in Figure 32 and Table S32 in Supplementary Materials, respectively.

Compounds **605a**/**605b** are a pair of angucyclinone enantiomers featuring a unique epoxybenzo[*f*]naphtho[1,8-*bc*]oxocine heterocyclic scaffold, and were isolated from a *Streptomyces* sp. in 2019, with the abs. configs. being determined by X-ray diffraction analysis [291]. Compound **606**, a simple prenylated indole alkaloid bearing a rare cyano group, was isolated as a racemate without further chiral separation from *Streptomyces* sp. ZZ820 in 2019 [292]. Compounds **607a**/**607b**−**609a**/**609b** are three pairs of enantiomeric indole alkaloids with a spiro indolinone-naphthofuran skeleton reported from a *Streptomyces* sp. in 2017 [293].

## 5. Enantiomers from Kingdom Animalia

Compared with those from plants and microorganisms, compounds from animals only account for a small proportion of the large NP family, and have been mainly reported from lower animals such as sponges and corals. Therefore, the number of enantiomers from kingdom Animalia is also limited. According to their biological source, animal-derived enantiomers will be divided into the following three subcategories.

### 5.1. Enantiomers from Phylum Porifera

**Figure 33:**
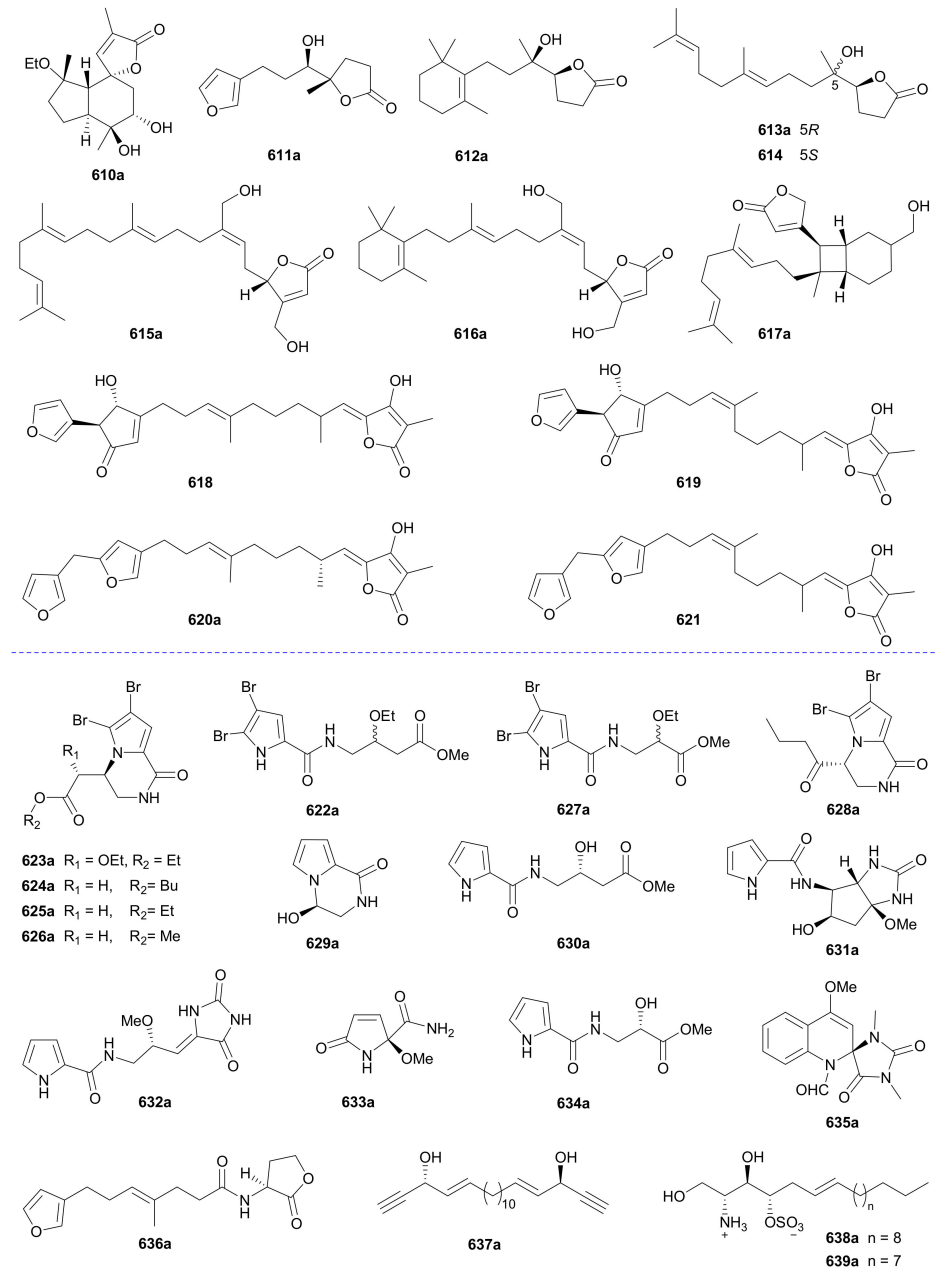
Structures of metabolites from phylum Porifera.

Animals from phylum Porifera (also termed Spongia) are generally known as sponges. They also represent a very important source of bioactive NPs. Natural enantiomers from sponges mainly include terpenoids and alkaloids; see Figure 33 and Table S34.

#### 5.1.1. Terpenoids

Compounds **610a**/**610b** were identified as a pair of valerenane-type sesquiterpene enantiomers from a *Spongia* sp. in 2019 [294], while the trinorsesquiterpenoid enantiomers **611a**/**611b** incorporating furan and butenolide rings were isolated from the Beihai sponge *Spongia officinalis* in 2018, with the abs. configs. being determined by biomimetic total synthesis and modified Mosher’s method [295]. The three C_17_ norditerpenoid pairs **612**−**614** with a *γ*-lactone unit, together with two pairs of sesterterpenoids **615** and **616** with a butenolide unit, were obtained from a *Cacospongia* sp. in 2019. Among them, all enantiomeric pairs except **614** were successfully separated. Compounds **617a**/**617b** are a pair of sesterterpenoid enantiomers featuring a bicyclo[4.2.0]octene core and were isolated from *Hippospongia lachne* in 2017 [296]. Compounds **618**−**621** are four pairs of furanosesterterpene tetronic acids from a *Psammocinia* sp., and **618** and **619** were found to be geometrical isomers of two pairs of enantiomers as revealed by chiral HPLC analysis. Similar to the case of **618** and **619**, compounds **620** and **621** were also proved to be two enantiomeric pairs, but only **620** was finally separated into pure enantiomers [297].

#### 5.1.2. Alkaloids

Interestingly, alkaloid enantiomers from sponges were discovered from species collected in South China Sea, with most of them belonging to the pyrrole alkaloid family, incorporating a pyrrole-2-carboxylic acid residue. Compounds **622a**/**623b−628a**/**628b** were characterized as a panel of bromopyrrole enantiomers from an *Agelas* sp. in 2016 by Zhu et al. [298]. Except **622a**/**622b** and **627a**/**627b**, the abs. configs. of the others were assigned by one of the three following methods including TDDFT-ECD calculation, ECD exciton chirality method and ECD comparison with known analogues [298]. Pyrrole alkaloids **629a**/**629b−631a**/**631b** are three pairs of enantiomers obtained from *Agelas aff. Nemoechinata* in 2017, and **631a**/**631b** possess an interesting cyclopentane-fused imidazole ring system [299]. Compounds **632a**/**632b−634a**/**634b** are also pyrrole alkaloid enantiomer pairs obtained from *Agelas nakamurai* in 2017 [300]. Alkaloids **635a**/**635b** featuring an unusual spiro bisheterocyclic quinoline-imidazole backbone were reported from *Fascaplysinopsis reticulate* in 2015 [301], while **636a**/**636b** represent a pair of trinorsesquiterpenoid amide enantiomers isolated from the Beihai sponge *Spongia officinalis* in 2018 [295].

#### 5.1.3. Lipids

Compounds **637a**/**637b**, a pair of interesting C_20_ bisacetylenic lipid enantiomers, were discovered from the marine sponge *Callyspongia* sp. in 2013, with the abs. configs. being determined by modified Mother’s method [302]. The lipid zwitterions **638a** and **639a** were separated from *Spirastrella abata* in 2012 [303], and their respective enantiomers (**638b** and **639b**) had been previously reported from the same species in 2002 [304].

### 5.2. Enantiomers from Phylum Arthropoda

**Figure 34:**
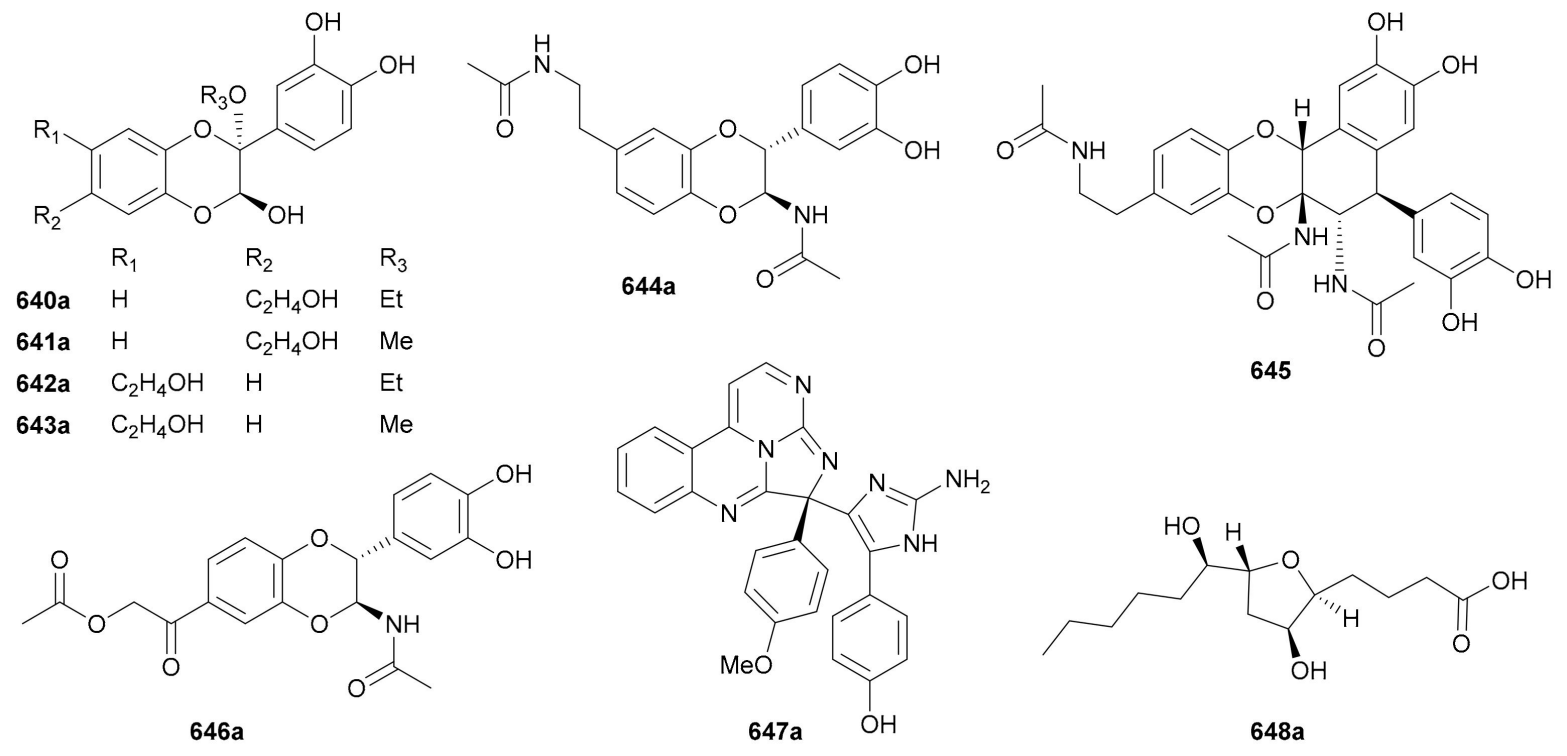
Structures of metabolites from phyla Arthropoda and Chordata.

Compounds **640a**/**640b−643a**/**643b** (Figure 34, names see Table S34 in Supplementary Materials) bearing a 2,3-dihydrobenzo[*b*][1,4]dioxin fragment were separated from the insect *Blaps japanensis* in 2015 [305], with the abs. config. of **640a** being determined by X-ray crystallographic analysis. Compounds **644a**/**644b** and **645** were characterized as *N*-acetyldopamine dimer and trimer, respectively, from the insect *Aspongopus chinensis* in 2014, and **645** possesses a novel tetrahydrobenzo[*a*]dibenzo[*b,e*][1,4]dioxine moiety and occurs as a racemate [306]. Compounds **646a**/**646b** are a pair of dimeric *N*-acetyldopamine enantiomers obtained from the insect *Polyphaga plancyi* in 2016 [307].

### 5.3. Enantiomers from Phylum Chordata

There have been only two pairs of enantiomers reported from the animals of phylum Chordata (see Figure 34 and Table S34 in Supplementary Materials). Compounds **647a**/**647b**, a highly nitrogenated enantiomer pair with a novel heterocyclic scaffold incorporating two extra phenol units, were isolated from a marine ascidian *Eudistoma* sp. in 2016 [308]. A pair of oxygenated myristic acid enantiomers bearing a tetrahydrofuran moiety (**648a**/**648b**) was obtained from a larval sea lamprey *Petromyzon marinus* in 2015, with the abs. configs. being determined by modified Mosher’s method [309].

## 6. Biological Properties

As is well known, NPs, on one hand, play a decisive role in maintaining their source organisms’ health, helping defend against internal or external adverse stresses and enticing favorable stimuli. On the other hand, NPs in the form of herbal medicines have long been used by humans as therapeutic agents against various diseases, thus guaranteeing the continuation of human civilization. With the advances of science and technology, NPs and their derivatives still shine in the research field of modern drug discovery and development [2].

It is widely accepted that chirality, as an important feature of most NPs, is closely related with their bioactivities. Normally, life systems tend to produce/utilize only one molecule of an enantiomeric pair. For example, humans only take in d-glucose and l-amino acids as nutrients. The fact that a pair of enantiomers can exert utterly different bioactivities was recognized as far back as the 1960s, when the ‘Phocomelia infants event’ caused by the (*S*)-enantiomer of the synthetic drug thalidomide taught the pharmaceutical industry an important lesson. For many years, however, NP workers failed to recognize the widespread occurrence of enantiomerism in nature, and failed to explore the differences in bioactivity between pairs of enantiomers. Fortunately, as data on natural enantiomers increase in scope, more and more biological properties of different classes of enantiomeric pairs have also been reported, and this has provided more examples with which to investigate the differences in bioactivity among enantiomers.

As bioassay protocols vary in different research labs and even in different batches from the same lab, it should be clarified that we do not intend to invite direct comparisons regarding the activity potency by tabulating the assay data from different reports. Instead, bioactivity comparisons between different labs will be completely avoided in the current review and the use of potency descriptors will also be kept to a minimum. Meanwhile, we will not list the biological data of all reported enantiomers, and only selective cases with obvious activity differences at the enantiomeric level are discussed, under the following subcategories: cytotoxic, antiviral, antibacterial, antifungal, anti-inflammatory, antioxidative, cell protective, enzyme inhibitory, β-amyloid (Aβ) aggregation inhibitory and miscellaneous activities.

### 6.1. Cytotoxicity

Cytotoxic evaluations of chemical entities were likely the most important primary strategy in the past in the search for potential chemotherapies for cancers, and remain among the most popular bioassays for NPs. The cytotoxic activities of selective enantiomeric pairs against a series of human tumor cell lines are summarized in Table 2.

The antiproliferative activities of the alkaloid racemates (±)-**171** and (±)-**172**, along with their respective enantiomers, against human HL-60 tumor cells were assessed in Hua’s lab. While the levoisomers (**171b** and **172b**) showed slightly better inhibitory activity than their respective dextroisomers (**171a** and **172a**), the racemic mixtures exhibited more potency than both enantiomers, indicating a likely synergistic effect [105]. The flavane enantiomers **268a**/**268b** were reported to show cytotoxicity against human Hep3B cells, and the dextroisomer **268b** was obviously more active than the levoisomer **268a** [146]. The xanthones 2**97a**/**297b** were able to inhibit the proliferation of human HL-60 and MDA-MB-231 cancer cells, and the (−)-enantiomer **297b** showed much stronger inhibitory activity than its (+)-enantiomer **297a** against MDA-MB-231 cells [156]. The levorotatory enantiomer **312b** was found to inhibit the proliferation of colorectal HCT-116 cell line with an IC_50_ of 14.23 μM, but its antipodal enantiomer **312a** was considered to be inactive [160].

The bisabolene-derived sesquiterpenoids **314a**/**314b** were tested in vitro for their cytotoxicities against five human tumor cell lines (HCT-116, HepG2, BGC-823, NIC-H1650 and A2780), and the (−)-enantiomer **314a** exerted significant inhibition against all tested cell lines with IC_50_ values in the range of 1.38−8.19 μM, while the (+)-enantiomer **314b** was considered inactive (IC_50_ > 10 μM) [162]. In contrast, the (+)-enantiomer **381a**, an acylphloroglucinol derivative, showed cytotoxic activities against the tested tumor cell lines (HL-60, SMMC-7721, A549, MCF-7 and SW480) with IC_50_ values in the range of 3.42−7.22 μM, while its (−)-enantiomer **381b** was taken inactive (IC_50_ > 20 μM) [191]. The same research group that reported **381a**/**381b** also screened the cytotoxicities of both racemates and pure enantiomers of acylphloroglucinols **382a**/**382b−385a**/**385b** against the aforementioned tumor cell lines [192], and as a result, the levorotary series exhibited higher potency than both the dextrorotary series and the racemates for most cells [192]. Of particularly note, the racemate (±)-**383** showed apparently decreased activity compared with its both enantiomers against all cell lines especially toward HL-60 cells (5.7- and 7.7-fold decrements), indicative of an antagonistic action between the two enantiomers, and similar effects were also observed for **384a**/**384b** and **385a**/**385b** on selective cell lines [192].

The methyl 2-naphthoate enantiomers **390a**/**390b** were found to show inhibition against the proliferation of three types of cancer cells (A549, MCF-7 and MDA-MB-231), with the levoisomer **390a** being ca. three times more active than the dextroisomer **390b** [8]. In addition, only the dextrorotary enantiomers of the spirocyclic diketopiperazines **536** and **537** showed growth inhibition against SF-268 and HepG2 tumor cell lines [261], but their corresponding levoisomers were inactive (>100 μM).

### 6.2. Antiviral

The antiviral activities of the enantiomers described in this review are shown in Table 3. The coumarins **254a**/**254b** did not show significant inhibition differences against either the herpes simplex virus 1 (HSV-1) or the host cell between enantiomers, but their racemate (±)-**254** exhibited obviously increased activity which was suggestive of a strong synergistic action [66]. Similar synergistic effects of enantiomers were also observed for another two pairs of coumarins, **104a**/**104b** and **105a**/**105b**, with the racemates (±)-**104** and (±)-**105** displaying 3.2- to 6.1-fold antiviral activity against the influenza virus A (H3N2) compared with the pure enantiomers [67]. Four pairs of phloroglucinol enantiomers **368a**/**368b−371a**/**371b** were subjected to antiviral assay against Kaposi’s sarcoma-associated herpes virus (KSHV); they all showed certain degrees of bioactivity differences at the enantiomeric level [184]. The fungus-derived alkaloid enantiomers **558a**/**558b** and the racemate (±)-**558** all exhibited antiviral activity against EV71 virus, with the dextrorotary enantiomer being nearly five times as active as its antipodal isomer [272].

### 6.3. Antibacterial

It appears that most of the antibacterial enantiomeric pairs collected in this review showed remarkably differentiable activities between enantiomers. Nonetheless, a few exceptions were still found and are listed in Table 4. The furoquinoline alkaloid enantiomers **147a**/**147b** were reported to have antibacterial activity against *Enterococcus faecalis*, and the (–)-enantiomer showed about two-fold activity as the (+)-enantiomer [97]. The *p*-hydroxycinnamoylated dihydrochalcone enantiomers **285a**/**285b**−**288a**/**288b** exhibited in vitro antibacterial activity against *Staphylococcus aureus* with IC_50_ values ranging from 0.61 to 6.0 μM [153], and it appeared that all the dextrorotary enantiomers were more effective than their respective levorotary isomers, with **288a**/**288b** showing the greatest activity difference, i.e., 3.7 fold [153].

### 6.4. Antifungal

Few reports have been published on the antifungal activities of the enantiomers mentioned in this review, although a handful of examples have shown about two-fold bioactivity differences between enantiomers (Table 5). The *δ*-lactone enantiomer (−)-**464a** was reported to display inhibitory activity against *Candida albicans* with an MIC of 26.4 μM, while its antipodal enantiomer (+)-**464b** was considered inactive [219]. In addition, both levorotary enantiomers of compounds (±)-**483** and (±)-**484** exhibited better antifungal activity again *C. albicans* than their respective dextrorotary isomers [229], and similar effect against *Fusarium solani* was also recorded for the indole-piperidine enantiomer pair (±)-**524** [256].

### 6.5. Anti-Inflammation

The anti-inflammatory activities of NPs have often been evaluated by testing their inhibitory capability against NO release in LPS-induced BV-2 microglial cells or RAW 264.7 macrophages (Table 6). The benzofuran-type lignan enantiomers **43a**/**43b** and **44a**/**44b** were tested for their NO production inhibitory effect in LPS-induced BV-2 microglial cells, with (−)-**43b** and (+)-**44a** exhibiting pronounced activity with IC_50_ values of 8.9 and 5.9 μM, being nearly twice as active as their respective antipodal enantiomers [39]. The levorotary spirodienone lignan enantiomers (−)-**82b** and (−)-**83b** showed significant inhibition against NO production in LPS-induced RAW 264.7 macrophages, with both being >3 fold as active as their respective dextrorotary enantiomers [54]. In the same bioassay model, the indolizidine dextroisomer **221a** displayed much stronger inhibitory activity (6.3 fold) than the levoisomer **221b** [124,128,164]. In contrast, the levorotary enantiomer **407b** was much more active (*ca.* 5 fold) than its antipodal enantiomer **407a** in the LPS-induced NO release assay in BV-2 cells [130,132,226,265].

### 6.6. Antioxidation

The DPPH and ABTS radical scavenging assay models have been widely used to evaluate the antioxidative capacity of NPs, although not many of the listed enantiomers in this review have been tested with these bioassays. Owing to their radical mechanism, most tested enantiomers displayed equal potency in both assays as expected, whereas the tryptophan-alanine dipeptide enantiomers **527a**/**527b**–**529a**/**529b** showed some activity differences at the enantiomeric level, particularly for dextroisomers **527a** and **528a**, that showed obviously enhanced radical scavenging activity (4.1 and 2.5 fold, respectively) compared with their levoisomers in the DPPH assay model (Table 7) [257].

### 6.7. Cell Protection

Cell protection assays are usually performed in neuronal cells to explore new chemicals that could be developed for the treatment of neurodegenerative disorders, but which could likely also be used in the search for molecules with which to treat other diseases (Table 8). Generally speaking, a >10% cell viability difference can be considered significant. The protective activity of neolignan enantiomers **24a**/**24b** against H_2_O_2_-induced cell injury in human neuroblastoma SHSY5Y cells was tested and the (+)-enantiomer showed obviously better activity than the (−)-enantiomer [20]. In a same assay model by Zhou et al., the analogous enantiomeric pair **72a**/**72b** also exhibited a similar trend of bioactivity difference, with the dextroisomer displaying better protective effect than the reference drug and the levoisomer being found to be inactive [46]. Further investigations revealed that (+)-**72a** could significantly decrease the percentages of both early and late apoptotic cells. The phenylpropanoid dextrorotary enantiomer **119a** presented much better neuroprotective activity than its levorotary enantiomer in the H_2_O_2_-treated SH-SY5Y cell injury assay, with nearly 20% cell viability increment [75]. Further studies demonstrated that (+)-**119a** could selectively inhibit the apoptosis induction and reactive oxygen species (ROS) accumulation by enhancing the activity of catalase (CAT). Compared with their respective antipodal enantiomers, indole alkaloids (−)-**129b** and (+)-**130a** also increased the cell viability by about 20% in an OKA-induced PC12 cell damage assay [83]. The isoquinoline alkaloids (−)-**165a**, (−)-**166a** and (+)-**173b** exhibited slightly better protective effects (51%−55% cell viability) than the positive control on hypoxic H9C2 cells, while (+)-**166b** were less active (45% cell viability) and (+)-**165b** and (−)-**173a** were considered inactive [103]. Two pairs of acetophenone enantiomers **423a**/**423b** and **426a**/**426b** exerted excellent protection on human vein endothelial cells (HUVEC) against extreme glucose-induced oxidative stress at 1 μM [200], with both dextrorotary enantiomers being much more active than their levorotary counterparts and showing complete cell protection. The (+)-enantiomer of diarylheptanoids (±)-**433** significantly increased the cell viability of cortical neurons compared with the control group (MPP^+^ treatment alone), while its (−)-enantiomer was inactive [206].

### 6.8. Enzyme Inhibition

A number of diseases are caused by the dysfunction of enzymes, so the discovery of enzyme inhibitors is one the most important tasks of the study of NPs. The enantiomers in this review have been shown to exert inhibitory activities against many enzymes including phosphodiesterase-9A (PDE9A), acetylcholinesterase (AChE), butyrylcholinesterase (BChE), *α*-glucosidase, tyrosinase, protein tyrosine phosphatase 1B (PTP1B), serine protease HLE, isocitrate lyase deubiquitinating enzyme USP7, isocitrate lyase, Na^+^/K^+^-ATPase and cyclooxygenase 2 (COX-2). Selective enantiomeric pairs with activity differences between enantiomers are listed in Table 9.

Isoquinoline enantiomers **157a**/**157b** were evaluated for their anti-AChE activity, with the (−)-enantiomer being >3.5 fold more active than the (+)-enantiomer [100]. In the same assay from another lab, the dextrorotary indole-diketopiperazine enantiomer **523a** was reported to be six times as active as its antipodal enantiomer, and their racemate showed a compromised activity [256]. The fungus-originated xanthones **495a**/**495b** and their racemate (±)-**495** were identified as potent *α*-glucosidase inhibitors with the levoisomer showing stronger activity [237]. The meroterpenoid enantiomers (+)-**339b**, (+)-**340a**, (−)-**341a** and (−)-**342a** displayed inhibitory effects against PTP1B with IC_50_ values ranging from 38.1 to 61.0 μM [176], while their respective antipodal enantiomers were considered inactive [178]. Two pairs of *N*-acetyldopamine enantiomers (**641** and **644**) derived from insect exhibited inhibitory activity against COX-2 with IC_50_ values in the range of 2.52–17.8 μM [305], and (+)-**641a** and (−)-**644b** were around two times as active as their respective antipodal enantiomers.

### 6.9. Aβ Aggregation Inhibition

Eleven pairs of plant-originated enantiomers including lignans (**1a**/**1b**, **2a**/**2b**, **6a**/**6b**, **7a**/**7b**, **73a**/**73b** and **76a**/**76b**) and alkaloids (**132a**/**132b**, **133a**/**1333b**, **241a**/**241b** and **242a**/**242b**) were evaluated for their inhibitory effects on β-amyloid (Aβ) aggregation, which had been considered as a central event in the pathogenesis of Alzheimer’s disease according to the “amyloid hypothesis”. Still, most enantiomeric pairs did not show much difference in Aβ aggregation inhibitory activity. Nevertheless, four pairs (**6a**/**6b**, **73a**/**73b**, **133a**/**1333b** and **242a**/**242b**) did display obvious activity variations at the enantiomeric level (Table 10). Notably, the 8,4′-oxyneolignan pair **6a**/**6b** presented a significant gap in their inhibition against Aβ aggregation, with the (−)-enantiomer showing a 141% activity increment compared with the (+)-enantiomer [17].

### 6.10. Miscellaneous Activities

In addition to the above-described biological properties, the enantiomers covered by the current review also showed positive responses in a variety of other bioassays. Those with obvious activity differences between enantiomers are listed in Table 11. The levorotary indole-diketopiperazine enantiomer (−)-**145a** exerted impact on MT_1_ and MT_2_ receptors with agonistic rates of 11.26% and 52.44% (at 0.25 mM), respectively, while its enantiomer (+)-**145b** was evaluated as inactive [93]. The (−)-enantiomer of diterpenoid **330** exhibited NF-κB inhibition with an IC_50_ value of 7.27 μM, while its (+)-enantiomer was considered inactive [169]. The fungus-derived indole-diketopiperazine enantiomer (+)-**530b** displayed antifouling activity against the barnacle *Balanus reticulatus* with an adhesive rate of 48.4% at 10 μg/cm^2^, while the (−)-enantiomer **530a** was inactive [259]. The nitrogen-rich alkaloid enantiomer (−)-**647b** was identified as a moderate protein-protein interaction inhibitor of HIF-1α and p300, while its antipodal enantiomer (+)-**647a** was inactive [308]. Lastly, the fish-produced dextrorotary lipid enantiomer (+)-**648a** elicited a strong olfactory response on the sea lamprey, and its levorotary enantiomer (−)-**648b** only showed weak activity [309].

## 7. Conclusions

**Figure 35:**
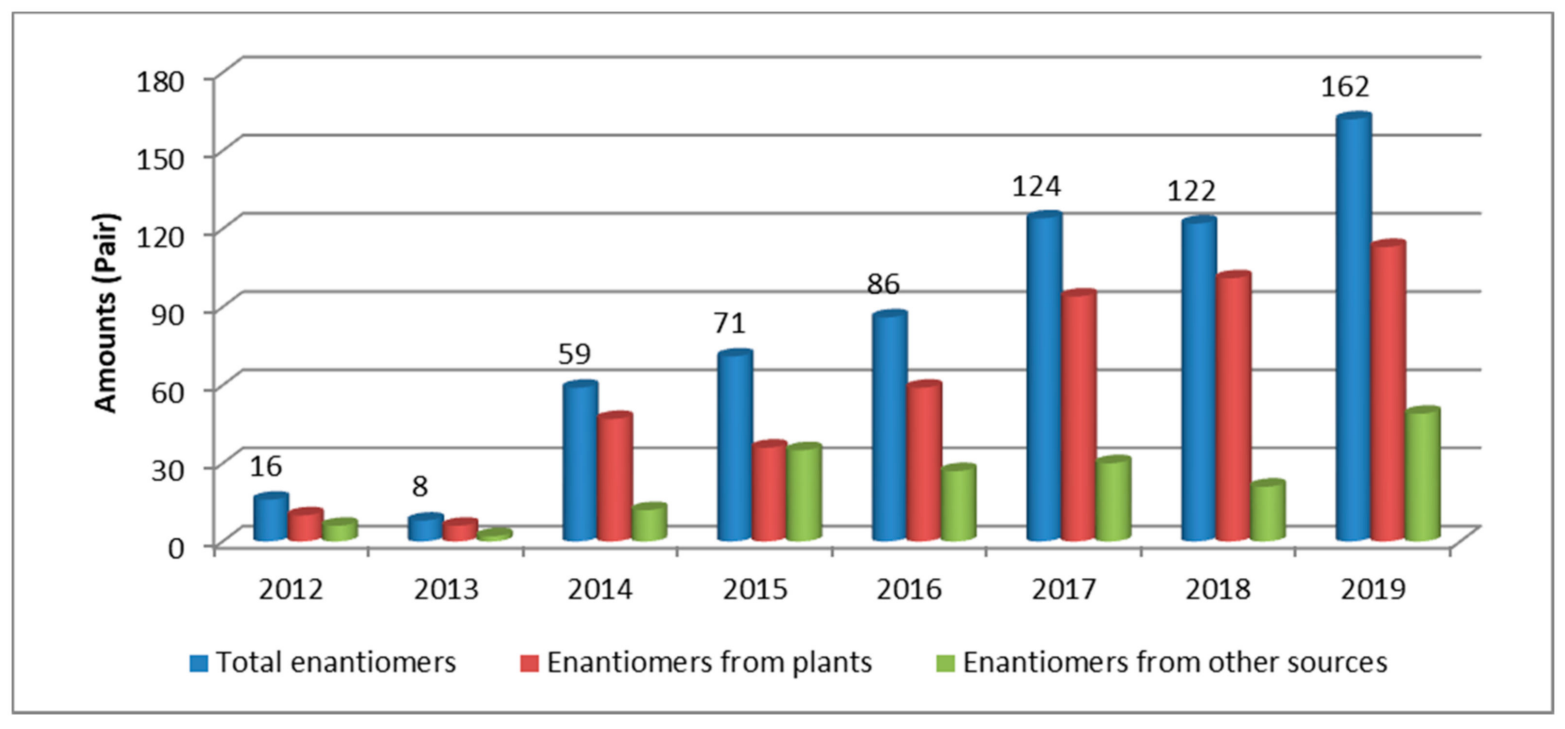
A comparison of enantiomeric pairs from plants and other sources.

**Figure 36:**
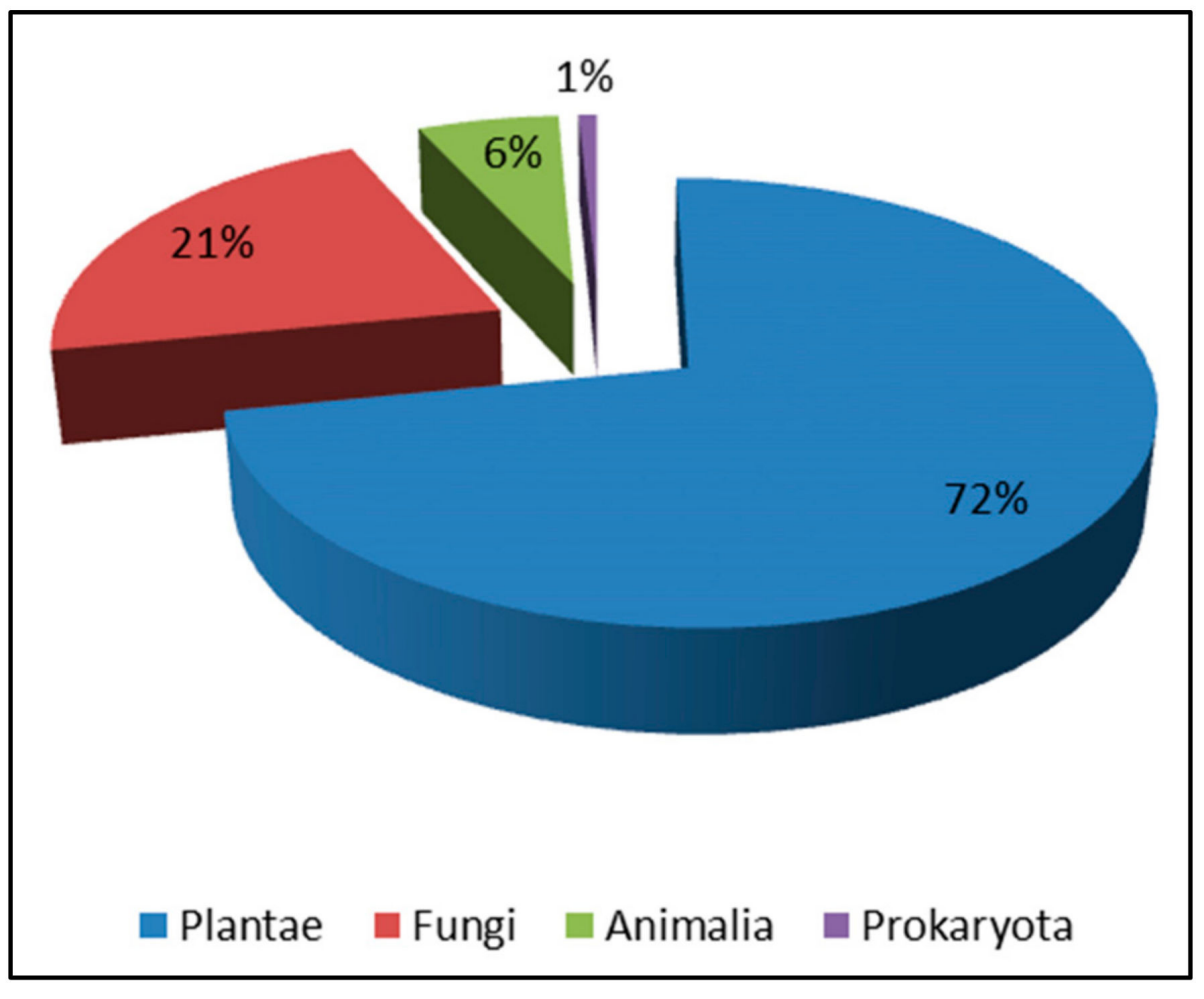
Distributions of enantiomers in kingdoms Plantae, Fungi, Animalia and Prokaryota.

**Figure 37:**
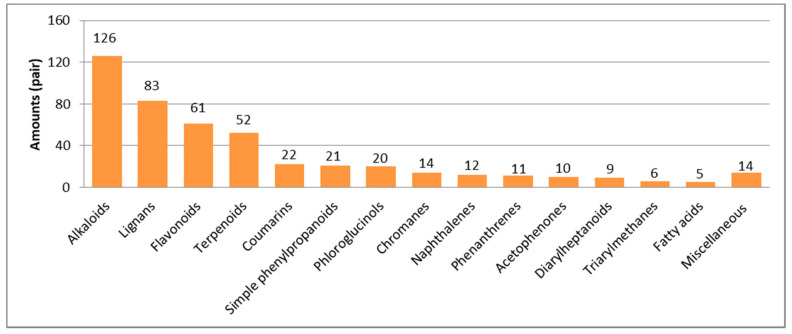
Statistics of different types of enantiomeric pairs from plants.

As can be seen from Figure 35, the number of identified natural enantiomers steadily increased during the period covered by this study, albeit with slight drops in 2013 and 2018. Notably, more than 100 enantiomers have been reported in the last three years (2017–2019), indicating rapid development in this field. It is also worth noting that plant-derived enantiomers made up 72% of all cases (Figure 36) in the study period, which suggests the continuing vitality of phytochemical studies, despite severe funding cutbacks for traditional NP research in recent years [3]. Another set of statistics (Figure 37) revealed that alkaloid enantiomers represent the biggest group of molecules from plants, followed by lignans and flavonoids.

### 7.1. Natural Distribution of Enantiomers

As demonstrated by the examples in this review, natural enantiomers have been widely reported from species of all kingdoms except Protoctista, which could be attributed to the fact that few NP researchers have been focusing on Protoctista organisms since they are not well-known sources of interesting molecules. Therefore, the discovery of enantiomers from Protoctista species in the near future is to be expected if NP workers continue to focus on them. From another perspective, the enantiomers collected in the period covered in this review have a broader distribution at the originated species level, from microbial fungi (e.g., mold) to macrofungi (e.g., mushrooms), from lower plants (e.g., moss) to higher plants (e.g., herbs), and from lower animals (e.g., sponges) to higher animals (e.g., fishes). Another noteworthy point is the distribution of enantiomers in different structural families, which can be clearly revealed by the examples in the current and previous reviews [3] that were discovered in all major structural classes such as terpenoids, alkaloids, flavonoids and polyketides (mainly from a biogenetic view). At the lower level of classification, it seems that there have been no enantiomeric cases reported for triterpenoids and steroids. The above-mentioned two points clearly demonstrate the universal occurrence of enantiomerism in nature.

### 7.2. Natural Formation of Enantiomers

It is interesting to note that unlike the previous report [3], in which many enantiomeric examples were obtained from different species, the majority of the cases collected in the current study were isolated from the same species as scalemic or racemic mixtures. Although Williams and colleagues predicted in 2012 [3] that the biogenetic studies of natural enantiomers would be “a fertile area for future inquiry and discovery”, there has been no significant progress in this research field since then. Nonetheless, some common reasons or rules regarding enantiomeric production can still be rationalized on the basis of currently available knowledge: (1) For cases in which the two antipodal enantiomers are produced by two different species (from the same or different genus or even different families), such as (+) and (–)-limonenes [3], two distinct enzymes and mechanisms are involved in their biosynthesis; (2) When an enantiomeric pair (racemic or scalemic mixture) is discovered from the same species, the lack (partially or completely) of stereo-specificity of the catalytic enzyme could be responsible for the enantiodivergent formation; (3) The absence of enzyme substrate or a completely chemical process would also lead to the production of two enantiomers, which is especially true for many NPs with only one chiral center. The following two explanations, though not as reasonable as the above-mentioned three, could also not be excluded. (4) In some biochemical processes which involve radicals, though normally stereo-controlled by enzymes, the generation of enantiomers is possible due to the extremely high reactivity of radicals. (5) The extraction and isolation procedures of NPs could also lead to the formation of new chiral centers, and thus, the production of enantiomers [310,311]. At this point, these enantiomeric molecules should be classified as NP derivatives or artifacts.

### 7.3. Structures Tend to Exist as Enantiomers in Nature

With the discovery of more and more enantiomeric NPs containing diverse structures, it can be concluded that enantiomerism may occur for each structural type, although no enantiomers have been reported for triterpenoids and steroids. Compared with enantiomers from plants, those from microorganisms are able to incorporate more complicated structures, e.g., with high molecular weights. It is possible that the enzyme systems in microorganisms are not fully developed and stereoselectivity is lacking. With this investigation into the structures of enantiomers reported from 2012–2019 in hand, we can easily conclude which structures or which groups in the structures tend to exist in nature as enantiomers. (1) NPs contain C6-C3 units in their structures, such as lignans, flavones, coumarins, simple phenylpropanoids, and hybrids between C6-C3 units and other structures. The enantiomerism for those structures presumably derives from the nonstereoselective oxidation of the C3 unit or nonstereoselective coupling of the C6-C3 units, through enzymatic or nonenzymatic reactions. (2) NPs formed by combination of 2~4 isopentenyl units, such as monoterpenoids, sesquiterpenoids, diterpenoids, and meroterpenoids, or having isopentenyl units as side chains, can exist in the form of enantiomers, and need to be further researched. (3) Alkaloid NPs have a variety of structural types, each of which may exist in the form of enantiomers. (4) When NPs with long chain, e.g., fatty acids and diarylheptanoids, have chiral centers, testing whether they are enantiomers or not is necessary. (5) NPs with axial chirality tend to exist as enantiomers in nature.

### 7.4. Identification of the Presence of Enantiomers

The criteria of enantiomeric presence vary, and a confirmative conclusion should be made based on comprehensive considerations. Ideally, the enantiomeric purity of every NP should be checked, but apparently this is neither economical nor technically feasible. Nevertheless, some general guidelines can still be summarized. Firstly, if a NP belongs to a structural group with strong enantiomeric tendency as listed in this review, such as 8,4′-oxyneolignans, caution is required. Secondly, for a previously undescribed NP, when its [α]_D_ value is very small (e.g., <5) or close to zero, the presence of an enantiomeric mixture should be considered. However, this method is not always fully indicative, as some chiral compounds naturally have low [α]_D_. For a known NP, regardless of whether the magnitude of [α]_D_ value is big or small, if it obviously deviates from the reported datum, the occurrence of enantiomerism is possible, and the purity of the tested NP should first be guaranteed. Thirdly, ECD measurement can also be used to check the enantiomeric purity of a NP (in case it shows a response in the experiment). A good-quality ECD curve usually looks smooth with clear Cotton effect(s) in the normal wavelength range (mostly 190–400 nm); if not, there is a high probability of enantiomeric presence. The aforementioned empirical knowledge is only based on general cases, and in fact, determination of the presence of enantiomers can be complicated. Notably, when the natural *e.e.* value of a pair of enantiomers is very high, as in the case of neosecurinane alkaloids [5], the researchers’ level of experience and sensitivity to chirality will make the difference.

### 7.5. Separation and Differentiation of Enantiomers

The separation (use of different chiral stationary materials) and differentiation (abs. config. assignment of an enantiomeric pair) of natural enantiomers were well documented in the review by Cass and Batista Jr. [10] and will not be included here. However, we do wish to emphasize that no omnipotent separation material and single technique can be applied for the purification and abs. config. determination, respectively, for all types of enantiomers, and any doubt regarding enantiomeric purity deserves further investigation.

### 7.6. Stereochemistry–Bioactivity Relationship of Enantiomers

As for the stereochemistry–bioactivity relationship (SBR) of natural enantiomers, analyses of the biological data gathered in this review do not provide many meaningful clues, and the relevance between the bioactivity and the chirality (dextroisomer or levoisomer) of a pair of enantiomers seems random and irregular in both enzymatic and cellular level bioassays. Although factors regarding the ‘chirality’ of life systems are well-known (e.g., d-glucose and l-amino acids as primary metabolites), there is still a long way to go before we are able to reveal the secrets of the exact SBR of enantiomers. Nevertheless, some general conclusions can still be reached according to the presently accessible information, similar to what Prof. Mori described for insect pheromones [4]. For a specific bioassay model: (1) One enantiomer is active, while the opposite enantiomer is less or not active, and the mixture of them does not result in any extra effect; (2) Both enantiomers are equally active, and their mixture does not result in any extra effect; (3) Both enantiomers are inactive or active, but their mixture is active or more active, suggestive of a synergistic action; (4) One enantiomer is active, whereas the antipodal enantiomer exhibits antagonistic activity, and thus, their mixture will exert an offset effect. Please note that the aforementioned general rules vary for different bioassays and are thus to be taken on a case-by-case basis, because all NPs are produced by the source organisms for their own use, and not for use by humans; we simply take advantage of their biological properties.

All in all, notwithstanding the rapidly growing number of reports and improving awareness of natural enantiomers in recent years, there are still a number of questions which remain to be answered. Our understanding of this fascinating natural phenomenon is only in its infancy

Here, we would like to say to the NP community that enantiomerism in nature is ubiquitous and vital. We hope that this review will prompt future researchers to routinely ask “Is my natural product enantiomerically pure, and if so, which enantiomer have I obtained?”, and in so doing, to perhaps even alter the methods applied by scientists in the future.

## Figures and Tables

**Table 1:** *J*_7,8_ Values, specific optical rotations and ECD data of 8,4′-oxyneolignians.

No.	C-7 & C-8 Configurations	*J*_7,8_ Values		Specific Optical Rotations			ECD Data	
		*J*_7,8_ (Hz)	Solvent	[α]_D_	Solvent	T (°C)	Δ*ε*	λ (nm)
1a	(7*R*,8*S*)-*erythro*	3.5	CD_3_OD	+3.3	MeOH	25	+2.25	239
1b	(7*S*,8*R*)-*erythro*	3.5	CD_3_OD	−4.7	MeOH	25	−2.75	239
2a	(7*R*,8*R*)-*threo*	6.8	CD_3_OD	−21.7	MeOH	25	−3.45	231
2b	(7*S*,8*S*)-*threo*	6.8	CD_3_OD	+16.0	MeOH	25	+2.83	232
3a	(7*S*,8*S*)-*threo*	8.8	CDCl_3_	+18.0	MeOH	20	−1.12	239
3b	(7*R*,8*R*)-*threo*	8.8	CDCl_3_	−20.0	MeOH	20	+1.40	237
4a	(7*R*,8*S*)-*erythro*	2.6	CDCl_3_	+20.5	MeOH	20	+2.38	245
4b	(7*S*,8*R*)-*erythro*	2.6	CDCl_3_	−22.0	MeOH	20	−1.89	243
5a	(7*R*,8*S*)-*erythro*	3.3	CDCl_3_	+32.0	MeOH	20	+0.25	238
5b	(7*S*,8*R*)-*erythro*	3.3	CDCl_3_	−28.2	MeOH	20	−0.01	238
6a	(7*S*,8*S*)-*threo*	6.9	CDCl_3_	+36.7	MeOH	20	+8.92	239
6b	(7*R*,8*R*)-*threo*	6.9	CDCl_3_	−33.5	MeOH	20	−6.50	240
7a	(7*R*,8*R*)-*threo*	8.0	CDCl_3_	−28.5	MeOH	20	+9.64	230
7b	(7*S*,8*S*)-*threo*	8.0	CDCl_3_	+26.9	MeOH	20	−8.74	230
8a	(7*R*,8*S*)-*erythro*	7.2	CDCl_3_	−31.0	MeOH	20	−3.55	240
8b	(7*S*,8*R*)-*erythro*	7.2	CDCl_3_	+29.0	MeOH	20	+2.80	240
9a	(7*S*,8*S*)-*threo*	7.4	CDCl_3_	+34.0	MeOH	20	+3.70	244
9b	(7*R*,8*R*)-*threo*	7.4	CDCl_3_	−34.0	MeOH	20	−2.81	243
10a	(7*R*,8*R*)-*threo*	6.5	CDCl_3_	−28.0	MeOH	20	+16.90	240
10b	(7*S*,8*S*)-*threo*	6.5	CDCl_3_	+32.0	MeOH	20	−17.65	238
11a	(7*R*,8*R*)-*threo*	7.6	CDCl_3_	−20.0	MeOH	20	−6.57	238
11b	(7*S*,8*S*)-*threo*	7.6	CDCl_3_	+21.0	MeOH	20	+8.87	242
12a	(7*R*,8*S*)-*erythro*	4.7	CDCl_3_	−31.0	MeOH	20	−3.55	240
12b	(7*S*,8*R*)-*erythro*	4.7	CDCl_3_	+29.0	MeOH	20	+2.80	240
13a	(7*S*,8*R*)-*erythro*	3.0	CDCl_3_	+17.1	CHCl_3_	20	−1.03	232
13b	(7*R*,8*S*)-*erythro*	3.0	CDCl_3_	−16.2	CHCl_3_	20	+1.03	232
14a	(7*S*,8*S*)-*threo*	6.3	CDCl_3_	−35.1	CHCl_3_	20	−1.14	240
14b	(7*R*,8*R*)-*threo*	6.3	CDCl_3_	+32.6	CHCl_3_	20	+1.17	240
15a	(7*R*,8*R*)-*threo*	8.1	CDCl_3_	+30.4	CHCl_3_	20	+1.58	240
15b	(7*S*,8*S*)-*threo*	8.1	CDCl_3_	−29.8	CHCl_3_	20	−1.54	240
16a	(7*S*,8*R*)-*erythro*	3.3	CDCl_3_	+16.6	CHCl_3_	20	+3.18	234
16b	(7*R*,8*S*)-*erythro*	3.3	CDCl_3_	−16.4	CHCl_3_	20	−3.20	234
17a	(7*R*,8*R*)-*threo*	8.0	CDCl_3_	+7.0	MeOH	20	-	-
17b	(7*S*,8*S*)-*threo*	8.0	CDCl_3_	−7.0	MeOH	20	-	-
18a	(7*S*,8*R*)-*erythro*	4.7	CDCl_3_	+20.0	MeOH	20	+11.17	230
18b	(7*R*,8*S*)-*erythro*	4.7	CDCl_3_	−18.0	MeOH	20	−4.00	235
19a	(7*S*,8*S*)-*threo*	6.1	CD_3_OD	+18.0	MeOH	20	+1.70	230
19b	(7*R*,8*R*)-*threo*	6.1	CD_3_OD	−18.0	MeOH	20	−1.20	230
20a	(7*R*,8*S*)-*erythro*	4.8	CD_3_OD	−10.0	MeOH	20	+2.70	232
20b	(7*S*,8*R*)-*erythro*	4.8	CD_3_OD	+8.0	MeOH	20	−2.82	233
21a	(7*S*,8*R*)-*erythro*	5.4	CD_3_OD	+15.0	MeOH	20	+3.15	228
21b	(7*R*,8*S*)-*erythro*	5.4	CD_3_OD	−15.0	MeOH	20	−2.65	228
22a	(7*S*,8*S*)-*threo*	7.7	CDCl_3_	+17.2	MeOH	20	−0.59	233
22b	(7*R*,8*R*)-*threo*	7.7	CDCl_3_	−19.0	MeOH	20	+0.51	232
23a	(7*R*,8*R*)-*threo*	7.6	CDCl_3_	−24.5	MeOH	20	-	-
23b	(7*S*,8*S*)-*threo*	7.6	CDCl_3_	+26.0	MeOH	20	-	-
24a	(7*R*,8*S*)-*erythro*	4.6	CDCl_3_	−18.0	MeOH	20	+12.20	241
24b	(7*S*,8*R*)-*erythro*	4.6	CDCl_3_	+22.0	MeOH	20	−10.81	241
25a	(7*S*,8*S*)-*threo*	6.8	CDCl_3_	+23.0	MeOH	20	+0.57	240
25b	(7*R*,8*R*)-*threo*	6.8	CDCl_3_	−25.0	MeOH	20	−1.05	237

**Table 2:** Cytotoxic activities of enantiomers. (**a**) part 1; (**b**) part 2.

Cell lines	Compds.	IC_50_ (μM)	Reference	Cell Lines	Compds.	IC_50_ (μM)	Reference
(**a**)							
HL-60	(+)-**171a**	5.62	[105]	HCT-116	(−)-**314a**	1.38	[162]
	(−)-**171b**	3.51	[105]		(+)-**314b**	>10	[162]
	(±)-**171**	2.65	[105]	HepG2	(−)-**314a**	3.30	[162]
	(+)-**172a**	9.64	[105]		(+)-**314b**	>10	[162]
	(−)-**172b**	8.16	[105]	BGC-823	(−)-**314a**	6.51	[162]
	(±)-**172**	5.58	[105]		(+)-**314b**	>10	[162]
Hep3B	(−)-**268a**	>10	[146]	NIC-H1650	(−)-**314a**	8.19	[162]
	(+)-**268b**	5.1	[146]		(+)-**314b**	>10	[162]
HL-60	(+)-**297a**	12.08	[156]	A2780	(−)-**314a**	2.14	[162]
	(−)-**297b**	19.24	[156]		(+)-**314b**	>10	[162]
MDA-MB-231	(+)-**297a**	>50	[156]	SF-268	(+)-**536a**	12.5	[261]
	(−)-**297b**	18.46	[156]		(−)-**536b**	>100	[261]
HCT-116	(+)-**312a**	inactive	[160]		(+)-**537a**	30.1	[261]
	(−)-**312b**	14.23	[160]		(−)-**537b**	>100	[261]
A549	(−)-**390a**	4.64	[8]	HepG2	(+)-**536a**	15.0	[261]
	(+)-**390b**	10.54	[8]		(−)-**536b**	>100	[261]
MCF-7	(−)-**390a**	5.60	[8]		(+)-**537a**	37.3	[261]
	(+)-**390b**	15.52	[8]		(−)-**537b**	>100	[261]
MDA-MB-231	(−)-**390a**	3.86	[8]				
	(+)-**390b**	11.86	[8]				
(**b**)							
HL-60	(+)-**381a**	3.42	[191,192]	MCF-7	(+)-**381a**	4.18	[191,192]
	(−)-**381b**	>20	[191,192]		(−)-**381b**	>20	[191,192]
	(−)-**382a**	16.54	[191,192]		(−)-**382a**	14.44	[191,192]
	(+)-**382b**	>40	[191,192]		(+)-**382b**	>40	[191,192]
	(±)-**382**	14.44	[191,192]		(±)-**382**	36.00	[191,192]
	(−)-**383a**	3.15	[191,192]		(−)-**383a**	5.85	[191,192]
	(+)-**383b**	2.35	[191,192]		(+)-**383b**	10.76	[191,192]
	(±)-**383**	18.08	[191,192]		(±)-**383**	17.05	[191,192]
	(−)-**384a**	3.45	[191,192]		(−)-**384a**	3.17	[191,192]
	(+)-**384b**	2.36	[191,192]		(+)-**384b**	3.08	[191,192]
	(±)-**384**	2.93	[191,192]		(±)-**384**	11.92	[191,192]
	(−)-**385a**	2.63	[191,192]		(−)-**385a**	14.60	[191,192]
	(+)-**385b**	5.41	[191,192]		(+)-**385b**	15.02	[191,192]
	(±)-**385**	13.90	[191,192]		(±)-**385**	15.47	[191,192]
SMMC-7721	(+)-**381a**	4.19	[191,192]	SW480	(+)-**381a**	7.22	[191,192]
	(−)-**381b**	>20	[191,192]		(−)-**381b**	>20	[191,192]
	(−)-**382a**	16.20	[191,192]		(−)-**382a**	17.43	[191,192]
	(+)-**382b**	>40	[191,192]		(+)-**382b**	>40	[191,192]
	(±)-**382**	22.83	[191,192]		(±)-**382**	27.73	[191,192]
	(−)-**383a**	5.55	[191,192]		(−)-**383a**	3.04	[191,192]
	(+)-**383b**	12.30	[191,192]		(+)-**383b**	5.53	[191,192]
	(±)-**383**	20.71	[191,192]		(±)-**383**	13.60	[191,192]
	(−)-**384a**	3.80	[191,192]		(−)-**384a**	1.99	[191,192]
	(+)-**384b**	10.83	[191,192]		(+)-**384b**	1.52	[191,192]
	(±)-**384**	15.36	[191,192]		(±)-**384**	7.62	[191,192]
	(−)-**385a**	12.53	[191,192]		(−)-**385a**	3.39	[191,192]
	(+)-**385b**	13.21	[191,192]		(+)-**385b**	4.31	[191,192]
	(±)-**385**	14.82	[191,192]		(±)-**385**	8.84	[191,192]
A549	(+)-**381a**	4.51	[191,192]	A549	(±)-**383**	24.02	[191,192]
	(−)-**381b**	>20	[191,192]		(−)-**384a**	2.96	[191,192]
	(−)-**382a**	17.27	[191,192]		(+)-**384b**	18.78	[191,192]
	(+)-**382b**	>40	[191,192]		(±)-**384**	15.44	[191,192]
	(±)-**382**	17.30	[191,192]		(−)-**385a**	22.36	[191,192]
	(−)-**383a**	4.40	[191,192]		(+)-**385b**	9.53	[191,192]
	(+)-**383b**	16.33	[191,192]		(±)-**385**	10.24	[191,192]

**Table 3:** Antiviral activities of enantiomers ^a^.

Virus/Host	Compds.	EC_50_ (μM)	IC_50_ (μM)	SI	Reference
HSV-1/Vero	(+)-**254a**	6.41	19.25	3.0	[7]
	(−)-**254b**	3.70	16.02	4.3	[7]
	(±)-**254**	1.23	2.14	1.7	[7]
	Acyclovir ^b^	>100	0.41	>243.9	[7]
Virus A (H3N3)/MDCK	(+)-**104a**	9.86	19.25	1.9	[67]
	(−)-**104b**	11.11	77.61	6.9	[67]
	(±)-**104**	3.13	6.42	2.1	[67]
	(−)-**105a**	8.62	77.61	7.9	[67]
	(+)-**105b**	17.46	57.74	3.3	[67]
	(±)-**105**	2.87	25.87	9.0	[67]
	Oseltamivir ^b^	3.38	3073	910.5	[67]
	Ribavirin ^b^	6.19	4771	770.7	[67]
KSHV/Vero	(+)-**368a**	8.75	140.6	16.06	[184]
	(−)-**368b**	29.13	173.7	5.96	[184]
	(+)-**369a**	202.9	>500	>2.46	[184]
	(−)-**369b**	140.9 17.67	>500	>2.55	[184]
	(+)-**370a**		211.1	12.51	[184]
	(−)-**370b**	39.80	>300	>7.50	[184]
	(+)-**371a**	40.00	>300	>7.50	[184]
	(−)-**371b**	158.50	>300	>1.89	[184]
	Acyclovir ^b^	0.41	99.18	241.9	[184]
EV71/Vero	(−)-**558a**	69.1	143.7	2.1	[272]
	(+)-**558b**	14.2	130.2	9.2	[272]
	(±)-**558**	14.2	126.6	7.9	[272]
	Ribavirin ^b^	>256.1	4098	>16	[272]

^a^ EC_50_ represents concentration required to inhibit virus growth by 50%; IC_50_ represents concentration required to inhibit host cell growth by 50%; SI (Selectivity index) = IC_50_/EC_50_. ^b^ Positive controls.

**Table 4:** Antibacterial activities of enantiomers.

Bacterial Strains	Compds.	Activities	Reference
*Enterococcus faecalis*	(+)-**147a**	MIC = 21.97 μg/mL	[97]
	(−)-**147b**	MIC = 12.54 μg/mL	[97]
	Penicilin ^a^	MIC < 2.96 μg/mL	[97]
*Staphylococcus aureus*	(+)-**285a**	IC_50_ = 1.27 μM	[153]
	(−)-**285b**	IC_50_ = 1.79 μM	[153]
	(+)-**286a**	IC_50_ = 2.27 μM	[153]
	(−)-**286b**	IC_50_ = 4.3 μM	[153]
	(+)-**287a**	IC_50_ = 3.6 μM	[153]
	(−)-**287b**	IC_50_ = 6.0 μM	[153]
	(+)-**288a**	IC_50_ = 0.61 μM	[153]
	(−)-**288b**	IC_50_ = 2.27 μM	[153]
	Chloramphenicol ^a^	IC_50_ = 0.43 μM	[153]

^a^ Positive controls.

**Table 5:** Antifungal activities of enantiomers.

Fungal Strains	Compds.	Activities	Reference
*Candida albicans*	(−)-**464a**	MIC = 26.2 μM	[219]
	(+)-**464b**	inactive	[219]
*Candida albicans*	(+)-**483a**	MIC_80_ = 19.5 μg/mL	[229]
	(−)-**483b**	MIC_80_ = 48.8 μg/mL	[229]
	(+)-**484a**	MIC_80_ = 24.0 μg/mL	[229]
	(−)-**484b**	MIC_80_ > 50.0 μg/mL	[229]
*Fusarium solani*	(+)-**524a**	MIC > 64 μg/mL	[256]
	(−)-**524b**	MIC = 32 μg/mL	[256]

**Table 6:** Anti-inflammatory activities of enantiomers.

Assay Model	Compds.	IC_50_ (μM)	Reference
BV-2/NO	(+)-**43a**	26.4	[39]
	(−)-**43b**	8.9	[39]
	(+)-**44a**	5.9	[39]
	(−)-**44b**	14.7	[39]
	Quercetin ^a^	17.0	[39]
RAW 264.7/NO	(+)-**82a**	17.9	[54]
	(−)-**82b**	5.6	[54]
	(+)-**83a**	15.1	[54]
	(−)-**83b**	4.3	[54]
RAW 264.7/NO	(+)-**221a**	3.6	[124]
	(−)-**221b**	22.8	[124]
	(±)-**221**	14.7	[124]
	Indomethacin ^a^	42.2	[124]
BV-2/NO	(+)-**407a**	6.9	[130]
	(−)-**407b**	1.4	[130]
	(±)-**407**	1.0	[130]
	Minocyclinebe ^a^	27.2	[130]

^a^ positive controls.

**Table 7:** Antioxidative activities of enantiomers.

Assay Model	Compds.	IC_50_ (μM)	Reference
DPPH	(+)-**527a**	5.8	[257]
	(−)-**527b**	23.5	[257]
	(+)-**528a**	9.8	[257]
	(−)-**528b**	24.9	[257]
	(+)-**529a**	3.7	[257]
	(−)-**529b**	6.1	[257]
	Ascorbic acid ^a^	23.0	[257]

^a^ Positive control.

**Table 8:** Cell protective activities of enantiomers.

Cell/Inducing Agents	Compds.	Cell Viability	Reference
SH-SY5Y/H_2_O_2_	(−)-**24a**	54.7% at 50 μM	[21]
	(+)-**24b**	70.5% at 50 μM	[21]
	Trolox ^a^	~69.0% at 50 μM	[21]
SH-SY5Y/H_2_O_2_	(+)-**72a**	~69% at 25 μM	[46]
	(−)-**72b**	inactive at 25 μM	[46]
	Trolox ^a^	~62% at 25 μM	[46]
SH-SY5Y/H_2_O_2_	(+)-**119a**	76.29% at 50 μM	[75]
	(−)-**119b**	56.48% at 50 μM	[75]
PC12/OKA	(+)-**129a**	65.4% at 10 μM	[83]
	(−)-**129b**	83.4% at 10 μM	[83]
	(+)-**130a**	91.2% at 10 μM	[83]
	(−)-**130b**	69.5% at 10 μM	[83]
H9C2/ischemia-hypoxia	(−)-**165a**	~51% at 0.1 μM	[103]
	(+)-**165b**	inactive	[103]
	(−)-**166a**	~55% at 0.1 μM	[103]
	(+)-**166b**	~45% at 0.1 μM	[103]
	(−)-**173a**	inactive	[103]
	(+)-**173b**	~52% at 0.1 μM	[103]
	Salvianolic acid B ^a^	~46% at 0.1 μM	[103]
HUVEC/glucose	(+)-**423a**	102.6% at 1 μM	[200]
	(−)-**423b**	79.9% at 1 μM	[200]
	(+)-**426a**	102.6% at 1 μM	[200]
	(−)-**426b**	79.9% at 1 μM	[200]
Cortical neurons/MPP^+^	(+)-**433a**	~90% at 16 μM	[206]
	(−)-**433b**	inactive	[206]

^a^ Positive controls.

**Table 9:** Enzyme inhibitory activities of enantiomers.

Enzymes	Compds.	IC_50_ (μM)	Reference
AchE	(+)-**157a**	>100	[100]
	(−)-**157b**	28.3	[100]
	Galanthamine ^a^	1.9	[100]
AchE	(+)-**523a**	2.3	[256]
	(−)-**523b**	13.8	[256]
	(±)-**523**	9.5	[256]
	Tacrine ^a^	0.14	[256]
α-Glucosidase	(+)-**495a**	63.7	[237]
	(−)-**495b**	27.9	[237]
	(±)-**495**	36.1	[237]
	Acarbose ^a^	477.0	[237]
PTP1B	(−)-**339a**	Inactive	[176]
	(+)-**339b**	43.6	[176]
	(+)-**340a**	38.1	[176]
	(−)-**340b**	Inactive	[176]
	(−)-**341a**	61.0	[176]
	(+)-**341b**	Inactive	[176]
	(−)-**342a**	58.2	[176]
	(+)-**342b**	Inactive	[176]
	Oleanolic acid ^a^	2.5	[176]
COX-2	(+)-**641a**	2.52	[305]
	(−)-**641b**	6.04	[305]
	(+)-**644a**	17.8	[305]
	(−)-**644b**	9.7	[305]
	Celecoxib ^a^	0.016	[305]

^a^ Positive controls.

**Table 10:** Aβ aggregation inhibition of enantiomers.

Compds.	Inhibition (%)	Reference
(+)-**6a**	31.2	[19]
(−)-**6b**	75.3	[19]
Curcumin ^a^	62.1	[19]
(+)-**73a**	62.1	[51]
(−)-**73b**	81.6	[51]
Curcumin ^a^	63.2	[51]
(+)-**132a**	85.8	[85]
(−)-**132b**	73.6	[85]
Curcumin ^a^	57.0	[85]
(+)-**242a**	33.9	[131]
(−)-**242b**	50.6	[131]
Curcumin ^a^	63.3	[131]

^a^ Positive controls.

**Table 11:** Miscellaneous activities of enantiomers.

Models	Compds.	Activities	Reference
MT_1_ receptor agonistic activity	(−)-**145a**	agonistic rate = 11.26%	[93]
	(+)-**145b**	inactive	[93]
MT_2_ receptor agonistic activity	(−)-**145a**	agonistic rate = 52.44%	[93]
	(+)-**145b**	inactive	[93]
NF-κB inhibition	(+)-**330a**	inactive	[169]
	(−)-**330b**	IC_50_ = 7.27 μM	[169]
Antifouling activity	(−)-**530a**	inactive	[259]
	(+)-**530b**	adhesive rate = 48.4%	[259]
Protein-protein interaction inhibition	(+)-**647a**	inactive	[308]
	(−)-**647b**	modestly	[308]
Olfactory responses	(+)-**648a**	strong	[309]
	(−)-**648b**	weak	[309]

## Data Availability

Not applicable.

## Supplementary Materials

The following supporting information can be downloaded at: Tables S1−S34 contain names, source species and references of all collected enantiomers. Refs [312,313,314,315,316] are cited in the Supplementary Materials
