# Supplementary material for: Natural Enantiomers: Occurrence, Biogenesis and Biological Properties
Source: Molecules. 2022 Feb 14;27(4):1279. doi: 10.3390/molecules27041279 (PMC8880303; doi:10.3390/molecules27041279)
Supplement: Supplementary file 1 [file molecules-27-01279-s001.zip › molecules-1564733-supplementary.pdf]

Supporting Information for

Natural Enantiomers: Occurrence, Biogenesis and Biological Properties

Jin-Hai Yu,[a] Zhi-Pu Yu,[a] Robert J. Capon,[b,*] Hua Zhang[a,*]

[a]School of Biological Science and Technology, University of Jinan, 336 West Road of Nan Xinzhuang, Jinan 250022, China

[b] Institute for Molecular Bioscience, The University of Queensland, St. Lucia, QLD 4072, Australia

*Corresponding authors: H. Zhang (email: bio_zhangh@ujn.edu.cn); R. J. Capon (email: r.capon@uq.edu.au)

Table of Contents

Table S1. Names, source species and references of 8,4'-oxylignan enantiomers 1
Table S2. Names, source species and references of other acyclic lignan enantiomers....3
Table S3. Names, source species and references of furan-incorporating lignan enantiomers 4
Table S4. Names, source species and references of other cyclic lignan enantiomers....5
Table S5. Names, source species and references of sesquineolignan enantiomers....6
Table S6. Names, source species and references of coumarin enantiomers 7
Table S7. Names, source species and references of simple phenylpropanoid enantiomers....8
Table S8. Names, source species and references of indole alkaloid enantiomers 9
Table S9. Names, source species and references of quinoline and isoquinoline alkaloid enantiomers 10
Table S10. Names, source species and references of *β*-carboline and carbazole alkaloid enantiomers 11
Table S11. Names, source species and references of piperidine alkaloid enantiomers 12
Table S12. Names, source species and references of thiohydantoin alkaloid enantiomers 13
Table S13. Names, source species and references of indolizidine and quinolizidine alkaloid enantiomers 14
Table S14. Names, source species and references of other alkaloid enantiomers 15
Table S15. Names, source species and references of flavone and isoflavone enantiomers....17
Table S16. Names, source species and references of chalcone enantiomers 19
Table S17. Names, source species and references of xanthone enantiomers....20
Table S18. Names, source species and references of sesquiterpenoid enantiomers 21
Table S19. Names, source species and references of diterpenoid enantiomers 22
Table S20. Names, source species and references of meroterpenoid enantiomers....23
Table S21. Names, source species and references of phloroglucinol enantiomers....24
Table S22. Names, source species and references of naphthalene and phenanthrene enantiomers 25
Table S23. Names, source species and references of chromane enantiomers 27
Table S24. Names, source species and references of acetophenone enantiomers 28
Table S25. Names, source species and references of diarylheptanoid enantiomers 29
Table S26. Names, source species and references of triarylmethane enantiomers....30
Table S27. Names, source species and references of fatty acid enantiomers 31
Table S28. Names, source and species of miscellaneous enantiomers....32
Table S29 Names, source species and references of non-alkaloids enantiomers from phylum Ascomycota....33
Table S30. Names, source species and references of alkaloid enantiomers from phylum Ascomycota....35
Table S31. Names, source species and references of enantiomers from phylum Basidiomycota ..37
Table S32. Names and species of enantiomeric compounds from actinomycetes....39
Table S33. Names, source species and references of enantiomers from phylum Porifera 40
Table S34. Names, source species and references of enantiomers from phyla Arthropoda and Chordata....42
References 43

Table S1. Names, source species and references of 8,4'-oxylignan enantiomers

No	Name	Species & Reference
**1a**	(7*R*,8*S*)-3′,4,7,9′-tetrahydroxy-3-methoxy-8,4′-oxyneolignan	*Paeonia lactiflora*[1]
**1b**	(7*S*,8*R*)-3′,4,7,9′-tetrahydroxy-3-methoxy-8,4′-oxyneolignan	*Paeonia lactiflora*[1]
**2a**	(7*R*,8*R*)-3′,4,7,9′-tetrahydroxy-3-methoxy-8,4′-oxyneolignan	*Paeonia lactiflora*[1]
**2b**	(7*S*,8*S*)-3′,4,7,9′-tetrahydroxy-3-methoxy-8,4′-oxyneolignan	*Paeonia lactiflora*[1]
**3a**	(+)-7*S*,8*S*-4,7,9,9'-tetrahydroxy-3,3'5'-trimethoxy-8,4'-oxyneolignan	*Crataegus pinnatifida*[2]
**3b**	(−)-7*R*,8*R*-4,7,9,9'-tetrahydroxy-3,3'5'-trimethoxy-8,4'-oxyneolignan	*Crataegus pinnatifida*[2]
**4a**	(−)-7*R*,8*S*-4,7,9,9'-tetrahydroxy-3,3',5'-trimethoxy-8,4'-oxyneolignan	*Crataegus pinnatifida*[2]
**4b**	(+)-7*S*,8*R*-4,7,9,9'-tetrahydroxy-3,3',5'-trimethoxy-8,4'-oxyneolignan	*Crataegus pinnatifida*[2]
**5a**	(+)-7*R*,8*S*-4,7,9,9'-tetrahydroxy-3,5,3',5'-tetramethoxy-8,4'-oxyneolignan	*Crataegus pinnatifida*[2]
**5b**	(−)-7*S*,8*R*-4,7,9,9'-tetrahydroxy-3,5,3',5'-tetramethoxy-8,4'-oxyneolignan	*Crataegus pinnatifida*[2]
**6a**	7*S*,8*S*-4,7,9,9′-tetrahydroxy-8,4′-oxyneolignan	*Rubus idaeus*[3]
**6b**	7*R*,8*R*-4,7,9,9′-tetrahydroxy-8,4′-oxyneolignan	*Rubus idaeus*[3]
**7a**	7*R*,8*R*-4,7,9,9′-tetrahydroxy-3′-methoxy-8,4′-oxyneolignan	*Rubus idaeus*[3]
**7b**	7*S*,8*S*-4,7,9,9′-tetrahydroxy-3′-methoxy-8,4′-oxyneolignan	*Rubus idaeus*[3]
**8a**	(−)-7*R*,8*S*-4,9,9′-trihydroxy-3,7,3′-trimethoxy-8,4′-oxyneolignan	*Ailanthus altissima*[4]
**8b**	(+)-7*S*,8*R*-4,9,9′-trihydroxy-3,7,3′-trimethoxy-8,4′-oxyneolignan	*Ailanthus altissima*[4]
**9a**	(+)-7*S*,8*S*-4,9,9′trihydroxy-3,7,3′-trimethoxy-8,4′-oxyneolignan	*Ailanthus altissima*[4]
**9b**	(−)-7*R*,8*R*-4,9,9′trihydroxy-3,7,3′-trimethoxy-8,4′-oxyneolignan	*Ailanthus altissima*[4]
**10a**	(−)-7*R*,8*R*-4,9,9′-trihydroxy-7,3′,5′-trimethoxy-8,4′-oxyneolignan	*Ailanthus altissima*[4]
**10b**	(+)-7*S*,8*S*-4,9,9′-trihydroxy-7,3′,5′-trimethoxy-8,4′-oxyneolignan	*Ailanthus altissima*[4]
**11a**	(−)-7*R*,8*R*-4,9,9′-trihydroxy-3,7,3′,5′-tetramethoxy-8,4′- oxyneolignan	*Ailanthus altissima*[4]
**11b**	(+)-7*S*,8*S*-4,9,9′-trihydroxy-3,7,3′,5′-tetramethoxy-8,4′-oxyneolignan	*Ailanthus altissima*[4]
**12a**	(−)-7*R*,8*S*-4,7,9,9′-trihydroxy-3′-methoxy-8,4′-oxyneolignan	*Ailanthus altissima*[4]
**12b**	(−)-7*S*,8*R*-4,7,9,9′-trihydroxy-3′-methoxy-8,4′-oxyneolignan	*Ailanthus altissima*[4]
**13a**	(+)-(7′*S*,8′*R*)-*erythro*-7′-methylcarolignan E	*Euphorbia sikkimensis*[5]
**13b**	(−)-(7′*R*,8′*S*)-*erythro*-7′-methylcarolignan E	*Euphorbia sikkimensis*[5]
**14a**	(−)-(7′*S*,8′*S*)-*threo*-7′-methylcarolignan E	*Euphorbia sikkimensis*[5]
**14b**	(+)-(7′*R*,8′*R*)-*threo*-7′-methylcarolignan E	*Euphorbia sikkimensis*[5]
**15a**	(+)-(7′*R*,8′*R*)-*threo*-carolignan E	*Euphorbia sikkimensis*[5]
**15b**	(−)-(7′*S*,8′*S*)-*threo*-carolignan E	*Euphorbia sikkimensis*[5]
**16a**	(+)-(7′*S*,8′*R*)-*erythro*-carolignan E	*Euphorbia sikkimensis*[5]
**16b**	(−)-(7′*R*,8′*S*)-*erythro*-carolignan E	*Euphorbia sikkimensis*[5]
**17a**	(7′*R*,8′*R*)-brachangobinan C	*Brachanthemum gobicum*[6]
**17b**	(7′S, 8′*S*)-brachangobinan C	*Brachanthemum gobicum*[6]
**18a**	(−)-7*R*,8*S*-4,7,9-trihydroxy-3,3′,9′-trimethoxy-1′-allyl-8,4′-oxyneolignan	*Ailanthus altissima*[4]
**18b**	(+)-7*S*,8*R*-4,7,9-trihydroxy-3,3′,9′-trimethoxy-1′-allyl-8,4′-oxyneolignan	*Ailanthus altissima*[4]
**19a**	(+)-acortatarinowin A	*Acorus tatarinowii*[7]
**19b**	(−)-acortatarinowin A	*Acorus tatarinowii*[7]
**20a**	(−)-acortatarinowin B	*Acorus tatarinowii*[7]
**20b**	(+)-acortatarinowin B	*Acorus tatarinowii*[7]
**21a**	(+)-acortatarinowin C	*Acorus tatarinowii*[7]
**21b**	(−)-acortatarinowin C	*Acorus tatarinowii*[7]

No	Name	Species & Reference
**22a**	(+)-7*S*,8*S*-guaiacylglycerol-8-acetovanillone ether	*Crataegus pinnatifida*[2]
**22b**	(−)-7*R*,8*R*-guaiacylglycerol-8-acetovanillone ether	*Crataegus pinnatifida*[2]
**23a**	(−)-7*R*,8*R*-guaiacylglycerol-8-vanillin ether	*Crataegus pinnatifida*[2]
**23b**	(+)-7*S*,8*S*-guaiacylglycerol-8-vanillin ether	*Crataegus pinnatifida*[2]
**24a**	(−)-7*R*,8*S*-guaiacylglycerol 8-(4-hydroxymethyl-2-ethoxyphenyl) ether	*Ailanthus altissima*[4]
**24b**	(+)-7*S*,8*R*-guaiacylglycerol 8-(4-hydroxymethyl-2-ethoxyphenyl) ether	*Ailanthus altissima*[4]
**25a**	(+)-acortatarinowin D	*Acorus tatarinowii*[7]
**25b**	(−)-acortatarinowin D	*Acorus tatarinowii*[7]

Table S2. Names, source species and references of other acyclic lignan enantiomers

No	Name	Species & Reference
**26a**	(+)-asarolignan B	*Acorus tatarinowii*[8]
**26b**	(−)-asarolignan B	*Acorus tatarinowii*[8]
**27a**	(+)-asarolignan C	*Acorus tatarinowii*[8]
**27b**	(−)-asarolignan C	*Acorus tatarinowii*[8]
**28**	(±)-retusiusine C	*Bulbophyllum retusiusculum*[9]
**29a**	(+)-torreyunlignan A	*Torreya yunnanensis*[10]
**29b**	(−)-torreyunlignan A	*Torreya yunnanensis*[10]
**30a**	(−)-torreyunlignan B	*Torreya yunnanensis*[10]
**30b**	(+)-torreyunlignan B	*Torreya yunnanensis*[10]
**31a**	(−)-torreyunlignan C	*Torreya yunnanensis*[10]
**31b**	(+)-torreyunlignan C	*Torreya yunnanensis*[10]
**32a**	(−)-torreyunlignan D	*Torreya yunnanensis*[10]
**32b**	(+)-torreyunlignan D	*Torreya yunnanensis*[10]
**33**	(±)-asarolignan E	*Acorus tatarinowii*[8]
**34**	(±)-asarolignan F	*Acorus tatarinowii*[8]
**35**	(±)-asarolignan D	*Acorus tatarinowii*[8]
**36a**	(+)-liriodenol	*Liriodendron hybrid*[11]
**36b**	(−)-liriodenol	*Liriodendron hybrid*[11]
**37a**	(−)-selamoellenin D	*Selaginella moellendorffii*[12]
**37b**	(+)-selamoellenin D	*Selaginella moellendorffii*[12]
**38a**	alashinol I	*Syringa pinnatifolia*[13]
**38b**	alashinol J	*Syringa pinnatifolia*[13]
**39a**	(8*R*)-brachangobinan B	*Brachanthemum gobicum*[6]
**39b**	(8S)-Brachangobinan B	*Brachanthemum gobicum*[6]

Table S3. Names, source species and references of furan-incorporating lignan enantiomers

No	Name	Species & Reference
**40a**	(+)-acortatarinowin E	*Acorus tatarinowii*[14]
**40b**	(−)-acortatarinowin E	*Acorus tatarinowii*[14]
**41a**	(−)-acortatarinowin I	*Acorus tatarinowii*[15]
**41b**	(+)-acortatarinowin I	*Acorus tatarinowii*[15]
**42a**	(+)-jatrointelignan C	*Jatropha integerrima*[16]
**42b**	(−)-(7′*R*,8′*S*)-5′-methoxyl-(dimeric coniferyl acetate)	*Jatropha integerrima*[16]
**43a**	(+)-jatrointelignan D	*Jatropha integerrima*[16]
**43b**	(−)-jatrointelignan D	*Jatropha integerrima*[16]
**44a**	(+)-schisphenlignan I	*Jatropha integerrima*[16]
**44b**	(−)-schisphenlignan I	*Jatropha integerrima*[16]
**45a**	(7′*S*,8′*R*)-brachangobinan A	*Brachanthemum gobicum*[6]
**45b**	(7′*R*,8′*S*)-brachangobinan A	*Brachanthemum gobicum*[6]
**46a**	(+)-(7*S*,8*R*)-dehydrodiconiferyl alcohol	*Picrasma quassioides*[17]
**46b**	(−)-(7*R*, 8*S*)-dehydrodiconiferyl alcohol	*Picrasma quassioides*[17]
**47a**	(−)-rasidasin I	*Rubus idaeus*[18]
**47b**	(+)-rasidasin I	*Rubus idaeus*[18]
**48a**	(7*S*,8*R*)-idaeusin D	*Rubus ideaus*[19]
**48b**	(7*R*,8*S*)-idaeusin D	*Rubus ideaus*[19]
**49a**	(−)-phyllanglaucin A	*Phyllanthus glaucus*[20]
**49b**	(+)-phyllanglaucin A	*Phyllanthus glaucus*[20]
**50a**	(7′*R*,8′*S*)-brachangobinan H	*Brachanthemum gobicum*[6]
**50b**	(7′*S*,8′*R*)-brachangobinan H	*Brachanthemum gobicum*[6]
**51a**	(−)-(7*R*,8*S*)-balanophonin	*Picrasma quassioides*[17]
**51b**	(+)-(7*S*,8*R*)-balanophonin	*Picrasma quassioides*[17]
**52a**	(−)-(7*R*,8*S*)-5-methoxyl-balanophonin	*Picrasma quassioides*[17]
**52b**	(+)-(7S,8R)-5-methoxyl-balanophonin	*Picrasma quassioides*[17]
**53a**	(7*R*,8*S*)-idaeusin C	*Rubus ideaus*[18]
**53b**	(7*S*,8*R*)-idaeusin C	*Rubus ideaus*[18]
**54a**	(−)-rasidasin II	*Rubus idaeus*[18]
**54b**	(+)-rasidasin II	*Rubus idaeus*[18]
**55a**	(7*S*,8*R*)-idaeusin B	*Rubus ideaus*[19]
**55b**	(7*R*,8*S*)-idaeusin B	*Rubus ideaus*[19]
**56a**	(7′*R*,8′*S*)-brachangobinan I	*Brachanthemum gobicum*[6]
**56b**	(7′*S*,8′*R*)-brachangobinan I	*Brachanthemum gobicum*[6]
**57a**	(7*R*,8*S*)-idaeusin A	*Rubus ideaus*[19]
**57b**	(7S,8R)-idaeusin A	*Rubus ideaus*[19]
**58a**	(+)-idaeusinol A	*Rubus idaeus*[21]
**58b**	(−)-idaeusinol A	*Rubus idaeus*[21]
**59a**	(*1R*,*5S*,*6S*)-kachiranol	*Archidendron clypearia*[22]
**59b**	(*1S*,*5R*,*6R*)-kachiranol	*Archidendron clypearia*[22]
**60a**	(+)-dendrolactone	*Dendrobium nobile*[23]
**60b**	(−)-dendrolactone	*Dendrobium nobile*[23]
**61a**	(+)-idaeusinol B	*Rubus idaeus*[21]
**61b**	(−)-idaeusinol B	*Rubus idaeus*[21]
**62a**	(+)-morifolia A	*Morinda citrifolia*[24]
**62b**	(−)-morifolia A	*Morinda citrifolia*[24]
**63a**	(+)-acortatarinowin F	*Acorus tatarinowii*[14]
**63b**	(−)-acortatarinowin F	*Acorus tatarinowii*[14]

Table S4. Names, source species and references of other cyclic lignan enantiomers

No	Name	Species & Reference
**64a**	(7*S*, 8*S*)-pithecellobiumin A	*Pithecellobium clypearia*[25]
**64b**	(7*R*,8*R*)-pithecellobiumin A	*Pithecellobium clypearia*[25]
**65a**	(+)-acortatarinowin G	*Acorus tatarinowii*[15]
**65b**	(−)-acortatarinowin G	*Acorus tatarinowii*[15]
**66a**	(+)-asarolignan G	*Acorus tatarinowii*[8]
**66b**	(−)-asarolignan G	*Acorus tatarinowii*[8]
**67a**	(7*R*,8*R*,8′*R*)-pithecellobiumin B	*Pithecellobium clypearia*[25]
**67b**	(7*S*,8*S*,8′*S*)-pithecellobiumin B	*Pithecellobium clypearia*[25]
**68a**	(+)-acortatarinowin H	*Acorus tatarinowii*[15]
**68b**	(−)-acortatarinowin H	*Acorus tatarinowii*[15]
**69a**	(+)-(2*S*,3*R*,2'*S*,3'*R*)-isatiscycloneolignan A	*Isatis indigotica*[26]
**69b**	(−)-(2R,3*S*,2'*R*,3'*S*)-isatiscycloneolignan A	*Isatis indigotica*[26]
**70a**	(+)-tylopilusin A	*Tylopilus eximius*[27]
**70b**	(−)-tylopilusin A	*Tylopilus eximius*[27]
**71a**	(+)-tylopilusin B	*Tylopilus eximius*[27]
**71b**	(−)-tylopilusin B	*Tylopilus eximius*[27]
**72a**	(+)-subaveniumin A	*Cinnamomum subavenium*[28]
**72b**	(−)-subaveniumin A	*Cinnamomum subavenium*[28]
**73a**	(+)-subaveniumin A	*Cinnamomum subavenium*[28]
**73b**	(−)-subaveniumin A	*Cinnamomum subavenium*[28]

Table S5. Names, source species and references of sesquineolignan enantiomers

No	Name	Species & Reference
**74a**	(+)-phyllanglaucin B	*Phyllanthus glaucus*[20]
**74b**	(−)-phyllanglaucin B	*Phyllanthus glaucus*[20]
**75a**	(+)-phyllanglaucin C	*Phyllanthus glaucus*[20]
**75b**	(−)-phyllanglaucin C	*Phyllanthus glaucus*[20]
**76a**	(+)-phyllanglaucin D	*Phyllanthus glaucus*[20]
**76b**	(−)-phyllanglaucin D	*Phyllanthus glaucus*[20]
**77a**	(+)-(7*R*,8*S*,7′*R*,8′*R*)-acernikol	*Phyllanthus glaucus*[20]
**77b**	(−)-(7*S*,8R,7′*S*,8′*S*)-acernikol	*Phyllanthus glaucus*[20]
**78a**	(−)-(7*R*,8*S*,7′*S*,8′*S*)-acernikol	*Phyllanthus glaucus*[20]
**78b**	(+)-(7*S*,8*R*,7′*R*,8′*R*)-acernikol	*Phyllanthus glaucus*[20]
**79a**	(+)-jatrointelignan A	*Jatropha integerrima*[16]
**79b**	(−)-jatrointelignan A	*Jatropha integerrima*[16]
**80a**	(+)-jatrointelignan B	*Jatropha integerrima*[16]
**80b**	(−)-jatrointelignan B	*Jatropha integerrima*[16]
**81a**	(+)-sibiricumin A	*Xanthium sibiricum*[29]
**81b**	(−)-sibiricumin A	*Xanthium sibiricum*[29]
**82a**	(+)-idaeusinol C	*Rubus idaeus*[21]
**82b**	(−)-idaeusinol C	*Rubus idaeus*[21]
**83a**	(+)-idaeusinol D	*Rubus idaeus*[21]
**83b**	(−)-idaeusinol D	*Rubus idaeus*[21]

Table S6. Names, source species and references of coumarin enantiomers

No	Name	Species & Reference
**84a**	(3′*S*,4′*S*)-3′-*O*-acetyl-4′-*O*-senecioylkhellactone	*Peucedanum japonicum*[30]
**84b**	(3′*R*,4′*R*)-3′-*O*-acetyl-4′-*O*-senecioylkhellactone	*Angelica morii*[31]
**85a**	(3′*S*,4′*S*)-4′-*O*-angeloyl-3′-*O*-(2-methylbutyroyl)-khellactone	*Peucedanum japonicum*[30]
**85b**	(3′*R*,4′*R*)-4′-*O*-angeloyl-3′-*O*-(2-methylbutyroyl)-khellactone	*Peucedanum praeruptorum*[32]
**86a**	(3′*S*,4′*S*)-4′-*O*-angeloyl-3′-*O*-isovaleroylkhellactone	*Peucedanum japonicum*[30]
**86b**	(3′*R*,4′*R*)-4′-*O*-angeloyl-3′-*O*-isovaleroylkhellactone	*Seseli gummiferum*[33]
**87a**	(3′*S*,4′*S*)-3′-*O*-acetyl-4′-*O*-(2-methylbutyroyl)-khellactone	*Peucedanum japonicum*[30]
**87b**	(3′*R*,4′*R*)-3′-*O*-acetyl-4′-*O*-(2-methylbutyroyl)-khellactone	*Angelica furcijuga*[34]
**88a**	(+)-(3′*S*,4′*S*)-3′-isovaleryl-4′-acetylkhellactone	*Peucedani radix*[35]
**88b**	(–)-(3′*R*,4′*R*)-3′-isovaleryl-4′-acetylkhellactone	*Peucedani radix*[35]
**89a**	(+)-(3′*S*,4′*S*)-3′,4′-diisovalerylkhellactone	*Peucedani radix*[35]
**89b**	(–)-(3′*R*,4′*R*)-3′,4′-diisovalerylkhellactone	*Peucedani radix*[35]
**90a**	(–)-(3′*S*,4′*S*)-3′-acetyl-4′-angeloylkhellactone	*Peucedani radix*[35]
**90b**	(+)-(3′*R*,4′*R*)-3′-acetyl-4′-angeloylkhellactone	*Peucedani radix*[35]
**91a**	(+)-(3′*S*,4′*S*)-3′-angeloyl-4′-isovalerylkhellactone	*Peucedani radix*[35]
**91b**	(–)-(3′*R*,4′*R*)-3′-angeloyl-4′-isovalerylkhellactone	*Peucedani radix*[35]
**92a**	(+)-(3′*S*,4′*R*)-3′-acetyl-4′-isobutyrylkhellactone	*Peucedani radix*[35]
**92b**	(–)-(3′*R*,4′*S*)-3′-acetyl-4′-isobutyrylkhellactone	*Peucedani radix*[35]
**93a**	(+)-(3′*S*,4′*R*)-3′-angeloylkhellactone	*Peucedani radix*[35]
**93b**	(–)-(3′*R*,4′*S*)-3′-angeloylkhellactone	*Peucedani radix*[35]
**94a**	(+)-(3′*S*,4′*R*)-3′-acetyl-4′-angeloylkhellactone	*Peucedani radix*[35]
**94b**	(–)-(3′*R*,4′*S*)-3′-acetyl-4′-angeloylkhellactone	*Peucedani radix*[35]
**95a**	(+)-(3′*S*)-3′angeloyloxy-4′-oxo-3′,4′-dihydroseselin	*Peucedani radix*[35]
**95b**	(–)-(3′*R*)-3′angeloyloxy-4′-oxo-3′,4′-dihydroseselin	*Peucedani radix*[35]
**96a**	(+)-toddalin E	*Toddalia asiatica*[36]
**96b**	(–)-toddalin E	*Toddalia asiatica*[36]
**97a**	(+)-toddalin E	*Toddalia asiatica*[36]
**97b**	(–)-toddalin E	*Toddalia asiatica*[36]
**98a**	(+)-baccatune A	*Sapium baccatum*[37]
**98b**	(–)-baccatune A	*Sapium baccatum*[37]
**99a**	(+)-baccatune B	*Sapium baccatum*[37]
**99b**	(–)-baccatune B	*Sapium baccatum*[37]
**100a**	(+)-baccatune G	*Sapium baccatum*[37]
**100b**	(–)-baccatune G	*Sapium baccatum*[37]
**101a**	(*P*)-corinepalenin A	*Coriaria nepalensis*[38]
**101b**	(*M*)-corinepalenin A	*Coriaria nepalensis*[38]
**102a**	(+)-(11′*R*)-cnidimonin C	*Cnidium monnieri*[39]
**102b**	(–)-(11′*S*)-cnidimonin C	*Cnidium monnieri*[39]
**103a**	(+)-(11′*S*)-cnidimonin B	*Cnidium monnieri*[39]
**103b**	(–)-(11′*R*)-cnidimonin B	*Cnidium monnieri*[39]
**104a**	(+)-spirotriscoumarins A	*Toddalia asiatica*[40]
**104b**	(–)-spirotriscoumarins A	*Toddalia asiatica*[40]
**105a**	(–)-spirotriscoumarins B	*Toddalia asiatica*[40]
**105b**	(+)-spirotriscoumarins B	*Toddalia asiatica*[40]

Table S7. Names, source species and references of simple phenylpropanoid enantiomers

No	Name	Species & Reference
**106a**	(+)-(7*S*,8*S*)-alatusol D	*Eucommia ulmoides*[41]
**106b**	(−)-(7*R*,8*R*)-alatusol D	*Eucommia ulmoides*[41]
**107a**	(−)-(7*S*,8*R*)-alatusol D	*Eucommia ulmoides*[41]
**107b**	(+)-(7*R*,8*S*)-alatusol D	*Eucommia ulmoides*[41]
**108a**	(+)-(*R*)-2,3-dihydroxy-1-(4-hydroxy-3-methoxyphenyl)propan-1-one	*Clausena lansium*[42]
**108b**	(−)-(*S*)-2,3-dihydroxy-1-(4-hydroxy-3-methoxyphenyl)propan-1-one	*Clausena lansium*[42]
**109a**	(−)-(7*R*,8*S*)-7,8-dihydroxy-9-chloro-7-(2,3,5-trimethoxyphenyl)propane	*Acorus tatarinowii*[43]
**109b**	(+)-(7*S*,8*R*)-7,8-dihydroxy-9-chloro-7-(2,3,5-trimethoxyphenyl)propane	*Acorus tatarinowii*[43]
**110a**	(−)-(7*R*,8*R*)-7,8-dihydroxy-9-chloro-7-(2,3,5-trimethoxyphenyl)propane	*Acorus tatarinowii*[43]
**110b**	(+)-(7*S*,8*S*)-7,8-dihydroxy-9-chloro-7-(2,3,5-trimethoxyphenyl)propane	*Acorus tatarinowii*[43]
**111a**	*ent*-acoraminol A	*Acorus tatarinowii*[43]
**111b**	acoraminol A	*Acorus tatarinowii*[44]
**112a**	*ent*-acoraminol C	*Acorus tatarinowii*[44]
**113b**	acoraminol C	*Acorus tatarinowii*[44]
**113a**	*ent*-acoraminol B	*Acorus tatarinowii*[44]
**113b**	acoraminol B	*Acorus tatarinowii*[44]
**114a**	*ent*-acoraminol D	*Acorus tatarinowii*[44]
**114b**	acoraminol D	*Acorus tatarinowii*[44]
**115a**	(−)-*R*-isoacorphenylpropanoid	*Acorus tatarinowii*[44]
**115b**	(+)-*S*-isoacorphenylpropanoid	*Acorus tatarinowii*[44]
**116a**	(−)-crataegusoid E	*Crataegus pinnatifida*[44]
**116b**	(+)-crataegusoid E	*Crataegus pinnatifida*[44]
**117a**	(−)-crataegusoid A	*Crataegus pinnatifida*[44]
**117b**	(+)-crataegusoid A	*Crataegus pinnatifida*[44]
**118a**	(*S*)-3-hydroxy-2-(4-hydroxy-3-methoxyphenyl)-1-(4-hydroxyphenyl)propan-1-one	*Rubus idaeus*[45]
**118b**	(*R*)-3-hydroxy-2-(4-hydroxy-3-methoxyphenyl)-1-(4-hydroxyphenyl)propan-1-one	*Rubus idaeus*[45]
**119a**	(*S*)-3-hydroxy-1,2-bis(4-hydroxy-3-methoxyphenyl)-1-propanone	*Rubus idaeus*[45]
**119b**	(*R*)-3-hydroxy-1,2-bis(4-hydroxy-3-methoxyphenyl)-1-propanone	*Rubus idaeus*[45]
**120a**	(7*S*,8*R*)-2-(4-hydroxy-3methoxyphenyl)-1-(4-hydroxyphenyl)-1-methoxy-propan-3-ol	*Rubus idaeus*[45]
**120b**	(7*R*,8*S*)-2-(4-hydroxy-3methoxyphenyl)-1-(4-hydroxyphenyl)-1-methoxy-propan-3-ol	*Rubus idaeus*[45]
**121a**	(+)-eucophenolic A	*Eucommia ulmoides*[46]
**121b**	(−)-eucophenolic B	*Eucommia ulmoides*[46]
**122a**	(−)-eucophenolic C	*Eucommia ulmoides*[46]
**122b**	(+)-eucophenolic D	*Eucommia ulmoides*[46]
**123a**	(+)-crataegusanoid A	*Crataegus pinnatifida*[47]
**123b**	(−)-crataegusanoid A	*Crataegus pinnatifida*[47]
**124a**	(+)-crataegusanoid B	*Crataegus pinnatifida*[47]
**124b**	(−)-crataegusanoid B	*Crataegus pinnatifida*[47]
**125a**	(+)-crataegusanoid C	*Crataegus pinnatifida*[47]
**125b**	(−)-crataegusanoid C	*Crataegus pinnatifida*[47]
**126a**	(+)-crataegusanoid D	*Crataegus pinnatifida*[47]
**126b**	(−)-crataegusanoid D	*Crataegus pinnatifida*[47]

Table S8. Names, source species and references of indole alkaloid enantiomers

No	Name	Species & Reference
**127a**	(−)-(3*R*)-isatindinoline E	*Isatis tinctoria*[48]
**127b**	(+)-(3*S*)-isatindinoline E	*Isatis tinctoria*[48]
**128a**	(−)-(3*R*)-isatindinoline F	*Isatis tinctoria*[48]
**128b**	(+)-(3*S*)-isatindinoline F	*Isatis tinctoria*[48]
**129a**	(+)-(2′*S*,3′*R*)-clauselansine A	*Clausena lansium*[49]
**129b**	(−)-(2′*R*,3′*S*)-clauselansine A	*Clausena lansium*[49]
**130a**	(+)-(2′*S*,3′*R*)-clauselansine B	*Clausena lansium*[49]
**130b**	(−)-(2′*R*,3′*S*)-clauselansine B	*Clausena lansium*[49]
**131a**	(4*S*)-5,8-dihydroxy-4-(1*H*-indol-3-yl)-3,4-dihydronaphthalen1(2*H*)-one	*Juglans regia*[50]
**131b**	(4*R*)-5,8-dihydroxy-4-(1*H*-indol-3-yl)-3,4-dihydronaphthalen1(2*H*)-one	*Juglans regia*[50]
**132a**	(+)-isatindigotindoline A	*Isatis indigotica*[51]
**132b**	(−)-isatindigotindoline A	*Isatis indigotica*[51]
**133a**	(−)-isatindigotindoline B	*Isatis indigotica*[51]
**133b**	(+)-isatindigotindoline B	*Isatis indigotica*[51]
**134a**	(−)-isatindigotindoline C	*Isatis indigotica*[51]
**134b**	(+)-isatindigotindoline C	*Isatis indigotica*[51]
**135a**	(−)-isatindigotindoline D	*Isatis indigotica*[51]
**135b**	(+)-isatindigotindoline D	*Isatis indigotica*[51]
**136a**	(+)-(3′*R*,2″*S*)-isatisindigoticanine B	*Isatis indigotica*[52]
**136b**	(−)-(3′*R*,2″*S*)-isatisindigoticanine B	*Isatis indigotica*[52]
**137a**	(−)-isatithiopyrin B	*Isatis indigotica*[53]
**137b**	(+)-isatithiopyrin B	*Isatis indigotica*[53]
**138a**	(−)-(2‴*S*,3*S*)-3′-{3″-[2‴-hydroxybut-3‴-en-1‴-yl]-1″,2″,4″-thiadiazol-5″-yl}-5′,6′-dihydrospiro[indoline-3,2′-thiopyran]2-one	*Isatis indigotica*[54]
**138b**	(+)-(2‴*R*,3*R*)-3′-{3″-[2‴-hydroxybut-3‴-en-1‴-yl]-1″,2″,4″-thiadiazol-5″-yl}-5′,6′-dihydrospiro[indoline-3,2′-thiopyran]2-one	*Isatis indigotica*[54]
**139a**	(11c*R*)-5,6,7,11c-tetrahydro-1H-indolizino[7,8-b]indol-3(2H)-one	*Juglans regia*[50]
**139b**	(11c*S*)-5,6,7,11c-tetrahydro-1H-indolizino[7,8-b]indol-3(2H)-one	*Juglans regia*[50]
**140a**	(+)-conodusine E	*Tabernaemontana corymbosa*[55]
**140b**	(−)-ervatamine I	*Ervatamia hainanensis*[56]
**141a**	isatidifoliumindolinone A	*Isatis indigotica*[57]
**141b**	isatidifoliumindolinone B	*Isatis indigotica*[57]
**142a**	isatidifoliumindolinone C	*Isatis indigotica*[57]
**142b**	isatidifoliumindolinone D	*Isatis indigotica*[57]
**143a**	(+)-evodiakine	*Evodia rutaecarpa*[58]
**143b**	(−)-evodiakine	*Evodia rutaecarpa*[58]
**144a**	(−)-uncarilins A	*Uncaria rhynchophylla*[59]
**144b**	(+)-uncarilins A	*Uncaria rhynchophylla*[59]
**145a**	(−)-uncarilins A	*Uncaria rhynchophylla*[59]
**145b**	(+)-uncarilins A	*Uncaria rhynchophylla*[59]
**146a**	(−)-(2*R*,3*R*)-3-Hydroxy-2H-pyrrolo[2,3-*b*]indolo[5,5a,6-*b*,*a*]quinazoline-9(8H),7′-dione	*Isatis indigotica*[60]
**146b**	(+)-(2*S*,3*S*)-3-Hydroxy-2H-pyrrolo[2,3-*b*]indolo[5,5a,6-*b*,*a*]quinazoline-9(8H),7′-dione	*Isatis indigotica*[60]

Table S9. Names, source species and references of quinoline and isoquinoline alkaloid enantiomers

No	Name	Species & Reference
**147a**	(+)-zanthonitidine A	*Zanthoxylum nitidum*[61]
**147b**	(−)-zanthonitidine A	*Zanthoxylum nitidum*[61]
**148a**	(+)-(3*R*)-isatindinoline D	*Isatis tinctoria*[48]
**148b**	(−)-(3*S*)-isatindinoline D	*Isatis tinctoria*[48]
**149a**	(−)-(3*R*)-isatindinoline C	*Isatis tinctoria*[48]
**149b**	(+)-(3*S*)-isatindinoline C	*Isatis tinctoria*[48]
**150a**	(−)-(10*S*,14*S*)-isatindinoline A	*Isatis tinctoria*[48]
**150b**	(+)-(10*R*,14*R*)-isatindinoline A	*Isatis tinctoria*[48]
**151a**	(−)-(4*S*,2′*R*,3′*R*)-isatisindigoticanine D	*Isatis indigotica*[52]
**151b**	(+)-(4*R*,2′*S*,3′*S*)-isatisindigoticanine D	*Isatis indigotica*[52]
**152a**	isatindigoticoic acid A	*Isatis indigotica*[62]
**152b**	epiisatindigoticoic acid A	*Isatis indigotica*[62]
**153a**	(+)-mucroniferanine A	*Corydalis mucronifera*[63]
**153b**	(−)-mucroniferanine A	*Corydalis mucronifera*[63]
**154a**	(+)-hendersine A	*Corydalis hendersonii*[64]
**154b**	(−)-hendersine A	*Corydalis hendersonii*[64]
**155a**	(+)-mucroniferanine B	*Corydalis mucronifera*[63]
**155b**	(−)-mucroniferanine B	*Corydalis mucronifera*[63]
**156a**	(+)-mucroniferanine C	*Corydalis mucronifera*[63]
**156b**	(−)-mucroniferanine C	*Corydalis mucronifera*[63]
**157a**	(+)-mucroniferanine D	*Corydalis mucronifera*[63]
**157b**	(−)-mucroniferanine D	*Corydalis mucronifera*[63]
**158a**	(+)-mucroniferanine E	*Corydalis mucronifera*[63]
**158b**	(−)-mucroniferanine E	*Corydalis mucronifera*[63]
**159a**	(−)-macleayin D	*Macleaya cordata*[65]
**159b**	(+)-macleayin D	*Macleaya cordata*[65]
**160a**	(−)-macleayin C	*Macleaya cordata*[65]
**160b**	(+)-macleayin C	*Macleaya cordata*[65]
**161a**	(−)-macleayin E	*Macleaya cordata*[65]
**161b**	(+)-macleayin E	*Macleaya cordata*[65]
**162a**	(−)-6-acetonyldihydrosanguinarine	*Macleaya cordata*[65]
**162b**	(+)-6-acetonyldihydrosanguinarine	*Macleaya cordata*[65]
**163a**	(−)-6-acetonyldihydrochelerythrine	*Macleaya cordata*[65]
**163b**	(+)-6-acetonyldihydrochelerythrine	*Macleaya cordata*[65]
**164**	(±)-6-methoxyldihydrosanguinarine	*Macleaya cordata*[65]
**165a**	ambidalmine A1	*Corydalis ambigua*[66]
**165b**	ambidalmine A2	*Corydalis ambigua*[66]
**166a**	ambidalmine B1	*Corydalis ambigua*[66]
**166b**	ambidalmine B2	*Corydalis ambigua*[66]
**167**	(±)-ambidalmine C	*Corydalis ambigua*[66]
**168**	(±)-ambidalmine D	*Corydalis ambigua*[66]
**169**	(±)-ambidalmine E	*Corydalis ambigua*[66]
**170a**	(+)-5-hydroxyl-8-oxyberberine	*Coptis chinensis*[67]
**170b**	(−)-5-hydroxyl-8-oxyberberine	*Coptis chinensis*[67]
**171a**	(+)-macleayins A	*Macleaya cordata*[68]
**171b**	(−)-macleayins A	*Macleaya cordata*[68]
**172a**	(+)-macleayins B	*Macleaya cordata*[68]
**172b**	(−)-macleayinsB	*Macleaya cordata*[68]
**173a**	ambidimerine F1	*Corydalis ambigu*[66]
**173b**	ambidimerine F2	*Corydalis ambigu*[66]

Table S10. Names, source species and references of *β*-carboline and carbazole alkaloid enantiomers

No	Name	Species & Reference
**174a**	(+)-kumudine A	*Picrasma quassioides*[69]
**174b**	(−)-kumudine A	*Picrasma quassioides*[69]
**175a**	(+)-kumudine B	*Picrasma quassioides*[69]
**175b**	(−)-kumudine B	*Picrasma quassioides*[69]
**176a**	(+)-kumudine C	*Picrasma quassioides*[69]
**176b**	(−)-kumudine C	*Picrasma quassioides*[69]
**177a**	(+)-kumudine D	*Picrasma quassioides*[69]
**177b**	(−)-kumudine D	*Picrasma quassioides*[69]
**178a**	(+)-*S*-quassidine K	*Picrasma quassioides*[70]
**178b**	(−)-*R*-quassidine K	*Picrasma quassioides*[70]
**179a**	(+)-*S*-quassidine I	*Picrasma quassioides*[71]
**179b**	(−)-*R*-quassidine I	*Picrasma quassioides*[71]
**180a**	(+)-*S*-quassidine J	*Picrasma quassioides*[71]
**180b**	(−)-*R*-quassidine J	*Picrasma quassioides*[71]`
**181a**	(−)-peharmaline A	*Peganum harmala*[72]
**181b**	(+)-peharmaline A	*Peganum harmala*[72]
**182**	mitralactonal B	*Pausinystalia yohimbe*[73]
**183**	mitralactonal C	*Pausinystalia yohimbe*[73]
**184a**	(−)-dunnine A	*Clausena dunniana*[74]
**184b**	(+)-dunnine A	*Clausena dunniana*[74]
**185a**	(−)-dunnine B	*Clausena dunniana*[74]
**185b**	(+)-dunnine B	*Clausena dunniana*[74]
**186a**	(−)-clausenawalline A	*Clausena dunniana*[74]
**186b**	(+)-clausenawalline A	*Clausena dunniana*[74]
**187a**	(−)-dunnine C	*Clausena dunniana*[74]
**187b**	(+)-dunnine C	*Clausena dunniana*[74]
**188a**	(1′*R*,2′*R*)-dunnine D	*Clausena dunniana*[74]
**188b**	(1′*S*,2′*S*)-dunnine D	*Clausena dunniana*[74]
**189a**	(−)-microphyline F	*Murraya microphylla*[75]
**189b**	(+)-microphyline F	*Murraya microphylla*[75]
**190a**	(−)-microphyline D	*Murraya microphylla*[75]
**190b**	(+)-microphyline D	*Murraya microphylla*[75]
**191a**	(−)-microphyline E	*Murraya microphylla*[75]
**191b**	(+)-microphyline E	*Murraya microphylla*[75]
**192a**	(2′*S*,3′*R*)-microphyline K	*Murraya microphylla*[76]
**192b**	(2′*R*,3′*S*)-microphyline K	*Murraya microphylla*[76]

Table S11. Names, source species and references of piperidine alkaloid enantiomers

No	Name	Species & Reference
**193a**	(+)-pyracyclumine A	*Anacyclus pyrethrum*[77]
**193b**	(–)-pyracyclumine A	*Anacyclus pyrethrum*[77]
**194a**	(+)-pyracyclumine B	*Anacyclus pyrethrum*[77]
**194b**	(–)-pyracyclumine B	*Anacyclus pyrethrum*[77]
**195a**	(+)-pyracyclumine C	*Anacyclus pyrethrum*[77]
**195a**	(–)-pyracyclumine C	*Anacyclus pyrethrum*[77]
**196a**	(+)-pyracyclumine D	*Anacyclus pyrethrum*[77]
**196b**	(–)-pyracyclumine D	*Anacyclus pyrethrum*[77]
**197a**	(+)-pyracyclumine E	*Anacyclus pyrethrum*[77]
**197b**	(–)-pyracyclumine E	*Anacyclus pyrethrum*[77]
**198a**	(+)-tishaviolamine C	*Viola tianschanica*[78]
**198b**	(–)-tishaviolamine C	*Viola tianschanica*[78]
**199a**	(+)-tishaviolamine E	*Viola tianschanica*[78]
**199b**	(–)-tishaviolamine E	*Viola tianschanica*[78]
**200a**	(+)-tishaviolamine D	*Viola tianschanica*[78]
**200b**	(–)-tishaviolamine D	*Viola tianschanica*[78]
**201a**	(+)-tishaviolamine A	*Viola tianschanica*[78]
**201b**	(–)-tishaviolamine A	*Viola tianschanica*[78]
**202a**	(+)-tishaviolamine B	*Viola tianschanica*[78]
**202a**	(–)-tishaviolamine B	*Viola tianschanica*[78]
**203a**	(+)-tishaviolamine F	*Viola tianschanica*[78]
**203b**	(–)-tishaviolamine F	*Viola tianschanica*[78]
**204a**	(+)-tishaviolamine G	*Viola tianschanica*[78]
**204b**	(–)-tishaviolamine G	*Viola tianschanica*[78]
**205a**	(+)-tishaviolamine H	*Viola tianschanica*[78]
**205b**	(–)-tishaviolamine H	*Viola tianschanica*[78]
**206a**	(+)-claulansamide A	*Clausena lansium*[79]
**206b**	(–)-claulansamide A	*Clausena lansium*[79]
**207**	(±)-claulansamide B	*Clausena lansium*[79]

Table S12. Names, source species and references of thiohydantoin alkaloid enantiomers

No	Name	Species & Reference
**208a**	(+)-macathiohydantoin A	*Lepidium meyenii*[80]
**208b**	(−)-macathiohydantoin A	*Lepidium meyenii*[80]
**209a**	(+)-macathiohydantoin B	*Lepidium meyenii*[80]
**209b**	(−)-macathiohydantoin B	*Lepidium meyenii*[80]
**210a**	(+)-macathiohydantoin C	*Lepidium meyenii*[80]
**210b**	(−)-macathiohydantoin C	*Lepidium meyenii*[80]
**211a**	(+)-macathiohydantoin D	*Lepidium meyenii*[80]
**211b**	(−)-macathiohydantoin D	*Lepidium meyenii*[80]
**212a**	(+)-macathiohydantoin E	*Lepidium meyenii*[80]
**212b**	(−)-macathiohydantoin E	*Lepidium meyenii*[80]
**213a**	(+)-macathiohydantoin F	*Lepidium meyenii*[80]
**213b**	(−)-macathiohydantoin F	*Lepidium meyenii*[80]
**214a**	(+)-macathiohydantoin G	*Lepidium meyenii*[80]
**214b**	(−)-macathiohydantoin G	*Lepidium meyenii*[80]
**215a**	(+)-macathiohydantoin H	*Lepidium meyenii*[80]
**215b**	(−)-macathiohydantoin H	*Lepidium meyenii*[80]
**216a**	(+)-macathiohydantoin I	*Lepidium meyenii*[80]
**216b**	(−)-macathiohydantoin I	*Lepidium meyenii*[80]
**217a**	(+)-macathiohydantoin J	*Lepidium meyenii*[80]
**217b**	(−)-macathiohydantoin J	*Lepidium meyenii*[80]
**218a**	(+)-macathiohydantoin K	*Lepidium meyenii*[80]
**218b**	(−)-macathiohydantoin K	*Lepidium meyenii*[80]

Table S13. Names, source species and references of indolizidine and quinolizidine alkaloid enantiomers

No	Name	Species & Reference
**219**	tengechlorenine	*Ficus fistulosa var. tengerensis*[81]
**220a**	(+)-tylophorine	*Tylophora indica*[82]
**220b**	(−)-tylophorine	*Tylophora indica*[82]
**221a**	(+)-homocrepidine A	*Dendrobium crepidatum*[83]
**221b**	(−)-homocrepidine A	*Dendrobium crepidatum*[83]
**222a**	(+)-tengerensine	*Ficus fistulosa var. tengerensis*[81]
**222b**	(−)-tengerensine	*Ficus fistulosa var. tengerensis*[81]
**223a**	(+)-2-episecurinol A	*Flueggea virosa*[84]
**223b**	(−)-2-episecurinol A	*Flueggea virosa*[84]
**224a**	(+)-virosine B	*Flueggea virosa*[84]
**224b**	(−)-virosine B	*Flueggea virosa*[84]
**225a**	virosine A	*Flueggea virosa*[84]
**225b**	secu′amamine	*Flueggea virosa*[84]
**226a**	(+)-securinol A	*Flueggea virosa*[84]
**226b**	(−)-securinol A	*Flueggea virosa*[84]

Table S14. Names, source species and references of other alkaloid enantiomers

No	Name	Species & Reference
**227a**	(*R*)-vasicinolone	*Peganum harmala* [85]
**227b**	(*S*)-vasicinolone	*Peganum harmala* [85]
**228a**	(−)-(2′*R*)-isatisindigoticanine C	*Isatis indigotica* [52]
**228b**	(+)-(2′*S*)-isatisindigoticanine C	*Isatis indigotica* [52]
**229a**	phaitanthrin A	*Isatis indigotica* [62]
**229b**	epiphaitanthrin A	*Isatis indigotica* [62]
**230a**	(−)-peganumine E	*Peganum harmala* [86]
**230b**	(+)-peganumine E	*Peganum harmala*[86]
**231a**	(4*R*)-4-(6-amino-9H-purin-9-yl)-5,8-dihydroxy-3,4-dihydronap hthalen-1(2*H*)-one	*Juglans regia*[50]
**231b**	(4*S*)-4-(6-amino-9H-purin-9-yl)-5,8-dihydroxy-3,4-dihydronap hthalen-1(2*H*)-one	*Juglans regia*[50]
**232a**	(4*R*)-4-(6-amino-9H-purin-9-yl)-5-hydroxy-3,4-dihydronaphtha len-1(2*H*)-one	*Juglans regia*[50]
**232b**	(4*S*)-4-(6-amino-9H-purin-9-yl)-5-hydroxy-3,4-dihydronaphtha len-1(2*H*)-one	*Juglans regia*[50]
**233a**	(*S*)-parvifloranines A	*Geijera parviflora*[87]
**233b**	(*R*)-parvifloranines A	*Geijera parviflora*[87]
**234**	parvifloranine B	*Geijera parviflora*[87]
**235a**	(2*R*)-9-Hydroxy-5-oxo-2,3,4,5-tetrahydro-1H-benzo[*b*]azepine-2carboxamide	*Juglans regia*[50]
**235b**	(2*S*)-9-Hydroxy-5-oxo-2,3,4,5-tetrahydro-1H-benzo[*b*]azepine-2carboxamide	*Juglans regia*[50]
**236a**	(+)-peganumine I	*Peganum harmala*[86]
**236b**	(−)-peganumine I	*Peganum harmala*[86]
**237a**	isoepigoitrin	*Isatis indigotica*[53]
**237b**	isogoitrin	*Isatis indigotica*[53]
**238a**	(+)-(3*S*,4*R*,5*S*,6*S*)-clauselansine C	*Clausena lansium*[49]
**238b**	(−)-(3*R*,4*S*,5*R*,6*R*)-clauselansine C	*Clausena lansium*[49]
**239a**	(+)-phaeocaulin A	*Curcuma phaeocaulis*[88]
**239b**	(−)-phaeocaulin A	*Curcuma phaeocaulis*[88]
**240a**	(+)-phaeocaulin B	*Curcuma phaeocaulis*[88]
**240b**	(−)-phaeocaulin B	*Curcuma phaeocaulis*[88]
**241a**	scumoniline A	*Scutellaria moniliorrhiza*[89]
**241b**	scumoniline B	*Scutellaria moniliorrhiza*[89]
**242a**	scumoniline C	*Scutellaria moniliorrhiza*[89]
**242b**	scumoniline D	*Scutellaria moniliorrhiza*[89]
**243a**	(a*S*)-bletillatin B	*Bletilla striata*[90]
**243b**	(a*R*)-bletillatin B	*Bletilla striata*[90]
**244a**	(2*R*)-juglanaloid A	*Juglans mandshurica*[91]
**244b**	(2*S*)-juglanaloid A.	*Juglans mandshurica*[91]
**245a**	(2*R*)-juglanaloid B	*Juglans mandshurica*[91]
**245b**	(2*S*)-juglanaloid B.	*Juglans mandshurica*[91]
**246a**	(−)-(4*S*)-isatindinoline B	*Isatis tinctoria*[48]
**246b**	(+)-(4*R*)-isatindinoline B	*Isatis tinctoria*[48]
**247a**	(2*S*,3*S*,*E*)-3-[2,3-dihydro-2-(4-hydroxy-3,5-dimethoxyphenyl)-3-(hydroxymethyl)-1,4-benzodioxin-6yl]-*N*-[2-(4-hydroxyphen ethyl)ethyl]-2-propenamide	*Lycium chinense*[92]
**247b**	(2*R*,3*R*,*E*)-3-[2,3-dihydro-2-(4-hydroxy-3,5-dimethoxyphenyl)-3-(hydroxymethyl)-1,4-benzodioxin-6yl]-*N*-[2-(4-hydroxyphen ethyl)ethyl]-2-propenamide	*Lycium chinense*[92]
**248a**	(+)-sativamides A	*Cannabis sativa*[93]
**248b**	(−)-sativamides A	*Cannabis sativa*[93]
**249a**	(+)-sativamides B	*Cannabis sativa*[93]
**249b**	(−)-sativamides B	*Cannabis sativa*[93]

No	Name	Species & Reference
**250**	kingianins O	*Endiandra kingiana*[94]
**251**	kingianins P	*Endiandra kingiana*[94]
**252**	kingianins Q	*Endiandra kingiana*[94]

Table S15. Names, source species and references of flavone and isoflavone enantiomers

No	Name	Species & Reference
**253a**	(+)-trichocladabiflavone A	*Selaginella trichoclada*[95]
**253b**	(−)-trichocladabiflavone A	*Selaginella trichoclada*[95]
**254a**	(+)-(11*S*)-cnidimonins A	*Cnidium monnieri*[39]
**254b**	(−)-(11*R*)-cnidimonins A	*Cnidium monnieri*[39]
**255a**	(−)-uncariol C	*Uncaria rhynchophylla*[96]
**255b**	(+)-uncariol C	*Uncaria rhynchophylla*[96-97]
**256a**	(−)-uncariol D	*Uncaria rhynchophylla*[96-97]
**256b**	(+)-uncariol D	*Uncaria rhynchophylla*[96-97]
**257a**	(2*S*)-6-formyl-5,7-dihydroxyflavanone	*Eugenia rigida*[98]
**257b**	(2*R*)-6-formyl-5,7-dihydroxyflavanone	*Eugenia rigida*[98]
**258a**	(+)-(2*R*)-6-propionyloxyethyl-4′,5,7-trihydroxyisoflavanone	*Abrus precatorius*[99]
**258b**	(−)-(2*S*)-6-propionyloxyethyl-4′,5,7-trihydroxyisoflavanone	*Abrus precatorius*[99]
**259a**	(+)-(2*R*)-6-propionyloxymethyl-4′,,5,7-trihydroxyisoflavanone	*Abrus precatorius*[99]
**259b**	(−)-(2*S*)-6-propionyloxymethyl-4′,,5,7-trihydroxyisoflavanone	*Abrus precatorius*[99]
**260a**	(+)-cajanusflavanol C	*Cajanus cajan*[100]
**260b**	(−)-cajanusflavanol C	*Cajanus cajan*[100]
**261a**	(+)-cajanusflavanol A	*Cajanus cajan*[100]
**261b**	(−)-cajanusflavanol A	*Cajanus cajan*[100]
**262a**	(+)-cajanusflavanol B	*Cajanus cajan*[100]
**262b**	(−)-cajanusflavanol B	*Cajanus cajan*[100]
**263a**	nigranol C-a	*Morus nigra*[101]
**263b**	nigranol C-b	*Morus nigra*[101]
**264a**	nigragenon E-a	*Morus nigra*[101]
**264b**	nigragenon E-b	*Morus nigra*[101]
**265**	6,7,3′-trimethoxy-4′-hydroxyflavan	*Celastrus hindsii*[102]
**266**	6,7-dimethoxy-3′,4′-dihydroxyflavan	*Celastrus hindsii*[102]
**267a**	(2*S*,2′′′*R*)-daphnegiralin A	*Daphne giraldii*[103]
**267b**	(2*R*,2′′′*S*)-daphnegiralin A	*Daphne giraldii*[103]
**268a**	(2*S*,2′′′*S*)-daphnegiralin A	*Daphne giraldii*[103]
**268b**	(2*R*,2′′′*R*)-daphnegiralin A	*Daphne giraldii*[103]
**269a**	(2*S*,2′′′*R*)-daphnegiralin B	*Daphne giraldii*[103]
**269b**	(2*R*,2′′′*S*)-daphnegiralin B	*Daphne giraldii*[103]
**270a**	(2*S*,2′′′*S*)-daphnegiralin B	*Daphne giraldii*[103]
**270b**	(2*R*,2′′′*R*)-daphnegiralin B	*Daphne giraldii*[103]
**271a**	(−)-cochinchinenin K	*Dracaena cochinchinensis*[104]
**271b**	(+)-cochinchinenin K	*Dracaena cochinchinensis*[104]
**272a**	(+)-cudraisoflavone U	*Maclura tricuspidata*[105]
**272b**	(−)-cudraisoflavone U	*Maclura tricuspidata*[105]
**273a**	(−)-epi-cudraisoflavone U	*Maclura tricuspidata*[105]
**273b**	(+)-epi-cudraisoflavone U	*Maclura tricuspidata*[105]
**274a**	(+)-cudraisoflavone V	*Maclura tricuspidata*[105]
**274b**	(−)-cudraisoflavone V	*Maclura tricuspidata*[105]
**275a**	(+)-epi-cudraisoflavone V	*Maclura tricuspidata*[105]
**275b**	(−)-epi-cudraisoflavone V	*Maclura tricuspidata*[105]
**276a**	(+)-cudraisoflavone W	*Maclura tricuspidata*[105]
**276b**	(−)-cudraisoflavone W	*Maclura tricuspidata*[105]
**277a**	(+)-epi-cudraisoflavone W	*Maclura tricuspidata*[105]
**277b**	(−)-epi-cudraisoflavone W	*Maclura tricuspidata*[105]
**278a**	(+)-cudraisoflavone X	*Maclura tricuspidata*[105]
**278b**	(−)-cudraisoflavone X	*Maclura tricuspidata*[105]
**279a**	pisonone A	*Pisonia umbellifera*[106]
**279b**	pisonone B	*Pisonia umbellifera*[106]
**280a**	pisonone C	*Pisonia umbellifera*[106]

No	Name	Species & Reference
**280b**	pisonone D	*Pisonia umbellifera*[106]

Table S16. Names, source species and references of chalcone enantiomers

No	Name	Species & Reference
**281a**	(+)-(*βR*)-3′,4-dihydroy-3,4′-dimethoxy-dihydrochalcone	*Pteris ensiformis*[107]
**281b**	(−)-(*βS*)-3′,4-dihydroy-3,4′-dimethoxy-dihydrochalcone	*Pteris ensiformis*[107]
**282a**	(+)-oxyfadichalcone F	*Oxytropis chiliophylla*[108]
**282b**	(−)-oxyfadichalcone F	*Oxytropis chiliophylla*[108]
**283a**	(−)-oxyfadichalcone C	*Oxytropis chiliophylla*[108]
**283b**	(+)-oxyfadichalcone C	*Oxytropis chiliophylla*[108]
**284a**	(−)-oxyfadichalcone G	*Oxytropis chiliophylla*[108]
**284b**	(+)-oxyfadichalcone G	*Oxytropis chiliophylla*[108]
**285a**	(+)-balsacone J	*Populus balsamifera*[109]
**285b**	(−)-balsacone J	*Populus balsamifera*[109]
**286a**	(+)-iryantherin D	*Populus balsamifera*[109]
**286b**	(−)-iryantherin D	*Populus balsamifera*[109]
**287a**	(+)-balsacone K	*Populus balsamifera*[109]
**287b**	(−)-balsacone K	*Populus balsamifera*[109]
**288a**	(+)-balsacone L	*Populus balsamifera*[109]
**288b**	(−)-balsacone L	*Populus balsamifera*[109]
**289**	balsacone M	*Populus balsamifera*[109]
**290a**	(−)-cochinchinenin I	*Dracaena cochinchinensis*[104]
**290b**	(+)-cochinchinenin I	*Dracaena cochinchinensis*[104]
**291a**	(−)-cochinchinenin K	*Dracaena cochinchinensis*[104]
**291b**	(+)-cochinchinenin K	*Dracaena cochinchinensis*[104]
**292**	horsfiequinone B	*Horsfieldia tetratepala*[110]
**293**	horsfiequinone C	*Horsfieldia tetratepala*[110]
**294**	horsfiequinone D	*Horsfieldia tetratepala*[110]
**295**	horsfiequinone E	*Horsfieldia tetratepala*[110]
**296**	horsfiequinone F	*Horsfieldia tetratepala*[110]

Table S17. Names, source species and references of xanthone enantiomers

No	Name	Species & Reference
**297a**	(+)-cracochinxanthone A	*Cratoxylum cochinchinense*[111]
**297b**	(−)-cracochinxanthone A	*Cratoxylum cochinchinense*[111]
**298a**	(4a*S*,9a*R*,2Z,2′*E*)-valderramenol A	*Uvaria valderramensis*[112]
**298b**	(4a*R*,9a*S*,2Z,2′*E*)-valderramenol A	*Uvaria valderramensis*[112]
**299a**	(4a*S*,9a*R*,2Z,2′*E*)-valderramenol B	*Uvaria valderramensis*[112]
**299b**	(4a*R*,9a*S*,2Z,2′*E*)-valderramenol B	*Uvaria valderramensis*[112]
**300a**	(+)-pruniflorone T	*Cratoxylum formosum*[113]
**300b**	(−)-pruniflorone T	*Cratoxylum formosum*[113]
**301a**	(+)-cochinchinone C	*Cratoxylum formosum*[113]
**301b**	(−)-cochinchinone C	*Cratoxylum formosum*[113]
**302a**	(+)-pruniflorone U	*Cratoxylum formosum*[113]
**302b**	(−)-pruniflorone U	*Cratoxylum formosum*[113]
**303a**	(−)-epiisobractatin	*Garcinia bracteata*[114]
**303b**	(+)-epiisobractatin	*Garcinia bracteata*[114]
**304a**	(−)-13-hydroxyisobractatin	*Garcinia bracteata*[114]
**304b**	(+)-13-hydroxyisobractatin	*Garcinia bracteata*[114]
**305a**	(−)-13-hydroxyepiisobractatin	*Garcinia bracteata*[114]
**305b**	(+)-13-hydroxyepiisobractatin	*Garcinia bracteata*[114]
**306a**	(−)-8-methoxy-8,8a-dihydrobractatin	*Garcinia bracteata*[114]
**306b**	(+)-8-methoxy-8,8a-dihydrobractatin	*Garcinia bracteata*[114]
**307a**	(−)-8-Ethoxy-8,8a-dihydrobractat	*Garcinia bracteata*[114]
**307b**	(+)-8-Ethoxy-8,8a-dihydrobractat	*Garcinia bracteata*[114]
**308a**	(+)-doitunggarcinone F	*Garcinia propinqua*[115]
**308b**	(−)-doitunggarcinone F	*Garcinia propinqua*[115]
**309a**	(+)-doitunggarcinone I	*Garcinia propinqua*[115]
**309b**	(−)-doitunggarcinone I	*Garcinia propinqua*[115]
**310a**	(+)-doitunggarcinone G	*Garcinia propinqua*[115]
**310b**	(−)-doitunggarcinone G	*Garcinia propinqua*[115]
**311a**	(+)-doitunggarcinone H	*Garcinia propinqua*[115]
**311b**	(−)-doitunggarcinone H	*Garcinia propinqua*[115]
**312a**	(+)-doitunggarcinone J	*Garcinia propinqua*[115]
**312b**	(−)-doitunggarcinone J	*Garcinia propinqua*[115]
**313a**	(+)-doitunggarcinone E	*Garcinia propinqua*[115]
**313b**	(−)-doitunggarcinone E	*Garcinia propinqua*[115]

Table S18. Names, source species and references of sesquiterpenoid enantiomers

No	Name	Species & Reference
**314a**	(−)-8*R*-artaboterpenoid B	*Artabotrys hexapetalus*[116]
**314b**	(+)-8*S*-artaboterpenoid B	*Artabotrys hexapetalus*[116]
**315a**	(+)-(3*S*,4*S*)-eucomegastigmane B	*Eucommia ulmoides*[117]
**315b**	(−)-(3*R*,4*R*)-eucomegastigmane B	*Eucommia ulmoides*[117]
**316a**	(+)-(6*R*,9*S*)-blumenol C	*Eucommia ulmoides*[117]
**316b**	(−)-(6*S*,9*R*)-blumenol C	*Eucommia ulmoides*[117]
**317a**	(+)-(6*R*,9*R*)-blumenol C	*Eucommia ulmoides*[117]
**317b**	(−)-(6*S*,9*S*)-blumenol C	*Eucommia ulmoides*[117]
**318a**	(+)-phaeocaulin C	*Curcuma phaeocaulis*[88]
**318b**	(−)-phaeocaulin C	*Curcuma phaeocaulis*[88]
**319a**	(+)-phaeocaulin D	*Curcuma phaeocaulis*[88]
**319b**	(−)-phaeocaulin D	*Curcuma phaeocaulis*[88]
**320a**	(−)-2,9-humuladien-6-ol-8-one	*Syringa pinnatifolia*[118]
**320b**	(+)-2,9-humuladien-6-ol-8-one	*Syringa pinnatifolia*[118]
**321a**	(+)-alashanoid C	*Syringa pinnatifolia*[118]
**321b**	(−)-alashanoid C	*Syringa pinnatifolia*[118]
**322a**	(−)-alashanoid D	*Syringa pinnatifolia*[118]
**322b**	(+)-alashanoid D	*Syringa pinnatifolia*[118]
**323a**	(+)-alashanoid E	*Syringa pinnatifolia*[118]
**323b**	(−)-alashanoid E	*Syringa pinnatifolia*[118]
**324a**	(+)-alashanoid F	*Syringa pinnatifolia*[118]
**324b**	(−)-alashanoid F	*Syringa pinnatifolia*[118]
**325a**	(+)-(6*R*)-eucomegastigmane A	*Eucommia ulmoides*[117]
**325b**	(−)-(6*S*)-eucomegastigmane A	*Eucommia ulmoides*[117]
**326a**	(+)-commyrrin A	*Commiphora myrrha*[119]
**326b**	(−)-commyrrin A	*Commiphora myrrha*[119]
**327a**	(+)-4-hydroxy-10-epirotundone	*Daphne genkwa*[120]
**327b**	(−)-4-hydroxy-10-epirotundone	*Daphne genkwa*[120]
**328**	4*β*,10*β*-dihydroxy-1*β*,5*β*-*H*-guaia-6-en	*Kadsura interior*[121]
**329a**	(+)-alashanoid A	*Syringa pinnatifolia*[118]
**329b**	(−)-alashanoid A	*Syringa pinnatifolia*[118]

Table S19. Names, source species and references of diterpenoid enantiomers

No	Name	Species & Reference
**330a**	(+)-mangelonoid A	*Croton mangelong*[122]
**330b**	(−)-mangelonoid A	*Croton mangelong*[122]
**331a**	(+)-mangelonoid B	*Croton mangelong*[122]
**331b**	(−)-mangelonoid B	*Croton mangelong*[122]
**332a**	(+)-cryptomeriolide	*Cryptomeria fortune*[123]
**332b**	(−)-cryptomeriolide	*Cryptomeria fortune*[123]
**333a**	miltiolactone A	*Salvia miltiorrhiza*[124]
**333b**	miltiolactone B	*Salvia miltiorrhiza*[124]
**334a**	(2*S*,11*S*)-salviaprione	*Salvia prionitis*[125]
**334b**	(2*R*,11*R*)-salviaprione	*Salvia prionitis*[125]
**335a**	(+)-sweriledugenin A	*Swertia leducii*[126]
**335b**	(−)-sweriledugenin A	*Swertia leducii*[126]
**336a**	(7*R*,8*R*,9*S*)-paeoveitol	*Paeonia veitchii*[127]
**336b**	(7*S*,8*S*,9*R*)-paeoveitol	*Paeonia veitchii*[127]
**337a**	(+)-japonone A	*Hypericum japonicum*[128]
**337b**	(−)-japonone A	*Hypericum japonicum*[128]
**338a**	(+)-japonone B	*Hypericum japonicum*[128]
**338b**	(−)-japonone B	*Hypericum japonicum*[128]

Table S20. Names, source species and references of meroterpenoid enantiomers

No	Name	Species & Reference
**339a**	(−)-nyingchinoid A	*Rhododendron nyingchiense*[129]
**339b**	(+)-nyingchinoid A	*Rhododendron nyingchiense*[129]
**340a**	(+)-nyingchinoid B	*Rhododendron nyingchiense*[129]
**340b**	(−)-nyingchinoid B	*Rhododendron nyingchiense*[129]
**341a**	(−)-nyingchinoid C	*Rhododendron nyingchiense*[129]
**341b**	(+)-nyingchinoid C	*Rhododendron nyingchiense*[129]
**342a**	(−)-nyingchinoid D	*Rhododendron nyingchiense*[129]
**342b**	(+)-nyingchinoid D	*Rhododendron nyingchiense*[129]
**343a**	(+)-nyingchinoid E	*Rhododendron nyingchiense*[129]
**343b**	(−)-nyingchinoid E	*Rhododendron nyingchiense*[129]
**344a**	(−)-nyingchinoid H	*Rhododendron nyingchiense*[129]
**344b**	(+)-nyingchinoid H	*Rhododendron nyingchiense*[129]
**345a**	(+)-nyingchinoid F	*Rhododendron nyingchiense*[129]
**345b**	(−)-nyingchinoid F	*Rhododendron nyingchiense*[129]
**346a**	(+)-nyingchinoid G	*Rhododendron nyingchiense*[129]
**346b**	(−)-nyingchinoid G	*Rhododendron nyingchiense*[129]
**347a**	(−)-rhodonoid G	*Rhododendron capitatum*[130]
**347b**	(+)-rhodonoid G	*Rhododendron capitatum*[130]
**348a**	(+)-rhodonoid C	*Rhododendron capitatum*[130]
**348b**	(−)-rhodonoid C	*Rhododendron capitatum*[130]
**349a**	(−)-rhodonoid D	*Rhododendron capitatum*[130]
**349b**	(+)-rhodonoid D	*Rhododendron capitatum*[130]
**350a**	(−)-rhodonoid A	*Rhododendron capitatum*[131]
**350b**	(+)-rhodonoid A	*Rhododendron capitatum*[131]
**351a**	(+)-rhodonoid E	*Rhododendron capitatum*[130]
**351b**	(−)-rhodonoid E	*Rhododendron capitatum*[130]
**352a**	(+)-rhodonoid F	*Rhododendron capitatum*[130]
**352b**	(−)-rhodonoid F	*Rhododendron capitatum*[130]
**353a**	(−)-rhodonoid B	*Rhododendron capitatum*[131]
**353b**	(+)-rhodonoid B	*Rhododendron capitatum*[131]
**354a**	(1*S*,2*R*,3*S*,4*R*)- rasumatranin A	*Radula sumatrana*[132]
**354b**	(1*R*,2*S*,3*R*,4*S*)- rasumatranin A	*Radula sumatrana*[132]
**355a**	(1*S*,2*S*,3*S*,4*R*)- rasumatranin C	*Radula sumatrana*[132]
**355b**	(1*R*,2*R*,3*R*,4*S*)- rasumatranin C	*Radula sumatrana*[132]
**356a**	(1*S*,2*S*,3*S*,4*R*)- rasumatranin B	*Radula sumatrana*[132]
**356b**	(1*R*,2*R*,3*R*,4*S*)- rasumatranin B	*Radula sumatrana*[132]
**357a**	(1*S*,2*R*,3*S*,7*S*)- rasumatranin D	*Radula sumatrana*[132]
**357b**	(1*R*,2*S*,3*R*,7*R*)- rasumatranin D	*Radula sumatrana*[132]
**358**	(±)-radulanin M	*Radula sumatrana*[132]
**359**	(±)-radulanin N	*Radula sumatrana*[132]
**360a**	(1*S*,2*S*,3*R*,7*S*)-bibenzyl/o-cannabicyclol hybrid	*Radula sumatrana*[132]
**360b**	(1*R*,2*R*,3*S*,7*R*)-bibenzyl/o-cannabicyclol hybrid	*Radula sumatrana*[132]
**361a**	(+)-magmenthane A	*Magnolia officinalis*[133]
**361b**	(−)-magmenthane A	*Magnolia officinalis*[133]
**362a**	(+)-magmenthane B	*Magnolia officinalis*[133]
**362b**	(−)-magmenthane B	*Magnolia officinalis*[133]
**363a**	(+)-magmenthane C	*Magnolia officinalis*[133]
**363b**	(−)-magmenthane C	*Magnolia officinalis*[133]
**364a**	(+)-magmenthane D	*Magnolia officinalis*[133]
**364b**	(−)-magmenthane D	*Magnolia officinalis*[133]
**365a**	(+)-magmenthane E	*Magnolia officinalis*[133]
**365b**	(−)-magmenthane E	*Magnolia officinalis*[133]

Table S21. Names, source species and references of phloroglucinol enantiomers

No	Name	Species & Reference
**366a**	(+)-myrtuspirone A	*Myrtus communis*[134]
**366b**	(−)-myrtuspirone A	*Myrtus communis*[134]
**367a**	(+)-cratosumatranone B	*Cratoxylum sumatranum* ssp. *neriifolium*[135]
**367b**	(−)-cratosumatranone B	*Cratoxylum sumatranum* ssp. *neriifolium*[135]
**368a**	(+)-japonicol B	*Hypericum japonicum*[136]
**368b**	(−)-japonicol B	*Hypericum japonicum*[136]
**369a**	(+)-japonicol A	*Hypericum japonicum*[136]
**369b**	(−)-japonicol A	*Hypericum japonicum*[136]
**370a**	(+)-japonicol C	*Hypericum japonicum*[136]
**370b**	(−)-japonicol C	*Hypericum japonicum*[136]
**371a**	(+)-japonicol D	*Hypericum japonicum*[136]
**371b**	(−)-japonicol D	*Hypericum japonicum*[136]
**372a**	(+)-mallopenin B	*Mallotus philippensis*[137]
**372b**	(−)-mallopenin B	*Mallotus philippensis*[137]
**373a**	(+)-mallopenin A	*Mallotus philippensis*[137]
**373b**	(−)-mallopenin A	*Mallotus philippensis*[137]
**374a**	(1′*S*,1′′′*R*)-myrtucommulone A	*Myrtus communis*[138]
**374b**	(1′*R*,1′′′*S*)-myrtucommulone A	*Myrtus communis*[138]
**375a**	(+)-xanthchrysone A	*Xanthostemon chrysanthus*[139]
**375b**	(−)-xanthchrysone A	*Xanthostemon chrysanthus*[139]
**376a**	(+)-xanthchrysone B	*Xanthostemon chrysanthus*[139]
**376b**	(−)-xanthchrysone B	*Xanthostemon chrysanthus*[139]
**377a**	(+)-xanthchrysone C	*Xanthostemon chrysanthus*[139]
**377b**	(−)-xanthchrysone C	*Xanthostemon chrysanthus*[139]
**378a**	(−)-cleistoperlone A	*Cleistocalyx operculatus*[140]
**378b**	(+)-cleistoperlone A	*Cleistocalyx operculatus*[140]
**379a**	(+)-cleistoperlone B	*Cleistocalyx operculatus*[140]
**379b**	(−)-cleistoperlone B	*Cleistocalyx operculatus*[140]
**380a**	(−)-nemorosonol	*Triadenum japonicum*[141]
**380b**	(+)-nemorosonol	*Clusia nemorosa*[142]
**381a**	(+)-garcimulin A	*Garcinia multiflora*[143]
**381b**	(−)-garcimulin A	*Garcinia multiflora*[143]
**382a**	(−)-garmultin B	*Garcinia multiflora*[144]
**382b**	(+)-garmultin B	*Garcinia multiflora*[144]
**383a**	(−)-garmultin C	*Garcinia multiflora*[144]
**383b**	(+)-garmultin C	*Garcinia multiflora*[144]
**384a**	(−)-garmultin D	*Garcinia multiflora*[144]
**384b**	(+)-garmultin D	*Garcinia multiflora*[144]
**385a**	(−)-garmultin F	*Garcinia multiflora*[144]
**385b**	(+)-garmultin F	*Garcinia multiflora*[144]

Table S22. Names, source species and references of naphthalene and phenanthrene enantiomers

No	Name	Species & Reference
**386a**	(+)-mornaphthoate A	*Morinda officinalis var. officinalis*[145]
**386b**	(−)-mornaphthoate A	*Morinda officinalis var. officinalis*[145]
**387a**	(+)-mornaphthoate B	*Morinda officinalis var. officinalis*[145]
**387b**	(−)-mornaphthoate B	*Morinda officinalis var. officinalis*[145]
**388a**	(+)-mornaphthoate C	*Morinda officinalis var. officinalis*[145]
**388b**	(−)-mornaphthoate C	*Morinda officinalis var. officinalis*[145]
**389a**	(+)-mornaphthoate D	*Morinda officinalis var. officinalis*[145]
**389b**	(−)-mornaphthoate D	*Morinda officinalis var. officinalis*[145]
**390a**	(−)-mornaphthoate E	*Morinda officinalis var. officinalis*[145]
**390b**	(+)-mornaphthoate E	*Morinda officinalis var. officinalis*[145]
**391a**	(+)-nonin A	*Morinda officinalis var. officinalis*[145]
**391b**	(−)-nonin A	*Morinda officinalis var. officinalis*[145]
**392a**	(1*S*)-2,3-dihydro-4′,8,8′-trihydroxy-(1,1′-binaphthalen)-4(1H)-one	*Juglans regia*[146]
**392b**	(1*R*)-2,3-dihydro-4′,8,8′-trihydroxy-(1,1′-binaphthalen)-4(1H)-one	*Juglans regia*[146]
**393a**	(1*S*)-2,3-dihydro-4′,5,8,8′-tetrahydroxy-(1,1′-binaphthalen)-4(1H)one	*Juglans regia*[146]
**393b**	(1*R*)-2,3-dihydro-4′,5,8,8′-tetrahydroxy-(1,1′-binaphthalen)-4(1H)one	*Juglans regia*[146]
**394a**	(1*R*)-2,3-dihydro-1′,5,5′,8-tetrahydroxy-(1,2′-binaphthalen)-4(1H)-one	*Juglans regia*[146]
**394b**	(1*S*)-2,3-dihydro-1′,5,5′,8-tetrahydroxy-(1,2′-binaphthalen)-4(1H)-one	*Juglans regia*[146]
**395a**	(+)-rubioncolin D	*Rubia oncotricha*[147]
**395b**	(−)-rubioncolin D	*Rubia oncotricha*[147]
**396a**	(+)-(2*S*,3*S*,4*R*,2′*S*,3′*R*)-rubialatin A	*Rubia alata*[148]
**396b**	(−)-(2*R*,3*R*,4*S*,2′*R*,3′*S*)-rubialatin A	*Rubia alata*[148]
**397a**	(+)-(4*S*)-altaicusin A	*Eremurus altaicus*[149]
**397b**	(+)-(4*R*)-altaicusin A	*Eremurus altaicus*[149]
**398a**	(*aS*,7′*S*,8′*R*)-bletillatin A	*Bletilla striata*[90]
**398b**	(*aR*,7′*R*,8′*S*)-bletillatin A	*Bletilla striata*[90]
**399a**	(*aS*,7′*S*,8′*R*)-shanciol H	*Bletilla striata*[90]
**399b**	(*aR*,7′*R*,8′*S*)-shanciol H	*Bletilla striata*[90]
**400a**	(*aS*,7′*S*,8′*R*)-bletilol C	*Bletilla striata*[90]
**400b**	(*aR*,7′*R*,8′*S*)-bletilol C	*Bletilla striata*[90]
**401a**	(*aS*,7′*S*,8′*R*)-pleionesin C	*Bletilla striata*[90]
**401b**	(*aR*,7′*R*,8′*S*)- pleionesin C	*Bletilla striata*[90]
**402a**	(*aS*,7′*S*,8′*R*)-(2,3-trans)-2-(4-hydroxy-3-methoxyphenyl)-3-hydroxymethyl-10-methoxy-2,3,4,5tetrahydrophenanthro [2,1-b]furan-7-ol	*Bletilla striata*[90]
**402b**	(*aR*,7′*R*,8′*S*)-(2,3-trans)-2-(4-hydroxy-3-methoxyphenyl)-3-hydroxymethyl-10-methoxy-2,3,4,5tetrahydrophenanthro [2,1-b]furan-7-ol	*Bletilla striata*[90]
**403a**	(*aS*,7′*S*,8′*R*)-bletilol B	*Bletilla striata*[90]
**403b**	(*aR*,7′*R*,8′*S*)-bletilol B	*Bletilla striata*[90]
**404a**	(*aR*,*a*′*R*)-blestriarene B	*Bletilla striata*[90]
**404b**	(*aS*,*a*′*S*)-blestriarene B	*Bletilla striata*[90]
**405a**	(*aS*)-1,4,7-trihydroxy-2-methoxy-9,10-dihydrophenanthrene	*Bletilla striata*[90]
**405b**	(*aR*)-1,4,7-trihydroxy-2-methoxy-9,10-dihydrophenanthrene	*Bletilla striata*[90]
**406a**	(*aR a*′*S*,*a*″*R*)-blestriarene A	*Bletilla striata*[90]

No	Name	Species & Reference
**406b**	(*aR a'R*,*a''S*)-blestriarene A	*Bletilla striata*[90]
**407a**	(*aS*)-4,8,4′,8′-tetramethoxy-(1,1′-biphenanthrene)-2,7,2′,7′-tetrol	*Bletilla striata*[90]
**407b**	(*aR*)-4,8,4′,8′-tetramethoxy-(1,1′-biphenanthrene)-2,7,2′,7′-tetrol	*Bletilla striata*[90]
**408a**	(*aS*)-monbarbatain A	*Bletilla striata*[90]
**408b**	(*aR*)-monbarbatain A	*Bletilla striata*[90]

Table S23. Names, source species and references of chromane enantiomers

No	Name	Species & Reference
**409a**	(+)-4*S*-hydroxy-6-methoxy-2,2-dimethylchromane	*Tussilago farfara*[150]
**409b**	(–)-4*R*-hydroxy-6-methoxy-2,2-dimethylchromane	*Tussilago farfara*[150]
**410a**	(+)-4*S*-hydroxy-2,2-dimethylchroman-6-acetone	*Tussilago farfara*[150]
**410b**	(–)-4*R*-hydroxy-2,2-dimethylchroman-6-acetone	*Tussilago farfara*[150]
**411a**	(+)-2,2-dimethyl-6-[1*S*-hydroxyethyl]-chroman-4-one	*Tussilago farfara*[150]
**411b**	(–)-2,2-dimethyl-6-[1*R*-hydroxyethyl]-chroman-4-one	*Tussilago farfara*[150]
**412a**	(+)-2,2-dimethyl-6-[1*S*-methoxyethyl]-chroman-4*S*-ol	*Tussilago farfara*[150]
**412b**	(–)-2,2-dimethyl-6-[1*R*-methoxyethyl]-chroman-4*R*-ol	*Tussilago farfara*[150]
**413a**	(+)-2,2-dimethyl-6-[1*S*-ethoxyethyl]-chroman-4*S*-ol	*Tussilago farfara*[150]
**413b**	(–)-2,2-dimethyl-6-[1*R*-ethoxyethyl]-chroman-4*R*-ol	*Tussilago farfara*[150]
**414a**	(–)-2,2-dimethyl-6-[1*R*-methoxyethyl]-chroman-4*S*-ol	*Tussilago farfara*[150]
**414b**	(+)-2,2-dimethyl-6-[1*S*-methoxyethyl]-chroman-4*R*-ol	*Tussilago farfara*[150]
**415a**	(–)-2,2-dimethyl-6-[1*R*-ethoxyethyl]-chroman-4*S*-ol	*Tussilago farfara*[150]
**415b**	(+)-2,2-dimethyl-6-[1*S*-ethoxyethyl]-chroman-4*R*-ol	*Tussilago farfara*[150]
**416a**	(+)-curcumane D	*Curcuma longa*[151]
**416b**	(–)-curcumane D	*Curcuma longa*[151]
**417a**	(+)-perforatin C	*Harrisonia perforate*[152]
**417b**	(–)-perforatin C	*Harrisonia perforate*[152]
**418a**	(+)-2′-*O*-acetylperforatin C	*Harrisonia perforate*[152]
**418b**	(–)-2′-*O*-acetylperforatin C	*Harrisonia perforate*[152]
**419a**	(+)-erythro-10-hydroxyperforatin C	*Harrisonia perforate*[152]
**419b**	(–)-erythro-10-hydroxyperforatin C	*Harrisonia perforate*[152]
**420a**	(+)-horriperfin A	*Harrisonia perforate*[152]
**420b**	(–)-horriperfin A	*Harrisonia perforate*[152]
**421a**	(+)-horriperfin B	*Harrisonia perforate*[152]
**421b**	(–)-horriperfin B	*Harrisonia perforate*[152]
**422a**	(+)-perforison A	*Harrisonia perforate*[153]
**422b**	(–)-perforison A	*Harrisonia perforate*[153]

Table S24. Names, source species and references of acetophenone enantiomers

No	Name	Species & Reference
**423a**	(+)-evodialone A	*Evodia lepta* [154], *Melicope ptelefolia*[155]
**423b**	(−)-evodialone A	*Evodia lepta*[154], *Melicope ptelefolia*[155]
**424a**	(+)-evodialone B	*Evodia lepta*[154], *Melicope ptelefolia*[155]
**424b**	(−)-evodialone B	*Evodia lepta*[154], *Melicope ptelefolia*[155]
**425a**	(+)-evodialone C	*Melicope ptelefolia*[155]
**425b**	(−)-evodialone C	*Melicope ptelefolia*[155]
**426a**	(+)-evodialone D	*Melicope ptelefolia*[155]
**426b**	(−)-evodialone D	*Melicope ptelefolia*[155]
**427a**	(+)-xanchryone E	*Xanthostemon chrysanthus*[139]
**427b**	(−)-xanchryone E	*Xanthostemon chrysanthus*[139]
**428a**	(+)-meliviticines A	*Melicope viticina*[156]
**428b**	(−)-meliviticines A	*Melicope viticina*[156]
**429a**	(−)-eupachinin A	*Eupatorium chinense*[157]
**429b**	(+)-eupachinin A	*Eupatorium chinense*[157]
**430a**	(−)-eupachinin B	*Eupatorium chinense*[157]
**430b**	(+)-eupachinin B	*Eupatorium chinense*[157]
**431a**	(+)-melicolone A	*Melicope ptelefolia*[158]
**431b**	(−)-melicolone A	*Melicope ptelefolia*[158]
**432a**	(+)-melicolone B	*Melicope ptelefolia*[158]
**432b**	(−)-melicolone B	*Melicope ptelefolia*[158]

Table S25. Names, source species and references of diarylheptanoid enantiomers

No	Name	Species & Reference
**433a**	(+)-alpininoid A	*Alpinia officinarum*[159]
**433b**	(−)-alpininoid A	*Alpinia officinarum*[159]
**434a**	(+)-alpininoid B	*Alpinia officinarum*[159]
**434b**	(−)-alpininoid B	*Alpinia officinarum*[159]
**435a**	(+)-alpininoid C	*Alpinia officinarum*[159]
**435b**	(−)-alpininoid C	*Alpinia officinarum*[159]
**436a**	(+)-alpininoid D	*Alpinia officinarum*[159]
**436b**	(−)-alpininoid D	*Alpinia officinarum*[159]
**437a**	(+)-alpininoid E	*Alpinia officinarum*[159]
**437b**	(−)-alpininoid E	*Alpinia officinarum*[159]
**438a**	(1*S*,3*S*,5*S*)-1,7-bis(4-hydroxyphenyl)-1,5-epoxy-3hydroxyheptane	*Dioscorea villosa*[160]
**438b**	(1*R*,3*R*,5*R*)-1,7-bis(4-hydroxyphenyl)-1,5-epoxy-3hydroxyheptane	*Dioscorea villosa*[160]
**439a**	(1*S*,3*R*,5*S*)-1,7-Bis(4-hydroxyphenyl)-1,5-epoxy-3-hydroxyheptane	*Dioscorea villosa*[160]
**439b**	(1*R*,3*S*,5*R*)-1,7-Bis(4-hydroxyphenyl)-1,5-epoxy-3-hydroxyheptane	*Dioscorea villosa*[160]
**440a**	(5*R*,1*E*)-1,7-Bis(4-hydroxyphenyl)-5-hydroxyhept-1-en-3-one	*Dioscorea villosa*[160]
**440b**	(5*S*,1*E*)-1,7-Bis(4-hydroxyphenyl)-5-hydroxyhept-1-en-3-one	*Dioscorea villosa*[160]
**441a**	(5*S*)-1,7-bis(4-hydroxyphenyl)-5-hydroxyheptan-3-one	*Dioscorea villosa*[160]
**441b**	(5*R*)-1,7-bis(4-hydroxyphenyl)-5-hydroxyheptan-3-one	*Dioscorea villosa*[160]

Table S26. Names, source species and references of triarylmethane enantiomers

No	Name	Species & Reference
**442a**	(−)-securidane B	*Securidaca inappendiculata*[161]
**442b**	(+)-securidane B	*Securidaca inappendiculata*[161]
**443a**	(−)-securidane A	*Securidaca inappendiculata*[161]
**443b**	(+)-securidane A	*Securidaca inappendiculata*[161]
**444a**	(+)-*R*-selaginellin P	*Selaginella pulvinata*[162]
**444b**	(−)-*S*-selaginellin P	*Selaginella pulvinata*[162]
**445**	selaginellin Q	*Selaginella pulvinata*[162]
**446**	selaginellin R	*Selaginella pulvinata*[162]
**447a**	(+)-*R*-selaginellin D	*Selaginella pulvinata*[162]
**447b**	(−)-*S*-selaginellin D	*Selaginella pulvinata*[162]

Table S27. Names, source species and references of fatty acid enantiomers

No	Name	Species & Reference
**448a**	methyl (10*E*,12*E*)-14*S*-hydroxy-9-oxo-10,12-octadecadienoate	*Plantago depressa*[163]
**448b**	methyl (10*E*,12*E*)-14*R*-hydroxy-9-oxo-10,12-octadecadienoate	*Plantago depressa*[163]
**449a**	methyl (10*E*,12*E*,14*E*)-16*S*-methoxy-9-oxo-10,12,14-octadecatrienoate	*Plantago depressa*[163]
**449b**	methyl (10*E*,12*E*,14*E*)-16*R*-methoxy-9-oxo-10,12,14-octadecatrienoate	*Plantago depressa*[163]
**450a**	methyl (10*E*,12*E*,14*E*)-16*S*-hydroxy-9-oxo-10,12,14-octadecatrienoate	*Plantago depressa*[163]
**450b**	methyl (10*E*,12*E*,14*E*)-16*R*-hydroxy-9-oxo-10,12,14-octadecatrienoate	*Plantago depressa*[163]
**451a**	methyl (10*E*,12*E*,)-14*R,*15*S*-dihydroxy-16*R*-chloro-9-oxo-10,12-octadecadienoate	*Plantago depressa*[163]
**451b**	methyl (10*E*,12*E*,)-14*S,*15*R*-dihydroxy-16*S*-chloro-9-oxo-10,12-octadecadienoate	*Plantago depressa*[163]
**452a**	(2'*S*)-1-*O*-(9-oxo-12Z-octadecamonoyl) glycerol	*Ipomoea nil*[164]
**452b**	(2'*R*)-1-*O*-(9-oxo-12Z-octadecamonoyl) glycerol	*Ipomoea nil*[164]

Table S28. Names, source and species of miscellaneous enantiomers

No	Name	Species & Reference
**453a**	(−) riligustilide	*Angelica sinensis*[165]
**453b**	(+) riligustilide	*Angelica sinensis*[165]
**454a**	(+) tokinolide A	*Angelica sinensis*[165]
**454b**	(−) tokinolide A	*Angelica sinensis*[165]
**455a**	(−) triligustilide A	*Angelica sinensis*[165]
**455b**	(+) triligustilide A	*Angelica sinensis*[165]
**456a**	(−) triligustilide B	*Angelica sinensis*[165]
**456b**	(+) triligustilide B	*Angelica sinensis*[165]
**457a**	(+)-cajanusine,	*Cajanus cajan*[166]
**457b**	(−)-cajanusine,	*Cajanus cajan*[166]
**458a**	(+)-(*E*)-cyperusphenol A	*Cyperus rhizomes*[167-168]
**458b**	(−)-(*E*)-cyperusphenol A	*Cyperus rhizomes*[167]
**459a**	(−)-decumbic acid	*Dendrobium nobile*[23]
**459b**	(+)-decumbic acid	*Dendrobium nobile*[23]
**460a**	(+)-melivitic ines B	*Melicope viticina*[156]
**460b**	(−)-meliviticines B	*Melicope viticina*[156]
**461a**	(+)-ligusticumtone	*Ligusticum chuanxiong*[169]
**461b**	(−)-ligusticumtone	*Ligusticum chuanxiong*[169]
**462a**	(−)-incarvilleatone	*Incarvillea younghusbandii*[170]
**462b**	(+)-incarvilleatone	*Incarvillea younghusbandii*[170]
**463a**	(+)-denobilone A	*Dendrobium nobile*[171]
**463b**	(−)-denobilone A	*Dendrobium nobile*[171]
**464a**	(+)-6-styryl-7,8-dihydroxy-4methoxypyran-2-one	*Sanrafaelia ruffonammari Ophrypetalum odoratum*[172]
**464b**	(−)-6-styryl-7,8-dihydroxy-4methoxypyran-2-one	*Sanrafaelia ruffonammari Ophrypetalum odoratum*[172]
**465**	(±)-5-methoxy-7-phenyl-[4-methoxy-2-pyronyl]-1-(*E*)-styryl-2-oxabi cyclo-[4.2.0]-octa-4-en-3-one	*Sanrafaelia ruffonammari Ophrypetalum odoratum*[172]
**466a**	(+)-indidene A	*Streblus indicus*[173]
**466b**	(−)-indidene A	*Streblus indicus*[173]

Table S29. Names, source species and references of non-alkaloids enantiomers from phylum Ascomycota

No	Name	Species & Reference
**467**	penicilliode A	*Penicillium* sp. GGF16-1-2[174]
**468a**	(−)-penicilliode B	*Penicillium* sp. GGF16-1-2[174]
**468b**	(+)-isoconiochaetone B	*Penicillium* sp. GGF16-1-2[174]
**469a**	(+)-penicilliode C	*Penicillium* sp. GGF16-1-2[174]
**469b**	(−)-isopenicilliode C	*Penicillium* sp. GGF16-1-2[174]
**470a**	(+)-coniochaetone B	*Penicillium* sp. GGF16-1-2[174]
**470b**	(−)-isoconiochaetone B	*Penicillium* sp. GGF16-1-2[174]
**471a**	(+)-coniochaetone C	*Penicillium* sp. GGF16-1-2[174]
**471b**	(−)-isoconiochaetone C	*Penicillium* sp. GGF16-1-2[174]
**472a**	(+)-(4S,5S)-4-hydroxy-3-methoxy-5-methyl-2-cyclopentenone	*Aspergillus Sclerotiorum*[175]
**472b**	(+)-(4S,5S)-4-hydroxy-3-methoxy-5-methyl-2-cyclopentenone	*Aspergillus Sclerotiorum*[175]
**473a**	(−)-aspersclerolide D	*Aspergillus Sclerotiorum*[175]
**473b**	(+)-aspersclerolide D	*Aspergillus Sclerotiorum*[175]
**474a**	(−)-nigrosporaol A	*Nigrospora sphaerica, Alternaria alternata, and Phialophora* sp.[176]
**474b**	(+)-nigrosporaol A	*Nigrospora sphaerica, Alternaria alternata, and Phialophora* sp.[176]
**475a**	(−)-peniphenone A	*Penicillium dipodomyicola* HN4-3A[177]
**475b**	(+)-peniphenone A	*Penicillium dipodomyicola* HN4-3A[177]
**476a**	(1′*S*)-sporulosol	*Paraconiothyrium sporulosum*[178]
**476b**	(1′*R*)-sporulosol	*Paraconiothyrium sporulosum*[178]
**477**	penicoffeazine B	*Penicillium coffeae* MA-314[179]
**478**	penicoffeazine C	*Penicillium coffeae* MA-314[179]
**479a**	(+)-peniorthoester A	*Penicillium minioluteum*[180]
**479b**	(−)-peniorthoester A	*Penicillium minioluteum*[180]
**480a**	(+)-peniorthoester B	*Penicillium minioluteum*[180]
**480b**	(−)-peniorthoester B	*Penicillium minioluteum*[180]
**481a**	(+)-(*S*)-6-Hydroxy-1,8-dimethoxy-3a-methyl-3,3a-dihydrocyclopenta[c]isochromene-2,5-dione	*Alternaria* sp. TNXY-P-1[181]
**481b**	(−)-(*R*)-6-Hydroxy-1,8-dimethoxy-3a-methyl-3,3a-dihydrocyclopenta[c]isochromene-2,5-dione	*Alternaria* sp. TNXY-P-1[181]
**482a**	(+)-alternarilactone A	*Endophytic alternaria* sp.[182]
**482b**	(−)-Alternarilactone A	*Endophytic alternaria* sp.[182]
**483a**	(+)-(2*S*,3*S*,4a*S*)-altenuene-2-acetoxy ester	*Alternaria alternate*[183]
**483b**	(−)-(2*R*,3*R*,4a*R*)-altenuene-2-acetoxy ester	*Alternaria alternate*[183]
**484a**	(+)-(2*S*,3*S*,4a*S*)altenuene-3-acetoxy ester	*Alternaria alternate*[183]
**484b**	(−)-(2*S*,3*S*,4a*S*)altenuene-3-acetoxy ester	*Alternaria alternate*[183]
**485a**	(*R*)-taeniolin	*Taeniolella* sp. *BCC31839*[184]
**485b**	(*S*)-taeniolin	*Taeniolella* sp. *BCC31839*[184]
**486**	pericocin A	*Periconia* sp.[185]
**487a**	3*R*,4*S*-3,8-dimethoxy-3-methylisochromane-4,6-diol	*Aspergillus fumigatus*[186]
**487b**	3*S*,4*R*-3,8-dimethoxy-3-methylisochromane-4,6-diol	*Aspergillus fumigatus*[186]
**488a**	3*S*,4*R*-3,8-dimethoxy-3-methylisochromane-4,6-diol	*Aspergillus fumigatus*[186]
**488b**	3*R*,4*S*-3,8-dimethoxy-3-methylisochromane-4,6-diol	*Aspergillus fumigatus*[186]
**489**	fimetarone A	*Fimetariella* sp.[187]
**490a**	(*S*)-3,6-dihydroxy-8-methoxy-3-methylisochroman-4-one	*Aspergillus fumigatus*[188]
**490b**	(*R*)-3,6-dihydroxy-8-methoxy-3-methylisochroman-4-one	*Aspergillus fumigatus*[188]
**491a**	(2*S*,3*R*)- floricolin I	*Floricola striata*[189]
**491b**	(2*R*,3*S*)- floricolin I	*Floricola striata*[189]

No	Name	Species & Reference
**492a**	(1*R*,4*S*)- floricolin J	*Floricola striata*[189]
**492b**	(1*S*,3*R*)- floricolin J	*Floricola striata*[189]
**493a**	(−)-mangostafeejin A	*Xylaria feejeensis* GM06[190]
**493b**	(+)-mangostafeejin A	*Xylaria feejeensis* GM06[190]
**494a**	(−)-mangostafeejin B	*Xylaria feejeensis* GM06[190]
**494b**	(+)-mangostafeejin B	*Xylaria feejeensis* GM06[190]
**495a**	(+)-ascomlactone A	*Ascomycota* sp. SK2YWS-L[191]
**495b**	(−)-ascomlactone A	*Ascomycota* sp. SK2YWS-L[191]
**496a**	(+)-ascomindone D	*Ascomycota* sp. SK2YWS-L[192]
**496b**	(−)-ascomindone D	*Ascomycota* sp. SK2YWS-L[192]
**497a**	(+)-terreinlactone A	*Aspergillus terreus*[193]
**497b**	(−)-terreinlactone A	*Aspergillus terreus*[193]
**498**	tolypocladone A	*Tolypocladium* sp.[194]
**499a**	(+)-phomone A	*Phoma* sp. YN02-P-3[195]
**499b**	(−)-phomone A	*Phoma* sp. YN02-P-3[195]
**500a**	(+)-phomone B	*Phoma* sp. YN02-P-3[195]
**500b**	(−)-phomone B	*Phoma* sp. YN02-P-3[195]
**501**	pochoniolide A	*Pochonia chlamydosporia* var. *spinulospora* FKI-7537[196]
**502**	pochoniolide B	*Pochonia chlamydosporia* var. *spinulospora* FKI-7537[196]
**503a**	penicichrysogenin K	*Penicillium chrysogenum* MT-12[197]
**503b**	penicichrysogenin J	*Penicillium chrysogenum* MT-12[197]
**504a**	pinophilone A	*Penicillium pinophilum* SCAU037[198]
**504b**	pinophilone B	*Penicillium pinophilum* SCAU037[198]
**505a**	(−)-asperlone A	*Penicillium chrysogenum* MT-12[197]
**505b**	(+)-asperlone A	*Penicillium chrysogenum* MT-12[197]
**506a**	(+)-canescone A	*Penicillium canescens*[199]
**506b**	(−)-canescone A	*Penicillium canescens*[199]
**507a**	(+)-canescone B	*Penicillium canescens*[199]
**507b**	(−)-canescone B	*Penicillium canescens*[199]
**508a**	(+)-preuisolactone A	*Preussia isomera*[200]
**508b**	(−)-preuisolactone A	*Preussia isomera*[200]
**509a**	(+)-pestalachlorides G	*Pestalotiopsis* sp.[201]
**509b**	(−)-pestalachlorides G	*Pestalotiopsis* sp.[201]
**510a**	(*S*)-europhenol A	*Eurotium rubrum* MA-150[202]
**510b**	(*R*)-europhenol A	*Eurotium rubrum* MA-150[202]
**511a**	(+)-*S*-arugosin K	*Talaromyces flavus*[203]
**511b**	(−)-*R*-arugosin K	*Talaromyces flavus*[203]
**512a**	(+)-*S*-arugosin L	*Talaromyces flavus*[203]
**512b**	(−)-*R*-arugosin L	*Talaromyces flavus*[203]
**513a**	(+)-*S*-arugosin M	*Talaromyces flavus*[203]
**513b**	(−)-*R*-arugosin M	*Talaromyces flavus*[203]
**514a**	(6*R*)-arugosin J	*Xylaria* sp.[204]
**514b**	(6*S*)-arugosin J	*Xylaria* sp.[204]
**515a**	(+)-cytorhizophin A	*Cytospora rhizophorae*[205]
**515b**	(−)-cytorhizophin A	*Cytospora rhizophorae*[205]
**516a**	(−)-alternamgin	*Alternaria* sp. MG1[206]
**516b**	(+)-alternamgin	*Alternaria* sp. MG1[206]
**517a**	(+)-eurotone A	*Eurotium* sp. SCSIO F452[207]
**517b**	(−)-eurotone A	*Eurotium* sp. SCSIO F452[207]

Table S30. Names, source species and references of alkaloid enantiomers from phylum Ascomycota

No	Name	Species & Reference
**518a**	(+)-fusaspoid A	*Fusarium* sp. XBB-9[208]
**518b**	(−)-fusaspoid A	*Fusarium* sp. XBB-9[208]
**519a**	(+)-brevianamide X	*Aspergillus versicolor* OUCMDZ-2738[209]
**519b**	(−)-brevianamide X	*Aspergillus versicolor* OUCMDZ-2738[209]
**520a**	(+)-brevianamide R	*Aspergillus versicolor* OUCMDZ-2738[209]
**520b**	(−)-brevianamide R	*Aspergillus versicolor* OUCMDZ-2738[209]
**521a**	(+)-brevianamide Q	*Aspergillus versicolor OUCMDZ-2738*[209]
**521b**	(−)-brevianamide Q	*Aspergillus versicolor* OUCMDZ-2738[209]
**522a**	(+)-brevianamide V	*Aspergillus versicolor* OUCMDZ-2738[209]
**522b**	(−)-brevianamide V	*Aspergillus versicolor* OUCMDZ-2738[209]
**523a**	(+)-acrozine A	*Acrostalagmus luteoalbus* TK-43[210]
**523b**	(−)-acrozine A	*Acrostalagmus luteoalbus* TK-43[210]
**524a**	(+)-acrozine B	*Acrostalagmus luteoalbus* TK-43[210]
**524b**	(−)-acrozine B	*Acrostalagmus luteoalbus* TK-43[210]
**525a**	(+)-acrozine C	*Acrostalagmus luteoalbus* TK-43[210]
**525b**	(−)-acrozine C	*Acrostalagmus luteoalbus* TK-43[210]
**526a**	(+)-eurotinoid A	*Eurotium* sp. SCSIO F452[211]
**526b**	(−)-eurotinoid A	*Eurotium* sp. SCSIO F452[211]
**527a**	(−)-eurotinoid B	*Eurotium* sp. SCSIO F452[211]
**527b**	(+)-eurotinoid B	*Eurotium* sp. SCSIO F452[211]
**528a**	(+)-eurotinoid C	*Eurotium* sp. SCSIO F452[211]
**528b**	(−)-eurotinoid C	*Eurotium* sp. SCSIO F452[211]
**529a**	(12*R*,28*S*,31*S*)-dihydrocryptoechinulin D	*Aspergillus effuses* H1-1[212]<br>*Eurotium* sp. SCSIO F452[211]
**529b**	(12*S*,28*R*,31*R*)-dihydrocryptoechinulin D	*Aspergillus effuses* H1-1[212]<br>*Eurotium* sp. SCSIO F452[211]
**530a**	(−)-asperginulin A	Aspergillus sp. SK-28[213]
**530b**	(+)-asperginulin A	Aspergillus sp. SK-28[213]
**531a**	(12*R*,21*R*,28*R*,29*R*)-effusin A	*Aspergillus effuses* H1-1[212]
**531b**	(12*S*,21*S*,28*S*,29*S*)-effusin A	*Aspergillus effuses* H1-1[212]
**532a**	(−)-(*S*)-7-O-methylvariecolortide A	*Eurotium sp.*[214]
**532b**	(+)-(*R*)-7-O-methylvariecolortide A	*Eurotium* sp.[214]
**533a**	(−)-(*S*)-variecolortide B	*Eurotium* sp.[214]
**533b**	(+)-(*R*)-variecolortide B	*Eurotium* sp.[214]
**534a**	(−)-(*S*)-variecolortide C	*Eurotium* sp.[214]
**534b**	(+)-(*R*)-variecolortide C	*Eurotium* sp.[214].
**535a**	(+)-variecolortin A	*Eurotium* sp. SCSIO F452[215]
**535b**	(−)-variecolortin A	*Eurotium* sp. SCSIO F452[215]
**536a**	(+)-variecolortin B	*Eurotium* sp. SCSIO F452[215]
**536b**	(−)-variecolortin B	*Eurotium* sp. SCSIO F452[215]
**537a**	(+)-variecolortin C	*Eurotium* sp. SCSIO F452[215]
**537b**	(−)-variecolortin C	*Eurotium* sp. SCSIO F452[215]
**538a**	(+)-didymellamide E	*Coniochaeta cephalothecoides*[216]
**538b**	(−)-didymellamide E	*Coniochaeta cephalothecoides*[216]
**539a**	(+)-conipyrrolidone C	*Coniochaeta cephalothecoides*[216]
**539b**	(−)-conipyrrolidone C	*Coniochaeta cephalothecoides*[216]
**540a**	(−)-conipyrrolidone D	*Coniochaeta cephalothecoides*[216]
**540b**	(+)-conipyrrolidone D	*Coniochaeta cephalothecoides*[216]
**541a**	(−)-conipyrrolidone A	*Coniochaeta cephalothecoides*[216]
**541b**	(+)-conipyrrolidone A	*Coniochaeta cephalothecoides*[216]
**542a**	(5*R*,8*S*,9*S*,14*R*,17*S*)-conipyrrolidone B	*Coniochaeta cephalothecoides*[216]
**542b**	(5*S*,8*R*,9*R*,14*S*,17*R*)-conipyrrolidone B	*Coniochaeta cephalothecoides*[216]
**543a**	(+)-xylaridine A	*Xylaria longipes*[217]

No	Name	Species & Reference
**543b**	(−)-xylaridine A	*Xylaria longipes*[217]
**544a**	(+)-xylaridines C	*Xylaria longipes*[218]
**544b**	(−)-xylaridine C	*Xylaria longipes*[218]
**545a**	(+)-xylaridine D	*Xylaria longipes*[218]
**545b**	(−)-xylaridine D	*Xylaria longipes*[218]
**546a**	(+)-penicamide A	*Penicillium* sp. 4829[219]
**546b**	(−)-penicamide A	*Penicillium* sp. 4829[219]
**547a**	2-[(*S*)-hydroxy(phenyl)methyl]-3-methylquinazolin4(3*H*)-one	*Talaromyces* sp.[220]
**547b**	2-[(*R*)-hydroxy(phenyl)methyl]-3-methylquinazolin4(3*H*)-one	*Talaromyces* sp.[220]
**548a**	(+)-xylaridine B	*Xylaria longipes*[217]
**548b**	(−)-xylaridine B	*Xylaria longipes*[217]
**549a**	(+)-penicilactam A	*Penicillium griseofulvum*[221]
**549b**	(−)-penicilactam A	*Penicillium griseofulvum*[221]
**550a**	(+)-canescone C	*Penicillium canescens*[199]
**550b**	(−)-canescone C	*Penicillium canescens*[199]
**551a**	marilines A_1	*Stachylidium* sp.[222]
**551b**	marilines A_2	*Stachylidium* sp.[222]
**552a**	(−)-trichodermadione A	*Trichoderma atroviride* S361[223]
**552b**	(+)-trichodermadione A	*Trichoderma atroviride* S361[223]
**553a**	(−)-fusagerin A	*Fusarium* sp.[224]
**553b**	(+)-fusagerin A	*Fusarium* sp.[224]
**554a**	(+)-brasilamide G	*Paraconiothyrium brasiliense*[225]
**554b**	(−)-brasilamide G	*Paraconiothyrium brasiliense*[225]
**555**	brasilamide H	*Paraconiothyrium brasiliense*[225]
**556**	brasilamide I	*Paraconiothyrium brasiliense*[225]
**557**	brasilamide J	*Paraconiothyrium brasiliense*[225]
**558a**	(−)-pestaloxazine A	*Pestalotiopsis* sp.[226]
**558b**	(+)-pestaloxazine A	*Pestalotiopsis* sp.[226]

Table S31. Names, source species and references of enantiomers from phylum Basidiomycota

No	Name	Species & Reference
**559a**	(–)-lucidumone	*Ganoderma lucidum*[227]
**559b**	(+)-lucidumone	*Ganoderma lucidum*[227]
**560a**	(+)-ganocin A	*Ganoderma cochlear*[228]
**560b**	(–)-ganocin A	*Ganoderma cochlear*[228]
**561a**	(+)-ganocin B	*Ganoderma cochlear*[228]
**561b**	(–)-ganocin B	*Ganoderma cochlear*[228]
**562a**	(+)-ganocin C	*Ganoderma cochlear*[228]
**562b**	(–)-ganocin C	*Ganoderma cochlear*[228]
**563a**	(+)-ganocin D	*Ganoderma cochlear*[228]
**563b**	(–)-ganocin D	*Ganoderma cochlear*[228]
**564a**	(–)-lingzhiol	*Ganoderma lucidum*[229]
**564b**	(+)-lingzhiol	*Ganoderma lucidum*[229]
**565a**	(+)-ganoresinain A	*Ganoderma resinaceum*[230]
**565b**	(–)-ganoresinain A	*Ganoderma resinaceum*[230]
**566a**	(+)-applanatumol B	*Ganoderma applanatum*[231]
**566b**	(–)-applanatumol B	*Ganoderma applanatum*[231]
**567a**	(+)-chizhine E	*Ganoderma applanatum*[232]
**567b**	(–)-chizhine E	*Ganoderma applanatum*[232]
**568a**	(+)-ganomycin I	*Ganoderma applanatum*[232]
**568b**	(–)-ganomycin I	*Ganoderma applanatum*[232]
**569a**	(+)-fornicin B	*Ganoderma applanatum*[232]
**569b**	(–)-fornicin B	*Ganoderma applanatum*[232]
**570a**	(+)-lucidulactone B	*Ganoderma lucidum*[233]
**570b**	(–)-lucidulactone B	*Ganoderma lucidum*[233]
**571a**	(+)-chizhine A	*Ganoderma applanatum*[232]
**571b**	(–)-chizhine A	*Ganoderma applanatum*[232]
**572a**	(+)-chizhine B	*Ganoderma applanatum*[232]
**572b**	(–)-chizhine B	*Ganoderma applanatum*[232]
**573a**	(+)-chizhine F	*Ganoderma applanatum*[232]
**573b**	(–)-chizhine F	*Ganoderma applanatum*[232]
**574a**	(+)-chizhine D	*Ganoderma applanatum*[232]
**574b**	(–)-chizhine D	*Ganoderma applanatum*[232]
**575a**	(+)-chizhine C	*Ganoderma applanatum*[232]
**575b**	(–)-chizhine C	*Ganoderma applanatum*[232]
**576a**	(+)-applanatumol Z3	*Ganoderma applanatum*[234]
**576b**	(–)-applanatumol Z3	*Ganoderma applanatum*[234]
**577a**	(+)-zizhine A	*Ganoderma sinensis*[235]
**577b**	(–)-zizhine A	*Ganoderma sinensis*[235]
**578a**	(+)-zizhine B	*Ganoderma sinensis*[235]
**578b**	(–)-zizhine B	*Ganoderma sinensis*[235]
**579a**	(+)-zizhine D	*Ganoderma sinensis*[235]
**579b**	(–)-zizhine D	*Ganoderma sinensis*[235]
**580a**	(+)-zizhine C	*Ganoderma sinensis*[235]
**580b**	(–)-zizhine C	*Ganoderma sinensis*[235]
**581a**	(+)-zizhine E	*Ganoderma sinensis*[235]
**581b**	(–)-zizhine E	*Ganoderma sinensis*[235]
**582**	zizhine F	*Ganoderma sinensis*[235]
**583a**	ganosinensol A	*Ganoderma sinense*[236]
**583b**	ganosinensol B	*Ganoderma sinense*[236]
**584a**	ganosinensol C	*Ganoderma sinense*[236]
**584b**	ganosinensol D	*Ganoderma sinense*[236]
**585a**	(+)-ganoapplanin	*Ganoderma applanatum*[237]
**585b**	(–)-ganoapplanin	*Ganoderma applanatum*[237]

No	Name	Species & Reference
**586a**	(+)-applanatumine A	*Ganoderma applanatum*[238]
**586b**	(–)-applanatumine A	*Ganoderma applanatum*[238]
**587a**	(+)-applanatumine B	*Ganoderma applanatum*[238]
**587b**	(–)-applanatumine B	*Ganoderma applanatum*[238]
**588a**	(+)-applanatumine C	*Ganoderma applanatum*[238]
**588b**	(–)-applanatumine C	*Ganoderma applanatum*[238]
**589a**	(+)-gancochlearol A	*Ganoderma cochlear*[239]
**589b**	(–)-gancochlearol A	*Ganoderma cochlear*[239]
**590a**	(+)-gancochlearol B	*Ganoderma cochlear*[239]
**590b**	(–)-gancochlearol B	*Ganoderma cochlear*[239]
**591a**	(+)-cochlearoid N	*Ganoderma cochlear*[240]
**591b**	(–)-cochlearoid N	*Ganoderma cochlear*[240]
**592a**	(+)-cochlearoid P	*Ganoderma cochlear*[240]
**592b**	(–)-cochlearoid P	*Ganoderma cochlear*[240]
**593a**	(+)-cochlearoid Q	*Ganoderma cochlear*[240]
**593b**	(–)-cochlearoid Q	*Ganoderma cochlear*[240]
**594a**	(+)-cochlearoid A	*Ganoderma cochlear*[241]
**594b**	(–)-cochlearoid A	*Ganoderma cochlear*[241]
**595a**	(+)-cochlearoid B	*Ganoderma cochlear*[241]
**595b**	(–)-cochlearoid B	*Ganoderma cochlear*[241]
**596a**	(+)-cochlearoid C	*Ganoderma cochlear*[241]
**596b**	(–)-cochlearoid C	*Ganoderma cochlear*[241]
**597a**	(+)-cochlearoid E	*Ganoderma cochlear*[241]
**597b**	(–)-cochlearoid E	*Ganoderma cochlear*[241]
**598a**	(+)-cochlearoid D	*Ganoderma cochlear*[241]
**598b**	(–)-cochlearoid D	*Ganoderma cochlear*[241]
**599a**	(+)-cochlearine A	*Ganoderma cochlear*[241]
**599b**	(–)-cochlearine A	*Ganoderma cochlear*[241]
**600a**	(–)-cochlearine B	*Ganoderma cochlear*[241]
**600b**	(–)-cochlearine B	*Ganoderma cochlear*[241]
**601a**	(+)-6*S*-hydroxyganocochlearine A	*Ganoderma luteomarginatum*[242]
**601b**	(–)-6*S*-hydroxyganocochlearine A	*Ganoderma luteomarginatum*[242]
**602a**	(+)-lucidulactone A	*Ganoderma lucidum*[233]
**602b**	(–)-lucidulactone A	*Ganoderma lucidum*[233]
**603a**	(3*S*,7*R*)-illudin M	*Granulobasidium vellereum*[243]
**603b**	(3*R*,7*S*)-illudin M	*Granulobasidium vellereum*[243]
**604a**	(3*S*,4*S*,7*R*)-dihydroilludin M	*Granulobasidium vellereum*[243]
**604b**	(3*R*,4*R*,7*S*)-dihydroilludin M	*Granulobasidium vellereum*[243]

Table S32. Names and species of enantiomeric compounds from actinomycetes

No	Name	Species & Reference
**605a**	(+)-actinoxocine	*Streptomyces* sp.[244]
**605b**	(−)-actinoxocine	*Streptomyces* sp.[244]
**606**	streptoprenylindole A	*Streptomyces* sp. ZZ820[245]
**607a**	(+)-(*S*)-pratensilins A	*Streptomyces* sp.[246]
**607b**	(−)-(*R*)-pratensilins A	*Streptomyces* sp.[246]
**608a**	(+)-(*S*)-pratensilins B	*Streptomyces* sp.[246]
**608b**	(−)-(*R*)-pratensilins B	*Streptomyces* sp.[246]
**609a**	(+)-(*S*)-pratensilins C	*Streptomyces* sp.[246]
**609b**	(−)-(*R*)-pratensilins C	*Streptomyces* sp.[246]

Table S33. Names, source species and references of enantiomers from phylum Porifera

No	Name	Species & Reference
**610a**	(+)-spongiterpene	*Spongia* sp.[247]
**610b**	(−)-spongiterpene	*Spongia* sp.[247]
**611a**	(+)-sponalisolide A	*Spongia officinalis*[248]
**611b**	(−)-sponalisolide A	*Spongia officinalis*[248]
**612a**	(+)-cavernosine	*Cacospongia* sp.[249]
**612b**	(−)-cavernosine	*Cacospongia* sp.[249]
**613a**	(+)-8,13-secocavernosine	*Cacospongia* sp.[249]
**613b**	(−)-8,13-secocavernosine	*Cacospongia* sp.[249]
**614**	(±)-8,13-secoepicavernosine	*Cacospongia* sp.[249]
**615a**	(+)-hippolide E	*Cacospongia* sp.[249]
**615a**	(−)-hippolide E	*Cacospongia* sp.[249]
**616a**	(+)-(6*E*)-neomanoalide	*Cacospongia* sp.[249]
**616b**	(−)-(6*E*)-neomanoalide	*Cacospongia* sp.[249]
**617a**	(−)-hippolide J	*Hippospongia lachne*[250]
**617b**	(+)-hippolide J	*Hippospongia lachne*[250]
**618**	sulawesin A	*Psammocinia* sp.[251]
**619**	sulawesin B	*Psammocinia* sp.[251]
**620a**	(+)-ircinin-1	*Psammocinia* sp.[251]
**620b**	(−)-ircinin-1	*Psammocinia* sp.[251]
**621**	ircinin-2	*Psammocinia* sp.[251]
**622a**	(+)-3-oxethyl-4-[1-(4,5-dibromopyrrole-2-yl)-formamido]-butanoic acid methyl ester	*Agelas* sp.[252]
**622b**	(−)-3-oxethyl-4-[1-(4,5-dibromopyrrole-2-yl)-formamido]-butanoic acid methyl ester	*Agelas* sp.[252]
**623a**	(+)-(9*S*,10*R*)-longamide D	*Agelas* sp.[252]
**623b**	(−)-(9*R*,10*R*)-longamide D	*Agelas* sp.[252]
**624a**	(+)-(*R*)-longamide F	*Agelas* sp.[252]
**624b**	(−)-(*S*)-longamide F	*Agelas* sp.[252]
**625a**	(+)-(*R*)-hanishin	*Agelas* sp.[252]
**625b**	(−)-(*S*)-hanishin	*Agelas* sp.[252]
**626a**	(+)-(*R*)-longamide B methyl ester	*Agelas* sp.[252]
**626b**	(−)-(*S*)-longamide B methyl ester	*Agelas* sp.[252]
**627a**	(+)-2-oxethyl-3-[1-(4,5-dibromopyrrole-2-yl)-formamido]-methyl propionate	*Agelas* sp.[252]
**627b**	(−)-2-oxethyl-3-[1-(4,5-dibromopyrrole-2-yl)-formamido]-methyl propionate	*Agelas* sp.[252]
**628a**	(+)-(*R*)-longamide E	*Agelas* sp.[252]
**628b**	(−)-(*S*)-longamide E	*Agelas* sp.[252]
**629a**	(+)-nemoechine B	*Agelas aff. Nemoechinata*[253]
**629b**	(−)-nemoechine B	*Agelas aff. Nemoechinata*[253]
**630a**	(+)-nemoechine C	*Agelas aff. Nemoechinata*[253]
**630b**	(−)-nemoechine C	*Agelas aff. Nemoechinata*[253]
**631a**	(+)-nemoechine A	*Agelas aff. Nemoechinata*[253]
**631b**	(−)-nemoechine A	*Agelas aff. Nemoechinata*[253]
**632a**	(+)-nakamurine D	*Agelas nakamurai*[254]
**632b**	(−)-nakamurine D	*Agelas nakamurai*[254]
**633a**	(+)-nakamurine E	*Agelas nakamurai*[254]
**633b**	(−)-nakamurine E	*Agelas nakamurai*[254]
**634a**	Methyl (*R*)-2-hydroxy-3-(1H-pyrrole-2-carboxamido)propanoate	*Agelas nakamurai*[254]
**634b**	Methyl (*S*)-2-hydroxy-3-(1H-pyrrole-2-carboxamido)propanoate	*Agelas nakamurai*[254]
**635a**	(+)-spiroreticulatine	*Fascaplysinopsis reticulate*[255]
**635b**	(−)-spiroreticulatine	*Fascaplysinopsis reticulate*[255]
**636a**	(+)-sponalisolide B	*Spongia officinalis*[248]

**636b**	(−)-sponalisolide B	*Spongia officinalis*[248]
**637a**	(−)-(3*R*,4*E*,16*E*,18*R*)-icosa-4,16-diene-1,19-diyne-3,18-diol	*Callyspongia* sp.[256]
**638b**	(+)-(3*S*,4*E*,16*E*,18*S*)-icosa-4,16-diene-1,19-diyne-3,18-diol	*Callyspongia* sp.[256]
**638a**	(*E*)-(2*R*,3*R*,4*S*)-2-amino-1,3-dihydroxyoctadec-6-ene-4-sulfate	*Spirastrella abata*[257]
**638b**	(*E*)-(2*S*,3*S*,4*R*)-2-amino-1,3-dihydroxyoctadec-6-ene-4-sulfate	*Spirastrella abata*[258]
**639a**	(*E*)-(2*R*,3*R*,4*S*)-2-amino-1,3-dihydroxyheptadec-6-ene-4-sulfate	*Spirastrella abata*[257]
**639b**	(*E*)-(2*S*,3*S*,4*R*)-2-amino-1,3-dihydroxyheptadec-6-ene-4-sulfate	*Spirastrella abata*[258]

Table S34. Names, source species and references of enantiomers from phyla Arthropoda and Chordata

No	Name	Species & Reference
**640a**	(+)-blapsol A	*Blaps japanensis*[259]
**640b**	(−)-blapsol A	*Blaps japanensis*[259]
**641a**	(+)-blapsol C	*Blaps japanensis*[259]
**641b**	(−)-blapsol C	*Blaps japanensis*[259]
**642a**	(+)-blapsol B	*Blaps japanensis*[259]
**642b**	(−)-blapsol B	*Blaps japanensis*[259]
**643a**	(+)-blapsol D	*Blaps japanensis*[259]
**643b**	(−)-blapsol D	*Blaps japanensis*[259]
**644a**	(2*R*,3*S*)-2-(3′,4′-dihydroxyphenyl)-3-acetylamino-7-(N-acetyl-2″-aminoethyl)-1,4-benzodioxane	*Aspongopus chinensis*[260]
**644b**	(2*S*,3*R*)-2-(3′,4′-dihydroxyphenyl)-3-acetylamino-7-(N-acetyl-2″-aminoethyl)-1,4-benzodioxane	*Aspongopus chinensis*[260]
**645a**	(±)-aspongamide A	*Aspongopus chinensis*[260]
**646a**	(+)-plancyamide B	*Polyphaga plancyi*[261]
**646b**	(−)-plancyamide B	*Polyphaga plancyi*[261]
**647a**	(+)-(*R*)-eudistidine C	*Eudistoma* sp.[262]
**647b**	(−)-(*S*)-eudistidine C	*Eudistoma* sp.[262]
**648a**	(+)-petromyroxol	*Petromyzon marinus*[263]
**648b**	(−)-Petromyroxol	*Petromyzon marinus*[263]

References

1. Liu X, Wang X B, Xie S S, Li Z R, Yang M H, Kong L Y, et al. Lignans from the root of Paeonia lactiflora and their anti-β-amyloid aggregation activities. *Fitoterapia.* 2015;**103**:136-142.
2. Zhao P, Zhang H, Han F-Y, Guo R, Huang S-W, Lin B, et al. Chiral resolution and neuroprotective activities of enantiomeric 8-O-4' neolignans from the fruits of Crataegus pinnatifida Bge. *Fitoterapia.* 2019;**136**:104164.
3. Zhou L, Lou L-L, Wang W, Lin B, Chen J-N, Wang X-B, et al. Enantiomeric 8-O-4' type neolignans from red raspberry as potential inhibitors of β-amyloid aggregation. *J Funct Foods.* 2017;**37**:322-329.
4. Du Y-Q, Yan Z-Y, Hou Z-L, Guo R, Bai M, Zhou L, et al. Enantiomeric 8,4'-type oxyneolignans from the root barks of Ailanthus altissima (Mill.) Swingle and their neuroprotective effects against H2O2-induced SH-SY5Y cells injury. *Fitoterapia.* 2019;**139**:104403.
5. Jiang C, Luo P, Zhao Y, Hong J, Morris-Natschke S L, Xu J, et al. Carolignans from the Aerial Parts of Euphorbia sikkimensis and Their Anti-HIV Activity. *J Nat Prod.* 2016;**79**:578-583.
6. Odonbayar B, Murata T, Buyankhishig B, Sasaki K, Suganuma K, Ishikawa Y, et al. Acylated Lignans Isolated from Brachanthemum gobicum and Their Trypanocidal Activity. *J Nat Prod.* 2019;**82**:774-784.
7. Lu Y, Xue Y, Liu J, Yao G, Li D, Sun B, et al. (±)-Acortatarinowins A-F, Norlignan, Neolignan, and Lignan Enantiomers from Acorus tatarinowii. *J Nat Prod.* 2015;**78**:2205-2214.
8. Qin D P, Feng X-L, Zhang W-Y, Gao H, Cheng X-R, Zhou W-X, et al. Anti-neuroinflammatory asarone derivatives from the rhizomes of Acorus tatarinowii. *RSC Adv.* 2017;**7**:8512-8520.
9. Fang Y-S, Cai L, Wang J-P, Yin T-P, Yu J, Ding Z-T, et al. New phenylpropanoids from Bulbophyllum retusiusculum. *Arch Pharm Res.* 2018;**41**:1074-1081.
10. Cheng Z-B, Lu X, Bao J-M, Han Q-H, Dong Z, Tang G-H, et al. (±)-Torreyunlignans A-D, Rare 8-9' Linked Neolignan Enantiomers as Phosphodiesterase-9A Inhibitors from Torreya yunnanensis. *J Nat Prod.* 2014;**77**:2651-2657.
11. Yang D-T, Lin S-S, Chen J-H, Yuan S-T, Shi J-S, Wang J-S, et al. (+)- and (-)-liriodenol, a pair of novel enantiomeric lignans from Liriodendron hybrid. *Bioorg Med Chem Lett.* 2015;**25**:1976-1978.
12. Zhu Y, Huang R-Z, Wang C-G, Ouyang X-L, Jing X-T, Liang D, et al. New inhibitors of matrix metalloproteinases 9 (MMP-9): Lignans from Selaginella moellendorffii. *Fitoterapia.* 2018;**130**:281-289.
13. Jiao S, Su G, Zhou X, Wuken S, Li J, Tu P, et al. Alashinols I and J, two novel phenols from stem barks of Syringa pinnatifolia. *Phytochem Lett.* 2019;**33**:61-63.
14. Lu Y, Xue Y, Liu J, Yao G, Li D, Sun B, et al. (±)-Acortatarinowins A-F, norlignan, neolignan, and lignan enantiomers from Acorus tatarinowii. *J Nat Prod.* 2015;**78**:2205-2214.
15. Lu Y, Xue Y, Chen S, Zhu H, Wang J, Liu J, et al. Antioxidant Lignans and Neolignans from Acorus tatarinowii. *Sci Rep.* 2016;**6**:22909.
16. Zhu J-Y, Cheng B, Zheng Y-J, Dong Z, Lin S-L, Tang G-H, et al. Enantiomeric neolignans

and sesquineolignans from Jatropha integerrima and their absolute configurations. *RSC Adv.* 2015;**5**:12202-12208.

17. Lou L-L, Yao G-D, Wang J, Zhao W-Y, Wang X-B, Huang X-X, et al. Enantiomeric neolignans from Picrasma quassioides exhibit distinctive cytotoxicity on hepatic carcinoma cells through ROS generation and apoptosis induction. *Bioorg Med Chem Lett.* 2018;**28**:1263-1268.
18. Zhou L, Yao G-D, Lu L-W, Song X-Y, Lin B, Song S-J, et al. Neolignans from Red Raspberry (Rubus idaeus L.) Exhibit Enantioselective Neuroprotective Effects against H2O2-Induced Oxidative Injury in SH-SY5Y Cells. *J Agric Food Chem.* 2018;**66**:11390-11397.
19. Zhou L, Xi Y-F, Wang W, Lin B, Wang X-B, Huang X-X, et al. Chiral resolution and bioactivity of enantiomeric benzofuran neolignans from the fruit of Rubus ideaus L. *Fitoterapia.* 2018;**127**:56-61.
20. Wu Z, Zhou L, Wu Y, Zhu H, Hu Z, Wang J, et al. Enantiomeric Lignans and Neolignans from Phyllanthus glaucus: Enantioseparation and Their Absolute Configurations. *Sci Rep.* 2016;**6**:24809.
21. Zhou L, Han F-Y, Lu L-W, Yao G-D, Zhang Y-Y, Wang X-B, et al. Isolation of enantiomeric furolactones and furofurans from Rubus idaeus L. with neuroprotective activities. *Phytochemistry (Elsevier).* 2019;**164**:122-129.
22. Wang Y-X, Lin B, Zhou L, Yan Z-Y, Zhang H, Huang X-X, et al. Anti-β-amyloid aggregation activity of enantiomeric furolactone-type lignans from Archidendron clypearia (Jack) I.C.N. *Nat Prod Res.* 2018;doi: 10.1080/14786419.2018.1488705.
23. Zhou X-M, Zheng C-J, Wu J-T, Chen G-Y, Chen J and Sun C-G. Five new lactone derivatives from the stems of Dendrobium nobile. *Fitoterapia.* 2016;**115**:96-100.
24. Liu W-J, Chen Y-J, Chen D-N, Wu Y-P, Gao Y-J, Li J, et al. A new pair of enantiomeric lignans from the fruits of Morinda citrifolia and their absolute configuration. *Nat Prod Res.* 2018;**32**:933-938.
25. Wang Y-X, Zhou L, Wang J, Lin B, Wang X-B, Huang X-X, et al. Enantiomeric lignans with anti-β-amyloid aggregation activity from the twigs and leaves of Pithecellobium clypearia Benth. *Bioorg Chem.* 2018;**77**:579-585.
26. Xi Y-F, Liu S-F, Hong W, Song X-Y, Lou L-L, Zhou L, et al. Discovery of cycloneolignan enantiomers from Isatis indigotica Fortune with neuroprotective effects against MPP+-induced SH-SY5Y cell injury. *Bioorg Chem.* 2019;**88**:102926.
27. Fukuda T, Nagai K and Tomoda H. (±)-Tylopilusins, Diphenolic Metabolites from the Fruiting Bodies of Tylopilus eximius. *J Nat Prod.* 2012;**75**:2228-2231.
28. Lai Y, Liu T, Sa R, Wei X, Xue Y, Wu Z, et al. Neolignans with a Rare 2-Oxaspiro[4.5]deca-6,9-dien-8-one Motif from the Stem Bark of Cinnamomum subavenium. *J Nat Prod.* 2015;**78**:1740-1744.
29. Shi Y, Liu Y, Li Y, Li L, Qu J, Ma S, et al. Chiral resolution and absolute configuration of a pair of rare racemic spirodienone sesquineolignans from Xanthium sibiricum. *Org Lett.* 2014;**16**:5406-5409.
30. Hong M J and Kim J. Determination of the Absolute Configuration of Khellactone Esters from Peucedanum japonicum Roots. *J Nat Prod.* 2017;**80**:1354-1360.
31. Hata K, Kozawa M, Baba K, Yen K-Y and Yang L-L. Coumarins from the roots of Angelica

morii. *Che Pharm Bull.* 1974;**22**:957-961.
32. Xiong Y-Y, Wu F-H, Wang J-S, Li J and Kong L-Y. Attenuation of airway hyperreactivity and T helper cell type 2 responses by coumarins from Peucedanum praeruptorum Dunn in a murine model of allergic airway inflammation. *J Ethnopharmacol.* 2012;**141**:314-321.
33. Nielsen B E, Larsen P K and Lemmich J. Constituents of umbelliferous plants. XVII. Coumarins from seseli gummiferum. Structure of two new coumarins. *Acta Chem Scand.* 1971;**25**:529-533.
34. Matsuda H, Murakami T, Nishida N, Kageura T and Yoshikawa M. Medicinal foodstuffs. XX. Vasorelaxant active constituents from the roots of Angelica furcijuga Kitagawa: structures of hyuganins A, B, C, and D. *Chem Pharm Bull.* 2000;**48**:1429-1435.
35. Song Y-L, Jing W-H, Tu P-F and Wang Y-T. Enantiomeric separation of angular-type pyranocoumarins from Peucedani Radix using AD-RH chiral column. *Nat Prod Res.* 2014;**28**:545-550.
36. Górski B, Talko A, Basak T and Barbasiewicz M. Olefination with Sulfonyl Halides and Esters: Scope, Limitations, and Mechanistic Studies of the Hawkins Reaction. *Org Lett.* 2017;**19**:1756-1759.
37. Li T, Wang S, Lou H and Fan P. New coumarins and monoterpene galloylglycoside from the stem bark of Sapium baccatum. *Fitoterapia.* 2019;**134**:435-442.
38. Chen L, Tang G-H, Guo F-L, Li W, Zhang J-S, Liu B, et al. (P)/(M)-corinepalensin A, a pair of axially chiral prenylated bicoumarin enantiomers with a rare C-5C-5' linkage from the twigs of Coriaria nepalensis. *Phytochemistry.* 2018;**149**:140-145.
39. Su F, Zhao Z, Ma S, Wang R, Li Y, Liu Y, et al. Cnidimonins A-C, Three Types of Hybrid Dimer from Cnidium monnieri: Structural Elucidation and Semisynthesis. *Org Lett.* 2017;**19**:4920-4923.
40. Tang Z-H, Liu Y-B, Ma S-G, Li L, Li Y, Jiang J-D, et al. Antiviral Spirotriscoumarins A and B: Two Pairs of Oligomeric Coumarin Enantiomers with a Spirodienone-Sesquiterpene Skeleton from Toddalia asiatica. *Org Lett.* 2016;**18**:5146-5149.
41. Shi X-L, Yan J-K, Li W-K, Donkor P O, Gao X-M, Ding L-Q, et al. Two pairs of phenylpropanoid enantiomers from the leaves of Eucommia ulmoides. *J Asian Nat Prod. Res.* 2018;**20**:1045-1054.
42. Bai L, Wang Y, Ge Y, Liu J and Luan X. Diastereoselective Synthesis of Dibenzo[b,d]azepines by Pd(II)-Catalyzed [5 + 2] Annulation of o-Arylanilines with Dienes. *Org Lett.* 2017;**19**:1734-1737.
43. Lu Y-Y, Gong X-P, Xue Y-B, Zhu H-C, Li X-N, Hu L-Z, et al. Two pairs of chlorine-containing phenylpropanoid enantiomers from Acorus tatarinowii. *Chin Chem Lett.* 2017;**28**:1460-1464.
44. Gao E, Zhou Z-Q, Zou J, Yu Y, Feng X-L, Chen G-D, et al. Bioactive Asarone-Derived Phenylpropanoids from the Rhizome of Acorus tatarinowii Schott. *J Nat Prod.* 2017;**80**:2923-2929.
45. Zhou L, Yao G-D, Song X-Y, Wang J, Lin B, Wang X-B, et al. Neuroprotective Effects of 1,2-Diarylpropane Type Phenylpropanoid Enantiomers from Red Raspberry against H2O2-Induced Oxidative Stress in Human Neuroblastoma SH-SY5Y Cells. *J Agric Food Chem.* 2018;**66**:331-338.
46. Yan J-K, Shi X-L, Donkor P O, Gao X-M, Ding L-Q, Qiu F, et al. Two pairs of phenolic

enantiomers from the leaves of Eucommia ulmoides Oliver. *Nat Prod Res.* 2019;**33**:1162-1168.

47. Guo R, Shang X-Y, Lv T-M, Yao G-D, Lin B, Wang X-B, et al. Phenylpropanoid derivatives from the fruit of Crataegus pinnatifida Bunge and their distinctive effects on human hepatoma cells. *Phytochemistry (Elsevier).* 2019;**164**:252-261.
48. Xi Y-F, Lou L-L, Xu Z-Y, Hou Z-L, Wang X-B, Huang X-X, et al. Alkaloid Enantiomers from Isatis tinctoria with Neuroprotective Effects against H 2 O 2 -Induced SH-SY5Y Cell Injury. *Planta Med.* 2019;**85**:1374-1382.
49. Liu J, Du Y-Q, Li C-J, Li L, Chen F-Y, Yang J-Z, et al. Alkaloids from the stems of Clausena lansium and their neuroprotective activity. *RSC Adv.* 2017;**7**:35417-35425.
50. Li Q, Deng A-J, Li L, Wu L-Q, Ji M, Zhang H-J, et al. Azacyclo-indoles and Phenolics from the Flowers of Juglans regia. *J Nat Prod* 2017;**80**:2189-2198.
51. Liu S-F, Lin B, Xi Y-F, Zhou L, Lou L-L, Huang X-X, et al. Bioactive spiropyrrolizidine oxindole alkaloid enantiomers from Isatis indigotica Fortune. *Org Biomol Chem.* 2018;**16**:9430-9439.
52. Zhang D, Shi Y, Xu R, Du K, Guo F, Chen K, et al. Alkaloid enantiomers from the roots of Isatis indigotica. *Molecules.* 2019;**24**:3140.
53. Guo Q, Xu C, Chen M, Lin S, Zhu C, Jiang J, et al. Sulfur-enriched alkaloids from the root of Isatis indigotica. *Acta Pharm Sin B.* 2018;**8**:933-943.
54. Chen M, Lin S, Li L, Zhu C, Wang X, Wang Y, et al. Enantiomers of an Indole Alkaloid Containing Unusual Dihydrothiopyran and 1,2,4-Thiadiazole Rings from the Root of Isatis indigotica. *Org Lett.* 2012;**14**:5668-5671.
55. Nge C-E, Chong K-W, Thomas N F, Lim S-H, Low Y-Y and Kam T-S. Ibogan, aspidosperman, vincamine, and bisindole alkaloids from a Malayan Tabernaemontana corymbosa: Iboga alkaloids with C-20α substitution. *J Nat Prod.* 2016;**79**:1388-1399.
56. Zhang D B, Yu D G, Sun M, Zhu X X, Yao X J, Zhou S Y, et al. Ervatamines A-I, Anti-inflammatory Monoterpenoid Indole Alkaloids with Diverse Skeletons from Ervatamia hainanensis. *J Nat Prod.* 2015;**78**:1253-1261.
57. Li D-W, Guo Q-L, Meng X-H, Zhu C-G, Xu C-B and Shi J-G. Two pairs of unusual scalemic enantiomers from Isatis indigotica leaves. *Chin Chem Lett.* 2016;**27**:1745-1750.
58. Li Y-H, Zhang Y, Peng L-Y, Li X-N, Zhao Q-S, Li R-T, et al. (±)-Evodiakine, A Pair of Rearranged Rutaecarpine-Type Alkaloids From Evodia rutaecarpa. *Nat Prod Bioprospect.* 2016;**6**:291-296.
59. Geng C-A, Huang X-Y, Ma Y-B, Hou B, Li T-Z, Zhang X-M, et al. (±)-Uncarilins A and B, dimeric isoechinulin-type alkaloids from Uncaria rhynchophylla. *J Nat Prod.* 2017;**80**:959-964.
60. Chen M, Gan L, Lin S, Wang X, Li L, Li Y, et al. Alkaloids from the root of Isatis indigotica. *J Nat Prod.* 2012;**75**:1167-1176.
61. Zhao L-N, Guo X-X, Liu S, Feng L, Bi Q-R, Wang Z, et al. (±)-Zanthonitidine A, a Pair of Enantiomeric Furoquinoline Alkaloids from Zanthoxylum nitidum with Antibacterial Activity. *Nat Prod Bioprospect.* 2018;**8**:361-367.
62. Liu Y, Wang X, Lin S, Li L, Shi J and Chen M. Three pairs of alkaloid enantiomers from the root of Isatis indigotica. *Acta Pharm Sin B.* 2016;**6**:141-147.
63. Zhang J, Zhang Q-Y, Tu P-F, Liang H and Xu F-C. Mucroniferanines A-G, Isoquinoline

Alkaloids from Corydalis mucronifera. *J Nat Prod.* 2018;**81**:364-370.
64. Yin X, Bai R, Guo Q, Su G, Wang J, Yang X, et al. Hendersine A, a novel isoquinoline alkaloid from Corydalis hendersonii. *Tetrahedron Lett.* 2016;**57**:4858-4862.
65. Sai C-M, Li D-H, Li S-G, Han T, Guo Y-Z, Pei Y-H, et al. Racemic alkaloids from Macleaya cordata: structural elucidation, chiral resolution, and cytotoxic, antibacterial activities. *RSC Adv.* 2016;**6**:41173-41180.
66. Yang Z, Liu Z, Han N, Jiang B, Guo D, Teng F, et al. Ambidalmines A-E and ambidimerine F: bioactive dihydrobenzophenanthridine alkaloids from Corydalis ambigua var. amurensis. *Eur J Med Chem.* 2014;**84**:417-424.
67. Wang L, Zhang S-Y, Chen L, Huang X-J, Zhang Q-W, Jiang R-W, et al. New enantiomeric isoquinoline alkaloids from Coptis chinensis. *Phytochem Lett.* 2014;**7**:89-92.
68. Sai C-M, Li D-H, Xue C-M, Wang K-B, Hu P, Pei Y-H, et al. Two Pairs of Enantiomeric Alkaloid Dimers from Macleaya cordata. *Org Lett.* 2015;**17**:4102-4105.
69. Zhao W-Y, Zhou W-Y, Chen J-J, Yao G-D, Lin B, Wang X-B, et al. Enantiomeric β-carboline dimers from Picrasma quassioides and their anti-hepatoma potential. *Phytochemistry.* 2019;**159**:39-45.
70. Guo X-M, Li F, Zheng F-F, Gong N-N, Li Y, Feng W-Z, et al. (±)-Quassidine K, a pair of cytotoxic bis-β-carboline alkaloid enantiomers from Picrasma quassioides. *Nat Prod Res.* 2018:Ahead of Print.
71. Jiao W-H, Chen G-D, Gao H, Li J, Gu B-B, Xu T-T, et al. (±)-Quassidines I and J, two pairs of cytotoxic bis-β-carboline alkaloid enantiomers from Picrasma quassioides. *J Nat Prod.* 2014;**77**:2707-2712.
72. Wang K-B, Li S-G, Huang X-Y, Li D-H, Li Z-L and Hua H-M. (±)-Peharmaline A: A Pair of Rare β-Carboline-Vasicinone Hybrid Alkaloid Enantiomers from Peganum harmala. *Eur J Org Chem.* 2017;**2017**:1876-1879.
73. Liu Y, Yu H-Y, Xu H-Z, Liu J-J, Meng X-G, Zhou M, et al. Alkaloids with Immunosuppressive Activity from the Bark of Pausinystalia yohimbe. *J Nat Prod.* 2018;**81**:1841-1849.
74. Cao N, Chen Y, Ma X, Zeng K, Zhao M, Tu P, et al. Bioactive carbazole and quinoline alkaloids from Clausena dunniana. *Phytochemistry.* 2018;**151**:1-8.
75. Ma X-L, Li J, Zheng J, Gu X-P, Ferreira D, Zjawiony J K, et al. LC-MS-guided isolation of insulin-secretion-promoting monoterpenoid carbazole alkaloids from Murraya microphylla. *J Nat Prod.* 2018;**81**:2371-2380.
76. Ma X, Cao N, Zhang C, Guo X, Zhao M, Tu P, et al. Cytotoxic carbazole alkaloid derivatives from the leaves and stems of Murraya microphylla. *Fitoterapia.* 2018;**127**:334-340.
77. Chen Q-B, Gao J, Zou G-A, Xin X-L and Aisa H A. Piperidine Alkaloids with Diverse Skeletons from Anacyclus pyrethrum. *J Nat Prod.* 2018;**81**:1474-1482.
78. Chen Q-B and Aisa H A. Alkaloid constituents from Viola tianschanica. *Phytochemistry (Elsevier).* 2017;**144**:233-242.
79. Song W-W, Zeng G-Z, Peng W-W, Chen K-X and Tan N-H. Cytotoxic amides and quinolones from Clausena lansium. *Helv Chim Acta.* 2014;**97**:298-305.
80. Yu M-Y, Qin X-J, Peng X-R, Wang X, Tian X-X, Li Z-R, et al. Macathiohydantoins B-K, novel thiohydantoin derivatives from Lepidium meyenii. *Tetrahedron.* 2017;**73**:4392-4397.
81. Al-Khdhairawi A A Q, Krishnan P, Lim K-H, Mai C-W, Leong C-O, Chung F F-L, et al. A

Bis-benzopyrroloisoquinoline Alkaloid Incorporating a Cyclobutane Core and a Chlorophenanthroindolizidine Alkaloid with Cytotoxic Activity from Ficus fistulosa var. tengerensis. *J Nat Prod.* 2017;**80**:2734-2740.
82. Stoye A, Peez T E and Opatz T. Left, Right, or Both? On the Configuration of the Phenanthroindolizidine Alkaloid Tylophorine from Tylophora indica. *J Nat Prod.* 2013;**76**:275-278.
83. Hu Y, Zhang C, Zhao X, Wang Y, Feng D, Zhang M, et al. (±)-Homocrepidine A, a Pair of Anti-inflammatory Enantiomeric Octahydroindolizine Alkaloid Dimers from Dendrobium crepidatum. *J Nat Prod.* 2016;**79**:252-256.
84. Zhang H, Zhu K-K, Gao X-H and Yue J-M. Natural occurrence of all eight stereoisomers of a neosecurinane structure from Flueggea virosa. *Tetrahedron.* 2017;**73**:4692-4697.
85. Li S-G, Wang K-B, Gong C, Bao Y, Qin N-B, Li D-H, et al. Cytotoxic quinazoline alkaloids from the seeds of Peganum harmala. *Bioorg Med Chem Lett.* 2018;**28**:103-106.
86. Yang Y-d, Cheng X-m, Liu W, Han Z-z, Chou G-x, Wang Y, et al. Peganumine B-I and two enantiomers: new alkaloids from the seeds of Peganum harmala Linn. and their potential cytotoxicity and cholinesterase inhibitory activities. *RSC Adv.* 2016;**6**:15976-15987.
87. Shou Q, Banbury L K, Renshaw D E, Smith J E, He X, Dowell A, et al. Parvifloranines A and B, Two 11-Carbon Alkaloids from Geijera parviflora. *J Nat Prod.* 2013;**76**:1384-1387.
88. Xia G-Y, Owusu D P, Ding L-Q, Qiu F, Xia G-Y, Sun D-J, et al. (+)/(-)-Phaeocaulin A-D, four pairs of new enantiomeric germacrane-type sesquiterpenes from Curcuma phaeocaulis as natural nitric oxide inhibitors. *Sci Rep.* 2017;**7**:43576.
89. Han Q-T, Li G-S, Xiang K-L, Ren Y and Dai S-J. Flavonoid alkaloids from Scutellaria moniliorrhiza with anti-inflammatory activities and inhibitory activities against aldose reductase. *Phytochemistry.* 2018;**152**:91-96.
90. Zhou D, Chen G, Ma Y-P, Wang C-G, Lin B, Yang Y-Q, et al. Isolation, structural elucidation, optical resolution, and antineuroinflammatory activity of phenanthrene and 9,10-dihydrophenanthrene derivatives from Bletilla striata. *J Nat Prod.* 2019;**82**:2238-2245.
91. Cheng Z-Y, Du Y-Q, Zhang Q, Lin B, Gao P-Y, Huang X-X, et al. Two pairs of new alkaloid enantiomers with a spiro [benzofuranone-benzazepine] skeleton from the bark of Juglans mandshurica. *Tetrahedron Lett.* 2018;**59**:2050-2053.
92. Chen F, Huang X-j, Liang Q-p, Huang Y-p, Lan T and Zhou G-x. Three new lignanamides from the root of Lycium chinense with anti-inflammatory activity. *Nat Prod Res.* 2019;**33**:3378-3382.
93. Zhu G-Y, Yang J, Yao X-J, Yang X, Fu J, Liu X, et al. (±)-Sativamides A and B, two pairs of racemic nor-lignanamide enantiomers from the fruits of Cannabis sativa. *J Org Chem.* 2018;**83**:2376-2381.
94. Azmi M N, Chan G, Peresse T, Remeur C, Roussi F, Litaudon M, et al. Kingianins O-Q: Pentacyclic polyketides from Endiandra kingiana as inhibitor of Mcl-1/Bid interaction. *Fitoterapia.* 2016;**109**:190-195.
95. Xie Y, Xu P-S, Xu K-P, Zou Z-X, Zhou G, Li D, et al. Two new biflavanoids from Selaginella trichoclada Alsto. *Nat Prod Res.* 2019:Ahead of Print.
96. Li R, Cheng J, Jiao M, Guo C, Chen S, Li L, et al. New phenylpropanoid-substituted flavan-3-ols and flavonols from the leaves of Uncaria rhynchophylla. *Fitoterapia.* 2017;**116**:17-23.

97. Zhang T-Y, Lin J-B, Li Q-Z, Kang J-C, Pan J-L, Hou S-H, et al. Copper-Catalyzed Selective ortho-C–H/N–H Annulation of Benzamides with Arynes: Synthesis of Phenanthridinone Alkaloids. *Org Lett.* 2017;**19**:1764-1767.
98. Zaki M A, Hetta M H, Mohammed R, Nanayakkara N P D, Jacob M R, Khan S I, et al. Bioactive Formylated Flavonoids from Eugenia rigida: Isolation, Synthesis, and X-ray Crystallography. *J Nat Prod.* 2016;**79**:2341-2349.
99. Li Y, Qin X-B, Liu H-X, Xu Z-F, Tan H-B and Qiu S-X. Two pairs of enantiomeric propylated flavonoids and a new lignan from the aerial parts of Abrus precatorius. *Fitoterapia.* 2019;**133**:125-129.
100. He Q-F, Wu Z-L, Huang X-J, Zhong Y-L, Jiang R-W, Li Y-L, et al. Cajanusflavanols A-C, Three Pairs of Flavonostilbene Enantiomers from Cajanus cajan. *Org Lett.* 2018;**20**:876-879.
101. Xu L, Huang T, Huang C, Wu C, Jia A and Hu X. Chiral separation, absolute configuration, and bioactivity of two pairs of flavonoid enantiomers from Morus nigra. *Phytochemistry (Elsevier).* 2019;**163**:33-37.
102. Hu X-q, Liu Q-x, Li H-l and Han W. Flavane constituents from branch of Celastrus hindsii. *Zhongcaoyao.* 2014;**45**:2132-2135.
103. Li F-F, Sun Q, Wang D, Liu S, Lin B, Liu C-T, et al. Chiral Separation of Cytotoxic Flavan Derivatives from Daphne giraldii. *J Nat Prod.* 2016;**79**:2236-2242.
104. Pang D-R, Su X-Q, Sun J, Li Y-T, Zhu Z-X, Song Y-L, et al. Flavonoid dimers from the total phenolic extract of Chinese dragon's blood, the red resin of Dracaena cochinchinensis. *Fitoterapia.* 2016;**115**:135-141.
105. Hiep N T, Lee D, Hiep N T, Kwon J, Hong S, Mar W, et al. Enantiomeric Isoflavones with neuroprotective activities from the Fruits of Maclura tricuspidata. *Sci Rep.* 2019;**9**:1757.
106. Liu Z, Zheng X, Wang Y, Tang M, Chen S, Zhang F, et al. Lignans and isoflavonoids from the stems of Pisonia umbellifera. *RSC Adv.* 2018;**8**:16383-16391.
107. Shi Y-S, Hu W-Z, Zhang X-F, Lv X, Shi Y-S, Zhang Y, et al. Dihydrochalcones and Diterpenoids from Pteris ensiformis and Their Bioactivities. *Molecules.* 2017;**22**: doi:10.3390/molecules22091413.
108. Liu Y, Zhang X, Kelsang N, Tu G, Kong D, Lu J, et al. Structurally diverse cytotoxic dimeric chalcones from Oxytropis chiliophylla. *J Nat Prod.* 2018;**81**:307-315.
109. Simard F, Gauthier C, Chiasson E, Lavoie S, Mshvildadze V, Legault J, et al. Antibacterial balsacones J-M, hydroxycinnamoylated dihydrochalcones from Populus balsamifera buds. *J Nat Prod.* 2015;**78**:1147-1153.
110. Ma Q, Min K, Li H-L, Jiang J-H, Liu Y, Zhan R, et al. Horsfiequinones A-F, Dimeric Diarylpropanoids from Horsfieldia tetratepala. *Planta Med.* 2014;**80**:688-694.
111. Jia C, Gong C, Pu J, Li D, Li Z, Hua H, et al. A pair of new enantiomers of xanthones from the stems and leaves of Cratoxylum cochinchinense. *Chin Med.* 2019;**14**:14.
112. Macabeo A P G, Martinez F P A, Kurtan T, Toth L, Mandi A, Schmidt S, et al. Tetrahydroxanthene-1,3(2H)-dione derivatives from Uvaria valderramensis. *J Nat Prod.* 2014;**77**:2717-2721.
113. Boonnak N, Chantrapromma S, Fun H-K, Yuenyongsawad S, Patrick B O, Maneerat W, et al. Three types of cytotoxic natural caged-scaffolds: pure enantiomers or partial racemates. *J Nat Prod.* 2014;**77**:1562-1571.
114. Niu S-L, Li D-H, Li X-Y, Wang Y-T, Li S-G, Bai J, et al. Bioassay- and chemistry-guided

isolation of scalemic caged prenylxanthones from the leaves of Garcinia bracteata. *J Nat Prod.* 2018;**81**:749-757.
115. Sriyatep T, Andersen R J, Patrick B O, Pyne S G, Muanprasat C, Seemakhan S, et al. Scalemic Caged Xanthones Isolated from the Stem Bark Extract of Garcinia propinqua. *J Nat Prod.* 2017;**80**:1658-1667.
116. Xi F-M, Ma S-G, Liu Y-B, Li L and Yu S-S. Artaboterpenoids A and B, Bisabolene-Derived Sesquiterpenoids from Artabotrys hexapetalus. *Org Lett.* 2016;**18**:3374-3377.
117. Yan J, Shi X, Donkor P O, Qiu F, Yan J, Shi X, et al. Nine pairs of megastigmane enantiomers from the leaves of Eucommia ulmoides Oliver. *J Nat Med.* 2017;**71**:780-790.
118. Zhang R, Feng X, Su G, Mu Z, Zhang H, Zhao Y, et al. Bioactive Sesquiterpenoids from the Peeled Stems of Syringa pinnatifolia. *J Nat Prod.* 2018;**81**:1711-1720.
119. Ge C-Y, Zhang J-L, Ge C-Y and Zhang J-L. Bioactive sesquiterpenoids and steroids from the resinous exudates of Commiphora myrrha. *Nat Prod Res.* 2019;**33**:309-315.
120. Zhang C-Y, Song Y-N, Zhang L-J, Zhang M, Ye Y, Zhang H, et al. Sesquiterpenes and lignans from the flower buds of Daphne genkwa and their nitric oxide inhibitory activities. *Nat Prod Res.* 2018;**32**:2893-2899.
121. Dong K, Pu J-X, Du X, Li X-N and Sun H-D. Two new guaianolide-type sesquiterpenoids from Kadsura interior. *Chin Chem Lett.* 2013;**24**:111-113.
122. Zhang W-Y, Gao K, Yue J-M, Zhao J-X, Sheng L, Fan Y-Y, et al. Mangelonoids A and B, Two Pairs of Macrocyclic Diterpenoid Enantiomers from Croton mangelong. *Org Lett.* 2018;**20**:4040-4043.
123. Feng L, Mandi A, Tang C, Kurtan T, Tang S, Ke C-Q, et al. A Pair of Enantiomeric Bis-seco-abietane Diterpenoids from Cryptomeria fortunei. *J Nat Prod.* 2018;**81**:2667-2672.
124. Li L-Z, Sun X, Qi X-L, Song S-J, Liang X, Wang J, et al. Bioactive norditerpenoids and neolignans from the roots of salvia miltiorrhiza. *Org Biomol Chem.* 2016;**14**:10050-10057.
125. Jiang Y-J, Zhang Y, He J, Wu X-D, Shao L-D, Li X-N, et al. (±)-Salviaprione, a pair of unprecedented abietane-type diterpenoids from Salvia prionitis. *Tetrahedron Lett.* 2015;**56**:5457-5459.
126. Geng C-A, Chen X-L, Zhou N-J, Chen H, Ma Y-B, Huang X-Y, et al. LC-MS Guided Isolation of (±)-Sweriledugenin A, a Pair of Enantiomeric Lactones from Swertia leducii. *Org Lett.* 2014;**16**:370-373.
127. Liang W-J, Geng C-A, Zhang X-M, Chen H, Yang C-Y, Rong G-Q, et al. (±)-Paeoveitol, a Pair of New Norditerpene Enantiomers from Paeonia veitchii. *Org Lett.* 2014;**16**:424-427.
128. Hu L, Zhu H, Li L, Huang J, Sun W, Liu J, et al. (±)-Japonones A and B, two pairs of new enantiomers with anti-KSHV activities from Hypericum japonicum. *Sci Rep.* 2016;**6**:27588.
129. Huang G-H, Hu Z, Lei C, Wang P-P, Yang J, Li J-Y, et al. Enantiomeric Pairs of Meroterpenoids with Diverse Heterocyclic Systems from Rhododendron nyingchiense. *J Nat Prod.* 2018;**81**:1810-1818.
130. Liao H-B, Huang G-H, Yu M-H, Lei C and Hou A-J. Five Pairs of Meroterpenoid Enantiomers from Rhododendron capitatum. *J Org Chem.* 2017;**82**:1632-1637.
131. Liao H-B, Lei C, Gao L-X, Li J-Y, Li J and Hou A-J. Two Enantiomeric Pairs of Meroterpenoids from Rhododendron capitatum. *Org Lett.* 2015;**17**:5040-5043.
132. Wang X, Li L, Zhu R, Zhang J, Zhou J and Lou H. Bibenzyl-based meroterpenoid enantiomers from the Chinese liverwort Radula sumatrana. *J Nat Prod.* 2017;**80**:3143-3150.

133. Li C, Li C-J, Ma J, Huang J-W, Wang X-Y, Wang X-L, et al. Magmenthanes A-H: Eight new meroterpenoids from the bark of Magnolia officinalis var. Biloba. *Bioorg Chem.* 2019;**88**:102948.
134. Cheng M-J, Yang X-Y, Cao J-Q, Liu C, Zhong L-P, Wang Y, et al. Isolation, structure elucidation, and total synthesis of myrtuspirone A from Myrtus communis. *Org Lett.* 2019;**21**:1583-1587.
135. Tantapakul C, Maneerat W, Sripisut T, Ritthiwigrom T, Andersen R J, Cheng P, et al. New Benzophenones and Xanthones from Cratoxylum sumatranum ssp. neriifolium and Their Antibacterial and Antioxidant Activities. *J Agric Food Chem.* 2016;**64**:8755-8762.
136. Hu L, Xue Y, Zhang J, Zhu H, Chen C, Li X-N, et al. (±)-Japonicols A-D, Acylphloroglucinol-Based Meroterpenoid Enantiomers with Anti-KSHV Activities from Hypericum japonicum. *J Nat Prod.* 2016;**79**:1322-1328.
137. Cheenpracha S, Pyne S G, Patrick B O, Andersen R J, Maneerat W and Laphookhieo S. Mallopenins A-E, antibacterial phenolic derivatives from the fruits of Mallotus philippensis. *J Nat Prod.* 2019;**82**:2174-2180.
138. Hans M, Charpentier M, Huch V, Jauch J, Bruhn T, Bringmann G, et al. Stereoisomeric Composition of Natural Myrtucommulone A. *J Nat Prod.* 2015;**78**:2381-2389.
139. Liu F, Tian H-Y, Huang X-L, Wang W-J, Li N-P, He J, et al. Xanthchrysones A-C: Rearranged Phenylpropanoyl-Phloroglucinol Dimers with Unusual Skeletons from Xanthostemon chrysanthus. *J Org Chem.* 2019;**84**:15355−15361.
140. Su J-C, Wang S, Cheng W, Huang X-J, Li M-M, Jiang R-W, et al. Phloroglucinol Derivatives with Unusual Skeletons from Cleistocalyx operculatus and Their in Vitro Antiviral Activity. *J Org Chem.* 2018;**83**:8522-8532.
141. Oya A, Tanaka N, Kusama T, Kim S-Y, Hayashi S, Kojoma M, et al. Prenylated Benzophenones from Triadenum japonicum. *J Nat Prod.* 2015;**78**:258-264.
142. Delle Monache F, Delle Monache G, Pinheiro R M and Radics L. Chemistry of Clusia genus. Part 3. Nemorosonol, a derivative of tricyclo-[4.3.1.03,7]-decane-7-hydroxy-2,9-dione from Clusia nemorosa. *Phytochemistry.* 1988;**27**:2305-2308.
143. Fan Y-M, Yi P, Li Y, Yan C, Huang T, Gu W, et al. Two Unusual Polycyclic Polyprenylated Acylphloroglucinols, Including a Pair of Enantiomers from Garcinia multiflora. *Org Lett.* 2015;**17**:2066-2069.
144. Tian D S, Yi P, Xia L, Xiao X, Fan Y M, Gu W, et al. Garmultins A-G, biogenetically related polycyclic Acylphloroglucinols from Garcinia multiflora. *Org Lett.* 2016;**18**:5904-5907.
145. Yu J-H, Zhai H-J, Yu Z-P, Zhang Q-Q, Ge Y-X, Zhang Y-Y, et al. Methyl 2-naphthoates from a traditional Chinese herb Morinda officinalis var. officinalis. *Tetrahedron.* 2019;**75**:3793-3801.
146. Li Q, Deng A-J, Qin H-L, Ji M, Li Z-H and Chen X-G. Racemic 3,4-dihydro-4-naphthyl-naphthalen-1(2H)-ones from Juglans regia flowers. *Fitoterapia.* 2019;**139**:104401.
147. Zhao S-M, Wang Z, Chen X-Q, Huang M-B and Tan N-H. (±)-Rubioncolin D, a pair of enantiomeric naphthohydroquinone dimers from Rubia oncotricha. *Tetrahedron Lett.* 2017;**58**:3041-3043.
148. Zhao S-M, Wang Z, Zeng G-Z, Song W-W, Chen X-Q, Li X-N, et al. New cytotoxic naphthohydroquinone dimers from Rubia alata. *Org Lett.* 2014;**16**:5576-5579.

149. Li X, Xie L Z, Li J, Chen G D and Aisa H. AkberA pair of new tetrahydro-naphthalenone enantiomers from Eremurus altaicus (Pall.) Stev." Phytochem Lett. 2015;**13**: 330-333.

150. Sun J, Yu J-H, Zhang J-S, Song X-Q, Bao J and Zhang H. Chromane enantiomers from the flower buds of Tussilago farfara L. and assignments of their absolute configurations. *Chem. Biodiversity.* 2019;**16**:n/a.

151. Qiao M-M, Liu F, Liu Y, Guo L, Zhou Q-M, Peng C, et al. Curcumane C and (±)-curcumane D, an unusual seco-cadinane sesquiterpenoid and a pair of unusual nor-bisabolane enantiomers with significant vasorelaxant activity from Curcuma longa. *Bioorg Chem.* 2019;**92**:103275.

152. Liu G, Zheng R-R, Liu Z-W, Wang W-J, Li G-Q, Fan C-L, et al. Enantiomeric chromones from Harrisonia perforata. *Phytochem. Lett.* 2014;**10**:295-299.

153. Yuan W-J, Gao W-F, Zhang J-H, Cao P, Zhang Y, Chen D-Z, et al. (±)-Perforison A, A Pair of New Chromone Enantiomers from Harrisonia perforata. *Nat Prod Commun.* 2017;**12**:63-65.

154. Tang Y-Q, Li Y-Q, Xie Y-B, Zhang J-S, Li W, Lou L-L, et al. Evodialones A and B: Polyprenylated Acylcyclopentanone Racemates with a 3-Ethyl-1,1-diisopentyl-4-methylcyclopentane Skeleton from Evodia lepta. *J Nat Prod.* 2018;**81**:1483-1487.

155. Xu J-F, Han C, Xu Q-Q, Wang X-B, Zhao H-J, Xue G-M, et al. Isolation, chiral-phase resolution, and determination of the absolute configurations of a complete series of stereoisomers of a rearranged acetophenone with three stereocenters. *J Nat Prod.* 2019;**82**:1399-1404.

156. Li W, Rao L, Liu Y, He Q, Fan Y, You Y-X, et al. (±)-Meliviticines A and B: Rearranged prenylated acetophenone derivatives from Melicope viticina and their antimicrobial activity. *Bioorg Chem.* 2019;**90**:103099.

157. Wang W-J, Wang L, Huang X-J, Jiang R-W, Yang X-L, Zhang D-M, et al. Two pairs of new benzofuran enantiomers with unusual skeletons from Eupatorium chinense. *Tetrahedron Lett.* 2013;**54**:3321-3324.

158. Xu J-F, Zhao H-J, Wang X-B, Li Z-R, Luo J, Yang M-H, et al. (±)-Melicolones A and B, rearranged prenylated acetophenone stereoisomers with an unusual 9-oxatricyclo[3.2.1.13,8]nonane core from the leaves of Melicope ptelefolia. *Org Lett.* 2015;**17**:146-149.

159. Liu H, Wu Z-L, Huang X-J, Peng Y, Huang X, Shi L, et al. Evaluation of diarylheptanoid-terpene adduct enantiomers from Alpinia officinarum for neuroprotective activities. *J Nat Prod.* 2018;**81**:162-170.

160. Dong S-H, Nikolic D, Simmler C, Qiu F, van Breemen R B, Soejarto D D, et al. Diarylheptanoids from Dioscorea villosa (Wild Yam). *J Nat Prod.* 2012;**75**:2168-2177.

161. Zhou B, Yuan X J, Li J Y, Xu Y C, Li J, Yue J M, et al. (-)- and (+)-Securidanes A and B, Natural Triarylmethane Enantiomers: Structure and Bioinspired Total Synthesis. *Research (Wash D C).* 2018;**2018**:2674182.

162. Cao Y, Yao Y, Huang X-J, Oberer L, Wagner T, Guo J-M, et al. Four new selaginellin derivatives from Selaginella pulvinata: mechanism of racemization process in selaginellins with quinone methide. *Tetrahedron.* 2015;**71**:1581-1587.

163. Song X-Q, Zhu K, Yu J-H, Zhang Q, Zhang Y, He F, et al. New octadecanoid enantiomers from the whole plants of Plantago depressa. *Molecules.* 2018;**23**:1723/1721-1723/1710.

164. Song X-Q, Yu S-J, Zhang J-S, Yu J-H and Zhang H. New octadecanoid derivatives from the seeds of Ipomoea nil. *Chin J Nat Med.* 2019;**17**:303-307.
165. Zou J, Chen G-D, Zhao H, Huang Y, Luo X, Xu W, et al. Triligustilides A and B: Two Pairs of Phthalide Trimers from Angelica sinensis with a Complex Polycyclic Skeleton and Their Activities. *Org Lett.* 2018;**20**:884-887.
166. Li X-L, Zhao B-X, Huang X-J, Zhang D-M, Jiang R-W, Li Y-J, et al. (+)- and (-)-Cajanusine, a Pair of New Enantiomeric Stilbene Dimers with a New Skeleton from the Leaves of Cajanus cajan. *Org Lett.* 2014;**16**:224-227.
167. Ito T, Endo H, Oyama M and Iinuma M. Novel isolation of stilbenoids with enantiomeric and meso forms from a cyperus rhizome. *Phytochem Lett.* 2012;**5**:267-270.
168. Ito T, Endo H, Shinohara H, Oyama M, Akao Y and Iinuma M. Occurrence of stilbene oligomers in Cyperus rhizomes. *Fitoterapia.* 2012;**83**:1420-1429.
169. Zhang X, Feng Z-M, Yang Y-N, Jiang J-S and Zhang P-C. Phenolic acid derivatives from Ligusticum chuanxiong. *Phytochem Lett.* 2019;**33**:114-118.
170. Gao Y-P, Shen Y-H, Zhang S-D, Tian J-M, Zeng H-W, Ye J, et al. Incarvilleatone, a New Cyclohexylethanoid Dimer from Incarvillea younghusbandii and Its Inhibition against Nitric Oxide (NO) Release. *Org Lett.* 2012;**14**:1954-1957.
171. Zhou X-M, Zheng C-J, Chen G-Y, Zhang X-P, Song X-P, Li G-N, et al. Bioactive Phenanthrene and Bibenzyl Derivatives from the Stems of Dendrobium nobile. *J Nat Prod.* 2016;**79**:1791-1797.
172. Malebo H M, Kihampa C, Mgina C A, Sung'hwa F, Jonker S A, Nkunya M H H, et al. Antifungal Enantiomeric Styrylpyrones from Sanrafaelia ruffonammari and Ophrypetalum odoratum. *Nat Prod Bioprospect.* 2014;**4**:129-133.
173. He R, Huang X, Zhang Y, Wu L, Nie H, Zhou D, et al. Structural Characterization and Assessment of the Cytotoxicity of 2,3-Dihydro-1H-indene Derivatives and Coumarin Glucosides from the Bark of Streblus indicus. *J Nat Prod.* 2016;**79**:2472-2478.
174. Wei X, Feng C, Li X-H, Mao X-X, Rong L, Yu X, et al. Enantiomeric Polyketides from the Starfish-Derived Symbiotic Fungus Penicillium sp. GGF16-1-2. *Chem Biodivers.* 2019;**16**:e1900052.
175. Ma L-Y, Zhang H-B, Kang H-H, Zhong M-J, Liu D-S, Liu W-Z, et al. New Butenolides and Cyclopentenones from Saline Soil-Derived Fungus Aspergillus Sclerotiorum. *Molecules.* 2019;**24**.
176. He J-W, Wang C-X, Yang L, Chen G-D, Hu D, Guo L-D, et al. A Pair of New Polyketide Enantiomers from Three Endolichenic Fungal Strains Nigrospora sphaerica, Alternaria alternata, and Phialophora sp. *Nat Prod Commun.* 2016;**11**:829-831.
177. Li H, Jiang J, Liu Z, Lin S, Xia G, Xia X, et al. Peniphenones A-D from the mangrove fungus Penicillium dipodomyicola HN4-3A as inhibitors of Mycobacterium tuberculosis phosphatase MptpB. *J Nat Prod.* 2014;**77**:800-806.
178. Zhao C, Fu P, Zhang Y, Liu X, Ren F and Che Y. Sporulosol, a new ketal from the fungus Paraconiothyrium sporulosum. *Molecules.* 2018;**23**:1263/1261-1263/1269.
179. Cao J, Li X-M, Li X, Li H-L, Meng L-H and Wang B-G. New lactone and isocoumarin derivatives from the marine mangrove-derived endophytic fungus Penicillium coffeae MA-314. *Phytochem Lett.* 2019;**32**:1-5.
180. Liu X, Chen C, Zheng Y, Zhang M, Tong Q, Liu J, et al. (±)-Peniorthoesters A and B, Two

Pairs of Novel Spiro-Orthoester en-antiomers With an Unusual 1,4,6-Trioxaspi-ro[4.5]decane-7-One Unit From Penicillium minioluteum. *Front Chem.* 2018;**6**:605.
181. Lu X-J, Chen S-F, Xu X-W, Zhao D, Wang H-F, Bai J, et al. One pair of new cyclopentaisochromenone enantiomer from Alternaria sp. TNXY-P-1 and their cytotoxic activity. *J Asian Nat Prod Res.* 2018;**20**:328-336.
182. Tang J-W, Xu H-C, Wang W-G, Hu K, Zhou Y-F, Chen R, et al. (+)- And (-)-Alternarilactone A: Enantiomers with a Diepoxy-Cage-like Scaffold from an Endophytic Alternaria sp. *J Nat Prod.* 2019;**82**:735-740.
183. Wang Y, Wang X-B, Li T-X, Yang M-H and Kong L-Y. Bioactive metabolites from the endophytic fungus Alternaria alternata. *Fitoterapia.* 2014;**99**:153-158.
184. Intaraudom C, Bunbamrung N, Dramae A, Boonyuen N, Choowong W, Rachtawee P, et al. Chromone derivatives, R- and S- taeniolin, from the marine-derived fungus Taeniolella sp. BCC31839. *Nat Prod Res.* 2019;doi:10.1080/14786419.2019.1634710.
185. Wu Y-H, Xiao G-K, Chen G-D, Wang C-X, Hu D, Lian Y-Y, et al. Pericocins A-D, New Bioactive Compounds from Periconia sp. *Nat Prod Commun.* 2015;**10**:2127-2130.
186. Guo D-L, Li X-H, Feng D, Jin M-Y, Cao Y-M, Cao Z-X, et al. Novel Polyketides Produced by the Endophytic Fungus Aspergillus Fumigatus from Cordyceps Sinensis. *Molecules.* 2018;**23**.
187. Li E, Zhang F, Niu S, Liu X, Liu G and Che Y. A Spiro[chroman-3,7'-isochromene]-4,6'(8'H)-dione from the Cordyceps-Colonizing Fungus Fimetariella sp. *Org Lett.* 2012;**14**:3320-3323.
188. Li X-H, Han X-H, Qin L-L, He J-L, Cao Z-X, Guo D-L, et al. Isochromanes from Aspergillus fumigatus, an endophytic fungus from Cordyceps sinensis. *Nat Prod Res.* 2019;**33**:1870-1875.
189. Li W, Gao W, Zhang M, Li Y-L, Li L, Li X-B, et al. p-Terphenyl Derivatives from the Endolichenic Fungus Floricola striata. *J Nat Prod.* 2016;**79**:2188-2194.
190. Arunrattiyakorn P, Kuno M, Aree T, Laphookhieo S, Sriyatep T, Kanzaki H, et al. Biotransformation of β-Mangostin by an Endophytic Fungus of Garcinia mangostana to Furnish Xanthenes with an Unprecedented Heterocyclic Skeleton. *J Nat Prod.* 2018;**81**:2244-2250.
191. Liu Z, Chen S, Qiu P, Tan C, Long Y, Lu Y, et al. (+)- and (-)-Ascomlactone A: a pair of novel dimeric polyketides from a mangrove endophytic fungus Ascomycota sp. SK2YWS-L. *Org Biomol Chem.* 2017;**15**:10276-10280.
192. Liu Z, Qiu P, Li J, Chen G, Chen Y, Liu H, et al. Anti-inflammatory polyketides from the mangrove-derived fungus Ascomycota sp. SK2YWS-L. *Tetrahedron.* 2018;**74**:746-751.
193. Zhang X, Wu Z, Lai Y, Li D, Wang J, Luo Z, et al. (±)-Terreinlactone A, a pair of 3-substituted δ-lactone enantiomers derived from terrein from the fungus aspergillus terreus. *Chem Pharm Bull.* 2018;**66**:764-767.
194. Hu C-H, Zhou Y-H, Xie F, Li Y-L, Zhao Z-T and Lou H-X. Two new α-pyrone derivatives from an endolichenic fungus Tolypocladium sp. *J Asian Nat Prod Res.* 2017;**19**:786-792.
195. Sang X-N, Chen S-F, Chen G, An X, Li S-G, Lu X-J, et al. Two pairs of enantiomeric α-pyrone dimers from the endophytic fungus Phoma sp. YN02-P-3. *RSC Adv.* 2017;**7**:1943-1946.

196. Miyano R, Matsuo H, Nonaka K, Mokudai T, Niwano Y, Shiomi K, et al. Pochoniolides A and B, new antioxidants from the fungal strain Pochonia chlamydosporia var. spinulospora FKI-7537. *J Biosci Bioeng.* 2018;**126**:661-666.
197. Qi B, Liu X, Mo T, Li S-S, Wang J, Shi X-P, et al. Nitric oxide inhibitory polyketides from Penicillium chrysogenum MT-12, an endophytic fungus isolated from Huperzia serrata. *Fitoterapia.* 2017;**123**:35-43.
198. He F, Li X, Yu J H, Zhang X, Nong X, Chen G, et al. Secondary metabolites from the mangrove sediment-derived fungus Penicillium pinophilum SCAU037. *Fitoterapia.* 2019;**136**:104177.
199. Zang Y, Gong Y-H, Li X-W, Li X-N, Liu J-J, Chen C-M, et al. Canescones A-E: aromatic polyketide dimers with PTP1B inhibitory activity from Penicillium canescens. *Org Chem Front.* 2019;**6**:3274-3281.
200. Xu L-L, Chen H-L, Hai P, Gao Y, Xie C-D, Yang X-L, et al. (+)- And (-)-Preuisolactone A: A Pair of Caged Norsesquiterpenoidal Enantiomers with a Tricyclo[4.4.01,6.02,8]decane Carbon Skeleton from the Endophytic Fungus Preussia isomera. *Org Lett.* 2019;**21**:1078-1081.
201. Song R-Y, Liu Y, Liu R-H, Wang X-B, Li T-X, Kong L-Y, et al. Benzophenone derivatives from the plant endophytic fungus, Pestalotiopsis sp. *Phytochem. Lett.* 2017;**22**:189-193.
202. Meng L-H, Li X-M, Liu Y, Wang B-G, Meng L-H, Liu Y, et al. Isolation, Stereochemical Study, and Antioxidant Activity of Benzofuranone Derivatives from a Mangrove-derived Fungus Eurotium rubrum MA-150. *Chirality.* 2016;**28**:581-584.
203. Sun T-Y, Kuang R-Q, Chen G-D, Qin S-Y, Wang C-X, Hu D, et al. Three pairs of new isopentenyl dibenzo[b,e]oxepinone enantiomers from Talaromyces flavus, a wetland soil-derived fungus. *Molecules.* 2016;**21**:1184/1181-1184/1112.
204. Hammerschmidt L, Ola A, Mueller W E G, Lin W, Mandi A, Kurtan T, et al. Two new metabolites from the endophytic fungus Xylaria sp. isolated from the medicinal plant Curcuma xanthorrhiza. *Tetrahedron Lett.* 2015;**56**:1193-1197.
205. Liu H, Tan H, Wang W, Zhang W, Chen Y, Li S, et al. Cytorhizophins A and B, benzophenone-hemiterpene adducts from the endophytic fungus Cytospora rhizophorae. *Org Chem Front.* 2019;**6**:591-596.
206. Wu J-C, Hou Y, Xu Q, Fang J, Wu Q-X, Jin X-J, et al. (±)-Alternamgin, a Pair of Enantiomeric Polyketides, from the Endophytic Fungi Alternaria sp. MG1. *Org Lett.* 2019;**21**:1551-1554.
207. Zhong W-M, Wang J-F, Wei X-Y, Zeng Q, Chen X-Y, Xiang Y, et al. (+)- And (-)-Eurotone A: A pair of enantiomeric polyketide dimers from a marine-derived fungus Eurotium sp. SCSIO F452. *Tetrahedron Lett.* 2019;**60**:1600-1603.
208. Shaker S, Fan R-Z, Lan W-J and Li H-J. A pair of novel bisindole alkaloid enantiomers from marine fungus Fusarium sp. XBB-9. *Nat Prod Res.* 2019:1-7.
209. Liu W, Wang L, Wang B, Xu Y, Zhu G, Lan M, et al. Diketopiperazine and diphenylether derivatives from marine algae-derived Aspergillus versicolor OUCMDZ-2738 by epigenetic activation. *Mar Drugs.* 2019;**17**:6.
210. Cao J, Li X-M, Meng L-H, Konuklugil B, Li X, Li H-L, et al. Isolation and characterization of three pairs of indolediketopiperazine enantiomers containing infrequent N-methoxy substitution from the marine algal-derived endophytic fungus Acrostalagmus luteoalbus

TK-43. *Bioorg Chem.* 2019;**90**:103030.
211. Zhong W, Wang J, Wei X, Fu T, Chen Y, Zeng Q, et al. Three pairs of new spirocyclic alkaloid enantiomers from the marine-derived fungus Eurotium sp. SCSIO F452. *Front Chem. (Lausanne, Switz.).* 2019;**7**:350.
212. Gao H, Liu W, Zhu T, Mo X, Mandi A, Kurtan T, et al. Diketopiperazine alkaloids from a mangrove rhizosphere soil derived fungus Aspergillus effuses H1-1. *Org Biomol Chem.* 2012;**10**:9501-9506.
213. Cai R, Jiang H, Xiao Z, Liu Z, Lin S e, She Z, et al. (-)- and (+)-Asperginulin A, a Pair of Indole Diketopiperazine Alkaloid Dimers with a 6/5/4/5/6 Pentacyclic Skeleton from the Mangrove Endophytic Fungus Aspergillus sp. SK-28. *Org Lett.* 2019;**21**:9633-9636.
214. Chen G-D, Bao Y-R, Huang Y-F, Hu D, Li X-X, Guo L-D, et al. Three pairs of variecolortide enantiomers from Eurotium sp. with caspase-3 inhibitory activity. *Fitoterapia.* 2014;**92**:252-259.
215. Zhong W, Wang J, Wei X, Chen Y, Fu T, Xiang Y, et al. Variecolortins A-C, Three Pairs of Spirocyclic Diketopiperazine Enantiomers from the Marine-Derived Fungus Eurotium sp. SCSIO F452. *Org Lett.* 2018;**20**:4593-4596.
216. Han J, Liu C, Li L, Zhou H, Liu L, Bao L, et al. Decalin-Containing Tetramic Acids and 4-Hydroxy-2-pyridones with Antimicrobial and Cytotoxic Activity from the Fungus Coniochaeta cephalothecoides Collected in Tibetan Plateau (Medog). *J Org Chem.* 2017;**82**:11474-11486.
217. Li J, Wang W-X, Chen H-P, Li Z-H, He J, Zheng Y-S, et al. (±)-Xylaridines A and B, Highly Conjugated Alkaloids from the Fungus Xylaria longipes. *Org Lett.* 2019;**21**:1511-1514.
218. Li J, Wang W-X, Li Z-H, He J, Huang R, Zheng Y-S, et al. Xylaridines C and D, unusual thiopyranodipyridine alkaloids from the fungus Xylaria longipes. *Org Lett.* 2019;**21**:6145-6148.
219. Chen S, Jiang M, Chen B, Niaz S-I, He J, Liu L, et al. Penicamide A, A Unique N,N'-Ketal Quinazolinone Alkaloid from Ascidian-Derived Fungus Penicillium sp. 4829. *Mar Drugs.* 2019;**17**.
220. Yang H, Li F and Ji N. Alkaloids from an algicolous strain of Talaromyces sp. *Chin J Oceanol Limnol.* 2016;**34**:367-371.
221. Yang J-K, Gao T, Yang M-Y, Zhao G-Z, Zhu H-J, Cao F, et al. A pair of enantiomeric 5-oxabicyclic[4.3.0]lactam derivatives and one new polyketide from the marine-derived fungus Penicillium griseofulvum. *Nat Prod Res.* 2018;**32**:2366-2369.
222. Almeida C, Hemberger Y, Schmitt S M, Bouhired S, Natesan L, Kehraus S, et al. Marilines A-C: novel phthalimidines from the sponge-derived fungus Stachylidium sp. *Chemistry.* 2012;**18**:8827-8834.
223. Kong Z, Jing R, Geng Y, Ji J, Wu Y, Guo Y, et al. Trichodermadiones A and B from the solid culture of Trichoderma atroviride S361, an endophytic fungus in Cephalotaxus fortunei. *Fitoterapia.* 2018;**127**:362-366.
224. Wen H, Li Y, Liu X, Ye W, Yao X and Che Y. Fusagerins A-F, New Alkaloids from the Fungus Fusarium sp. *Nat. Prod. Bioprospect.* 2015;**5**:195-203.
225. Liu L, Chen X, Li D, Zhang Y, Li L, Guo L, et al. Bisabolane Sesquiterpenoids from the Plant Endophytic Fungus Paraconiothyrium brasiliense. *J Nat Prod.* 2015;**78**:746-753.
226. Jia Y-L, Wei M-Y, Guan F-F, Wang C-Y, Shao C-L, Jia Y-L, et al. (+)- and (-)-Pestaloxazine

A, a Pair of Antiviral Enantiomeric Alkaloid Dimers with a Symmetric Spiro[oxazinane-piperazinedione] Skeleton from Pestalotiopsis sp. *Org Lett.* 2015;**17**:4216-4219.

227. Yan Y-M, Zhang H-X, Liu H, Wu J-B, Li Y-P, Cheng Y-X, et al. (+/-)-Lucidumone, a COX-2 Inhibitory Caged Fungal Meroterpenoid from Ganoderma lucidum. *Org Lett.* 2019;**21**:8523-8527.
228. Peng X-R, Liu J-Q, Wan L-S, Li X-N, Yan Y-X and Qiu M-H. Four New Polycyclic Meroterpenoids from Ganoderma cochlear. *Org Lett.* 2014;**16**:4838-4841.
229. Yan Y-M, Ai J, Zhou L-L, Chung A C K, Li R, Nie J, et al. Lingzhiols, Unprecedented Rotary Door-Shaped Meroterpenoids as Potent and Selective Inhibitors of p-Smad3 from Ganoderma lucidum. *Org Lett.* 2013;**15**:5488-5491.
230. Chen X, Chen L, Li S and Zhao J. Meroterpenoids from the fruiting bodies of higher fungus Ganoderma resinaceum. *Phytochem Lett.* 2017;**22**:214-218.
231. Luo Q, Di L, Yang X-H and Cheng Y-X. Applanatumols A and B, meroterpenoids with unprecedented skeletons from Ganoderma applanatum. *RSC Adv.* 2016;**6**:45963-45967.
232. Luo Q, Wang X-L, Di L, Yan Y-M, Lu Q, Yang X-H, et al. Isolation and identification of renoprotective substances from the mushroom Ganoderma lucidum. *Tetrahedron.* 2015;**71**:840-845.
233. Wang X-F, Yan Y-M, Wang X-L, Ma X-J, Fu X-Y and Cheng Y-X. Two new compounds from Ganoderma lucidum. *J Asian Nat Prod Res.* 2015;**17**:329-332.
234. Luo Q, Tu Z-C and Cheng Y-X. Two rare meroterpenoidal rotamers from Ganoderma applanatum. *RSC Adv.* 2017;**7**:3413-3418.
235. Cao W-W, Luo Q, Cheng Y-X and Wang S-M. Meroterpenoid enantiomers from Ganoderma sinensis. *Fitoterapia.* 2016;**110**:110-115.
236. Wang M, Wang F, Xu F, Ding L-Q, Zhang Q, Li H-X, et al. Two pairs of farnesyl phenolic enantiomers as natural nitric oxide inhibitors from Ganoderma sinense. *Bioorg Med Chem Lett.* 2016;**26**:3342-3345.
237. Li L, Li H, Peng X-R, Hou B, Yu M-Y, Dong J-R, et al. (±)-Ganoapplanin, a Pair of Polycyclic Meroterpenoid Enantiomers from Ganoderma applanatum. *Org Lett.* 2016;**18**:6078-6081.
238. Luo Q, Wang Z, Luo J-F, Tu Z-C and Cheng Y-X. (±)-Applanatumines B-D: novel dimeric meroterpenoids from Ganoderma applanatum as inhibitors of JAK3. *RSC Adv.* 2017;**7**:38037-38043.
239. Qin F-Y, Cheng Y-X, Qin F-Y, Cheng Y-X, Yan Y-M, Cheng Y-X, et al. (±) Gancochlearols A and B: cytotoxic and COX-2 inhibitory meroterpenoids from Ganoderma cochlear. *Nat Prod Res.* 2018:1-7.
240. Qin F-Y, Cheng Y-X, Qin F-Y, Cheng Y-X, Yan Y-M, Cheng Y-X, et al. (±) Cochlearoids N-P: three pairs of phenolic meroterpenoids from the fungus Ganoderma cochlear and their bioactivities. *J Asian Nat Prod Res.* 2019;**21**:542-550.
241. Zhou F-J, Nian Y, Yan Y, Gong Y, Luo Q, Zhang Y, et al. Two new classes of T-type calcium channel inhibitors with new chemical scaffolds from Ganoderma cochlear. *Org Lett.* 2015;**17**:3082-3085.
242. Li X-C, Liu F, Su H-G, Guo L, Zhou Q-M, Huang Y-J, et al. Two pairs of alkaloid enantiomers from Ganoderma luteomarginatum. *Biochem Syst Ecol.* 2019;**86**:103930.

243. Nord C, Menkis A and Broberg A. Cytotoxic illudane sesquiterpenes from the fungus Granulobasidium vellereum (Ellis and Cragin) Jülich. *J Nat Prod.* 2015;**78**:2559-2564.
244. Zhang S, Zhang L, Fu X, Li Z, Guo L, Kou L, et al. (+)- and (-)-actinoxocine, and actinaphthorans A-B, C-ring expansion and cleavage angucyclinones from a marine-derived Streptomyces sp. *Org Chem Front.* 2019;**6**:3925-3928.
245. Yi W, Li Q, Song T, Chen L, Li X-C, Zhang Z, et al. Isolation, structure elucidation, and antibacterial evaluation of the metabolites produced by the marine-sourced Streptomyces sp. ZZ820. *Tetrahedron.* 2019;**75**:1186-1193.
246. Zhang S, Yang Q, Guo L, Zhang Y, Feng L, Zhou L, et al. Isolation, structure elucidation and racemization of (+)- and (-)-pratensilins A-C: unprecedented spiro indolinone-naphthofuran alkaloids from a marine Streptomyces sp. *Chem Commun. (Cambridge, U. K.).* 2017;**53**:10066-10069.
247. Liang Y-Q, Liao X-J, Zhao B-X, Xu S-H, Liang Y-Q and Xu S-H. (+)- and (-)-Spongiterpene, a pair of new valerenane sesquiterpene enantiomers from the marine sponge Spongia Sp. *Nat Prod Res.* 2019:1-6.
248. Sun D-Y, Han G-Y, Yang N-N, Lan L-F, Li X-W and Guo Y-W. Racemic trinorsesquiterpenoids from the Beihai sponge Spongia officinalis: structure and biomimetic total synthesis. *Org Chem Front.* 2018;**5**:1022-1027.
249. Zhang X, Li P-L, Qin G-F, Li S, de Voogd N J, Tang X-L, et al. Isolation and absolute configurations of diversiform C17, C21 and C25 terpenoids from the marine sponge Cacospongia sp. *Mar Drugs.* 2019;**17**:14.
250. Jiao W-H, Hong L-L, Sun J-B, Piao S-J, Chen G-D, Deng H, et al. (±)-Hippolide J - A Pair of Unusual Antifungal Enantiomeric Sesterterpenoids from the Marine Sponge Hippospongia lachne. *Eur J.Org Chem.* 2017;**2017**:3421-3426.
251. Afifi A H, Kagiyama I, El-Desoky A H, Kato H, Mangindaan R E P, de Voogd N J, et al. Sulawesins A-C, Furanosesterterpene Tetronic Acids That Inhibit USP7, from a Psammocinia sp. Marine Sponge. *J Nat Prod.* 2017;**80**:2045-2050.
252. Zhu Y, Wang Y, Gu B-B, Yang F, Jiao W-H, Hu G-H, et al. Antifungal bromopyrrole alkaloids from the South China Sea sponge Agelas sp. *Tetrahedron.* 2016;**72**:2964-2971.
253. Allen E E, Zhu C, Panek J S and Schaus S E. Multicomponent Condensation Reactions via ortho-Quinone Methides. *Org Lett.* 2017;**19**:1878-1881.
254. Chu M-J, Tang X-L, Qin G-F, Sun Y-T, Li L, de Voogd N J, et al. Pyrrole Derivatives and Diterpene Alkaloids from the South China Sea Sponge Agelas nakamurai. *Chem Biodiversity.* 2017;**14**:n/a.
255. Wang Q, Tang X, Luo X, de Voogd N J, Li P and Li G. (+)- and (-)-Spiroreticulatine, A Pair of Unusual Spiro Bisheterocyclic Quinoline-imidazole Alkaloids from the South China Sea Sponge Fascaplysinopsis reticulata. *Org Lett.* 2015;**17**:3458-3461.
256. Shirouzu T, Watari K, Ono M, Koizumi K, Saiki I, Tanaka C, et al. Structure, synthesis, and biological activity of a C-20 bisacetylenic alcohol from a marine sponge Callyspongia sp. *J Nat Prod.* 2013;**76**:1337-1342.
257. Jang K H, Lee Y, Sim C J, Oh K-B and Shin J. Bioactive lipids from the sponge Spirastrella abata. *Bioorg Med Chem Lett.* 2012;**22**:1078-1081.
258. Alam N, Wang W, Hong J, Lee C-O, Im K S and Jung J H. Cytotoxic sphingosine 4-sulfates from the sponge Spirastrella abata. *J Nat Prod.* 2002;**65**:944-945.

259. Yan Y-M, Li L-J, Qin X-C, Lu Q, Tu Z-C and Cheng Y-X. Compounds from the insect Blaps japanensis with COX-1 and COX-2 inhibitory activities. *Bioorg Med Chem Lett.* 2015;**25**:2469-2472.
260. Yan Y-M, Ai J, Shi Y-N, Zuo Z-L, Hou B, Luo J, et al. (±)-Aspongamide A, an N-Acetyldopamine Trimer Isolated from the Insect Aspongopus chinensis, Is an Inhibitor of p-Smad3. *Org Lett.* 2014;**16**:532-535.
261. Zhu H-J, Yan Y-M, Tu Z-C, Luo J-F, Liang R, Yang T-H, et al. Compounds from Polyphaga plancyi and their inhibitory activities against JAK3 and DDR1 kinases. *Fitoterapia.* 2016;**114**:163-167.
262. Chan S T S, Nani R R, Schauer E A, Martin G E, Williamson R T, Sauri J, et al. Characterization and Synthesis of Eudistidine C, a Bioactive Marine Alkaloid with an Intriguing Molecular Scaffold. *J Org Chem.* 2016;**81**:10631-10640.
263. Wang L, Ma Y-T, Sun Q-Y, Li X-N, Yan Y, Yang J, et al. Structurally diversified diterpenoids from Euphorbia dracunculoides. *Tetrahedron.* 2015;**71**:5484-5493.
